# Dabrafenib plus trametinib in *BRAFV600E*-mutated rare cancers: the phase 2 ROAR trial

**DOI:** 10.1038/s41591-023-02321-8

**Published:** 2023-04-14

**Authors:** Vivek Subbiah, Robert J. Kreitman, Zev A. Wainberg, Anas Gazzah, Ulrik Lassen, Alexander Stein, Patrick Y. Wen, Sascha Dietrich, Maja J. A. de Jonge, Jean-Yves Blay, Antoine Italiano, Kan Yonemori, Daniel C. Cho, Filip Y. F. L. de Vos, Philippe Moreau, Elena Elez Fernandez, Jan H. M. Schellens, Christoph C. Zielinski, Suman Redhu, Aislyn Boran, Vanessa Q. Passos, Palanichamy Ilankumaran, Yung-Jue Bang

**Affiliations:** 1 Department of Investigational Cancer Therapeutics, Division of Cancer Medicine, The University of Texas MD Anderson Cancer Center, Houston, TX USA; 2 Laboratory of Molecular Biology, National Institutes of Health, Bethesda, MD USA; 3 Department of Medicine, University of California, Los Angeles, Los Angeles, CA USA; 4 Drug Development Department (DITEP), Gustave Roussy Cancer Institute, Villejuif, France; 5 Department of Oncology, Rigshospitalet, University of Copenhagen, Copenhagen, Denmark; 6 Department of Internal Medicine II (Oncology Center), University Medical Center Hamburg-Eppendorf, Hamburg, Germany; 7 Center for Neuro-Oncology, Dana-Farber Cancer Institute, Boston, MA USA; 8 University of Heidelberg, Heidelberg, Germany; 9 Department of Medical Oncology, Erasmus MC Cancer Institute, Rotterdam, The Netherlands; 10 Center Leon Berard & University Claude Bernard Lyon I, Lyon, France; 11 Early Phase Trials and Sarcoma Units, Institut Bergonié, Bordeaux, France; Faculty of Medicine, University of Bordeaux, Bordeaux, France; 12 National Cancer Center Hospital, Tokyo, Japan; 13 New York Medical College, Valhalla, NY USA; 14 Department of Medical Oncology, University Medical Center Utrecht, University Utrecht, Utrecht, The Netherlands; 15 Department of Hematology, CHU de Nantes, Nantes, France; 16 Department of Medical Oncology, Vall d’Hebron University Hospital (HUVH), Vall d’Hebron Institute of Oncology (VHIO), IOB-Quiron, UVic-UCC, Barcelona, Spain; 17 Netherlands Cancer Institute, Amsterdam, The Netherlands; 18 Medical University of Vienna, Vienna, Austria; 19 Global Program Biostatistics, Novartis Oncology, Cambridge, MA USA; 20 Global Drug Development, Oncology Development Unit, Novartis Services, Inc., East Hanover, NJ USA; 21 Seoul National University College of Medicine, Seoul, Republic of Korea

**Keywords:** Clinical trial design, Outcomes research

## Abstract

*BRAFV600E* alterations are prevalent across multiple tumors. Here we present final efficacy and safety results of a phase 2 basket trial of dabrafenib (BRAF kinase inhibitor) plus trametinib (MEK inhibitor) in eight cohorts of patients with *BRAFV600E*-mutated advanced rare cancers: anaplastic thyroid carcinoma (*n* = 36), biliary tract cancer (*n* = 43), gastrointestinal stromal tumor (*n* = 1), adenocarcinoma of the small intestine (*n* = 3), low-grade glioma (*n* = 13), high-grade glioma (*n* = 45), hairy cell leukemia (*n* = 55) and multiple myeloma (*n* = 19). The primary endpoint of investigator-assessed overall response rate in these cohorts was 56%, 53%, 0%, 67%, 54%, 33%, 89% and 50%, respectively. Secondary endpoints were median duration of response (DoR), progression-free survival (PFS), overall survival (OS) and safety. Median DoR was 14.4 months, 8.9 months, not reached, 7.7 months, not reached, 31.2 months, not reached and 11.1 months, respectively. Median PFS was 6.7 months, 9.0 months, not reached, not evaluable, 9.5 months, 5.5 months, not evaluable and 6.3 months, respectively. Median OS was 14.5 months, 13.5 months, not reached, 21.8 months, not evaluable, 17.6 months, not evaluable and 33.9 months, respectively. The most frequent (≥20% of patients) treatment-related adverse events were pyrexia (40.8%), fatigue (25.7%), chills (25.7%), nausea (23.8%) and rash (20.4%). The encouraging tumor-agnostic activity of dabrafenib plus trametinib suggests that this could be a promising treatment approach for some patients with *BRAFV600E*-mutated advanced rare cancers. ClinicalTrials.gov registration: NCT02034110.

## Abstract (web summary)

In the final analysis of all cohorts in the phase 2 ROAR basket trial, dabrafenib plus trametinib exhibited tumor-agnostic clinical activity in patients with rare *BRAFV800E*-mutated cancers, including anaplastic thyroid carcinoma, biliary tract cancer, low-grade glioma and hairy cell leukemia.

## Main

The *RAS/RAF/MEK/ERK* pathway (also called the mitogen-activated protein kinase (MAPK) pathway) is important in cancer biology. The role of this pathway has been described in cellular proliferation, migration, survival, angiogenesis and cell cycle regulation^1^. Alterations in B-Raf proto-oncogene (*BRAF*), a serine/threonine kinase, can constitutively activate the pathway, and *BRAF* mutations are seen in various cancers^2^. The most common *BRAF* mutation is the *V600E* mutation with substitution of valine (V) to glutamic acid (E) at position 600 of the amino acid sequence caused by transversion T → A at nucleotide 1799 (T1799A).

*BRAF* mutations are detected in ~7–15% of all cancers, and the most common locus of the mutation is at position V600. The mutation is seen in diverse cancers, including hairy cell leukemia (HCL, 79–100%), melanomas (40–70%), papillary thyroid cancers (45%), ovarian cancers (35%), cholangiocarcinomas (5–7%), multiple myeloma (MM, 4%) and non-small cell lung cancers (NSCLCs, 1–3%)^3,4^. *BRAFV600* mutations are also seen in rare and very rare cancers, such as gliomas, sarcomas, gastric and esophageal cancer, neuroendocrine cancer and ampullary cancer, among others^3^.

Genome-driven treatment options/agnostic treatments are evolving in oncology with advances in tropomyosin receptor kinase inhibition^5^, rearranged during transfection receptor tyrosine kinase inhibition^6,7^, and the treatment of tumors with high microsatellite instability^8^ or tumor mutation burden (TMB > 10)^9^. Dabrafenib selectively inhibits the mutated *BRAF* kinase, and trametinib shows a reversible, highly selective, allosteric inhibition of *MEK*1 and *MEK*2 activation and kinase activity. The combination of dabrafenib plus trametinib blocks oncogenic MAPK pathway signaling, inhibits growth and survival of *BRAFV600*-mutant cells and enhances anti-tumor activity versus either agent alone^10^. This combination therapy is approved for use in the so-called *BRAFV600E* anchor tumor types, namely metastatic melanoma, melanoma in adjuvant setting, NSCLC and anaplastic thyroid cancer (ATC)^11–14^. However, beyond these cancers, as *BRAFV600* mutations are prevalent in a long list of tumor types (>40 tumor types)^15^, there continued to be an unmet need for treatment of rare cancers in adults and children with *BRAFV600E* mutations where limited or no effective treatment options existed. These tumors lead to substantial burden and mortality in the relapsed or refractory settings.

The US Food and Drug Administration (FDA) granted an accelerated approval to dabrafenib (Tafinlar) plus trametinib (Mekinist) for the treatment of unresectable or metastatic solid tumors with a *BRAFV600E* mutation. The combination was approved for patients aged 6 years and older in whom the tumors progressed after prior treatment and who had no alternative treatment options. This approval was supported by the meaningful efficacy and safety for the combination in the Rare Oncology Agnostic Research (ROAR) and National Cancer Institute-Molecular Analysis for Therapy Choice (NCI-MATCH, NCT02465060) studies in adults and a study (NCT02124772) in pediatric patients with refractory or recurrent solid tumors. This was the first approval for a tumor-agnostic *BRAF* and *MEK* inhibitor combination approach and was a considerable advance in precision medicine.

The ROAR study was designed to assess the activity and safety of dabrafenib plus trametinib in patients with *BRAFV600E*-mutated rare cancers. Based on the discretion of the treating physician and according to the local available standards of care, these cancers did not have any satisfactory treatment options. Rare cancer types included in the study were ATC, biliary tract cancer (BTC), gastrointestinal stromal tumor (GIST), adenocarcinoma of the small intestine (ASI), low-grade (World Health Organization (WHO) grade 1 or grade 2) glioma (LGG), high-grade (WHO grade 3 or grade 4) glioma (HGG), non-seminomatous germ cell tumors (NSGCTs)/non-germinomatous germ cell tumors (NGGCTs), HCL and MM. We previously published interim results for the ATC^14^, BTC^16^, LGG and HGG^17^ and HCL cohorts (data cutoff dates: 26 August 2016 to 25 July 2018)^18^. Here we report the final results of the ROAR study for all cancer cohorts that were part of the study (data cutoff date: 10 December 2021). We also discuss multiple non-melanoma cancer studies showing evidence of the actionability of *BRAF* beyond anchor type cancers.

## Results

**Extended Data Fig. 1:**
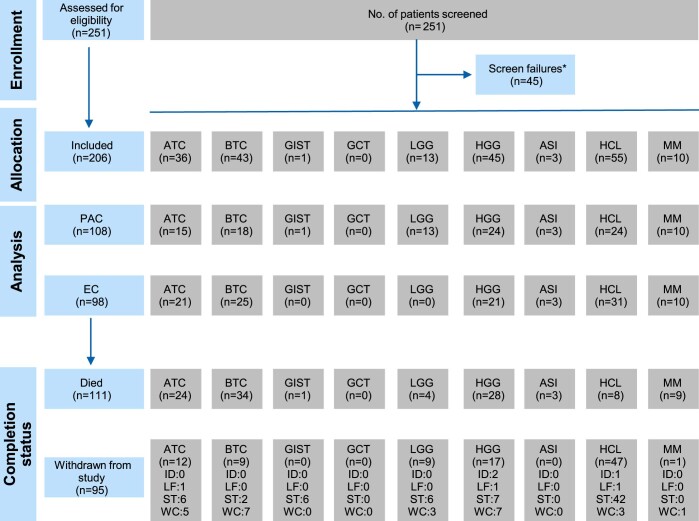
Patient disposition. *Reasons for screen failures were not collected ASI, adenocarcinoma of small intestine; ATC, anaplastic thyroid cancer; BTC, biliary tract cancer; DP, declined to participate; EC, expansion cohort; GCT, germ cell tumor; GIST, gastrointestinal stromal tumor; G, grade; GVHD, graft versus host disease; HCL, hairy cell leukemia; HGG, high (WHO G3/G4) grade glioma; ID, investigator discretion; LF, lost to follow-up; LGG, low (WHO G1/G2) grade glioma; MM, multiple myeloma; NIC, not meeting inclusion criteria; OR, other reasons; PAC, primary analysis cohort; ST, study terminated by sponsor; WC, withdrew consent; WHO, World Health Organization.

From 17 April 2014 to 25 July 2018, a total of 251 patients were screened and 206 patients were enrolled in the study across the ATC (*n* = 36), BTC (*n* = 43), GIST (*n* = 1), LGG (*n* = 13), HGG (*n* = 45), ASI (*n* = 3), NSGCT/NGGCT (*n* = 0), HCL (*n* = 55) and MM (*n* = 10) cohorts. The primary analysis cohort included a total of 108 patients: ATC (*n* = 15), BTC (*n* = 18), GIST (*n* = 1), LGG (*n* = 13), HGG (*n* = 24), ASI (*n* = 3), HCL (*n* = 24) and MM (*n* = 10); the expansion cohort included 98 patients: ATC (*n* = 21), BTC (*n* = 25), GIST (*n* = 0), LGG (*n* = 0), HGG (*n* = 21), ASI (*n* = 0), HCL (*n* = 31) and MM (*n* = 0) (Extended Data Fig. 1). B*RAFV600E* mutation status was confirmed by a central laboratory in 92%, 91%, 62%, 93% and 91% of patients in the ATC, BTC, LGG, HGG and HCL cohorts, respectively, and all patients in the GIST, ASI and MM cohorts. Baseline characteristics of the 206 patients are summarized in Table 1 (Supplementary Table 1). The median age was 60.5 years (range, 18–89 years), with 41% of patients ≥65 years of age. Most patients were male (56%) and White (78%). Eastern Cooperative Oncology Group (ECOG) performance status of 0, 1 and 2 was reported in 35%, 57% and 8% of patients, respectively.

**Table 1:** Demographic and baseline characteristics

	ATC (*n* = 36)	BTC (*n* = 43)	GIST (*n* = 1)	LGG (*n* = 13)	HGG (*n* = 45)	ASI (*n* = 3)	HCL (*n* = 55)	MM (*n* = 10)	Total (*n* = 206)
Age (years)									
Mean (s.d.)	69.6 (9.53)	57.0 (11.88)	77.0 ()	33.1 (11.51)	41.9 (14.70)	58.3 (3.21)	64.8 (10.77)	66.9 (6.89)	57.1 (16.40)
Age group (years)									
<18	0	0	0	0	0	0	0	0	0
18–64	9 (25%)	29 (67%)	0	13 (100%)	43 (96%)	3 (100%)	21 (38%)	4 (40%)	122 (59%)
65–74	13 (36%)	13 (30%)	0	0	2 (4%)	0	24 (44%)	5 (50%)	57 (28%)
75–84	12 (33%)	1 (2%)	1 (100%)	0	0	0	9 (16%)	1 (10%)	24 (12%)
≥85	2 (6%)	0	0	0	0	0	1 (2%)	0	3 (1%)
Sex									
Male	16 (44%)	19 (44%)	0	4 (31%)	23 (51%)	2 (67%)	47 (85%)	5 (50%)	116 (56%)
Female	20 (56%)	24 (56%)	1 (100%)	9 (69%)	22 (49%)	1 (33%)	8 (15%)	5 (50%)	90 (44%)
Baseline ECOG									
0	4 (11%)	17 (40%)	1 (100%)	5 (38%)	14 (31%)	3 (100%)	25 (45%)	3 (30%)	72 (35%)
1	30 (83%)	24 (56%)	0	7 (54%)	24 (53%)	0	27 (49%)	6 (60%)	118 (57%)
2	2 (6%)	2 (5%)	0	1 (8%)	7 (16%)	0	3 (5%)	1 (10%)	16 (8%)
Time since diagnosis (days)	125.0	347.0	325.0	2536.0	525	595	4578	2359	—
Median (range)	(14.0–4,606.0)	(26.0–3,224.0)	(−)	(45–9,367)	(59–9,549)	(147–1,014)	(88–12,126)	(1,107–5,740)	
Measurable disease at screening	36 (100)	43 (100)	1 (100)	13 (100)	43 (96%)	3 (100%)	—	—	—
Non-target lesions at screening	29 (81)	31 (72)	0	3 (23)	10 (22%)	1 (33%)	—	—	—
Stage, *n* (%)									
I	0	0	0	6 (46)	0	0		—	—
II	0	1 (2)	0	7 (54)	0	0		—	—
III	0	0	0	0	13 (29%)	0		—	—
IV	1 (3)	1 (2)	1 (100)	0	31 (69%)	2 (67%)	—	—	—
IVA	0	0	0	—	—	1 (33%)	—	—	—
IVB	0	40 (93)	0	—	—	0	−	—	—
IVC	35 (97)	0	0	—	—	0	—	—	—
Missing	0	1 (2)	0	0	1 (2%)	0	—	—	—
Prior radiotherapy regimens									
0	7 (19)	38 (88)	1 (100)	5 (38)	1 (2%)	3 (100%)	—	—	—
1	18 (50)	4 (9)	0	7 (54)	36 (80%)	0	—	—	—
2	11 (31)	1 (2)	0	1 (8)	7 (16%)	0	—	—	—
3	0	0	0	0	1 (2%)	0	—	—	—
Prior anti-cancer therapy									
Any therapy	36 (100)	43 (100)	1 (100)	12 (92)	45 (100)	3 (100)	55 (100)	10 (100)	205 (>99)
Biologic therapy	0	5 (12)	0	2 (15)	7 (16)	2 (67)	45 (82)	4 (40)	65 (32)
Chemotherapy	15 (42)	42 (98)	0	5 (38)	42 (93)	3 (100)	55 (100)	10 (100)	172 (83)
Hormonal therapy	0	0	0	0	0	0	0	0	0
Immunotherapy	4 (11)	2 (5)	0	0	1 (2)	0	15 (27)	10 (100)	32 (16)
Radioactive therapy	11 (31)	0	0	0	0	0	0	0	11 (5)
Small molecule targeted therapy	7 (19)	3 (7)	1 (100)	0	3 (7)	0	5 (9)	10 (100)	29 (14)
Radiotherapy	30 (83)	5 (12)	0	8 (62)	44 (98)	0	1 (2)	7 (70)	95 (46)
Surgery	30 (83)	24 (56)	1 (100)	12 (92)	42 (93)	3 (100)	6 (11)	3 (30)	121 (59)

Population is the efficacy evaluable population.

Values are for primary and expansion cohorts combined for all cancers except disease characteristics for GIST, LGG and ASI, which include the primary analysis cohort.

Patients received oral dabrafenib (150 mg twice daily) and oral trametinib (2 mg once daily) on a continuous dosing schedule until unacceptable toxicity, disease progression or death. Across all cohorts, the median duration of exposure to dabrafenib and trametinib was 12.5 months (range, 1–82 months) and 12.0 months (range, 1–84 months), respectively. Two-thirds of the patients received dabrafenib (66%) and trametinib (65%) for more than 6 months. The median daily dose of dabrafenib and trametinib was 282.1 mg (range, 16.9–315.5 mg) and 1.9 mg (range, 0.6–2 mg), respectively (a daily dose of 450 mg dabrafenib was administered in eight patients). A total of 111 patients (54%) died; 31% withdrew from the study due to the sponsor’s decision to terminate the study; and 13% withdrew consent. The most common reason for study treatment discontinuation was progressive disease (dabrafenib: 60%; trametinib: 59%) (Extended Data Fig. 1 and Extended Data Table 1).

## Primary endpoint

**Fig. 1:**
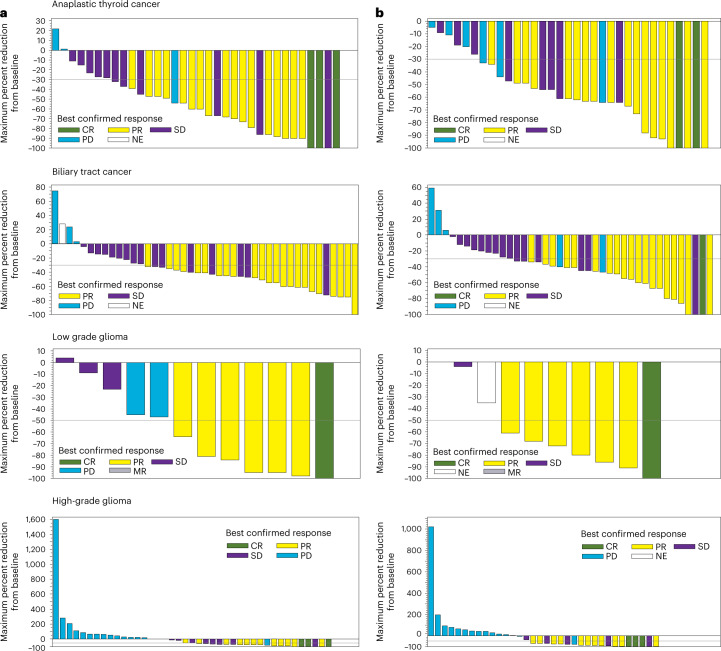
Waterfall plots for percentage of tumor reductions. Tumor reduction from baseline in patients with *BRAFV600E* mutation-positive rare cancers by investigator assessment (**a**) and by independent review (**b**). MR, minor response; NE, not evaluable; PD, progressive disease; SD, stable disease.

The primary endpoint was the investigator-assessed overall response rate (ORR) (see the section of study endpoints in Methods). As a supportive analysis defined in the study protocol, ORR was also assessed centrally by independent radiology review for the ATC, BTC, ASI, LGG and HGG cohorts. This helped to corroborate the results for ORR by investigator assessment. Concordance rates were determined for the best response by the investigator-assessed rates and those determined by independent radiology assessment. The ORR was ≥50% across all cohorts except the HGG cohort (ORR > 30%). Table 2 shows the best response and ORR in the study cohorts by investigator and independent radiology assessment. The GIST cohort enrolled only one patient. With exposure to dabrafenib plus trametinib for 30 months, the patient had stable disease as per the investigator assessment. The concordance rates for best response by investigator and independent radiology assessment were 66.7%, 58.1%, 66.7%, 46.2% and 66.7% for the ATC, BTC, ASI, LGG and HGG cohorts, respectively. Waterfall plots for percentage of tumor reductions in the ATC, BTC, LGG and HGG cohorts are shown in Fig. 1. Bayesian model-based results for ORR, along with the observed ORR (frequentist methodology) by investigator review, are shown in Supplementary Table 2.

**Table 2:** Best response and ORR in patient cohorts by investigator and independent radiology assessment

Cohorts	Investigator assessment						Independent radiology assessment					
	Best response						Best response					
	CR	PR	SD	PD	NE	RR	CR	PR	SD	PD	NE	RR
ATC (*n* = 36)	3 (8%)	17 (47%)	11 (31%)	4 (11%)	1 (3%)^a^	20 (56%) (38.1%, 72.1%)	2 (6%)	17 (47%)	8 (22%)	8 (22%)	1 (3%)^a^	19 (53%) (35.5%, 69.6%)
BTC (*n* = 43)	0	23 (53%)	16 (37%)	3 (7%)	1 (2%)^b^	23 (53%) (37.7%, 68.8%)	1 (2%)	19 (44%)	15 (35%)	6 (14%)	2 (5%)^c^	20 (47%) (31.2%, 62.3%)
ASI (*n* = 3)	—	2 (67%)	—	1 (33%)	—	67% (9.4%, 99.2%)	—	2 (67%)	—	1 (33%)	—	67% (9.4%, 99.2%)
LGG (*n* = 13)	1 (8%)	6 (46%)	3 (23%)	1 (8%)	0	7 (54%) (25.1%, 80.8%)	1 (8%)	6 (46%)	2 (15%)	0	3 (23%)^d^	7 (54%) (25.1%, 80.8%)
HGG (*n* = 45)	3 (7%)	12 (27%)	10 (22%)	20 (44%)	0	15 (33%) (20.0%, 49.0%)	3 (7%)	11 (24%)	5 (11%)	21 (47%)	5 (11%)^e^	14 (31%) (18.2%, 46.6%)
HCL (*n* = 55)	10 (18%) 26 (47%)^f^	13 (24%)	0	1 (2)	1 (2)^g^	49 (89%) (77.8%, 95.9%)	NA	NA	NA	NA	NA	NA
MM (*n* = 10)	0^h^	2 (20%)^i^	1 (10%)	4 (40%)	0	5 (50%) (18.7%, 81.3%)	NA	NA	NA	NA	NA	NA

In the LGG cohort, minor response was reported in two (15%) patients by investigator review and in one (8%) patient by independent radiology review. Minor response is not included in the calculated response rate. In the HCL cohort, minor response was reported in four (7%) patients. In the MM cohort, no patient showed minimal response.

ORRs were evaluated by RECIST 1.1 in the ATC and BTC cohorts, RANO criteria in the LGG and HGG cohorts and IMWG uniform response criteria for the MM cohort. The best response and RR criteria for HCL were adapted from NCCN guidelines, consensus resolution criteria and definitions in previous studies.

RR is CR + PR in the ATC, BTC, LGG and HGG cohorts. RR in the HCL cohort was calculated as (CR−MRD) + (CR + MRD) + PR. RR in the MM cohort was calculated as sCR + CR + VGPR + PR. The CIs are exact two-sided 95% CI based on the Clopper–Pearson method.

^a^No post-baseline assessments.

^b^Received anti-cancer therapy before disease progression observed (at first post-baseline assessment).

^c^No measurable disease at baseline.

^d^One patient had no measurable disease at baseline, and another one had no post-baseline assessments.

^e^One patient had no measurable disease at baseline, and three patients had no post-baseline assessments. All progressed by investigator assessment before first radiological assessment; one patient had an SD assessment, which was before the minimum 6 weeks after first dose of study treatment.

^f^Values show CR without and with MRD.

^g^One patient had no post-baseline assessments.

^h^No patient showed sCR in the MM cohort.

^i^VGPR was seen in three (30%) patients in the MM cohort.

MRD, minimal residual disease; NA, not available; NE, not evaluable; PD, progressive disease; RR, response rate; sCR, stringent complete response; SD, stable disease; VGPR, very good partial response.

## Secondary endpoints

### Duration of response

**Extended Data Fig. 2:**
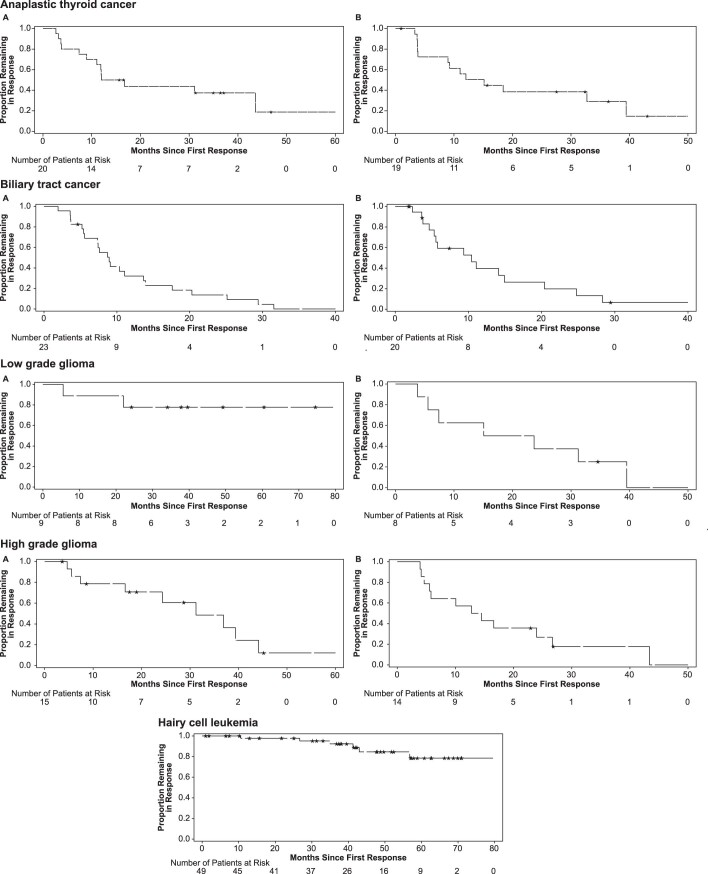
Duration of response by investigator and independent radiology assessment across tumor cohorts. Efficacy evaluable population; Primary and expansion cohorts; (A) Investigator assessment (B) Independent radiology assessment. **ATC:** Among 20 responders by investigator assessment, nine (45%) patients had disease progression and four (20%) died. Seven (35%) patients ended their follow-up and were censored. Among 19 responders by independent radiology assessment, 12 (63%) patients had disease progression and one (5%) died. Six (32%) patients ended their follow-up and were censored. **BTC:** Among 23 responders by investigator assessment, 20 (87%) patients had disease progression and 2 (9%) died. One (4%) patient was censored due to end of follow-up. Among the 20 responders by independent radiology assessment, 14 (70%) patients had disease progression and one (5%) died. Five (25%) patients were censored due to end of follow-up. **LGG:** Among nine responders by investigator assessment, two (22%) patients had disease progression. Seven (78%) patients were censored due to end of follow-up. Among eight responders by independent radiology assessment, seven (88%) patients had disease progression. One (13%) patient was censored due to end of follow-up. **HGG:** Among 15 responders by investigator assessment, nine (60%) patients had disease progression. Six (40%) patients were censored due to end of follow-up. Among 14 responders by independent radiology assessment, 12 (86%) patients had disease progression. Two (14%) patients were censored due to end of follow-up. **ASI:** Due to small numbers of responders, KM curves are not shown. Of the two responders by investigator assessment, one patient had disease progression and one was censored due to end of follow-up. Among the two responders by independent radiology assessment, both patients had disease progression. **HCL:** Among 49 responders by investigator assessment, four (8%) patients had disease progression and two (4%) patients died. Forty-three patients (88%) were censored due to end of follow-up. **MM:** Due to small numbers of responders, KM curves are not shown. Among five responders by investigator assessment, 4 (80%) patients had disease progression. One (20%) patient was censored due to end of follow-up. ASI, adenocarcinoma of small intestine; ATC, anaplastic thyroid cancer; BTC, biliary tract cancer; G, grade; HCL, hairy cell leukemia; HGG, high (WHO G3/G4) grade glioma; KM, Kaplan-Meier; LGG, low (WHO G1/G2) grade glioma; MM, multiple myeloma; WHO, World Health Organization.

Among responders, the duration of response (DoR) was defined as the time from complete response (CR) or partial response (PR) (ATC, BTC, LGG, HGG and ASI); CR with and without minimal residual disease or PR (HCL); or stringent CR, CR, PR or very good PR (MM) to disease progression or death. By investigator assessment, the median (95% confidence interval (CI)) DoR was 14.4 (7.4, not reached (NR)) months for ATC; 8.9 (5.6, 13.7) months for BTC; 7.7 (NR, NR) months for ASI; NR (5.5, NR) months for LGG; 31.2 (7.4, 44.2) months for HGG; and 11.1 (5.6, NR) months for MM cohorts. By independent radiology assessments, the DoR was 13.6 (3.8, 39.4); 10.4 (4.6, 14.9); 7.5 (7.4, NR); 19.4 (3.8, NR); and 13.6 (4.6, 26.7) months in the ATC, BTC, ASI, LGG and HGG cohorts, respectively. In the HCL cohort, median DoR was NR. The patient in the GIST cohort did not have a CR or a PR. Kaplan–Meier plots for DoR based on investigator and independent radiology assessment across tumor cohorts are shown in Extended Data Fig. 2.

### Progression-free survival

**Extended Data Fig. 3:**
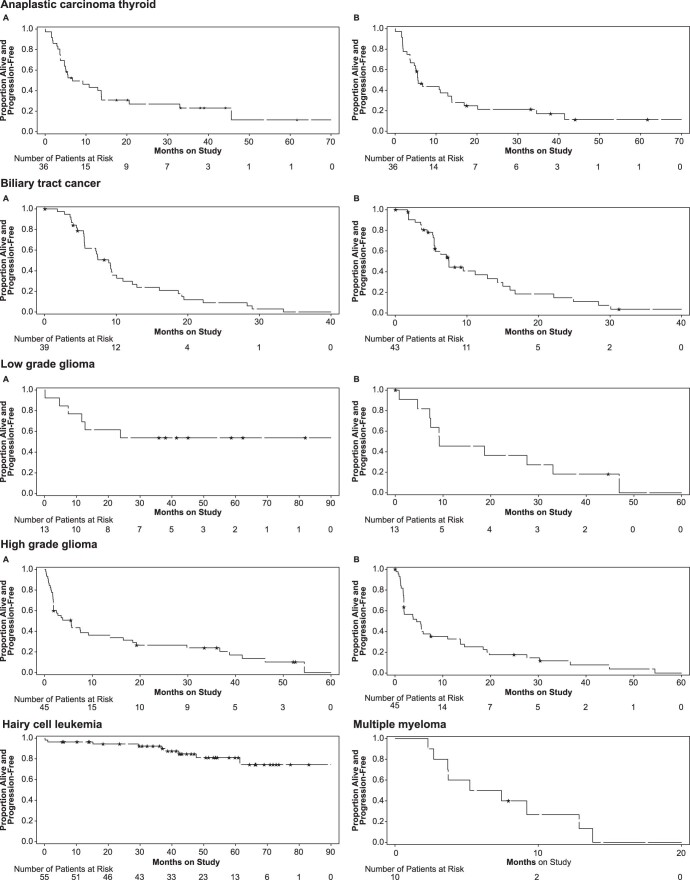
Progression free survival by investigator and independent radiology assessment across tumor cohorts. Efficacy evaluable population: Primary and expansion cohorts in all except low grade glioma and MM which is primary analysis cohort; (A) Investigator assessment (B) Independent radiology assessment. ATC: By investigator assessment in 36 patients, 21 (58%) patients had disease progression and six (17%) had died without disease progression. Nine (25%) patients were censored due to end of follow-up. By independent radiology review, progression of disease was observed in 26 (72%) patients and three (8%) patients died without disease progression. Seven (19%) patients were censored due to end of follow-up. BTC: By investigator assessment in 43 patients, 33 (77%) patients had disease progression and six (14%) patients had died without disease progression. Four patients were censored due to end of follow-up. By independent radiology review, progression of disease was observed in 27 (63%) patients and five (12%) patients died without disease progression. Eleven (26%) patients were censored due to end of follow-up. ASI: By investigator assessment in three patients, one patient (33%) had disease progression. Two (67%) patients were censored due to end of follow-up. By independent radiology review, progression of disease was observed in three (100%) patients. LGG: By investigator assessment in 13 patients, six (46%) patients had disease progression. Seven (54%) patients were censored due to end of follow-up. By independent radiology review, progression of disease was observed in nine (69%) patients and one (8%) patient died without disease progression. Three (23%) patients were censored due to end of follow-up. HGG: By investigator assessment in 45 patients, 38 (84%) patients had disease progression. Seven (16%) patients were censored due to end of follow-up. By independent radiology review, progression of disease was observed in 36 (80%) patients and four (9%) patients died without disease progression. Five (11%) patients were censored due to end of follow-up. HCL: By investigator assessment in 55 subjects, six (11%) patients had disease progression and three (5%) patients had died without disease progression. Forty-six (84%) patients were censored due to end of follow-up. MM: By investigator assessment in 10 patients, nine (90%) patients had disease progression. One patient was censored due to end of follow-up. ASI, adenocarcinoma of small intestine; ATC, anaplastic thyroid cancer; BTC, biliary tract cancer; G, grade; HCL, hairy cell leukemia; HGG, high (WHO G3/G4) grade glioma; LGG, low (WHO G1/G2) grade glioma; MM, multiple myeloma; WHO, World Health Organization.

By investigator assessment, the median (95% CI) progression-free survival (PFS) was 6.7 (4.7, 13.8), 9.0 (5.5, 9.4), 9.5, 5.5 (1.8, 13.7) and 6.3 (2.3, 12.9) months in the ATC, BTC, ASI, HGG and MM cohorts, respectively. Due to the small patient numbers, PFS could not be evaluated in the LGG (six patients had disease progression) and HCL (six patients had disease progression and three died) cohorts. By independent radiology assessment, the median (95% CI) PFS was 5.5 (3.7, 12.9), 7.5 (5.4, 12.9), 9.2 (0.9, NR), 9.2 (4.7, 33.0) and 4.5 (1.8, 7.4) months in the ATC, BTC, ASI, LGG and HGG cohorts, respectively. Kaplan–Meier plots for PFS by investigator and independent radiology assessment across tumor cohorts are shown in Extended Data Fig. 3.

### Overall survival

**Extended Data Fig. 4:**
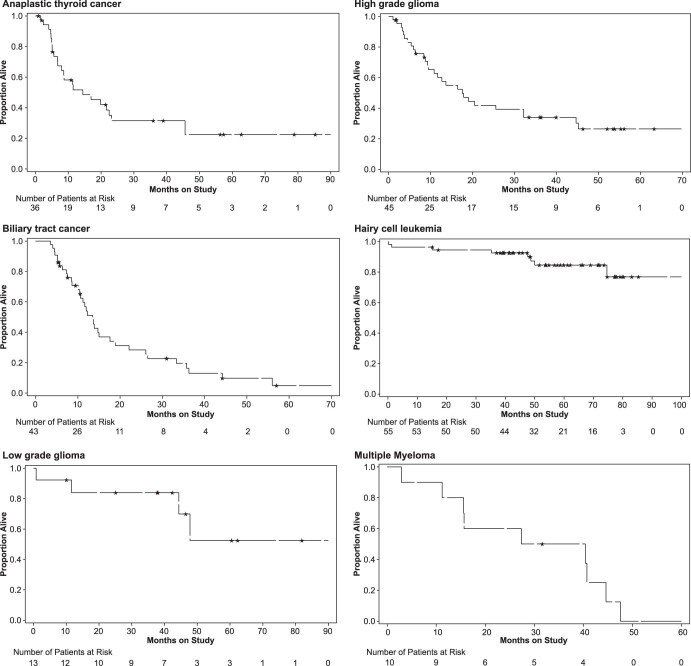
Overall survival across tumor cohorts. Efficacy evaluable population: Primary and expansion cohorts in all except low grade glioma and multiple myeloma which are primary analysis cohorts. ATC: Among 36 patients, 24 (67%) patients died, 12 (33%) patients were censored due to end of follow-up. BTC: Among 43 patients, 34 (79%) patients died, nine (21%) patients were censored due to end of follow-up. ASI: All three patients died during the study LGG: Among 13 patients (in the primary analysis cohort), four (31%) patients died, nine (69%) patients were censored due to end of follow-up. HGG: Among 45 patients, 28 (62%) patients died, 17 (38%) patients were censored due to end of follow-up. HCL: Among 55 patients, eight (15%) patients died, 47 (85%) patients were censored due to end of follow-up. MM: Among 10 patients, nine (90%) patients died, one patient was censored due to end of follow up. ASI, adenocarcinoma of small intestine; ATC, anaplastic thyroid cancer; BTC, biliary tract cancer; G, grade; HCL, hairy cell leukemia; HGG, high (WHO G3/G4) grade glioma; LGG, low (WHO G1/G2) grade glioma; MM, multiple myeloma; WHO, World Health Organization.

The median (95% CI) overall survival (OS) was 14.5 (6.8, 23.2), 13.5 (10.4, 17.6), 17.6 (9.5, 32.2) and 33.9 (2.9, 44.6) months in the ATC, BTC, HGG and MM cohorts, respectively. In the ASI cohort, the median OS was 21.8 (3.4, NR) months. All three patients in the ASI cohort died during the study. OS could not be estimated owing to the low numbers of deaths in the LGG (*n* = 4) and HCL (*n* = 8) cohorts; Kaplan–Meier plots for OS across tumor cohorts are shown in Extended Data Fig. 4.

## Safety

Regardless of relation to study treatment, adverse events (AEs) were reported in 201 (97.6%) patients across all cohorts. The most commonly (≥20% of patients) reported AEs were pyrexia (*n* = 113, 54.9%), fatigue (*n* = 87, 42.2%) and nausea (*n* = 86, 41.7%) (Table 3).

**Table 3:** Most frequent AEs (≥20%) by preferred term

Preferred terms	ATC (*n* = 36)	BTC (*n* = 43)	GIST (*n* = 1)	LGG (*n* = 13)	HGG (*n* = 45)	ASI (*n* = 3)	HCL (*n* = 55)	MM (*n* = 10)	Total (*n* = 206)
Any events	36 (100.0)	43 (100.0)	1 (100.0)	12 (92.3)	42 (93.3)	3 (100.0)	55 (100.0)	9 (90.0)	201 (97.6)
Pyrexia	17 (47.2)	29 (67.4)	1 (100.0)	8 (61.5)	11 (24.4)	2 (66.7)	42 (76.4)	3 (30.0)	113 (54.9)
Fatigue	13 (36.1)	14 (32.6)	0	8 (61.5)	20 (44.4)	0	29 (52.7)	3 (30.0)	87 (42.2)
Nausea	12 (33.3)	18 (41.9)	1 (100.0)	7 (53.8)	16 (35.6)	0	27 (49.1)	5 (50.0)	86 (41.7)
Chills	8 (22.2)	12 (27.9)	0	3 (23.1)	5 (11.1)	1 (33.3)	31 (56.4)	2 (20.0)	62 (30.1)
Headache	8 (22.2)	10 (23.3)	0	8 (61.5)	19 (42.2)	0	21 (38.2)	0	66 (32.0)
Constipation	8 (22.2)	9 (20.9)	1 (100.0)	4 (30.8)	8 (17.8)	1 (33.3)	24 (43.6)	3 (30.0)	58 (28.2)
Vomiting	7 (19.4)	15 (34.9)	1 (100.0)	4 (30.8)	13 (28.9)	1 (33.3)	14 (25.5)	3 (30.0)	58 (28.2)
Cough	4 (11.1)	10 (23.3)	0	3 (23.1)	8 (17.8)	0	30 (54.5)	1 (10.0)	56 (27.2)
Diarrhea	7 (19.4)	14 (32.6)	0	4 (30.8)	5 (11.1)	1 (33.3)	19 (34.5)	4 (40.0)	54 (26.2)
Rash	10 (27.8)	12 (27.9)	0	4 (30.8)	12 (26.7)	0	11 (20.0)	3 (30.0)	52 (25.2)
Increased AST increased	5 (13.9)	11 (25.6)	0	4 (30.8)	9 (20.0)	0	21 (38.2)	0	50 (24.3)
Anemia	13 (36.1)	10 (23.3)	0	4 (30.8)	9 (20.0)	0	10 (18.2)	3 (30.0)	49 (23.8)
Arthralgia	5 (13.9)	6 (14.0)	0	7 (53.8)	7 (15.6)	0	21 (38.2)	2 (20.0)	48 (23.3)
Hyperglycemia	5 (13.9)	8 (18.6)	0	2 (15.4)	5 (11.1)	0	26 (47.3)	0	46 (22.3)
Edema peripheral	5 (13.9)	4 (9.3)	0	2 (15.4)	3 (6.7)	1 (33.3)	27 (49.1)	2 (20.0)	44 (21.4)
Myalgia	2 (5.6)	8 (18.6)	0	2 (15.4)	7 (15.6)	0	25 (45.5)	1 (10.0)	45 (21.8)

Values are number of patients, *n* (%).

Preferred terms by MedDRA version 23.0 and CTCAE version 4.0.

All treated patients (primary and expansion cohorts).

AST, aspartate aminotransferase; MedDRA, Medical Dictionary for Regulatory Activities.

Across all cohorts, 181 (87.9%) patients reported AEs (of any grade) that were related to the study treatment, either dabrafenib or trametinib. The most frequent (≥10% of patients) treatment-related AEs were pyrexia (*n* = 84, 40.8%), fatigue (*n* = 53, 25.7%), chills (*n* = 52, 25.7%), nausea (*n* = 49, 23.8%) and rash (*n* = 42, 20.4%) (Extended Data Table 2). Regardless of relation to study drug, 122 (59.2%) patients had grade 3/4 AEs (grade 3: 50.5% and grade 4: 8.7%). Neutropenia was reported as the most frequent grade 3/4 AE in 15 (7.3%) patients, followed by anemia (6.3%) and pneumonia (5.3%). Nine (4.4%) patients had grade 5 AEs: one patient had sepsis, pneumonia and pleural effusion; one had sepsis and pneumonia; and the other seven had pulmonary embolism, general physical health deterioration, adenocarcinoma of the pancreas, diverticulitis, sepsis, sepsis and hemorrhagic stroke. None of these was related to the study drug. A summary of AEs (≥20% in all cohorts) by maximum toxicity grade is shown in Extended Data Table 3.

Adverse events of special interest (AESIs) included skin toxicity (*n* = 123, 59.7%), pyrexia (*n* = 113, 54.9%), hepatic disorders (*n* = 72, 35.0%), bleeding events (*n* = 62, 30.1%), neutropenia (*n* = 54, 26.2%), ocular events (*n* = 54, 26.2%), hyperglycemia (*n* = 50, 24.3%), new primary or secondary malignancy (*n* = 29, 14.1%), hypertension (*n* = 22, 10.7%), cardiac-related events (*n* = 20, 9.7%), hypersensitivity (*n* = 20, 9.7%), venous thromboembolism (*n* = 12, 5.8%), pre-renal and intrinsic renal failure (*n* = 8, 3.9%), pneumonitis and interstitial lung disease (*n* = 5, 2.4%), pancreatitis (*n* = 4, 1.9%) and uveitis (*n* = 4, 1.9%). AESIs in all cohorts are shown in Extended Data Table 4. There were three deaths in patients who had AESIs. One patient in the ATC cohort died of pulmonary embolism, and two patients in the HCL cohort died of adenocarcinoma of the pancreas and hemorrhagic stroke, respectively. None of these was related to the study drug.

### Serious adverse events

Across all cohorts, 93 (45.1%) patients had serious adverse events (SAEs) regardless of study treatment relationship. The most frequently reported (≥5 patients) SAEs were pyrexia (*n* = 23, 11.2%), pneumonia (*n* = 13, 6.3%), urinary tract infection (*n* = 8, 3.9%), vomiting (*n* = 7, 3.4%) and sepsis (*n* = 5, 2.4%) (Table 4). SAEs that were suspected to be related to the study treatment were seen in 46 (22.3%) patients. Pyrexia was the most frequent treatment-related SAE in 19 (9.2%) patients, followed by basal cell carcinoma, decreased neutrophil count, squamous cell carcinoma (*n* = 3, 1.5%), squamous cell carcinoma of the skin (*n* = 3, 1.5%) and chills, leukopenia, nausea and vomiting (*n* = 2, 1% each) (Extended Data Table 5).

**Table 4:** SAEs (≥2 patients across all cohorts) regardless of study drug relationship

Preferred term	ATC (*n* = 36)	BTC (*n* = 43)	GIST (*n* = 1)	LGG (*n* = 13)	HGG (*n* = 45)	ASI (*n* = 3)	HCL (*n* = 55)	MM (*n* = 10)	Total (*n* = 206)
Any event	20 (55.6%)	17 (39.5%)	1 (100.0%)	3 (23.1%)	16 (35.6%)	0	32 (58.2%)	4 (40.0%)	93 (45.1%)
Pyrexia	1 (2.8%)	9 (20.9%)	0	1 (7.7%)	1 (2.2%)	0	10 (18.2%)	1 (10.0%)	23 (11.2%)
Pneumonia	8 (22.2%)	1 (2.3%)	0	0	0	0	4 (7.3%)	0	13 (6.3%)
Urinary tract infection	2 (5.6%)	1 (2.3%)	0	1 (7.7%)	1 (2.2%)	0	2 (3.6%)	1 (10.0%)	8 (3.9%)
Vomiting	0	1 (2.3%)	0	1 (7.7%)	3 (6.7%)	0	2 (3.6%)	0	7 (3.4%)
Sepsis	1 (2.8%)	3 (7.0%)	0	0	0	0	1 (1.8%)	0	5 (2.4%)
Basal cell carcinoma	0	0	0	0	0	0	4 (7.3%)	0	4 (1.9%)
Chills	0	0	0	0	0	0	4 (7.3%)	0	4 (1.9%)
Seizure	0	0	0	0	4 (8.9%)	0	0	0	4 (1.9%)
Squamous cell carcinoma	0	0	0	0	0	0	4 (7.3%)	0	4 (1.9%)
Squamous cell carcinoma of the skin	0	0	0	0	0	0	4 (7.3%)	0	4 (1.9%)
Nausea	0	0	0	0	3 (6.7%)	0	0	1 (10.0%)	4 (1.9%)
Acute kidney injury	2 (5.6%)	1 (2.3%)	0	0	0	0	0	0	3 (1.5%)
Cholangitis	0	3 (7.0%)	0	0	0	0	0	0	3 (1.5%)
Dehydration	1 (2.8%)	1 (2.3%)	0	0	0	0	1 (1.8%)	0	3 (1.5%)
Fatigue	1 (2.8%)	0	0	0	0	0	1 (1.8%)	1 (10.0%)	3 (1.5%)
Decreased neutrophil count	2 (5.6%)	0	0	0	0	0	1 (1.8%)	0	3 (1.5%)
Pleural effusion	3 (8.3%)	0	0	0	0	0	0	0	3 (1.5%)
Pulmonary embolism	1 (2.8%)	0	0	0	1 (2.2%)	0	1 (1.8%)	0	3 (1.5%)
Vertigo	0	0	0	0	1 (2.2%)	0	2 (3.6%)	0	3 (1.5%)
Anemia	1 (2.8%)	0	0	1 (7.7%)	0	0	0	0	2 (1.0%)
Atrial fibrillation	0	1 (2.3%)	0	0	0	0	1 (1.8%)	0	2 (1.0%)
Bladder neoplasm	0	0	0	0	0	0	2 (3.6%)	0	2 (1.0%)
Cellulitis	0	0	0	0	0	0	2 (3.6%)	0	2 (1.0%)
Diarrhea	0	1 (2.3%)	0	0	0	0	0	1 (10.0%)	2 (1.0%)
Dizziness	1 (2.8%)	0	0	0	1 (2.2%)	0	0	0	2 (1.0%)
Decreased ejection fraction	1 (2.8%)	0	0	0	1 (2.2%)	0	0	0	2 (1.0%)
Febrile neutropenia	0	1 (2.3%)	0	0	0	0	1 (1.8%)	0	2 (1.0%)
Femoral neck fracture	1 (2.8%)	1 (2.3%)	0	0	0	0	0	0	2 (1.0%)
Hematochezia	2 (5.6%)	0	0	0	0	0	0	0	2 (1.0%)
Hematuria	1 (2.8%)	0	0	0	0	0	1 (1.8%)	0	2 (1.0%)
Headache	0	0	0	0	2 (4.4%)	0	0	0	2 (1.0%)
Hypotension	1 (2.8%)	0	0	0	0	0	1 (1.8%)	0	2 (1.0%)
Infection	0	0	0	0	0	0	2 (3.6%)	0	2 (1.0%)
Leukopenia	2 (5.6%)	0	0	0	0	0	0	0	2 (1.0%)
Neutropenia	1 (2.8%)	0	0	0	1 (2.2%)	0	0	0	2 (1.0%)
Wound infection	1 (2.8%)	0	0	0	0	0	1 (1.8%)	0	2 (1.0%)

Values are number of patients, *n* (%).

Preferred terms by MedDRA version 23.0 and CTCAE version 4.0.

All treated patients (primary and expansion cohorts).

MedDRA, Medical Dictionary for Regulatory Activities.

Fatal SAEs were reported in nine (4.4%) patients. Fatal SAEs were seen in three (8.3%) patients in the ATC (sepsis, pneumonia, diverticulitis, pleural effusion and pulmonary embolism) cohort and three (5.5%) patients in the HCL (sepsis, pneumonia, adenocarcinoma of the pancreas and hemorrhagic stroke) cohort. In the BTC cohort, two (4.7%) patients died due to sepsis, and one patient in the HGG cohort died due to general physical health deterioration. No fatal SAEs were reported in the LGG and MM cohorts. None of these fatal SAEs was related to the study drug.

### Deaths

Across all cohorts, 111 (53.9%) patients died; of these, 20 (9.7%) died within 30 days from the last dose of the study treatment. The most common primary cause of death was disease progression in 90 (43.7%) patients. One (0.5%) patient died due to other cancer, and the cause of death was missing in seven (3.4%) patients. A summary of deaths across tumor cohorts is shown in Extended Data Table 6. None of the deaths was reported to be related to the study drug.

### Study drug discontinuation and interruptions

Across all cohorts, 28 (13.6%) patients had AEs that led to discontinuation of the study treatment (dabrafenib or trametinib). The most common AEs leading to study drug discontinuation were nausea (1.5%) and dyspnea, decreased ejection fraction, headache, pleural effusion, pneumonia, pyrexia and sepsis (1.0% each) (Supplementary Table 3).

A total of 91 patients (44.2%) and 116 (56.3%) patients had AEs requiring dose reduction and any dose interruption, respectively. Pyrexia (*n* = 38, 18.4%) was the most frequent AE requiring dose reduction, followed by chills (*n* = 17, 8.3%) and fatigue (*n* = 10, 4.9%). The most frequently reported (≥5% of patients) AEs leading to dose interruptions were pyrexia (*n* = 48, 23.3%), chills (*n* = 20, 9.7%) and nausea (*n* = 12, 5.8%).

## Discussion

To our knowledge, ROAR is the first prospective study of a combination treatment approach with *BRAF* and *MEK* inhibitors in patients with advanced rare cancers harboring the *BRAFV600E* mutation. The ROAR study demonstrated pan-cancer activity of the *BRAF* and *MEK* inhibitor combination in 21 histologies. We adopted a basket design for this study where tumors expressing the same driver mutation (that is, *BRAFV600E*) were grouped and treated as a single disease entity. A similar multi-cohort basket design was used in the VE-BASKET study that evaluated the efficacy of vemurafenib monotherapy in patients (*n* = 172) with any *BRAFV600* mutation-positive cancers (26 unique cancer types) other than melanoma, papillary thyroid cancer and HCL^19^. After the initiation of the ROAR study, the NCI initiated the MATCH study in which dabrafenib plus trametinib was tested in various *BRAFV600E*-mutated solid and hematological malignancies.

The ROAR study included patients with advanced disease, and all patients had received pre-treatment with standard-of-care therapies. This reduced the risk of observing an inflated ORR. In this study, we deployed an adaptive design for ORR with a Bayesian hierarchical model to increase the power to identify clinically meaningful outcomes. This helped to identify cohorts that could be further expanded and allowed for multiple interim data evaluations to determine if a histologic cohort should discontinue enrollment early due to success or futility. The ATC, BTC, HGG and HCL cohorts were stopped early for efficacy, and an expansion cohort was opened for each of these cohorts. None of the cohorts was stopped early due to futility. Additional strengths of the study design included the analysis of ORR with standard frequentist estimates for each of the cohorts and an independent radiology review for solid tumor cohorts.

In line with available evidence in earlier studies^11,12,16,20^, treatment with dabrafenib plus trametinib in this study showed clinically meaningful outcomes in a diverse set of rare cancers with *BRAFV600E* mutations and had a manageable safety profile. There was consistency in ORR by an independent radiology review and in the investigator-assessed responses in the solid tumor cohorts.

The ROAR study supports a tumor-agnostic approach to *BRAFV600E* inhibition due to the inclusion of diverse cancer cohorts including hematological malignancies. The results of this study formed the basis of the FDA approval of dabrafenib plus trametinib for unresectable or metastatic solid tumors with *BRAFV600E* mutations. This emphasizes the utility of targeting tumor genomics in clinical practice for the treatment of rare cancers.

The results of the ROAR study further corroborate those of subprotocol H (EAY131-H) of the NCI-MATCH platform trial, which included 35 patients (primary efficacy analysis in 29 patients) with *BRAFV600E*-positive cancers of the gastrointestinal tract (*n* = 11) and central nervous system (CNS, *n* = 5), myeloma (*n* = 1), gynecological cancers (*n* = 6), adenocarcinoma of the lung (*n* = 5) and ameloblastoma of the mandible (*n* = 1). The NCI-MATCH study reported an ORR of 38% (90% CI: 22.9, 54.9; *P* < 0.0001) with dabrafenib plus trametinib in patients with *BRAF V600E/D/R/K* mutation-positive solid tumors, lymphomas or MM whose disease had progressed on at least one standard therapy^20^. Similar results for ORR were observed in the tumor cohorts in our study. Cancers such as histiocytic sarcoma of the brain and ameloblastoma, which are very rare and have no defined standard of care, have shown benefit with dabrafenib plus trametinib in the NCI-MATCH study.

The median DoR in included tumor cohorts in our study ranged from 31.2 months to NR and was higher when compared to the DoR reported in the NCI-MATCH study, which was 25.1 months (90% CI: 12.8, NR)^20^. Similarly, the NCI-MATCH study reported a higher median PFS of 11.4 months (90% CI: 7.2, 16.3) and OS of 28.6 months. In the ROAR study, the median PFS ranged from 5.5 months to 9.5 months and the median OS ranged from 13.5 months to 33.9 months in the included tumor cohorts.

The findings from the ROAR study are also complementary to available case reports supporting the efficacy of dabrafenib plus trametinib in various *BRAFV600E* mutation-positive tumors, including breast cancer^21^, pancreatic cancer^22,23^, salivary duct cancer^24^, Bellini duct cancer^25^, pituitary tumor^26^, ameloblastoma^27^, histiocytic sarcoma^28^ and gynecological malignancies^29,30^. Some of the cancers reported here are ultra-rare cancers and have never been studied before with *BRAFV600* inhibitors in clinical trials.

In a phase 2 trial (ClinicalTrials.gov identifier: NCT02684058), dabrafenib plus trametinib improved the ORR and prolonged PFS compared to standard chemotherapy in 110 pediatric patients (aged 1–17 years) with *BRAFV600*-mutant LGG^31^. There are also case reports of the pediatric population benefiting with dabrafenib plus trametinib in the treatment of *BRAF* mutation-positive glioblastoma and gliomas^32–34^, pancreatic acinar cell carcinoma^35^ and Wilms tumor^36^.

**Fig. 2:**
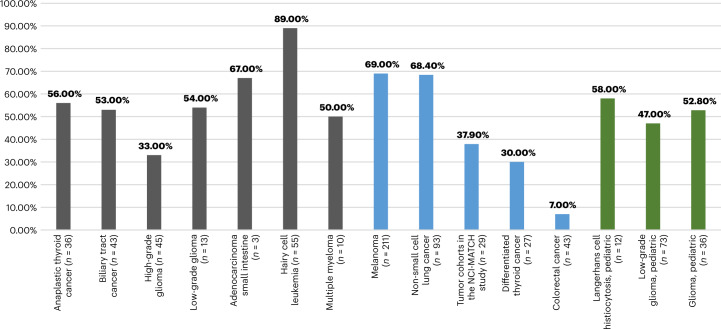
ORR with dabrafenib plus trametinib in cancers with *BRAFV600E* mutations. This figure presents data for the ROAR study (gray) and other studies in adults (blue) and the pediatric population (green). Data are presented for the COMBI-d study (NCT01584648) for melanoma, BRF113928 (NCT01336634) for NSCLC, NCT01723202 for differentiated thyroid cancer and ROAR (NCT02034110) and NCI-MATCH (NCT02465060) for the other tumor types. Pediatric data for gliomas and Langerhans cell histiocytosis are from the study NCT02124772, and those for LGGs are from the study NCT02684058. Data for colorectal cancer are from the study NCT01072175 (data on file, Novartis). The NCT01584648 study included previously untreated patients; NCT01723202 included patients who were refractory to radioactive iodine; NCT01336634 included both previously treated and treatment-naive patients; and other studies included patients who previously received standard treatment. Patient numbers are the patients who received treatment with dabrafenib plus trametinib. The ORR for GIST (*n* = 1) in the ROAR study was 0. Patients with melanoma, thyroid cancer, colorectal cancer and NSCLC were excluded in the NCI-MATCH study. This study included patients with BRAFV600E-positive tumors of the gastrointestinal tract (*n* = 11), lung (*n* = 5), CNS (*n* = 5), myeloma (*n* = 1), ameloblastoma of the mandible (*n* = 1) and gynecologic malignancies (*n* = 6). In the NCI-MATCH study, no CRs were observed; durable PRs were seen across a variety of tumor types, including papillary adenocarcinoma of the lung (*n* = 5), low-grade serous ovarian carcinoma (*n* = 5), mucinous-papillary serous adenocarcinoma of the peritoneum (*n* = 1), histiocytic sarcoma of the brain (*n* = 1), pleomorphic xanthoastrocytoma of the parietal lobe (*n* = 1) and cholangiocarcinoma (*n* = 4).

Figure 2 presents the ORR from various studies that provide evidence of the tumor-agnostic efficacy of dabrafenib plus trametinib in *BRAFV600*-positive rare cancers^20,31,37–42^.

In general, *BRAFV600* inhibitors lack meaningful efficacy in colorectal cancer, and this appears to be a unique case^43^. The suboptimal MAPK signaling with dabrafenib plus trametinib in colorectal cancer may be explained by an epigenetic mechanism including the utilization of epidermal growth factor receptor signaling to maintain *BRAF-MEK-ERK* signaling in the face of *BRAF* inhibition. The same mechanism has not been described in another cancer to date. In *BRAF*-mutated papillary thyroid cancer and differentiated thyroid cancer, a similar mechanism did not impact the clinical efficacy^41,44^.

There is an unmet need in the management of rare cancers with *BRAFV600E* mutations that currently have limited or no treatment options, and dabrafenib plus trametinib can help to address this need. Results from the ROAR study have supported the FDA approval for dabrafenib (Tafinlar) plus trametinib (Mekinist) in rare advanced solid cancers with *BRAFV600E* mutations. This approval opens up access to patients with rare cancers with *BRAFV600E* mutations who otherwise may not have access to this valuable treatment. This is key to advancing clinical research in rare cancers and enabling evidence-based clinical practice in the management of these cancers^45^. Similarly to other studies, such as ProTarget (NCT04341181), IMPRESS (NCT04817956) and MEGALiT (NCT04185831), the ROAR study is an endeavor to define actionable genomic targets to help advance management and provide equal treatment opportunities in the management of cancers, including rare cancers^46,47^.

Dabrafenib 150 mg twice daily plus trametinib 2 mg once daily was well tolerated in this study, with a clinically acceptable incidence of AEs, dose interruptions and treatment discontinuations. Across all cohorts, the observed safety profile of this combination was consistent with the known safety profile in approved indications of *BRAFV600* mutation-positive unresectable or metastatic melanoma, NSCLC and ATC^11–14^. No new safety signals were identified.

The most frequently reported AEs across all cohorts were pyrexia (54.9%), fatigue (42.2%) and nausea (41.7%). The other AEs noted during the study were largely consistent with what is expected in pre-treated populations with cancer and that could be attributed to the cancer type in a cohort. For example, sepsis was seen in three patients with BTC, and a higher incidence of headache was reported in glioma cohorts. Similarly, a higher incidence of hematological abnormalities was seen in hematological cohorts, even though the incidence of such abnormalities was low.

This study has some limitations. It has a non-randomized, single-arm design (per histology). Given the rarity of the tumor types included in the study, it is challenging to design a randomized study for the included cohorts. Although the study collected data for quality of life, these data were not available for most patients. When available, these data were highly variable and did not provide interpretable results. This study did not assess the heterogeneity of the *BRAFV600E* mutations. Further studies may be planned to correlate the efficacy of therapy with the degree of *BRAFV600E* mutation heterogeneity. Due to inclusion of diverse tumor cohorts, there was heterogeneity in patient characteristics. Therefore, the study did not assess the impact of baseline characteristics, previous treatment or any other factors on response to treatment. However, the correlation of patient characteristics with response to treatment has been reported in some studies^18,48,49^.

In conclusion, combination treatment with dabrafenib plus trametinib showed meaningful clinical activity in patients with *BRAFV600E*-mutated rare cancers and was approved in the United States as a therapeutic option in patients with advanced, rare solid tumors with *BRAFV600E* mutations. In clinical practice, testing for *BRAFV600E* mutations can help to improve outcomes by providing a targeted treatment option for patients with rare cancers and limited treatment options. Furthermore, genetic testing and tumor profiling should be introduced early in the management plan to promptly identify those patients who may be eligible for *BRAFV600E*-targeted treatment. In addition to the previously treated cancers in this study, future studies could evaluate the combination treatment with dabrafenib plus trametinib in treatment-naive *BRAFV600E* mutation-positive cancers.

## Methods

### Study design

This multicenter, single-arm, open-label, phase 2 basket study evaluated the efficacy and safety of dabrafenib plus trametinib in nine cohorts of patients with *BRAFV600E* mutation-positive rare cancers. The study was conducted at 27 community and academic cancer centers in 13 countries (Austria, Belgium, Canada, France, Germany, Italy, Japan, The Netherlands, Norway, South Korea, Spain, Sweden and the United States). The study centers include: AKH Wien-Vienna (Vienna, Austria), Universitaetsklinik Innsbruck-Innsbruck (Vienna, Austria), LKH Salzburg-Salzburg (Vienna, Austria), Krankenhaus der Elisabethinen Linz-Linz (Vienna, Austria), UZ Brussel-Brussel (Brussels, Belgium), Princess Margaret Hospital Toronto (Ontario, Canada), Rigshospitalet Onkologisk Afdeling, Fase 1 Enhed (Hillerød, Denmark), Institut Cancerologie de l’Ouest - Rene Gauducheau-Saint-Herblain cedex (Saint-Herblain, France), Institut de Cancerologie Gustave Roussy (Lyon, France), Institut Claudius Regaud - Toulouse cedex 9 (France), Centre Leon Berard (Lyon Cedex 08, France), Institut Bergonie (Lyon Cedex 08, France), CHU de Nantes - Hotel Dieu, Service Hematologie Clinique (Lyon Cedex 08, France), Universitaetsklinikum Freiburg-Inner-Freiburg (Freiburg im Breisgau, Germany), Universitaetsklinikum Mannheim GmbH-Haematologie-Mannheim (Tubingen, Germany), Universitaetsklinikum Eppendorf-II. Med. Klinik- Hamburg (Tubingen, Germany), Charite-Campus Virchow Klinikum-Onkologie (Berlin, Germany), Universitaetsklinikum Heidelberg-Medizinische Klinik V (Tubingen, Germany), Istituto Europeo di Oncologia (IRCCS) di Milano (Milano, Italy), Istituto Nazionale Tumori- Milano-Italy (Milano, Italy), Ospedale San Raffaele IRCCS-Milano-Italy (Milano, Italy), Seoul National University Hospital (Seoul, Korea), Gangnam Severance Hospital-Seoul-Korea (Seoul, Korea), The Netherlands Cancer Institute-Amsterdam (Amsterdam, The Netherlands), UMC Utrecht (Amsterdam, The Netherlands), Erasmus MC Rotterdam (Amsterdam, The Netherlands), VU Medisch Centrum-Amsterdam (Amsterdam, The Netherlands), REK Sør-Øst (Oslo, Norway), Dana-Farber Cancer Institute (Massachusetts, United States), Sarah Cannon Cancer Center (Washington, United States), National Cancer Institute (Maryland, United States), The University of Texas MD Anderson Cancer Center (Texas, United States), UCLA-Santa Monica (California, United States), University of Arkansas for Medical Sciences-Little Rock (Arkansas, United States), New York University Medical Center (New York, United States), Karolinska Universitetssjukhuset Solna (Solna, Sweden), Hospital Universitario Valle de Hebron (Barcelona, Spain), University Hospital 12 de Octubre (Madrid, Spain), National Cancer Center Hospital-Tokyo (Tokyo, Japan) and National Cancer Center Hospital East-Chiba (Chiba, Japan).

Patients received oral dabrafenib (150 mg twice daily) and oral trametinib (2 mg once daily) on a continuous dosing schedule until unacceptable toxicity, disease progression or death. Dose adjustments and interruptions were permitted for patients unable to tolerate the protocol-specified dose until tolerability improved. Data cutoff date for this study was 10 December 2021.

For patients who discontinued or withdrew from study treatment, follow-up visits were conducted within 28 days after the last dose, every month for the first 6 months for dermatologic assessments and every 3 months thereafter for survival data.

The study was conducted in compliance with ICH Good Clinical Practice guidelines and ethical principles described in the Declaration of Helsinki. The study protocol and all amendments were reviewed by the independent ethics committee or institutional review board for each participating study center. All patients signed a written informed consent before study-specific procedures. The study is registered in ClinicalTrials.gov as NCT02034110 and EudraCT as 2013-001705-87. The study protocol can be located in the Supplementary Information for this publication.

### Study population

Eligible patients were aged ≥18 years with histologically confirmed *BRAFV600E* mutation-positive advanced tumor with no standard treatment options and ECOG performance status score ≤2. Patients with ATC, BTC, GIST and ASI had at least one measurable lesion disease according to the Response Evaluation Criteria In Solid Tumors (RECIST) version 1.1 (ref. ^50^) outside of a prior radiation field or within the field with evidence of progression. Patients with LGG were required to have measurable non-enhancing disease (except pilocytic astrocytoma) at baseline using the Response Assessment in Neuro-Oncology (RANO) LGG criteria^51^. Tumor tissue samples were collected at baseline for retrospective histologic confirmation at a central reference laboratory. *BRAFV600E* mutations were identified using local assays at individual sites or using the THxID-BRAF kit (bioMérieux) at the designated central reference laboratory (Hematogenix Laboratory Services). All locally obtained mutation results were retrospectively tested by the central reference laboratory for *BRAFV600E* mutation status.

Patients were excluded if they had received prior treatment with *BRAF* and/or *MEK* inhibitor(s) or had a history of malignant disease (including tumors with confirmed activating RAS mutation). Patients with any serious and/or unstable preexisting medical or psychiatric disorder and those with CNS involvement (except LGG and HGG cohorts), relevant cardiac disease or gastrointestinal abnormalities, interstitial lung disease or pneumonitis or history of retinal vein occlusion were excluded. Pregnant or lactating women and patients with any unresolved grade ≥2 (per Common Terminology Criteria for Adverse Events (CTCAE) version 4.0) toxicity from previous anti-cancer therapy at the time of enrollment (except alopecia or grade 2 anemia) were also excluded. Eligibility criteria by tumor histology are detailed in Supplementary Table 4.

### Study objectives

#### Primary objective

The primary objective of the study was to determine the ORR of dabrafenib plus trametinib therapy in patients with rare *BRAFV600E*-mutated solid tumors or hematologic malignancies.

#### Secondary objectives

The secondary objectives of the study were to determine the DoR, PFS, OS and safety of dabrafenib plus trametinib in patients with rare *BRAFV600E*-mutated solid tumors or hematologic malignancies.

### Study endpoints

#### Primary endpoint

The primary endpoint was tumor response as assessed by the investigator and defined by RECIST version 1.1 for solid tumor histologies including ATC, BTC, GIST, ASI and NSGCT/NGGCT^50^, the RANO criteria for LGG and modified RANO for HGG^51,52^. Response was also assessed centrally by independent radiology review for the ATC, BTC, ASI, LGG and HGG cohorts. Tumor responses in MM were defined by the International Myeloma Working Group (IMWG) Uniform Response Criteria^53^, and those for HCL were adapted from the National Comprehensive Cancer Network (NCCN) Clinical Practice Guidelines in Oncology for HCL^54^, consensus resolution criteria^55^ and definitions from previous studies^18^.

#### Secondary endpoints

The secondary endpoints included DoR (defined as the time from the first documented evidence of CR or PR until documented disease progression or death from any cause); PFS (defined as the time from the first dose to disease progression or death from any cause, whichever occurred earlier); OS (defined as the time from the first dose of the study drug until death from any cause); and safety as assessed by the investigator. Safety assessments included change from baseline in physical examination findings, vital signs, AEs, laboratory values and cardiac assessments.

### Study assessments

ECOG performance status was assessed at screening and monitored monthly during treatment. The use of concomitant medications was assessed after 2 weeks of enrollment and then monthly during treatment.

#### Disease assessment

For solid tumors (ATC, BTC, GIST, LGG, HGG and ASI), assessments were done at baseline (≤35 days before enrollment) and post-baseline every 8 weeks during the first 48 weeks and then every 12 weeks thereafter, until disease progression. Tumor assessments were conducted as per RECIST version 1.1 (ref. ^1^) by using computed tomography scans or magnetic resonance imaging. In the HCL cohort, patients underwent disease assessments by local investigators every 4 weeks for the first 48 weeks and every 8 weeks thereafter, until disease progression. Central disease assessments were not done. Disease assessments included complete blood count, flow cytometry and routine hematoxylin and eosin staining of peripheral blood/bone marrow aspirate and bone marrow immunohistochemistry. Bone marrow biopsies were repeated after 6 months, 1 year, 2 years and 3 years and then every 2 years. In the MM cohort, baseline and post-baseline disease assessments were conducted by skeletal surveys, extramedullary disease assessment of bone marrow aspirate and biopsy samples by immunohistochemistry, flow cytometry, fluorescence in situ hybridization, cytogenetics and laboratory tests (urine protein electrophoresis, serum protein electrophoresis, C-reactive protein, β-2 microglobulin, serum free light chain assay and levels of immunoglobulin G, A and M). To monitor for secondary malignancies, physical examination and chest/abdominal imaging were performed at screening (≤14 days before the first dose) and every 3 months or as clinically indicated thereafter.

#### Laboratory assessments

Laboratory assessments at screening included clinical chemistry and hematological assessments, measurement of glycated hemoglobin, evaluation of coagulation factors and urinalysis. Chemistry and hematological assessments and urinalysis were done monthly during treatment, and glycated hemoglobin was assessed every 3 months.

#### Safety assessments

Safety assessments at screening included physical examination, full dermatologic examination, ophthalmic examination, assessment of vital signs (blood pressure, temperature, pulse and respiratory rate) and electrocardiogram and echocardiogram. During treatment, a brief dermatologic examination was performed monthly, and an ophthalmic examination was performed only after the first month unless clinically indicated thereafter. Monthly assessment of vital signs and electrocardiogram were also required during treatment, and an echocardiogram was performed after the first month and every 3 months thereafter. Besides the parameters described in the protocol, additional safety tests were to be performed by the investigators during the course of the study based on newly available data to ensure appropriate safety monitoring. Appropriate local regulatory and ethical approvals applied before any additional testing was performed.

AE monitoring was done continuously from the time the first dose of study treatment was administered until 30 days after discontinuation of study treatment. AEs were graded according to CTCAE version 4.0 (ref. ^56^).

### Statistical analysis

This study was designed to include nine cohorts of different solid and hematological malignancies.

#### Bayesian hierarchical model

To address the small sample size per histologic cohort, an adaptive design using a Bayesian hierarchical model was implemented to increase the power to detect clinically meaningful ORRs by borrowing information across the included histology cohorts while controlling the type I error rate. Across cohorts, the historical response rates used were 10%, except for ATC and MM (15%) and NSGCT/NGGCT (25%), and the threshold for clinically meaningful response in this study was 50%.

#### Primary analysis and expansion cohorts

The primary analysis cohort enrolled a maximum of 25 patients per tumor type. Multiple interim analyses (every 12 weeks) were performed to monitor the safety and efficacy and to determine whether a cohort should discontinue enrollment early because of success or futility. Response data for a minimum of five patients in a given cohort were required before discontinuation due to futility (<30% probability of exceeding the historical response rate), and a minimum of 10 patients were required before discontinuation for efficacy (>95% probability of exceeding the historical response rate). If a cohort closed early for efficacy, a histology subtype-specific expansion cohort, designed to provide supportive efficacy data, could be opened to accommodate additional patient enrollment. Expansion cohorts did not contribute to the Bayesian modeling, and there was no specific sample size calculation for these cohorts. Investigators were allowed to enroll patients into the expansion cohort for the duration of trial enrollment. At the final analysis, enrollment of a minimum of two patients in a given cohort was required to meet statistical significance.

#### Endpoint analyses

The primary endpoint of ORR was also analyzed using the frequentist methodology (point estimates and 95% CIs) including patients from the primary and expansion cohorts. Simulations were conducted to evaluate the performance of this study design under various ORR distributions across cohorts, accounting for power, type I error, ORR estimation and probability of halting at an interim analysis. With similar treatment effects across all cohorts, this design would maintain 84–98% power and a type I error rate ≤0.04; power ranges from 55% to 90% and type I error from 0.03 to 0.14 if treatment effect varies across histologic subtypes.

The efficacy evaluable population was defined as all enrolled patients, regardless of whether or not treatment was administered, and encompassed all patients enrolled in both the primary and expansion cohorts for a given histological cancer subtype. Evaluable patients included those who had progressive disease, had initiated new anti-cancer treatment, had withdrawn consent, had died, had stable disease for at least 6 weeks after the first dose day or had at least two post-baseline disease assessments (other than not evaluable).

PFS, DoR and OS were analyzed for the combined primary and expansion cohorts using the Kaplan–Meier methodology, with 95% CIs for median and milestone estimates constructed using the Brookmeyer–Crowley method with a complementary log–log transformation of the survivor function; 95% CIs were used for uncertainty estimates and were investigator assessed. Patients with an unknown or missing response were treated as non-responders and were included in the denominator when calculating the percentage. Time-to-event secondary endpoints were right censored if the event was not observed during the study follow-up.

The independent radiology review was done as a supportive analysis in the ATC, BTC, ASI, LGG and HGG cohorts. Primary and secondary efficacy endpoints based on independent reviewer assessment were summarized in the same way as the best response based on investigator assessment. An assessment of the concordance between the investigator-assessed response and independent reviewer-assessed response was performed. In addition, waterfall plots were generated for best confirmed response based on investigator and independent assessment.

The safety population included all patients who received at least one dose of dabrafenib or trametinib. Safety data were summarized descriptively by histology and across all cohorts. Incidence of deaths and primary cause of death were summarized. An independent data monitoring committee reviewed safety and activity results from interim analyses at regular intervals and provided recommendations to the sponsor.

#### Statistical tools

SAS (version 9.3, SAS Institute) was used for all statistical analysis except for the hierarchical Bayesian model, which used C++ and R version 2.15.2 to evaluate the performance of the design under various assumptions for the distribution of true ORRs across histological cohorts and accounting for anticipated small sample sizes.

### Reporting summary

Further information on research design is available in the Nature Portfolio Reporting Summary linked to this article.

## Online content

Any methods, additional references, Nature Portfolio reporting summaries, source data, extended data, supplementary information, acknowledgements, peer review information; details of author contributions and competing interests; and statements of data and code availability are available at 10.1038/s41591-023-02321-8.

## Supplementary information

Supplementary Information Supplementary Tables 1–3 and Study Protocol.

Reporting Summary

## Figures and Tables

**Extended Data Table 1:**
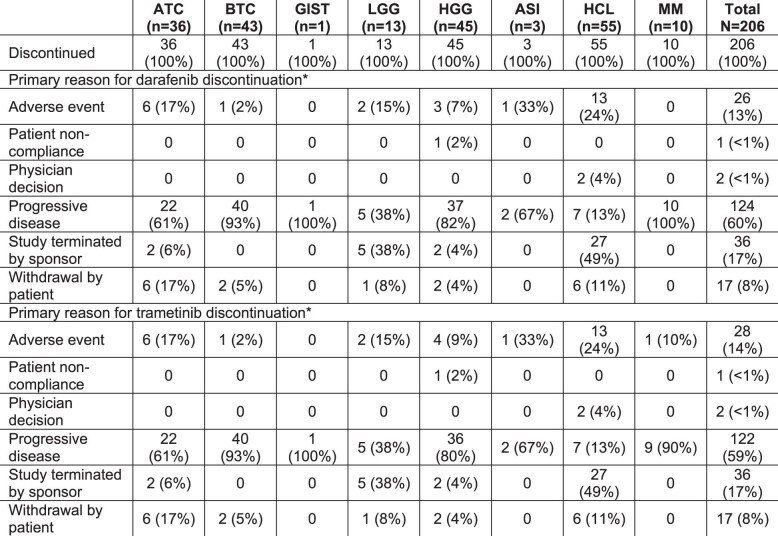
Study treatment discontinuation

Values are number of patients, *n* (%). Population is all treated patients. Primary and expansion cohorts.

^a^Patients may have only one primary reason for discontinuation.

G, grade.

**Extended Data Table 2:**
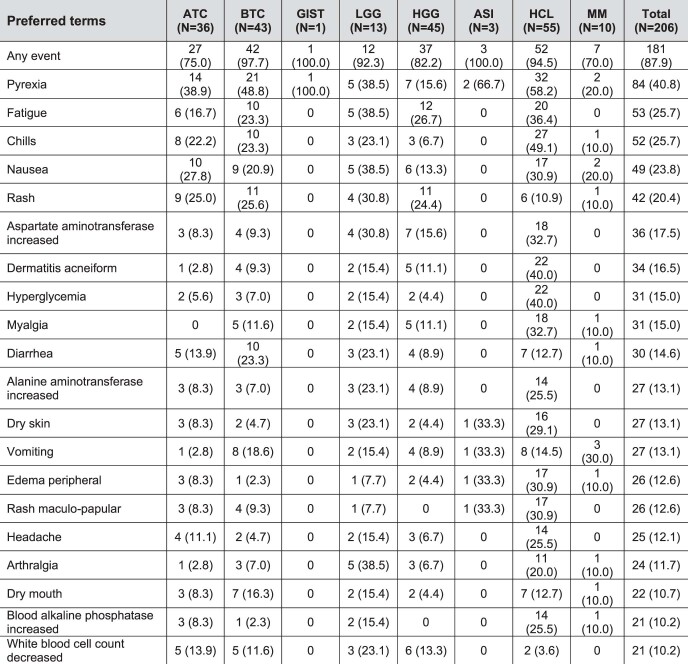
AEs (≥10% in all cohorts) suspected to be study treatment related

Values are number of patients, *n* (%).

Preferred terms by MedDRA version 23.0 and CTCAE version 4.0.

All treated patients (primary and expansion cohorts).

ALP, alkaline phosphatase; ALT, alanine aminotransferase; AST, aspartate aminotransferase; G, grade; MedDRA, Medical Dictionary for Regulatory Activities; WBC, white blood cell.

**Extended Data Table 3:**
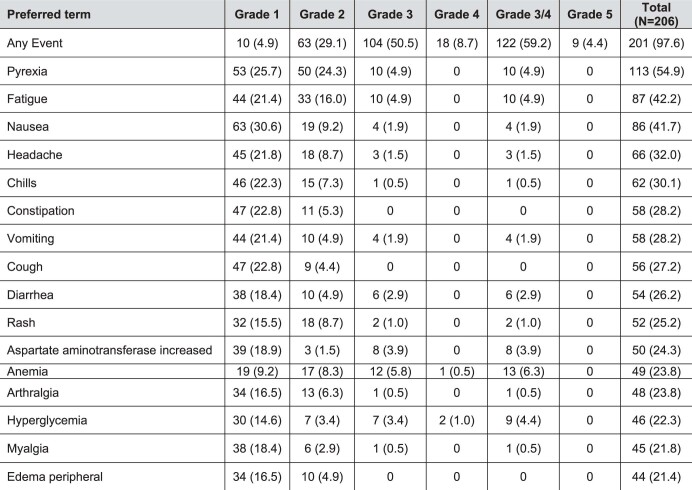
AEs (≥20% in all cohorts) regardless of study drug relationship by maximum toxicity grade

Values are number of patients, *n* (%).

Preferred terms by MedDRA version 23.0 and CTCAE version 4.0.

All treated patients (primary and expansion cohorts).

MedDRA, Medical Dictionary for Regulatory Activities.

**Extended Data Table 4:**
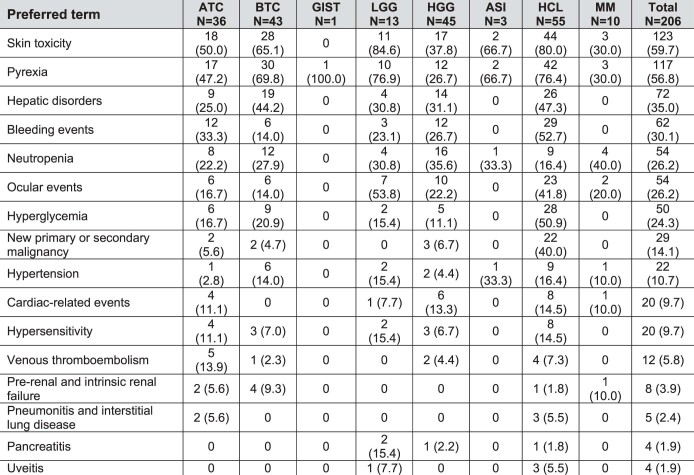
AESIs (all grades) across cohorts

Values are number of patients, *n* (%).

All treated patients (primary and expansion cohorts).

Preferred terms by MedDRA version 23.0 and CTCAE version 4.0.

G, grade; MedDRA, Medical Dictionary for Regulatory Activities.

**Extended Data Table 5:**
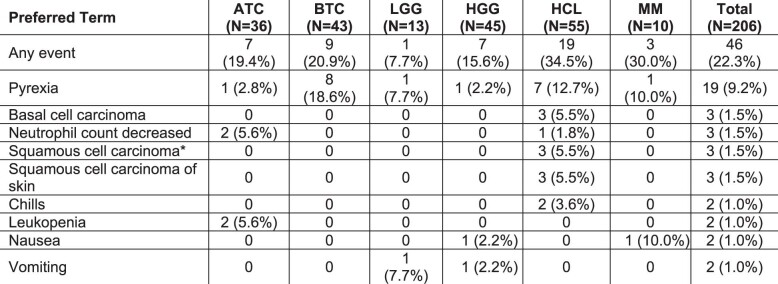
SAEs (≥2 patients in total) suspected to be related to the study treatments

Values are number of patients, *n* (%).

Preferred terms by MedDRA version 23.0 and CTCAE version 4.0.

All treated patients (primary and expansion cohorts).

No suspected SAEs in the GIST and the ASI cohorts; hence, they are not included in the table.

Includes SAEs that are related to either dabrafenib or trametinib.

^a^All three cases were cutaneous.

G, grade; MedDRA, Medical Dictionary for Regulatory Activities.

**Extended Data Table 6:**
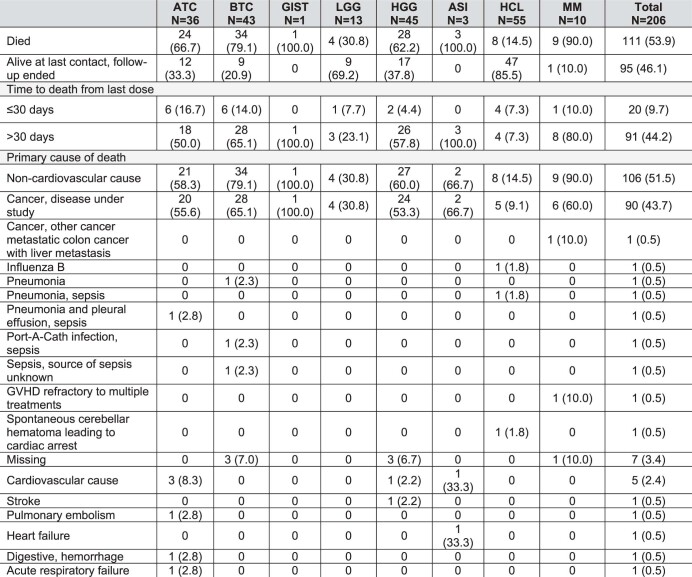
Summary of deaths

All treated patients (primary and expansion cohorts).

G, grade; GVHD, graft versus host disease.

Values are number of patients, *n* (%).

## Data Availability

Novartis is committed to sharing with qualified external researchers access to patient-level data and supporting clinical documents from eligible studies. These requests are reviewed and approved by an independent review panel based on scientific merit. All data provided are anonymized to respect the privacy of patients who have participated in the trial, in line with applicable laws and regulations. This trial data availability is according to the criteria and process described at https://www.clinicalstudydatarequest.com/. The authors declare that all data supporting the findings of this study are available within the article and its Supplementary Information files.
