# Supplementary material for: Dabrafenib plus trametinib in BRAFV600E-mutated rare cancers: the phase 2 ROAR trial
Source: Nat Med. 2023 Apr 14;29(5):1103–12. doi: 10.1038/s41591-023-02321-8 (PMC10202803; doi:10.1038/s41591-023-02321-8)
Supplement: Supplementary file 1 — Supplementary Tables 1–3 and Study Protocol. [file 41591_2023_2321_MOESM1_ESM.pdf]

nature medicine

Article

https://doi.org/10.1038/s41591-023-02321-8

Dabrafenib plus trametinib in *BRAFV600E*-mutated rare cancers: the phase 2 ROAR trial

In the format provided by the authors and unedited

Supplementary Table 1: Additional demographic and baseline characteristics

	ATC (N=36)	BTC (N=43)	GIST (N=1)	LGG (N=13)	HGG (N=45)	ASI (N=3)	HCL (N=55)	MM (N=10)	Total (N=206)
Race									
African American/African Heritage	0	0	0	0	2 (4%)	1 (33%)	0	1 (10%)	4 (2%)
American Indian or Alaska Native	0	0	0	0	1 (2%)	0	0	0	1 (<1%)
Asian - Central/South Asian Heritage	1 (3%)	0	0	0	0	0	0	0	1 (<1%)
Asian - East Asian Heritage	11 (31%)	1 (2%)	0	0	4 (9%)	0	0	1 (10%)	17 (8%)
Asian - Japanese Heritage	2 (6%)	2 (5%)	0	2 (15%)	1 (2%)	0	0	0	7 (3%)
Asian - South East Asian Heritage	2 (6%)	0	0	1 (8%)	1 (2%)	0	0	0	4 (2%)
White - Arabic/North African Heritage	1 (3%)	1 (2%)	0	0	2 (4%)	0	1 (2%)	0	5 (2%)
White - White/Caucasian/European Heritage	17 (47%)	39 (91%)	1 (100%)	10 (77%)	32 (71%)	2 (67%)	48 (87%)	8 (80%)	157 (76%)
Missing	2 (6%)	0	0	0	2 (4%)	0	6 (11%)	0	10 (5%)
TNM staging				-	-		-	-	-
T1	0	1 (2)	0	-	-	0	-	-	-
T2	1 (3)	3 (7)	0	-	-	0	-	-	-
T2B	0	4 (9)	0	-	-	0	-	-	-
T3	3 (8)	2 (5)	1 (100)	-	-	0	-	-	-
T4	0	8 (19)	0	-	-	1 (33%)	-	-	-
T4A	5 (14)	0	0	-	-	0	-	-	-
T4B	10 (28)	0	0	-	-	0	-	-	-
TX	17 (47)	24 (56)	0	-	-	2 (67%)	-	-	-
Missing	0	1 (2)	0	-	-	0	-	-	-
N0	2 (6)	6 (14)	1 (100)	-	-	1 (33%)	-	-	-
N1	0	12 (28)	0	-	-	0	-	-	-
N1A	9 (25)	0	0	-	-	0	-	-	-
N1B	9 (25)	0	0	-	-	0	-	-	-
NX	16 (44)	24 (56)	0	-	-	2 (67%)	-	-	-
Missing	0	1 (2)	0	-	-	0	-	-	-
M0	0	1 (2)	0	-	-	0	-	-	-
MX	1 (3)	0	0	-	-	0	-	-	-
M1	35 (97)	41 (95)	1 (100)	-	-	3 (100%)	-	-	-
Missing	0	1 (2)	0	-	-	0	-	-	-

Population is the efficacy evaluable population.
Values are for primary and expansion cohorts combined for all cancers except disease characteristics for GIST, LGG, and ASI which include the primary analysis cohort.
ASI, adenocarcinoma small intestine; ATC, anaplastic thyroid cancer; BTC, biliary tract cancer; ECOG, Eastern Cooperative Oncology Group; G, grade; GIST, gastrointestinal stromal tumor; HCL, hairy cell leukemia; HGG, high grade glioma (WHO G3/G4); LGG, low (WHO G1/G2) grade glioma; MM, multiple myeloma; TNM, tumor node metastasis; WHO, World Health Organization.

Supplementary Table 2: Summary of Bayesian hierarchical model-based analysis for investigator-assessed best response with confirmation

Cohort	Historical control RR	Number of patients	Number of confirmed responses*	Observed ORR	Estimated response rate and 95% CI**	Probability that the ORR exceeds historical control RR**
ATC	15%	15	11	73%	70% (46.8, 89.3)	1.00
BTC	10%	18	7	39%	41% (21.3, 62.2)	1.00
GIST	10%	1	0	0%	34% (0.1, 81.7)	0.78
LGG	10%	13	9	69%	64% (38.8, 87.1)	1.00
HGG	10%	24	6	25%	30% (13.0, 47.9)	0.99
ASI	10%	3	2	67%	57% (21.2, 91.2)	1.00
HCL	10%	24	22	92%	86% (69.2, 97.3)	1.00
MM	15%	10	5	50%	53% (26.9, 78.0)	1.00

Population is the efficacy evaluable population.
Best response is investigator assessed best response.
Primary analysis cohort.
*Response = CR + PR for ATC, BTC, GIST, HGG and ASI; Response = CR + PR + MR for LGG; Response = CR+PR for HGG; Response = sCR + CR + VGPR + PR for MM; Response = CR with/without MRD + PR for HCL
**Based on Bayesian hierarchical model-based analysis
The differences between the observed response rates and the model-based estimates are attributable to the shrinkage estimation and dynamic borrowing across cohorts. The posterior probability of the ORR exceeding the historical control was high in all cohorts with a caveat that the GIST cohort enrolled only one patient.
ASI, adenocarcinoma of small intestine; ATC, anaplastic thyroid cancer; BTC, biliary tract cancer; CI, credible interval; CR, complete response; G, grade; GIST, gastrointestinal stromal tumor; HCL, hairy cell leukemia; HGG, high (WHO G3/G4) grade glioma; LGG, low (WHO G1/G2) grade glioma; MM, multiple myeloma; MR, minor response; MRD, minimal residual disease; PD, progressive disease; PR, partial response; RR, response rate; sCR, stringent complete response; VGPR, very good partial response; WHO, World Health Organization.

Supplementary Table 3: Adverse events leading to study drug discontinuation

Preferred Term	ATC (N=36)	BTC (N=43)	GIST (N=1)	LGG (N=13)	HGG (N=45)	ASI (N=3)	HCL (N=55)	MM (N=10)	Total (N=206)
Any event	6 (16.7%)	1 (2.3%)	0	2 (15.4%)	4 (8.9%)	1 (33.3%)	13 (23.6%)	1 (10.0%)	28 (13.6)
Nausea	1 (2.8%)	0	0	0	0	0	1 (1.8%)	1 (10.0%)	3 (1.5%)
Dyspnea	2 (5.6%)	0	0	0	0	0	0	0	2 (1.0%)
Ejection fraction decreased	1 (2.8%)	0	0	0	1 (2.2%)	0	0	0	2 (1.0%)
Headache	0	0	0	0	1 (2.2%)	0	1 (1.8%)	0	2 (1.0%)
Pleural effusion	2 (5.6%)	0	0	0	0	0	0	0	2 (1.0%)
Pneumonia	1 (2.8%)	0	0	0	0	0	1 (1.8%)	0	2 (1.0%)
Pyrexia	0	0	0	0	0	1 (33.3%)	1 (1.8%)	0	2 (1.0%)
Sepsis	0	1 (2.3%)	0	0	0	0	1 (1.8%)	0	2 (1.0%)

All treated patients (primary and expansion cohorts).
Values are number of patients, n (%).
Preferred terms by MedDRA version 23.0, CTCAE version 4.0.
ASI, adenocarcinoma of small intestine; ATC, anaplastic thyroid cancer; BTC, biliary tract cancer; CTCAE, Common Terminology Criteria for Adverse Events; G, grade; GIST, gastrointestinal stromal tumor; HCL, hairy cell leukemia; HGG, high (WHO G3/G4) grade glioma; LGG, low (WHO G1/G2) grade glioma; MedDRA, Medical Dictionary for Regulatory Activities; MM, multiple myeloma; WHO, World Health Organization.

Supplementary Table 4: Eligibility criteria by tumor histology

Cohort by tumor type	Inclusion criteria	Exclusion criteria
ATC	• Histologically or cytologically confirmed, unresectable, metastatic ATC including ATC originating from within well-differentiated thyroid cancers or an ATC as part of a thyroid carcinoma of another histologic type.*<br>• Evaluation via indirect or direct laryngoscopy to ensure patency of trachea/airway prior to enrollment if bulky thyroid/neck masses were present and/or airway obstruction was suspected.<br>• Prior external beam radiotherapy and/or surgery to the primary tumor.**	• Presence of thyroid lymphomas, sarcomas, or metastatic disease from other sites of origin to the thyroid.<br>• Potentially curable ATC by surgical excision alone or patients who had not received treatment that might be considered standard of care.
BTC	• Histologically or cytologically confirmed, unresectable, metastatic or locally advanced or recurrent adenocarcinoma of the biliary tract or gallbladder.<br>• Progressed on or demonstrated intolerance (despite standard measures of supportive care and dose reduction) to treatment with a gemcitabine-based chemotherapy regimen.	• Biliary duct obstruction, unless a treatable, clinically relevant obstruction has been relieved by internal endoscopic drainage/stenting, palliative by-pass surgery or percutaneous drainage prior to enrollment.
GIST	• Histologically confirmed diagnosis of c-Kit and PDGFRA wild-type GIST.<br>• Had metastatic or locally advanced, unresectable, or recurrent post-surgical disease.<br>• Had progressed on or demonstrated intolerance (despite standard measures of supportive care and dose reduction) to treatment with a TKI (i.e., imatinib, sunitinib).	-
ASI	• Histologically confirmed, metastatic or locally advanced ASI, adenocarcinoma of the ampulla, or adenocarcinoma of the peri-ampulla.<br>• Progressed on or demonstrated intolerance (despite standard measures of supportive care and dose reduction) to one line of chemotherapy.	-
LGG	• Histologically confirmed recurrent or progressive WHO Grade 1 or 2 glioma<br>• For WHO Grade 1 glioma ONLY: To be eligible, patient:<br>◦ Presented with adequate level of favorable risk/benefit ratio including, but not limited to, the exhibited initial symptoms, AND<br>◦ Evaluated by a panel of neuro-oncologists and the Medical Lead prior to enrollment.<br>• For WHO Grade 2 glioma ONLY: To be considered eligible for study treatment, patient was not eligible for treatment with chemotherapy.<br>• Measurable non-enhancing disease based on two-dimensional MRI with contrast assessments as per RANO response criteria.***<br>• For WHO Grade 1 or 2 glioma patients receiving corticosteroid treatment: Must have received a stable or decreasing dose of corticosteroid treatment for 7 days prior to first dose of study treatment.****	• Prior treatment with enzyme-inducing anticonvulsants within 14 days prior to enrollment.<br>• Radiotherapy treatment within 3 months prior to enrollment.
HGG	• Histologically confirmed recurrent or progressive WHO Grade 3 or 4 glioma.<br>• Prior treatment with radiotherapy and first-line chemotherapy or concurrent chemoradiation therapy.*****<br>• Measurable disease at least 1 cm × 1 cm as per RANO response criteria.<br>• For WHO Grade 3 or 4 glioma patients receiving corticosteroid treatment:<br>◦ Received a stable or decreasing dose of corticosteroid treatment for 7 days prior to first dose of study treatments.****	• Prior treatment with enzyme-inducing anticonvulsants within 14 days prior to enrollment.<br>• Radiotherapy treatment within 3 months prior to enrollment.
NSGCT	• Histologically confirmed NSGCT/NGGCT.<br>• Either of the following:	-

	• Refractory disease defined as disease progression during or relapsed after salvage high-dose chemotherapy, or disease progression during cisplatin-based salvage chemotherapy<br>or<br>• Relapsed disease and ineligible for cisplatin-based salvage chemotherapy or high-dose chemotherapy	
HCL^	• Histologically confirmed diagnosis of HCL according to morphological and immunophenotypic criteria of WHO classification (Campo 2011) of lymphoid neoplasms.<br>• Either of the following:<br>• Refractory disease defined as no response or disease progression in ≤ 1 year following first-line treatment with a purine analog (i.e., pentostatin, cladribine, or fludarabine)<br>OR<br>• Relapsed disease defined as having relapsed following treatment with at least two prior treatments<br>• Leukemic cells in the peripheral blood or BM aspirate or BM biopsy AND one or more of the following:<br>• Bulky/symptomatic splenomegaly<br>• Hemoglobin <10 g/dL<br>• Platelets <100 x10^{9}/L<br>• ANC <1 x10^{9}/L	-
MM^	• Histologically confirmed secretory MM.<br>• At least two prior lines of therapy, such as prior treatment with a proteasome inhibitor and IMiD, and was then refractory or had demonstrated intolerance (despite standard measures of supportive care and dose reduction) to the most recent therapy received<br>• Measurable disease of MM as defined by at least one of the following:<br>• Serum M-protein ≥1 g/dL (≥10 g/L)<br>• Urine M-protein ≥200 mg/24 hr<br>• Serum FLC assay: involved FLC level ≥5 mg/dL (≥50 mg/L) and an abnormal serum FLC ratio (<0.26 or >1.65), or<br>• Biopsy proven plasmacytoma (measured within 28 days of screening)	-

*Squamoid differentiated subtype was not permitted. Diagnosis noted to be "consistent with ATC" with the presence of a thyroid mass was acceptable.
**Patients with small primary tumor that had been totally removed by surgical excision whereby no radiotherapy was indicated (only metastatic lesions are manifested) or patients with metastatic disease who did not require radiation or surgery to the neck mass were eligible for participation in the study.
***Enhancing disease was acceptable for pilocytic astrocytomas.
****Steroids dose was limited to up to 8 mg/day of dexamethasone or equivalent dose of steroid; patients were not required to be on corticosteroids.
*****Patients who had a WHO Grade 3 or 4 glioma for which chemotherapy and/or radiotherapy was not considered standard of care were eligible for the study. The Medical Lead was consulted to discuss and determine if patient was eligible for enrollment.
^ For patients with an opportunistic infection in the HCL and MM cohorts, the infection was to be adequately managed and the patient must be clinically stable. The investigator could discuss this issue with the Medical Lead.

ANC, absolute neutrophil count; ASI, adenocarcinoma of small intestine; ATC, anaplastic thyroid cancer; BM, bone marrow; BTC, biliary tract cancer; FLC, free light chain; G, grade; GIST, gastrointestinal stromal tumor; HCL, hairy cell leukemia; HGG, high (WHO G3/G4) grade glioma; hr, hour; IMiD, immunomodulatory drugs; LGG, low (WHO G1/G2) grade glioma; MM, multiple myeloma; MRI, magnetic resonance imaging; NGGCT, non-germinoma germ cell tumor; NSGCT, non-seminomatous germ cell tumor; PDGFRA, platelet-derived growth factor receptor alpha; RANO, response assessment for neuro-oncology; TKI, tyrosine kinase inhibitor; WHO, World Health Organization.

Reference:
Campo E, Swerdlow SH, Harris NL, Pileri S, Stein H, Jaffe ES. The 2008 WHO classification of lymphoid neoplasms and beyond: evolving concepts and practical applications. *Blood*. 2011 May 12;117(19):5019-32.

NOVARTIS

Clinical Development

GSK1120212+GSK2118436

Protocol BRF117019

A Phase II, Open-label, Study in Subjects with BRAF V600E- Mutated Rare Cancers with Several Histologies to Investigate the Clinical Efficacy and Safety of the Combination Therapy of Dabrafenib and Trametinib

Document type	Amended Protocol Version (Clean)
EUDRACT number	2013-001705-87
Version number	09
Development phase	Phase II
Document status	Final
Release date	12-Feb-2019
Novartis internal reference number	CDRB436X2201

Property of Novartis
Confidential
May not be used, divulged, published, or otherwise disclosed
without the consent of Novartis

Table of contents

Table of contents 2
AMENDMENT 09 (12-Feb-2019) 7
LIST OF ABBREVIATIONS 32
1 INTRODUCTION 36
1.1 Background 36
1.2 Dabrafenib (GSK2118436) 37
1.3 Trametinib (GSK1120212) 37
1.4 Risk Assessment for Dabrafenib + Trametinib Combination Therapy 38
1.4.1 Benefit Assessment 42
1.4.2 Overall Benefit: Risk Conclusion 42
1.5 Histology-Specific Background 42
1.5.1 Cohort 1: Anaplastic Thyroid Cancer (ATC) 42
1.5.2 Cohort 2: Biliary Tract Cancer (BTC) 43
1.5.3 Cohort 3: Gastrointestinal Stromal Tumors (GIST) 44
1.5.4 Cohort 4: WHO Grade 1 and 2 Glioma 45
1.5.5 Cohort 5: WHO Grade 3 and 4 Glioma 46
1.5.6 Cohort 6: Non-Seminomatous/Non-Germinomatous Germ Cell Tumors (NSGCT/NGGCT) 47
1.5.7 Cohort 7: Adenocarcinoma of the Small Intestine (ASI) including Periampullary and Ampullary 48
1.5.8 Cohort 8: Hairy Cell Leukemia (HCL) 49
1.5.9 Cohort 9: Multiple Myeloma (MM) 50
2 RATIONALE 51
2.1 Rationale for the Combination of Dabrafenib and Trametinib 51
2.2 Study Rationale 52
2.3 Dose Rationale 54
2.4 Design Rational 54
3 OBJECTIVES AND ENDPOINTS 55
4 STUDY DESIGN 56
4.1 Discussion of Design 57
4.1.1 BRAF Mutation Test and Companion Diagnostic 58
5 SUBJECT SELECTION 58
5.1 Primary analysis cohort 58
5.2 Expansion cohorts 58
5.3 Number of Subjects 58
5.4 Eligibility 59
5.4.1 Inclusion Criteria 59
5.4.2 Exclusion Criteria 64
6 STUDY TREATMENTS 66

6.1 Treatment Assignment 66
6.2 Blinding 67
6.3 Investigational Products 67
6.3.1 Dabrafenib (GSK2118436) Capsule 67
6.3.2 Trametinib (GSK1120212) Tablet 68
6.4 Handling/Accountability of Investigational Products 68
6.5 Treatment Compliance 69
6.6 Treatment of Overdose 69
6.6.1 Dabrafenib 69
6.6.2 Trametinib 69
6.7 Continuation of Treatment 70
7 STUDY ASSESSMENTS AND PROCEDURES 70
7.1 Time and Event Table 71
7.2 Demographic/Medical History Assessments 80
7.3 Critical Baseline Assessments 80
7.3.1 Baseline Confirmation of BRAF-Mutation Status 80
7.3.2 Baseline Documentation of Target and Non-Target Lesions for Solid Tumor Histologies 81
7.4 Safety 81
7.4.1 Physical Examinations 81
7.4.2 Dermatological Examinations 81
7.4.3 Ophthalmic Examinations 82
7.4.4 Vital Signs 82
7.4.5 Performance Status 83
7.4.6 Electrocardiogram 83
7.4.7 Echocardiogram 83
7.4.8 Clinical Laboratory Assessments 83
7.4.9 Monitoring for Non-Cutaneous Secondary/Recurrent Malignancy 85
7.4.10 Pregnancy Testing and Reporting 86
7.5 Disease Assessment 87
7.5.1 Disease Assessment for Solid Tumor Histologies 87
7.5.2 Disease Assessment for Histologies of Hematologic Malignancies 89
8 DOSE MODIFICATIONS AND STOPPING CRITERIA 91
8.1 Dose Modification 91
8.1.1 Cardiovascular Events 93
8.1.2 Pancreatitis 95
8.1.3 Hepatobiliary Events 96
8.2 Dose Modification and Management Guidelines for Events of Special

Interest......100
8.2.1 Dermatological Events......100
8.2.2 Malignancies...... 102
8.2.3 Pyrexia......103
8.2.4 Pneumonitis......105
8.2.5 Diarrhea......106
8.2.6 Renal Insufficiency......107
8.2.7 Visual Changes......108
9 LIFESTYLE AND/OR DIETARY RESTRICTIONS......110
9.1 Contraception Requirements......110
9.1.1 Female Subjects......110
9.1.2 Male Subjects......111
9.2 Lactation......111
9.3 Caffeine and Alcohol......111
9.4 Activity......111
10 CONCOMITANT MEDICATIONS AND NON-DRUG THERAPIES......111
10.1 Permitted Medications and Non-Drug Therapies......111
10.2 Prohibited Medications and Non-Drug Therapies......112
10.3 Medications to be usedwith Caution......113
11 ADVERSE EVENTS AND SERIOUS ADVERSE EVENTS......115
11.1 Definition of Adverse Event......115
11.2 Definition of Serious Adverse Event......116
11.3 Laboratory and Other Safety Assessment Abnormalities Reported as AEs and SAEs......117
11.4 Cardiovascular Events......117
11.5 Death Events......118
11.6 Disease-Related Events and/or Disease-Related Outcomes Not Qualifying as SAEs......118
11.7 Time Period and Frequency of Detecting AEs and SAEs......118
11.8 Method of Detecting AEsand SAEs......119
11.9 Recording of AEs and SAEs......119
11.10 Evaluating AEs and SAEs......119
11.10.1 Assessment of Intensity......119
11.10.2 Assessment of Causality......120
11.11 Follow-Up of AEs and SAEs......120
11.12 Prompt Reporting ofSAEs to Sponsor......120
11.13 Regulatory Reporting Requirements for SAEs......121

[redacted] 121
[redacted] 121
[redacted] 121
[redacted] 121
[redacted] 122
13 PREDICTIVE AND PHARMACODYNAMIC BIOMARKERS 122
13.1 Tumor Tissue for BRAFV600E Mutation Pre-Screening 122
13.2 Predictive Biomarker Research 122
13.2.1 Peripheral Blood Sample Collection and Analysis 123
13.3 Exploratory Ex Vivo Assay: HCL Cohort Only 123
14 PHARMACOGENETICS 124
[redacted] 124
[redacted] 124
[redacted] 124
[redacted] 124
16 COMPLETION OR WITHDRAWAL OF SUBJECTS 125
16.1 Screen Failures 125
16.2 Criteria for Permanent Discontinuation of Study Treatments 125
16.3 Follow-Up Assessments after Discontinuation of Study Treatment or Study Withdrawal 126
16.4 Subject Completion 126
16.5 Study Completion 126
16.6 Subject Withdrawal Procedures 127
17 DATA MANAGEMENT 127
[redacted] 127
18 DATA ANALYSIS AND STATISTICAL CONSIDERATIONS 128
18.1 Hypotheses and Treatment Comparisons 128
18.2 Sample Size Considerations 129
18.2.1 Sample Size Assumptions 129
18.2.2 Sample Size Sensitivity 129
18.2.3 Sample Size Re-estimation 129
18.3 Data Analysis Considerations 130
18.3.1 Analysis Populations 130
18.3.2 Interim Analysis 130
18.3.3 Final Analyses 131
18.4 Simulations and Design Operating Characteristics 134
18.4.1 Simulation Description 134
18.4.2 Operating Characteristics 136
18.4.3 Example Trials 144

19 STUDY CONDUCT CONSIDERATIONS..144
19.1 Posting of Information on Publicly Available Clinical Trial Registers 144
19.2 Regulatory and Ethical Considerations, Including the Informed Consent Process 144
19.3 Data Monitoring Committee..145
19.4 Quality Control (Study Monitoring) 145
19.5 Quality Assurance 146
19.6 Study and Site Closure..146
19.7 Records Retention 146
19.8 Provision of Study Results to Investigators, Posting of Information on Publicly Available Clinical Trials Registers and Publication 147
20 REFERENCES 147
21 APPENDICES 155
21.1 Appendix 1: ECOG Performance Status 155
21.2 Appendix 2: NYHA Functional Classification System 156
21.3 Appendix 3: Cockcroft-Gault Formula for Creatinine Clearance 156
21.4 Appendix 4: Pharmacogenetic(PGx) Research 157
[redacted] 161
21.6 Appendix 6: Details of Bayesian Hierarchical Model 163
21.7 Appendix 7: Supporting Simulation Results 165
21.8 Appendix 8: Hypothetical Example Trial Results. 166
21.9 Appendix 9: Country-Specific Requirements 178
21.10 0 Appendix 10: RECIST, version 1.1 180
21.11 Appendix 11: Response Criteria for NSGCT/NGGCT with Elevated Tumor Markers (AFP and β-HCG) at Baseline 185
21.12 Appendix 12: Response Criteria for HCL...185
21.13 Appendix 13: RANO Response Criteria: Response Assessment Criteria for WHO Grade 1 or 2 Glioma 189
21.14 Appendix 14: RANO Response Criteria: Updated Response Assessment Criteria for WHO Grade 3 and 4 Glioma 192
21.15 Appendix 15: Consensus Recommendations for the Uniform Reporting of Clinical Trials 195
21.16 Appendix 16: Imaging Guidelines for WHO Grade I or II Glioma 198
21.17 Appendix 17: Volumes of Collected Blood Samples 200
21.18 Appendix 18: Liver Safety Drug Restart or Rechallenge Guidelines 201
21.19 Appendix 19: Protocol Amendment Changes 205

AMENDMENT 09 (12-Feb-2019)

Amendment Rationale

As of the end of enrollment on 29-Jun-2018 the study had enrolled a total of 206 subjects. As of 04-Feb-2019, 85 patients were ongoing.

The primary purpose of amendment 9 is to revise female contraception requirements as follows,

1. Double barrier contraception methods are no longer allowed in the study as they are not classified as highly effective methods per the Clinical Trial Facilitation Group (CTFG) and Novartis internal guidelines.
2. For subjects taking trametinib, contraception using oral contraceptives are not allowed while the drug interaction study between trametinib and oral contraceptives is still ongoing and Novartis would like to take a conservative approach and restrict oral contraceptives until the results are available from the interaction study.

Throughout the document where contraception requirements after discontinuation of study treatments are discussed, language is clarified as follows,

"Women of childbearing potential should use effective methods of contraception during therapy and for 16 weeks following discontinuation of trametinib when taken in combination with dabrafenib, or for 2 weeks following discontinuation of dabrafenib monotherapy."

The Study Completion (Section 16.5) and Data Analysis and Statistical Considerations (Section 18) sections have been amended as they relate to study completion and data analysis. A new end of study definition is implemented.

Justification

At the time of Interim Analysis #14, 54 of 178 subjects had died, representing a death rate of approximately 30%. As a result, a new end of study definition is implemented with this protocol amendment to accommodate the better than expected outcome in this study. A minimum follow-up period of approximately 2 years for all patients is sufficient for collection and maturity of data for the analysis of the secondary endpoints, and will be the new end of study definition.

Details of the specific changes made in each section of the protocol are described below in the Changes to the Protocol section below.

Changes to the Protocol

Changes to specific sections of the protocol are shown in the track changes version of the protocol using strike through red font for deletions and red underline for insertions.

- Section 9.1.1. Contraception Requirements for Female Subjects.
  - Updated per change to contraception requirements (see above).
- Section 16.5. Study Completion.
  - Updated definition for study completion. Study will be considered complete at a date that would represent a minimum follow up of approximately 2 years for all subjects enrolled."

  - Updated language clarifying the possible options for alternative supply of study treatment for those subjects which continue to derive clinical benefit at study completion.
- Section 18.3.1. Analysis Populations.
  - Clarified the analysis population for supportive final efficacy analysis
- Section 18.3.3. Final Analyses.
  - Updated date of final analyses as the date representing a minimum follow up of approximately 2 years for all subjects enrolled.
- Section 18.3.3.2 Efficacy Analysis
  - Removed reference to pooled ORR calculations across histologies.
- Changes to Medical Lead Contact, Clinical Trial Head Contact and Signatory.
- Formatting and Typographical errors corrected throughout the document.

IRB/IEC/REB Approval

A copy of this amended protocol will be sent to the Institutional Review Boards (IRB)/Independent Ethics Committees (IECs), Research Ethics Boards (REBs) and Health Authorities.

The changes described in this amended protocol require IRB/IEC/REB approval prior to its implementation.

The changes herein affect the Informed Consent. Sites required to update and submit for approval a revised Informed Consent that takes into account the changes described in this amended protocol.

AMENDMENT 08 (14-Dec-2017)

Amendment Rationale

As of 20-Nov-2017:

- One hundred fifty five patients have been enrolled in 35 sites located in 13 countries (Austria, Belgium, Canada, Denmark, France, Germany, Italy, Korea, Netherlands, Norway, Spain, Sweden and the United States).
- Eighty patients have discontinued the study treatments (60 due to disease progression, 12 due to adverse events, 7 due to patient or investigator decision and 1 due to a protocol deviation).
- Forty-seven patient deaths have been reported of which three occurred during study treatment with the reason noted as sepsis for all the deaths.

The primary purpose of amendment 8 is to implement a change in the duration of contraception measures for male participants. The duration of male contraception measures are increased from 12 to 16 weeks in the period following stopping of the study treatments. This change was prompted by re-evaluation of the existing pre-clinical data and the application of Novartis contraception guidance. Furthermore, amendment 8 implements a change in the IND number under which the study is conducted, to comply with a request from the Food and Drug Administration (FDA) to administratively split Study CDRB436X2201 from IND#113557 and to file this study under a new IND (137160) and, other minor changes or clarifications to the study procedures.

Details of the specific changes made in each section of the protocol are described below in the Changes to the Protocol section below.

Changes to the Protocol

Changes to specific sections of the protocol are shown in the track changes version of the protocol using strike through red font for deletions and red underline for insertions.

- Sponsor Information Page. Updated the US IND # to 137160 (Previously 113557).
- List of Abbreviations. Added alloSCT (allogenic stem cell transplantation) and autoSCT (autologous stem cell transplantation) and removed ASCT (allogenic stem cell transplantation) to avoid ambiguity.
- Section 7.1 Time and Event Table. Added the following changes:
    - Table headings. Added "All Cohorts" or "Cohorts" to the applicable Assessment Headings for clarity,
    - Pregnancy[19] Under "Every 12 weeks (+/-3 days)" heading, added "X[21] serum or urine" to align with footnote [21] instructing every 12 week testing requirement after week 48,
    - TSH, Free T4. Added (ATC only) to clarify these tests are only required for patients enrolled to the ATC cohort,
    - Blood sample for CBC. Under "every 4 weeks (+/-3 days)" and "every 8 weeks (+/-3 days)" headings, added [31] and X[31] respectively to clarify and align with footnote [31],
    - Peripheral Blood Sample Staining for Hairy Cell Count. Under "every 4 weeks (+/-3 days)" and "every 8 weeks (+/-3 days)" headings, added footnote [31] and X[31] respectively, to clarify and align with the text noted in footnote [31],
    - Flow Cytometry: peripheral blood sample[33]. Under "every 4 weeks (+/-3 days)" heading, added [31] to clarify and align with the text noted in footnote[31],
    - Footnote [31]. Modified the instruction for HCL patients who are tolerating study drug treatment beyond week 48. These patients may reduce the frequency of response assessment evaluation from every 4 weeks (+/-3 days) to every 8 weeks (+/-3 days) if appropriate in the judgement of the treating investigator.
    - Footnote 58. Modified the instruction for extended follow-up as follows:

[58]**EXTENDED FOLLOW-UP:** The following evaluations will be performed for all patients in all cohorts

a. Dermatologic exam. Exams will be performed every 4 weeks (+/-7 days) for the first 6 months. The skin will be carefully examined and pictures and biopsy will be taken of any new or changed skin lesions.
b. If study treatments are permanently discontinued prior to the subject having confirmed disease progression, the subject will continue to have disease assessments every 12 weeks (+/-14 days) until disease progression or initiation of new anti-cancer treatment, or death, whichever comes first.
c. Subjects will be followed every 12 weeks (+/-14 days) for survival until the subject dies or withdraws consent or is lost to follow up, or up to a time period as defined in Section 16.3. Survival follow up may be performed via clinic visit, a telephone contact, an email or by mail. The initiation of any new anti-cancer treatment(s) and date of last contact should also be documented.

d. Subjects will have a head and neck exam performed every 12 weeks for the first 6 months to monitor for secondary or recurrent malignancy.
e. Subjects may have CT and/or MRI scans of the chest and abdomen every 6 months to monitor for secondary or recurrent malignancy.
    - Footnote List. Corrected previous mis-numbering error that caused inaccurate references for footnotes [39] through [58].
- Section 7.4.8. Table 6. Added Footnote [6] to clarify that although HbA1c testing is included in "Clinical Chemistry" section it is performed at an interval of every 12 weeks (+/-3 days).
- Section 7.5.2.1. Laboratory and Disease Assessments: HCL. Updated post-baseline assessments statement to align with change indicated in footnote 31 of Time and Event Table.
- Section 9.1.2. Added a new section to include the contraception requirements for male subjects.
    1. "Male subjects (including those that have had a vasectomy) must use a condom during intercourse while taking dabrafenib and/or trametinib treatment. Once dabrafenib and trametinib are both premanently discontinued, the patient must use a condom for 16 weeks after the last dose of the study treatments. The patient should not father a child during the study treatment period or during the specified time frames after discontinuation of study treatment."
- Section 10.3. Medications to be used with Caution. Removed statement regarding Dabrafenib solubility at higher pH. Consequently, proton pump inhibitors removed from Table 28 of Medications to be used with Caution.
- Section 11.7. Reinstated a sentence outlining the time period for detecting adverse events and serious adverse events inadvertently removed at amendment 7.
- [redacted]
- [redacted]
- [redacted]
- Section 21.13. Appendix 13: RANO Response Criteria: Response Assessment Criteria for WHO Grade 1 or 2 Glioma. Clarified the second bullet point under "Disease Progression (PD)",

  (Bolded text below added to statement as previously written)

  "A 25% increase **in the sum of the products of perpendicular diameters** of the T2 or FLAIR enhancing or non-enhancing lesion on stable or increasing doses of corticosteroids compared with baseline scan or best response after initiation of therapy, not attributable to radiation effect or to comorbid events."

IRB/IEC/REB Approval

A copy of this amended protocol will be sent to the Institutional Review Boards (IRB)/Independent Ethics Committees (IECs), Research Ethics Boards (REBs) and Health Authorities.

The changes described in this amended protocol require IRB/IEC/REB approval prior to its implementation.

The changes herein affect the Informed Consent. Sites required to update and submit for approval a revised Informed Consent that takes into account the changes described in this amended protocol.

AMENDMENT 07

Amendment Rationale

Subsequent to the acquisition of GlaxoSmithKline (GSK) compounds GSK1120212 and GSK2118436, the purpose of this protocol Amendment 07 is to:

- Delete or replace references to GlaxoSmithKline or its staff with that of Novartis and its authorized agents to align with the change of sponsorship;
- Make administrative changes to align with Novartis processes and procedures;

As of 7-Feb-2017:

- 121 patients have received study treatment in 11 countries (Austria, Belgium, Canada, Denmark, France, Germany, Italy, Korea, Netherlands, Norway and US);
- 63 patients have completed or discontinued study treatment.

The changes described in this amended protocol require IRB/IEC approval prior to implementation.

A copy of this amended protocol will be sent to the Institutional Review Board (IRBs)/Independent Ethics Committee (IECs) and Health Authorities (HAs).

The changes herein affect the Informed Consent and all sites are required to update and submit for approval, a revised Informed Consent that takes into account the change of study sponsorship described in the protocol amendment.

Upon approval of this amendment, patients who have already been enrolled in the study will sign a new informed consent form indicating Novartis is the new study sponsor and continue the appropriate visit schedule.

REVISION CHRONOLOGY

GlaxoSmithKline Document Number	Date	Version
2012N145247_00	2013-JUL-09	Original
2012N145247_01	2013-JUL-25	Amendment No. 1
• List of Authors was updated to add [redacted] and [redacted] and to remove [redacted] due to changes in study assignments within GSK.<br>• Previous primary Medical Monitor [redacted] was replaced by [redacted] (formerly secondary Medical Monitor) and [redacted] named secondary Medical Monitor.<br>• EudraCT Number was added.<br>• List of Abbreviations updated to insert any abbreviations omitted in original protocol		

- Section 7.3.8, Clinical Laboratory Assessments, Table 4, removed cardiac enzyme: troponin from list of assessments to be completed.
- Section 7.4.1, Physical Examination, revised to indicate that other medical personnel (i.e., physician assistant or nurse practioner) may be able to perform the physical examinations
- Section 8: Time and Event tables were revised to correct for inconsistencies with individual cohort text pertaining to samples to be obtained for BRAF mutation status, disease assessment, and biomarker assessments.
- Section 8: Time and Events tables were revised the frequency of select assessment for the first 52 weeks of treatment to 48 weeks of treatment to align with the timing of other assessments
- Section 8.1.2, Section 8.2.2, Section 8.3.2, Section 8.4.2, Section 8.5.2, Section 8.6.2, Section 8.7.2, Inclusion Criteria: clarified mandatory tumor sample required at Screening
- Section 8.8.2 and Section 8.9.2, Inclusion Criteria: clarified mandatory BM aspirate sample required at Screening
- Section 8.6.2, Section 8.7.2, Section 8.8.2, and Section 8.9.2, Exclusion Criteria: added text to confirm that no histology-specific exclusion criteria were included
- Section 8.4.3 and Section 8.5.3, Disease Assessments: corrected reference to CT scan; changed to MRI as CT is not permitted in these cohorts
- Section 8.6.4, Laboratory Assessments: corrected reference in cohort number
- Section 9.2.3.4, Restarting Study Treatment was revised to remove reference to restart of study treatment.

[redacted]

2012N145247_02	2013-DEC-18	Amendment No. 2

- Section 3, Objectives and End points, revised Table 3 primary endpoint to reflect use of Modified RANO and RANO is assessing tumor response in gliomas.
- Section 4, Study Design: revised sentence regarding eligibility of subjects to be consistent with new general inclusion criteria #5; revised to correctly identify timing of screening assessments and exceptions for consistency with Time and Events Tables.
- Section 5.2.1, General Inclusion Criteria #5: revised to delete the reference to a CLIA approved laboratory as this certification is not applicable to countries other than the US.
- Section 5.2.1, General Inclusion Criteria #7 (renumbered to #8): revised to describe contraception consistently with other sections of protocol; added “highly” to effective contraception.
- Section 5.2.1, General Inclusion Criteria (new) #5: new criteria added to clarify inclusion of subjects with specified histology and no available treatment options per local or regional standard of care.
- Section 7.3, Safety: inserted new text to provide estimation of blood volume collected during the study and to reference Appendix 17.

- Section 7.3.1, Physical Examination: replaced term 'neurological' to 'CNS' to better describe assessment to be made.
- Section 7.3.6, Electrocardiogram, second paragraph, revised to remove reference to QTcF as stopping criteria for QTc prolongation is limited to use of Bazett formula for correction.
- Section 7.3.7, Echocardiogram, revised to remove the use of MUGA scans to assess cardiac ejection fraction.
- Section 7.3.9, Monitoring of Non-Cutaneous Secondary/Recurrent Malignancy, added as a new section to reflect monitoring required per the special warnings and precautions required for use of dabrafenib.
- Section 8.3.2, Eligibility Criteria: GIST, added new inclusion criteria (#4) requiring progression with imatinib and sunitinib treatment; subsequent criteria were renumbered.
- Section 8.4.2, Eligibility Criteria: WHO Grade 1 or 2 Glioma: added new criteria for subjects with WHO Grade 2 glioma: only subjects for whom chemotherapy are not an option may be eligible for study participation
- Section 8.8.2, Eligibility Criteria: HCL, Inclusion Criteria #2, revised inclusion criteria
- #2 to provide examples of a purine analog and to clarify definition of refractory disease.
- Section 8.9.2, Eligibility Criteria: MM, Inclusion Criteria #2, revised to clarify prior treatment received by subjects with MM.
- Section 9.2.1, Cardiovascular Events, revised to indicate that cardiovascular events may occur with not only trametinib but also dabrafenib or both in combination.
- Section 9.2.1.1, QTc Prolongation, revised to be in line with the special warnings and precautions required for use of dabrafenib.
- Section 9.2.1.2, Left Ventricular Ejection Fraction Stopping Criteria, revised to remove use of MUGA scan and to request procedure to be performed by same operator throughout the study; removed requirement of reporting symptomatic events as SAE, and to provide that in case of asymptomatic absolute decrease of >10% in LVEF compared to baseline and ejection fraction below the institutional LLN, study treatment (trametinib and dabrafenib) must be temporary or definitely discontinued.
- Section 9.2.1.3.2, Management of Hypertension, clarified dose added new text directing subjects with persistent increase in systolic and/or diastolic BP that may be treatment-related to be managed by recommendations in Table 21 and revised to indicate that continuing treatment by dabrafenib in case of asymptomatic or symptomatic hypertension is not justified. In this situation, study treatment (trametinib and dabrafenib) must be temporary or definitely discontinued.
- Section 9.3.1.2, Cutaneous SCC, revised to remove reference to keratoacanthomas and added requirement of dermatological examinations monthly for 6 months after treatment discontinuation.
- Section 9.3.5, Renal Insufficiency, revised to include guidelines for management and dose reduction for renal insufficiency when considered treatment related (Table 26 added). (Tables from this point forwarded were renumbered accordingly.)

- Section 10.1.1, Female Subjects, revised in order to describe contraception consistently with other sections of protocol; added “highly” to effectivecontraception
- Section 11.3, Medications to be Used with Caution, revised to add updated reference for drugs known to induce QTc prolongation.
- Section 14.1, Tumor Tissue for BRAF V600E Mutation Pre-Screening, revised to delete the reference to a CLIA approved laboratory as this certification is not applicable to countries other than the US.
- Section 20.1, Data Monitoring Committee, new section added to address the formation of a data monitoring committee for review of data during interim analysis and to indicate that independent hematologist and oncologist will serve on data monitoring committee.
- Appendix 9, Country Specific Requirements, deleted text pertaining to French-specific QTc stopping criteria as the criteria was incorporated into Section 9.2.1.1.
- Appendix 17, Volumes of Collected Blood Samples, added new table to provide the volume of blood collected at various time points during study.

2012N145247_03	2014-JUL-21	Amendment No. 3

- Amendment No.: 03 was prepared to revise the trametinib and dabrafenib sections in order to remove information that was outdated and to reduce information that is found in product IBs.
- Revised information pertaining to BRAF mutation test and companion diagnostic.
- Reformatted the inclusion/exclusion criteria to eliminate repeated language found when previously presented in separate cohort sections.
- Revised inclusion/exclusion cohort where appropriate based on additional input from investigators or subject experts or to clarify criteria; revised text pertaining to treatment of overdose for both dabrafenib and trametinib to align with new standard protocol language.
- Condensed 9 separate Time and Events table into one table to eliminate duplication of information for ease of user; added language pertaining to retrospective confirmation of histology type for ATC cohort.
- Revised vision changes and ophthalmic exam language in appropriate sections with revised standard asset language.
- Reformatted document to eliminate individual cohort sections and reduce duplication of text describing study assessments such as laboratory and disease assessment, etc.
- Revised disease assessment sections to clarify type of assessment, timing and evaluation criteria.
- Stopping criteria and management and dose modification for special events were revised to reflect changes in standard asset language.
- Liver chemistry stopping criteria were revised to reflect recent changes in standard protocol language to be used.
- Concomitant medication and non-drug therapies section was revised to clarify use of anticoagulants, palliative radiation and use of dabrafenib during radiotherapy.

- Add Ex Vivo substudy for HCL cohort.
- Addressed change in information collected for screenfailures.
- New reference citations added, references no longer cited in text were removed.
- List of abbreviations updated to reflect revisions in overall text.
- Other general administrative changes were made to correct typographical errors, etc.

2012N145247_04	2014-OCT-24	Amendment No. 4

The changes made with Amendment No. 4 are to revise the protocol in response to the recent decision for the substantial amendment of a Voluntary Harmonization Procedure (VHP-SA) submission of Amendment 03 of the protocol. Specific changes made with Amendment No. 04 include the following:

- Section 1.3: revised to update regulatory approval status of trametinib monotherapy and trametinib in combination with dabrafenib
- Section 4.0: revised to correct inconsistency between text in protocol section and text change noted in summary of amendment changes in Appendix 20 for Amendment 03.
- Section 5.2.1.1.4 and Section 5.2.1.1.5: revised Inclusion Criteria #5 and #4, respectively, to clarify that the criterion applies to subjects who are already receiving corticosteroid therapy.
- Section 5.2.2: revised to Criterion #1 to clarify that the status of delayed toxicity applies to all types of therapy and not solely chemotherapy.
- Section 8.1.1.2, Table 12: reverted text in Table 20 to the Amendment 02 version as it was previously approved by the VHP.
- Section 8.1.3.1, Section 8.1.3.1.1, Section 8.1.3.1.2, Section 8.1.3.1.3: the liver chemistry stopping criteria sections were revised to correct omission of a portion of the criteria and to reformat the text originally found in Appendix 18 in Amendment 03 to assure that all guidelines were located together in Section 8.1.3.
- Section 8.2: revised to delete table that was incorrectly placed under this section heading; should have been included in previous section of protocol
- Section 8.2.3: revised based on recent revision of the asset standard language for pneumonitis
- Section 8.2.6, Table 21: revised text in Table 21 to align with the Summary of Product Characteristics (SmPC) language as requested by the VHP
- Section 13.1: revised to correct inconsistency between text in protocol section and text change noted in summary of amendment changes in Appendix 20 for Amendment 03.
- Other changes were made due to reassignment of GSK personnel (i.e., list of authors, secondary medical monitor and sponsor signatory for protocol).

2012N145247_05	2015-APR-28	Amendment No. 5

Amendment No. 05 was prepared to revise information pertaining to BRAF mutation test and companion diagnostic

- Revised inclusion/exclusion cohort where appropriate based on additional input from investigators or subject experts or to clarify criteria.
- Revised language in appropriate sections with revised standard asset language.
- Clarified who can perform physical exams.
- Clarified number of blood pressure readings to be taken and averaged value to be reported.
- Revised disease assessment sections to clarify type of assessment, timing and evaluation criteria.
- Revised LVEF stopping criteria to indicate when to report as SAE.
- Removed BRAT diet from diarrhea management guidelines.
- Removed oral contraceptives from the prohibited medications list and provided supporting information regarding interaction with dabrafenib; specified oral formulation for selected prohibited medications and medications to be used with caution.
- Concomitant medication and non-drug therapies section was revised to clarify use of NSAIDs.
- Clarified which samples to be submitted for confirmation of BRAF mutation status.
- Definition of SAEs revised for protocol specific SAEs based on updated list of AE of special interest.
- Added new section for malignancies to include section on cutaneous squamous cell carcinoma, new primary melanoma and non-cutaneous malignancies based on updated asset language for dabrafenib and trametinib.
- Clarified use of NSAIDs in subjects with MM for pyrexia and action to be taken with dabrafenib with pneumonitis.
- Revised disease assessments for solid tumors to clarify imaging modality to be used for specific cohorts.
- Statistical section revised to reflect change in study sample size and trial simulation output.
- [redacted]
- Revised Bayesian hierarchical model specification to correct typographical error.
- Updated reference citations, list of abbreviations updated to reflect revisions in overall text, and other general administrative changes were made to correct typographical errors.

2012N145247_06	2016-JAN-05	Amendment No. 6

Amendment 6 implements additional updates to the protocol Sections noted below:

- Section Sponsor/Medical Monitor Information Page: Updates the contact details for Medical Monitor and Sponsor Contact information.

- List of Abbreviations: Adds “SPEP – Serum Protein Electrophoresis” and “UPEP - Urine Proteine Electrophoreis” to the list.
- Section 1.2 Dabrafenib (GSK2118436): Updates the last paragraph as follows: Refer to the dabrafenib monotherapy and dabrafenib+trametinib combination therapy and updates the new IB v.07 document number.
- Section 1.3 Trametinib (GSK1120212):
  - Updates the second paragraph as follows: “….Trametinib in combination with dabrafenib has been approved in the US, **EU**, Chile, and Australia for the treatment of patients with unresectable or metastatic melanoma with BRAF V600E or V600K mutation.
  - Updates the third paragraph as follows: Refer to the **dabrafenib monotherapy and dabrafenib** IB for a complete summary….. and updates the new IB document number.
- Section 1.4 Risk Assessment for Dabrafenib + Trametinib Combination Therapy:
  - Adds a new paragraph about the the safety profile for dabrafenib + trametinib combination therapy.
  - Updates the guidance and timepoints for dermatolgical exams for cuSCC as follows: A brief dermatological exam should be performed every 4 weeks (±7 days) for the first 6 months after discontinuation of study treatments to monitor for new cutaneous malignancies.
  - Updates information for Pyrexia as follows: … About **one-third** of the subjects who experienced pyrexia had **3 or more** events.
  - Adds the following guidance: **trametinib should be continued at the same dose.**
  - Adds information for decrease left ventricular ejection fraction as follows: Left ventricular dysfunction has been reported with trametinib as well as with other MEKi in clinical development. **In the 2 Phase III studies decreased ejection fraction occurred in 7% of subjects receiving combination therapy and the majority of the LVEF decreases that met interruption criteria were asymptomatic and resolved. Most subjects who were re-challenged were able to continue on treatment without further dose modification.** Subjects enrolled in studies with dabrafenib in combination with trametinib undergo regular cardiac assessments with echocardiograms (ECHOs). Guidelines for clinical management of treatment-related LVEF decreases have been provided in Section 8.1.1.2.
  - Adds new information for haemorrhage as follows: **Haemorrhage: Haemorrhagic events, including major haemorrhagic events defined as symptomatic bleeding in a critical area or organ, and fatal intracranial haemorrhages, have occurred in patients taking dabrafenib in combination with trametinib. The majority of bleeding events were mild. Three of 209 subjects (1%) receiving trametinib in combination with dabrafenib in a phase III trial had fatal intracranial haemorrhagic events. The management of hemorrhagic events may require treatment interruption, dose reduction, or treatment discontinuation**.

- o Updates new information for Hyperglycemia as follows: **Hyperglycemia**: Hyperglycemia requiring an increase in the dose of, or initiation of, insulin or oral therapy can occur with dabrafenib **or dabrafenib in combination with trametinib**.
- o Updates information for Pancreatitis as follows: Pancreatitis: Subjects **receiving dabrafenib or dabrafenib in combination with trametinib** have reported AEs of acute pancreatitis or pancreatitis. In some cases, serious adverse events (SAEs) of Grade 4 pancreatitis were reported as treatment-related by the investigator. The time to onset of pancreatitis ranged from study Day 21 to 292 (median 138 days) **with dabrafenib monotherapy, and from Study Day 24 to 312 (median 50 days) with combination therapy**. Discontinuation of study treatment due to pancreatitis was not deemed necessary in any of the cases by the investigators.
- o Updates information for Fertility as follows: **Trametinib may impair fertility in humans**.
- o Updates information for Pregnancy as follows: Women of childbearing potential should use effective methods of contraception during therapy and for 4 months following discontinuation of trametinib **when taken** in combination with dabrafenib, or for 4 weeks following discontinuation of dabrafenib.

- Section 1.5.6 Cohort 6: Non-Seminomatous/Non-Germinomatous Germ Cell Tumors (NSGCT/NGGCT): Updates the second paragraph as follows: Chest pain and difficulty breathing are early signs of NSGCT. Other symptoms include: cough, fever, headache, **and change** in bowel habits, fatigue, difficulty walking and visual problems (difficulty seeing or moving eyes).
- Section 2.1 Rational: Updates information about overall survival data for combination therapy in advanced melanoma.
- Section 2.3 Dose Rationale:
  - o Updates the trametinib [redacted] [GlaxoSmithKline Document Number to CM2010/00010/05 and modi[redacted] the last sentence"The combination was relatively well tolerated in study BRF113220, study MEK115306 **and study MEK116513**, with toxicities manageable with appropriate intervention.
  - o Modifies the second paragraph as follows: As determined in Phase III studies, the safety profile of the combination of dabrafenib and trametinib generally reflects the well established safety profiles of the individual approved agents, with toxicities that are manageable with appropriate intervention.
- Section 4 Study Design:
  - o Updates the first paragraph as follows: "…For each cohort, up to 25 evaluable subjects will be enrolled **in the primary analysis cohort as defined in Section 5.1**. **If a given cohort is stopped early for efficacy, a histology specific expansion cohort may be opened to allow for additional patient enrollment (see Section 5.2)**"
  - o Updates the fourth paragraph regarding disease assessments as follows: "Subjects will undergo disease assessment of response after receiving at least 8 weeks of treatment"

- Section 5.1 Primary analysis cohort: Adds new section as follows: The "primary analysis cohort" will be comprised of those patients enrolled within a histology-specific group prior to capping at 25 patients per cohort or prior to early stopping for efficacy or futility. The primary analysis cohort will form the basis of the Bayesian modelling.
- Section 5.2 Expansion cohort: Adds a new section as follows: If a cohort closes early at an interim analysis because it meets the rules for early stopping for efficacy, an expansion cohort may be opened to allow additional patient enrollment for that particular histology. The patients in the expansion cohort will provide supportive efficacy data and will NOT contribute to the **Bayesian modeling. The expansion cohort (s) will enroll subjects for the duration of trial enrollment. The same general and histology specific eligibility criteria and T&E tables and biomarker/histology confirmation analyses will apply to patients in the expansion cohort.**
- Section 5.1 became 5.3 Number of Subjects: Updates first paragraph as follows: **For the primary analysis cohort,** enrollment will not exceed 25 subjects per histology cohort to ensure adequate subjects in the BRAF V600E confirmed population. After 25 subjects have been enrolled, the histology cohort will be closed to further enrollment. See Section 18.2 for sample size assumptions.
- 5.4 Eligibility: Updates reference to updated for the IB of dabrafenib + trametinib combination
- Section 5.4.1 Inclusion Criteria Table 3 Adequate Baseline Organ Function: Updates legend 2 as follows: **PTT and PT**/INR >1.5 times ULN will be acceptable in case of subjects receiving therapeutic anticoagulants such as warfarin as long as INR is monitored during the study according to clinical practice.
- 5.4.2 Exclusion Criteria: Modifies the time window for radiotherapy for the Glioma Grade 1-4 cohort only: Subjects may be ≥ **2** weeks from radiotherapy if a new lesion relative to the pre-radiation MRI develops outside the primary radiation field.
- 5.4.2.1.3 WHO Grade 1, 2, 3 or 4 Glioma: Adds additional clarification to the Note concerning the WHO Grade 1,2,3 or 4 Gliom as follows: NOTE: Extended period of time (>3 months) needed to prevent subjects with pseudo-progression from radiotherapy being enrolled in the study. **Subjects may be ≥2 weeks from radiotherapy if a new lesion relative to the pre-radiation MRI develops outside the primary radiation field. Treatment-related AEs must have resolved prior to enrollment.**
- Section 7.1 T&E Table: Clarifies the assessment timelines and corrects discrepancies between the T&E Table and footnotes as follows:
  - Updates footnote and legend numbering as deemed necessary.
  - Updates footnote 4: Subjects **may** be enrolled based on local determination of BRAF V600E mutation status. A subject may be tested for the BRAF V600E mutation greater than 28 days prior to enrollment NOTE: For subjects with HCL or MM: A fresh BM aspirate sample and a peripheral blood sample are required for confirmation testing by a sponsor designated central reference laboratory. If a BM aspirate cannot be obtained due to a dry tap, please contact the Medical Monitor. **In the event of a dry tap, fresh or archived tumor tissues such as lymph node or plasmacytoma biopsies may be acceptable pending medical monitor approval.**

- Updates footnote 6: All screening assessments must be completed within 14 days prior to first dose except informed consent. Disease Assessment (if MRI is used) can occur within 35 days prior to the first dose. Note: Procedures conducted as part of the subject's routine clinical management(e.g., blood counts, imaging study) and obtained prior to signing of informed consent may be utilized for screening or baseline purposes provided these procedures meet the protocol requirements.
- Deletes footnote 7: Screening ECGs, ECHO, laboratory assessments and tumor marker tests must be performed within 3 days prior to the first dose of study treatments.
- Foot Note 12, 14 and 17: Clarifies the time window for screening assessment forthe dermatological exam, ophthalmic exam and ECHO by adding "**(or within 35 days prior to the first dose)**".
- Implements "Tissue Sample for Histology Confirmation" forindependent pathology confirmation of ATC and WHO Grade 1-4 Glioma cohort only and corresponding footnote 28 (A tissue specimen for possible retrospective histology confirmation should be obtained and submitted to a GSK designated central reference laboratory. Please refer to the SPM for the requirements of the tissue specimen that is required for the histology confirmation.
- Updates foot note 29: **AFP, B-HCG and LDH are the disease assessment markers for the NSGCT/NGGCT cohort.** LDH result obtained from clinical chemistry panel can be used as a tumor marker result. Regardless of baseline values of AFP, β-HCG and LDH, a blood sample will be drawn every 4 weeks for first 48 weeks of study treatment, then every 8 to 12 weeks thereafter, and at the time of disease progression. Synchronize the collection of the blood sample with the imaging schedule.
- Updates foot note 31 for HCL cohort only as follows: All required disease assessments necessary for a post-baseline response determination should be performed every 4 weeks +/- 3 days. A response is to be determined every 4 weeks based on the response criteria (please consult Appendix 12) using data collected from the scheduled disease assessments.
- Clarifies foot note 32 for the HCL cohort only as follows: **BM biopsy and aspiration with H/E stain and IHC will be performed when blood counts are consistent with CR or PR for 4 weeks. If a patient has a CR or PR, and then blood counts become consistent with a CR or PR for 4 weeks, a repeat BM aspirate and biopsy is required to assess a response of CR or PR. Following best response, a bone marrow biopsy and aspirate must be performed every 6 months for two time intervals, then after a year for two time intervals, then every two years.**
- Clarifies Disease assessments post baseline (BM biopsy and aspirate with H/E stain and IHC and BM aspirate with flow cytometry) for HCL cohort only within corresponding section in T&E table as follows: When counts are consistent with CR **or PR** for 4 weeks; if evidence of response, then **after** every 6 months twice, then **after a** yearly twice **and** then every 2 years
- Clarifies foot note 33 for the HCL cohort only as follows: Recommend that flow cytometry panel consists of CD19, CD20, CD22, SmIg, CD11c, CD25, CD103 and CD123. **Flow cytometry of the bone marrow aspirate sample will be performed**

when blood counts are consistent with CR or PR for 4 weeks (please consult Appendix 12). If a patient has a PR, and then blood counts become consistent with a CR for 4 weeks, a repeat flow cytometry of the BM aspirate is required to assess a response of CR. Following best response, a flow cytometry of the bone marrow aspirate must be performed every 6 months for two time intervals, then after a year for two time intervals, then every two years.

- Adds foot note 34 for HCL cohort as follows: **BM biopsy (with H/E stain and IHC), BM aspirate (with flow cytometry) and peripheral blood sample (with flow cytometry) will be assessed within 28 days prior to enrollment.**
- Clarifies foot note 35 regarding the timelines for tumor assessment (chest CT scan, abdominal CT scan with contrast and MRI (if needed) at baseline for the HCL cohort only as follows: A chest CT scan and an abdominal CT scan with contrast will be performed **within 28 days prior to enrollment**. If an abdominal CT scan with contrast cannot be performed, then a MRI may be performed **within 35 days prior to enrollment**. If lymph nodes or spleen are enlarged at baseline, then the abnormal organ needs to be followed on post-baseline assessments using the same modalityof imaging.
- Clarifies Disease assessments post baseline (Chest CT scan and Apdominal CT scan w/o contrast) for HCL cohort only within corresponding section in T&E table as follows: When counts are consistent with CR **or PR** for 4 weeks; then 6 months later
- Clarifies foot note 36 regarding timelines of skeletal survey at screening for MM cohort only as follows: **A skeletal survey should be performed within 28 days prior to enrollment.** If myeloma associated bony lesions are present at baseline, skeletal surveys should be performed every 16 weeks or more frequently in order to assess response or upon clinical suspicion of progressive disease. Skeletal surveys typically consist of lateral radiographs of the skull, AP and lateral views of the spine, and AP views of the humeri, ribs, pelvis, andfemurs.
- Modifies foot note 37 for MM cohort only as follows: **Each patient should be clinically examined for soft tissue plasmacytomas / extramedullary disease at baseline and at each visit for response assessment.** If extramedullary disease is present at baseline, then Chest/Abdominal/Pelvis CT scan with contrast should be performed **to confirm and to determine size and location of the soft tissue plasmacytoma**. If no disease is identified at baseline, then subsequent scans are only required if clinically indicated. Alternative imaging modality (i.e., MRI) may be used if it is better to assess the lesion and approved by a Medical Monitor. The same imaging modality should be used for all subsequent assessments.
- Clarifies foot note 38 regarding the timelines for the screening assessments of the BM aspirate and BM biopsy for the MM cohort as follows: BM aspirate and BM biopsy samples will be assessed by IHC, flow cytometry, FISH and cytogenetics **within 90 days prior enrollment**.
- Clarifies foot note 40 regarding the timelines for the screening assessments of SPEP for the MM cohort only as follows: SPEP should be performed **within 28 days prior to enrollment**, every 4 weeks for the first 48 weeks of study treatment, then every 12 weeks thereafter, and at the Follow-up Visit.

- Clarifies foot note 42 regarding the timelines for the screening assessments of UPEP for the MM cohort only as follows: If UPEP is positive at Screening **(or within 28 days prior to enrollment)**, then a UPEP should be performed every 4 weeks for first 48 weeks of study treatment, then every 12 weeks thereafter, and at the Follow-up Visit.
- Clarifies foot note 43 regarding the timelines for screening assessments involving CRP, β2 microglobulin; immunoglobulins (IgG, IgA and IgM) and serum FLC assay for the MM cohort only as follows: CRP, β2 microglobulin; immunoglobulins (IgG, IgA and IgM) and serum FLC assay should be performed **within 28 days prior to enrollment**, every 4 weeks for the first 48 weeks of study treatment, then every 12 weeks thereafter, and at the Follow-up Visit.
- Clarifies foot note 50 regarding timelines for optional exploratory assessments for HCL Cohort only as follows: Blood samples (approximately 25 mL) will be collected from HCL subjects (see Section 13.2 for further details) **at the corresponding time point: Screening and at the Follow-up Visit.** The investigator should use their clinical judgment in determining whether it is appropriate for a subject enrolled in the HCL cohort to have this additional blood drawn. In addition, if the IEC or IRB does not approve the site for participation in the sub-study, no subjects at those sites will be included in the sub-study.

- Section 7.3.2 Baseline Documentation of Target and Non-Target Lesions for Solid Tumor Histologies: Clarifies time window for baseline assessments as follows: All baseline lesion assessments must be performed within 28 days of enrollment **except where indicated differently in the T&E table (Section 7.1).**
- Section 7.4.2 Dermatologic Evaluations: Clarifies timepoints as follows: **A complete or full body dermatological examination will be performed at the time points indicated in the Time and Event Table (Section 7.1) to identify any abnormal skin lesions.**
- Section 7.4.3 Ophthalmic Examinations: Updates as follows: "Subjects are required to have a standard ophthalmology exam performed by an ophthalmologist at the time points indicated in the Time and Event Table (Section 7.1)
- Section 7.4.7 Echocardiogram: Updates as follows "**ECHOs will be performed at the time points indicated in the Time and Event Table (Section 7.1)**"
- Section 7.4.8 Clinical Laboratory Assessments Table 6: Adds lab tests information as follows: Amylase and lipase [monitor via local laboratory when clinically indicated to evaluate certain AEs (i.e., abdominal pain, pancreatitis, etc.)
- Section 7.4.8.1 Modifies the header to read as follows: Additional Histology Specific Assessments **and Histology Confirmation**:
  - Adds a new paragraph for histology confirmation for the ATC cohort and Glioma G1-G4 cohort only as follows: **A tissue specimen from the biopsy of a primary or metastatic site that served as the basis for the**
  - **Original diagnosis must be submitted to a GSK designated central reference laboratory as soon as possible after enrollment for possible retrospective**

confirmation of histologic type. Refer to the SPM for details regarding sample submission.

- Section 7.49 Monitoring for Non-Cutaneous Secondary/Recurrent Malignancy: Modifies the second paragraph as follows: Abnormal findings should be managed according to standard clinical practice.
- Section 7.5.1 Disease Assments Methods for Glioma Grade 1-4 cohorts: Updates both Tables to indicate that MRI with contrast is the only method of tumor assessment allowed at baseline and post-baseline for this particular cohort.
- Section 7.5.2.1 Laboratory and Disease Assessments HCL:
  - Clarifies timelines as follows: “The following baseline disease assessments will be performed at the time points indicated in the Time and Event Table (Section 7.1)”
  - Clarifies that a BM aspirate is required at baseline.
  - Clarifies the timelines for post-baseline assessments as follows: **A response is to be determined every 4 weeks based on the HCL response criteria (please consult Appendix 12) using data collected from the scheduled disease assessments.**
  - Clarifies that a BM aspirate is required at post-baseline.
  - Modifies the the 4th bullet point (post-baseline) to read as follows “Flow cytometry on BM aspirate sample will be performed as indicated in the Time and Event Table (Section 7.1)”
  - Clarifies assessments of lymph nodes and spleen as follows: If lymph nodes and/**or** spleen were enlarged at baseline, they must be followed by post-baseline assessments using a consistent imaging modality as indicated in the Time and Event Table (Section 7.1)
- Section 7.5.2.2 Laboratory and Disease Assessment: MM:
  - Clarifies timelines of baseline assessments as follows: The following baseline disease assessments will be performed at the time points indicated in the Time and Event Table (Section 7.1):
  - Simplifies list of baseline assessments as follows: Extramedullary disease.
  - Simplifies list of baseline assessments as follows: UPEP
  - Modifies post-baseline assessment for MM cohort only as follows: Laboratory tests (SPEP, UPEP, CRP, β-2 microglobulin, serum FLC assay, levels of IgG, IgA, and IgM will be performed as indicated in the Time and Event Table (Section 7.1).
  - Modifies post-baseline assessment for MM cohort only as follows: Extramedullary disease assessment: If no disease is identified at baseline, then subsequent assessments are only required if clinically indicated.
- Section 8.1 Dose Modification:
  - Revises the dose modification guidelines for trametinib as follows: If a dose reduction of trametinib is required, but the toxicity resolves and no additional toxicities are seen after **one** treatment cycle **(4 weeks)**, the dose of trametinib may be re-escalated but should not exceed 2 mg once daily.

- Clarifies: **If a dose reduction of dabrafenib is required, but the toxicity resolves and no additional toxicities are seen after one treatment cycle (4 weeks), the dose of dabrafenib may be re-escalated but should not exceed 150 mg BIDdaily.**

- Section 8.1.1.3.2 Management of Hypertension Table 14: Clarifies foot note 3 as follows: Escalation to previous dose level can be considered if BP remains well-controlled for 4 weeks after restarting. Approval from the Medical Monitor isrequired.
- Section 8.1.2 Pancreatitis: Clarifies the the first paragraph as follows: "Subjects should be closely monitored when re-starting study treatment after an episodeof pancreatitis."
- Section 8.2.1.2 Palmar-plantar Erythrodysesthesia: Adds a new Section as follows: Palmar-plantar erythrodysesthesia (PPE) syndrome: Measures for PPE syndrome should include lifestyle modification (avoidance of hot water, traumatic activity, constrictive footwear, or excessive friction on the skin and the use of thick cotton socks and gloves, and shoes with padded insoles) and symptomatic treatments. Apply moisturizing creams frequently, topical keratolytics (e.g., urea 20 to 40% cream, salicylic acid 6%, tazarotene 0.1% cream, fluorouracil 5% cream), clobetasol propionate 0.05% ointment for erythematous areas, topical lidocaine 2%, and/or systemic pain medication such as nonsteroidal anti-inflammatory drugs, codeine, and pregabalin for pain. Dose modification may also be required.
- Section 8.2.2.1 Cutaneous squamous cell carcinoma (cuSCC):
  - Updates the document reference number for the new Investigator's Brochure (v07).
  - Deletes the following text: Approximately 70 % of events occurred within the first 12 weeks of treatment with a median time to onset of 8 weeks. These should be surgically removed according to institutional practices. Dose modification or interruption of study treatment is not required for cuSCC or KA, however cuSCC should be reported as an SAE. Tumor tissue should also be submitted for further analyses as described in the SPM.
  - Adds the following paragraph: "In a Phase III study, 10% of patients receiving dabrafenib monotherapy developed cuSCC, with a median time to onset of the first occurrence of approximately 8 weeks. In patients who received dabrafenib in combination with trametinib, 3% of patients developed cuSCC with median time to onset of the first occurrence of 32 weeks"
  - Updates the first sentence of the second paragraph as follows: **CuSCC/KA** should be surgically removed according to institutional practices.
- Section 8.2.2.3 Non-Cutaneous Malignancies:
  - Updates the guidance for biopsy of new malignancy as follows: A biopsy of thenew malignancy should be taken, where possible, and submitted **locally** for further analyses with the results provided to the sponsor. Testing of these biopsies may include analysis of genomic alterations, which include but not limited to DNA, RNA and protein analysis of these biopsy specimens, and would analyze the biological pathways known to be associated with, and relevant to, BRAF-mutant tumor activation including BRAF and/or RAS mutation testing.
- Section 8.2.3 Pyrexia:

- Modifies guidelines for monitoring of pyrexia as follows: In subjects experiencing **and after** pyrexia associated with rigors, severe chills, dehydration, hypotension, serum creatinine and other evidence of renal function should be monitoredcarefully.

- 8.2.7 Visual Changes:
  - Updates the management and dose reduction guidelines for visual changes considered to be related to **trametinib** and dabrafenib as follows: **For events of uveitis and related toxicities (e.g. iritis, iridocyclitis), no dose modifications are required as long as effective local therapies can control ocular inflammation. If uveitis does not respond to local ocular therapy, withhold dabrafenib until resolution of ocular inflammation and then restart dabrafenib reduced by one dose level. No dose modification of trametinib is required when taken in combination with dabrafenib.**
- Section 10.2 Prohibited Medications and Non-Drug Therapies:
  - Clarifies the instruction for prohibited medications and non-drug therapies as follows: The use of illicit drugs and the following medications within 28 days or 5 half-lives, whichever is shorter, prior to **start of treatment** will not beallowed:
    - **Antiretroviral drugs**
    - **Herbal remedies (e.g., St. John's wort)**
    - **Drugs that are strong inhibitors or inducers of CYP2C8 or CYP3A4 (Table 27)**
  - Deletes the following text: The use of certain medications and illicit drugs within28 days or 5 half-lives, whichever is shorter, prior to randomization and for the duration of the study will not be allowed.
  - Clarifies the instruction of use of strong inhibitors or inducers of CYP2C8 or CYP3A4: "Strong inhibitors or **inducers** should only be used under special circumstances…"
- Section 10.3 Medication to be used with Caution:
  - Updates the second bullet point as follows: Co-administration of dabrafenib and medicinal products which are affected by the induction of these enzymes such as **hormonal contraceptives**, warfarin, dexamethasone, antiretroviral agents, or Immunosuppressants may result in decreased concentrations and loss of effectiveness.
- 11.2 Definition of Serious Adverse Event:
  - Updates h) Protocol-specific SAEs first bullet point as follows: cuSCC (Section 8.2.2.1), new primary melanomas (Section 8.2.2.2) and non- cutaneous malignancies (Section 8.2.2.3) with the exception of basal cell carcinoma (BCC). BCC should be reported as an AE or SAE based on the discretion of the investigator.
- Section 18.1 Hypotheses and Treatment Comparisons:
  - Revises the third paragraph to read as follows: "The study is powered to detect a high clinically meaningful response rate and is based on the hierarchical model assessment of whether there is sufficiently high probability that < j exceeds Cj.The posterior

probability that the ORR for a given histology is greater than Cj will be computed according to the following comparison:"

- 18.2.1 Sample Size Assumptions:
  - Updates the first paragraph as follows: Each cohort of BRAF V600E mutation-positive tumor type of a given histology will enroll a maximum of 25 subjects **in the primary analysis cohort as defined in Section 5.1**. If all histologies enroll the maximum of 25 subjects, this will result in no more than 225 subjects in **the primary analysis cohort.**
  - Updates the second paragraph to include expansion cohort as follows: Enrollment into specific histology cohorts may be halted early based on results from interim analyses incorporating emerging response data. Response data from a minimum of 5 subjects will be required in a histologic cohort before it may discontinue enrollment for futility and response data from a minimum of 10 subjects will be required before discontinuing a histologic cohort for efficacy. **If a cohort closes early for efficacy at an interim analysis, a histology specific expansion cohort (see Section 5.2) may be opened to allow additional enrolment. At** the final analysis and after the study has been closed, a minimum of 2 subjects will be required in a histologic cohort in order to meet statistical success at the final analysis. See Section 18.3.2 for more details.
- Section 18.3.1 Analysis Population: Updates this particular section as follows:

  As the design for the study calls for repeated interim analyses to evaluate the accumulating efficacy data, it is necessary to shift the focus of the analysis population for efficacy depending upon the time at which the data are being analysed. Table 30 presents the population of interest based on the timing of the analysis for both efficacy and safety; accompanying definitions are provided beneath the table.

Table 30 Definition of Population for specific analysis

		**Analysis Period**	
	**Analysis Intent**	**Interim Analyses**	**Final Analysis**
**Efficacy**	Primary	ITT/Evaluable	BRAF V600E
	Supportive	BRAF V600/Evaluable	ITT Histology-specific cohorts[a]
**Safety**	Primary	ATS	ATS

[a] **Subset of the BRAF V600E population that include histologically similar subjects for cohorts that were stopped for promising efficacy; includes those from the primary analysis cohort and those from the expansion cohort, as defined in Section 5.1 and Section 5.2.**

The BRAF V600E Population is defined as all enrolled subjects regardless of whether or not treatment was administered, who obtain positive verification of the BRAF V600E mutation from a certified central reference laboratory.

The Intent-to-Treat (ITT) population is defined as all enrolled subjects regardless of whether or not treatment was administered.

The All-Treated Subjects (ATS) population is defined as all subjects who receive at least one dose of dabrafenib (GSK2118436) or trametinib (GSK1120212).

The Intent-to-Treat (ITT)/Evaluable population is defined as all ITT subjects in the primary analysis cohort who are also evaluable according to the evaluability defined in the interim analysis RAP.

The BRAF V600E/Evaluable Population is defined as all BRAF V600E subjects in the primary analysis cohort who are also evaluable according to the evaluability defined in the interim analysis RAP.

- 18.3.3 Final Analysis: Updates first paragraph as follows: The final primary analysis for ORR will occur once all subjects **in the primary analysis cohorts have either** discontinue treatment or have received treatment for at least 6 months, whichever comes first.
- Section 18.3.3.1 Safety Analysis: Updates as follows: Safety data will be presented in tabular and/or graphical format and summarized descriptively according to GSK's IDSL standards. The ATS population will be used for the analysis of safety data. In general, safety data will be summarized by histology and **across allcohorts.**

  Complete details of the safety analyses will be provided in the RAP.
- Section 18.3.3.1.3 Other Safety Measures:
  - Deletes the word "MUGA" as planned on amendment 5. It reads as follows: The results of scheduled assessments of vital signs, 12-lead ECG, ECHO and ECOG performance status will be summarized.
- Section 18.3.3.2 Efficacy Analyses:
  - Deletes the following paragraph: The BRAF V600E Population will be used for all final efficacy analyses and used as the supportive population for interim analyses for ORR. The ITT Population will be used for interim analyses for ORR and used as the supportive population for efficacy analyses.
  - Updates the second paragraph as follows: "ORR will be analyzed using an integrated analysis with the hierarchical model as well as analyzed independently **for each histology"**
  - Updates the Response Subcategories in Table 31 to include Marker-Negative CR, Marker-Positive CR, Marker-Negative PR and Marker-Positive PR as response categories for the NSGCT/NGGCT cohort
  - Adds a reference to the RAP in regards to further details on response confirmation criteria for each histological cohort.
  - Modifies the following sentence: "Bayesian inference based on summary statistics from the posterior distributions of each ORR will be reported"

○ Clarifies the definition for Duration of Response as: Duration of response **for** the subset of subjects who **have** a confirmed **response (See Table 31) is defined as** the time from first documented evidence of **response** until the first documented sign of disease progression or death.<br>• 18.4.1 Simulation Description: Updates section to clarify that the expansion cohort will not be taken into consideration in the simulation description.<br>• Section 20 References: Updates the reference to the new version of Dabrafenib (GSK2118436) Monotherapy and Dabrafenib+Trametinib (GSK1120212) Combination Therapy Investigator's Brochure (v7).<br>• Section 21.19 Appendix 19 Protocol Amendment Changes: Updates inconsistency with T&E Table: Laboratory tests (24-hr urine collection; SPEP, UPEP [perform if protein is present in 24-hr urine], CRP, β-2 microglobulin, **serum** free FLC assay, immunoglobulin levels [IgG, IgA, and IgM]) will be performed as indicated in the Time and Event Table.		
2012N145247_07	2016-Jul-19	Amendment No. 7
1. Delete or replace references to GSK or its staff with that of Novartis/Novartis and its authorized agents.<br>2. Make administrative changes to align with Novartis processes andprocedures.		

Sponsor Signatory: **Signature:** **Date:**

[redacted], MD
[redacted]
Novartis Pharmaceuticals
Corporation

SPONSOR INFORMATION PAGE

Clinical Study Identifier: BRF117019

Sponsor Contact Address

Novartis Pharmaceuticals Corporation

In some countries, the clinical trial sponsor may be the local Novartis and its authorized agents. Where applicable, the details of the Sponsor and contact person will be provided to the relevant regulatory authority as part of the clinical trial submission.

Sponsor Serious Adverse Events (SAE) Contact Information:

Please refer to the study procedures manual.

For study conduct questions not related to patient safety, the first line of contact should be with the designated local country company contact. In the event that the designated company contact is not available please contact the Medical Lead.

Medical Lead Contact Information:

[redacted], PhD

[redacted]

Novartis Pharmaceuticals Corporation

One Health Plaza

East Hanover, NJ 07936-1080

Mobile: [redacted]

Email: [redacted]

Clinical Trial Head Contact Information:

[redacted]

Novartis Pharma AG

Postfach (WSJ-103.209.18)

CH-4002 Basel, Switzerland

Phone: [redacted]

Email: [redacted]

If you have any questions regarding the protocol, please contact your local Novartis office.

European Drug Regulatory Authorities Clinical Trials (EudraCT) Number	2013-001705-87
Regulatory Agency Identifying Numbers	US IND #137160

INVESTIGATOR PROTOCOL AGREEMENT PAGE

For protocol number BRF117019

I confirm agreement to conduct the study in compliance with the protocol, as amended by this protocol amendment.

I acknowledge that I am responsible for overall study conduct. I agree to personally conduct or supervise the described study.

I agree to ensure that all associates, colleagues and employees assisting in the conductof the study are informed about their obligations. Mechanisms are in place to ensure that site staff receives the appropriate information throughout the study.

**Investigator Name:**	
**Investigator Address:**	
**Investigator Phone Number:**	
**Investigator Signature**	**Date**

LIST OF ABBREVIATIONS

AA	Anaplastic astrocytoma
ABMT	Autologus bone marrow transplant
ACS	American Cancer Society
ADL	Activities of daily living
AFP	Alpha fetoprotein
AE(s)	Adverse Event(s)
alloSCT	Allogenic stem cell transplantation
ALT	Alanine aminotransferase
ANC	Absolute neutrophil count
AO	Anaplastic oligodendroglioma
APXA	Anaplastic pleomorphic xanthroastrocytoma
ASI	Adenocarcinoma of small intestine
AST	Aspartate aminotransferase
ATC	Anaplastic thyroid cancer
ATP	Adenosine triphosphate
ATS	All-Treated Subjects
autoSCT	Autologous stem cell transplantation
BAL	Bronchioalveolar lavage
BBB	Blood brain barrier
BEP	Bleomycin, etoposide and cisplatin
β-HCG	Beta-Human Chorionic Gonadotropin
BID	Twice daily or two times a day
BLQ	Below the lower limit of quantification
BM	Bone marrow
BP	Blood pressure
BRAFi	BRAF inhibitor
BTC	Biliary tract cancer
BUN	Blood urea nitrogen
°C	Degrees centigrade
C_{τ}	Plasma concentration at end of dosing interval
[redacted]	[redacted]
CBC	Complete blood count
cfDNA	Circulating free DNA
CI	Confidence interval
cm	Centimeter
[redacted]	[redacted]
CNS	Central nervous system
CONSORT	Consolidated Standards of Reporting Trials
CR	Complete response
CRO	Clinical Research Organization
CRP	C-reactive protein
CT	Computed tomography
CTCAE	Common Toxicity Criteria for Adverse Events
cuSCC	Cutaneous squamous cell carcinoma
CYP	Cytochrome
DBP	Diastolic blood pressure
DILI	Drug-induced liver injury

dL	Deciliter
DMC	Data Monitoring Committee
DNA	Deoxyribonucleic acid
DPM	Dirichlet Process Mixtures
DSC	Dynamic susceptibility contrast
ECG	Electrocardiogram
ECOG	Eastern Cooperative Oncology Group
ECHO	Echocardiogram
EDTA	Ethylenediamine tetraacetic acid
eCRF	Electronic case report form
EGFR	Epidermal growth factor receptor
[redacted]	[redacted]
[redacted]	[redacted]
EU	Europe
°F	Degrees Fahrenheit
FDA	Food and Drug Administration
FDG-PET	[^{18}F]fluorodeoxyglucose-positron emission tomography
FET	Fluoroethyltryrosine
FFPE	Formalin-fixed paraffin-embedded
FISH	Fluorescence in situ hybridization
FLAIR	Fluid attenuation inversion recovery
FLC	Free light chain
FSH	Follicle Stimulating Hormone
g	Gram
GBM	Glioblastoma
GCP	Good Clinical Practice
GCPH	Global Clinical Program Head
GCT	Germ cell tumor
GGT	Gamma glutamyl transferase
GI	Gastrointestinal
GIST	Gastrointestinal stromal tumor
GSK	GlaxoSmithKline
HA	Health Authority
HbA1C	Hemoglobin A1C
HBsAg	Hepatitis B surface antigen
HCC	Hepatocellular carcinoma
HCL	Hairy cell leukemia
HDCT	High dose chemotherapy
H/E	Hematoxylin and eosin stain
HMG-CoA	3-hydroxy-3-methylglutaryl-coenzyme A
HPMC	Hydroxypropyl methylcellulose
hr/hrs	Hour(s)
HR	Hazard ratio
HRT	Hormone replacement therapy
[redacted]	[redacted]
IB	Investigator brochure
IBW	Ideal body weight
ICF	Informed consent form

ICH	International Conference on Harmonization
IEC	Independent Ethics Committee
IGCCCG	International Germ Cell Cancer Consensus Group
Ig	Immunoglobulin
IHC	Immunohistochemistry
IMiD	Immunomodulatory drugs
IMWG	International Myeloma Working Group
INR	International normalized ratio
IP	Investigational product
IRB	Institutional Review Board
ITT	Intent-to-treat
IV	Intravenous
IVRS	Interactive voice response system
kg	Kilogram
L	Liter
LBBB	Left bundle branch block
LDH	Lactate dehydrogenase
LDT	Laboratory developed test
LFT	Liver function test
LLN	Lower limit of normal
LVEF	Left ventricular ejection fraction
LSLV	Last subject, last visit
m2	Meter squared
MAO	Mixed anaplastic oligoastrocytoma
MAPK	Mitogen-activated protein kinase
MCH	Mean corpuscular hemoglobin
MCHC	Mean corpuscular hemoglobin concentration
MCV	Mean corpuscular volume
MedDRA	Medical Dictionary for Regulatory Activities
MEK	Mitogen-activated extracellular signal-regulated kinase kinase
MEKi	MEK inhibitor
MET	Methionine
mg	Milligram
MGMT	06-methylguanine-DNA-methyl transferase
mL	Milliliter
MM	Multiple myeloma
mm	Millimeter
mm^3	Cubic millimeter
mmHg	Millimeters of mercury
MR	Minimal/minor response
MRD	Minimal residual disease
MRI	Magnetic resonance imaging
MSDS	Material Safety Data Sheet
msec	Milliseconds
MUGA	Multigated acquisition scan
NA	Not applicable
NCCN	National Comprehensive Cancer Network
NCDB	National Cancer Data Base

NE	Not evaluable
ng	Nanogram
NGGCT	Non-geminomatous germ cell tumor
NHL	Non-Hodgkin’s lymphoma
NSAID(s)	Non-steroidal anti-inflammatory drug(s)
NSCLC	Non-small cell lung cancer
NSGCT	Non-seminomatous germ cell tumor
NYHA	New York Heart Association
O	Optional
ORR	Overall response rate
OS	Overall survival
PBSCT	Peripheral blood stem cell transplant
PCR	Polymerase chain reaction
PCV	Procarbazine, lomustine and vincristine
PD	Pharmacodynamic or progressive disease
PDGFRA	Platelet-derived grow factor receptor alpha
pERK	Phosphorylated extracellular signal related kinase
PET	Positron emission tomography
PFS	Progression-free survival
PGx	Pharmacogenetics
PK	Pharmacokinetics
PPE	Palmar-plantar erythrodysaesthesia
PR	Partial response or partial remission
PS	Performance status
PT	Prothrombin time
PTC	Papillary thyroid cancer
PTT	Partial thromboplastin time
PXA	Pleomorphic xanthoastrocytoma
QTc	Corrected QT interval
QTcB	Bazett-corrected QT interval
RANO	Response Assessment for Neuro-Oncology
RAP	Reporting and Analysis Plan
RBC	Red blood cell
RECIST	Response Evaluation Criteria in Solid Tumors
RNA	Ribonucleic acid
RPED	Retinal pigment epithelial detachment
RR	Response rate
RTK	Receptor tyrosine kinase
RVO	Retinal vein occlusion
SAE	Serious adverse event(s)
SBP	Systolic blood pressure
sCR	Stringent complete response
SD	Stable disease or standard deviation
SEER	Surveillance, Epideminology and End Results
SmPC	Summary of Product Characteristics
SPEP	Serum Protein Electrophoresis
SPF	Skin protection factor
SPM	Study Procedures Manual

SUV	Standard uptake value
T/B	Tumor background ratio
TKI	Tyrosine kinase inhibitor
TMZ	Temozolomide
TSH	Thyroid stimulating hormone
ULN	Upper limit of normal
UGT	UDP glucuronosyl transferase
UPEP	Urine Proteine Electrophoresis
US	United States
VEGF	Vascular endothelial growth factor
VGPR	Very good partial response
VHP	Voluntary harmonization procedure
VHP-SA	Substantial amendment of a Voluntary Harmonization Procedure
vs	Versus
WBC	White blood cells
WHO	World Health Organization

1 INTRODUCTION

1.1 Background

The RAS/RAF/MEK/ERK pathway, also known as the mitogen-activated protein kinase (MAPK) pathway, is a critical signal transduction cascade implicated in normal growth and the uncontrolled proliferation of many human cancers. Under normal physiological conditions, signal transduction through the MAPK pathway is tightly regulated through multiple negative feedback mechanisms [Niault, 2010]. However, this pathway can be constitutively activated by alterations in specific proteins, including BRAF, which phosphorylates mitogen-activated extracellular signal-regulated kinase kinase (MEK) on two regulatory serine residues. BRAF mutations have been identified at a high frequency in specific cancers [Davies, 2002]. Approximately 90% of all identified BRAF mutations that occur in human cancers are T1799A transversions (exon 15), which result in a V600E amino acid substitution [Wellbrock, 2004]. In melanoma, the BRAF V600E mutation has been identified in about 50% of subjects as a key driver mutation of tumorigenesis, and BRAF inhibitors (BRAFi), such as vemurafenib and dabrafenib, have demonstrated substantial efficacy in this setting [Chapman, 2011a].

While the introduction of BRAFi represent a significant advance in the treatment of BRAF V600 mutation-positive metastatic melanoma [Chapman, 2011a], limitations of this novel therapy have already been identified. As has been the pattern with other highly selective small molecule kinase inhibitors (e.g., imatinib, erlotinib and gefitinib) the rapid onset of drug resistance restricts the efficacy. Understanding the specific mechanisms of resistance to BRAFi is critical for the development of more effective strategies to inhibit the MAPK-pathway in order to delay or prevent the onset of resistance in BRAF-mutant melanoma.

[redacted]

[redacted]

1.2 Dabrafenib (GSK2118436)

[redacted]

Refer to the dabrafenib monotherapy and dabrafenib+trametinib combination therapy Investigator's Brochure (IB) [GlaxoSmithKline Document Number CM2010/00010/05] for a complete summary of currently available preclinical, safety, pharmacokinetic (PK), and clinical data.

1.3 Trametinib (GSK1120212)

Trametinib (GSK1120212) is a reversible, highly selective, allosteric inhibitor of MEK1 and MEK2 and kinase activity [Gilmartin, 2011]. Trametinib has been approved for the treatment of BRAF-mutant unresectable or metastatic melanoma in several countries, including the US, EU, Kazakhstan and Australia. Trametinib has been studied in Phase II trials in pancreatic cancer and RAS-mutant NSCLC as well as RAS-mutant acute myeloid leukemia.

[redacted]

Trametinib in combination with dabrafenib has been approved in the US, EU, Chile, and Australia for the treatment of patients with unresectable or metastatic melanomawith BRAF V600E or V600K mutation.

Refer to the dabrafenib monotherapy and dabrafenib+trametinib IB for a complete summary of the currently available chemistry, nonclinical, and clinical data to support the use of the combination in subjects with BRAF V600 mutation-positive solid tumors [GlaxoSmithKline Document Number CM2010/00010/05]. A separate IB is also available summarizing the preclinical, safety, PK, and clinical data for trametinib monotherapy [GlaxoSmithKline Document Number HM2009/00151/05].

1.4 Risk Assessment for Dabrafenib + Trametinib Combination Therapy

[redacted]

[redacted]

[redacted]

[redacted]

[redacted]

[redacted]

[redacted]

[redacted]

[redacted]

[redacted]

[redacted]

[redacted]

[redacted]

[redacted]

[redacted]

[redacted]

[redacted]

[redacted]

[redacted]

[redacted]

[redacted]

[redacted]

1.4.1 Benefit Assessment

This is not the initial clinical experience with dabrafenib in combination with trametinib, therefore, the benefit:risk has been established and should be favorable if the anticipated level of efficacy is demonstrated.

Other considerations for subjects who participate in this study may include:

- Contribution to the ongoing process of developing a new treatment
- Medical evaluations/assessments associated with study procedures (e.g., physical examinations, electrocardiograms [ECGs], clinical laboratory tests, etc.)

1.4.2 Overall Benefit: Risk Conclusion

Taking into account the measures to be taken to minimize the risk to subjects participating in this study, the potential risks identified in association with dabrafenib + trametinib combination therapy are justified by the anticipated benefits that may be afforded to subjects.

1.5 Histology-Specific Background

1.5.1 Cohort 1: Anaplastic Thyroid Cancer (ATC)

The American Cancer Society (ACS) estimates approximately 56,460 new cases of thyroid cancer in the US alone for 2012 [ACS, 2012]. Only 0.5% to 1.7% of all thyroid cancers are diagnosed as ATC [Papay, 2009; Pitt, 2010]. While differentiated thyroid cancers are typically curable, ATC is one of the most aggressive and fatal forms of thyroid cancer [Caronia, 2011]. Patients with ATC have the worst survival; a 1-year survival rate of approximately 10% [Goutsouliak, 2005]. Anaplastic thyroid carcinoma (ATC) is more commonly seen in females than males and is typically diagnosed in the elderly (median age: 70 years) [Papay, 2009; Pitt, 2010].

Patients with ATC typically present with a rapidly growing neck mass and commonly with complaints of sore throat, hoarseness, coughing, dysphagia, dyspnea, and neck pain. Airway and esophageal compromise is a major concern; therefore, obtaining local control of the disease in the neck is critical and essential to the quality of life of the patient in order to prevent asphyxiation and maintain oral nutrition with the hope of improving overall survival [Haigh, 2001; Papay, 2009; Pitt, 2010]. Methods utilized to improve local control in ATC include surgery and/or radiation therapy (e.g., radioactive iodine). The median survival from these methods is <6 months.

Surgery is limited as ATC tumors are rarely resectable at time of diagnosis due to ill- defined borders and/or attachment to surrounding vital structures in the neck [Haigh, 2001]. If the disease is confined to a local area, a total thyroidectomy is warranted to reduce symptoms due to the tumor mass; a tracheostomy is frequently required [GlaxoSmithKline Document Number HM2009/00151/05; Goldman, 1980]. There is some evidence suggesting that surgery followed by post-operative radiation therapy and chemotherapy results in improved outcome than radiotherapy alone [Haigh, 2001].

Patients who are not candidates for surgery or whose tumor cannot be surgically excised may receive external beam radiation therapy. However, treating ATC with high dose radiation is limited by the presence of the spinal cord and lungs due to their close proximity to the tumor target. Combining radiation therapy with chemotherapy has been shown to prolong survival in patients who have also completed surgical resection [Posner, 2000].

Single-agent doxorubicin is typically considered the "standard" chemotherapy agent for treating ATC in advanced disease with a reported response rate of approximately 22% [Nagaiah, 2011]. It appears that the combination of doxorubicin plus cisplatin is more active than doxorubicin alone and has been reported to result in a greater number of responses (3 complete responses [CRs] and 3 partial responses [PRs]) being reported [Shimaoka, 1985]. Ain et al reported a 53% response rate (using a non-conventional definition of response rate) with one CR and nine PRs reported in 19 evaluable subjects treated with a 96-hr continuous infusion of paclitaxel [Ain, 2000]. However, the response rate did not translate to prolongation of median survival which was only 25 weeks. Chemotherapy combined with radiation therapy in patients following complete resection has been evaluated and may provide prolonged survival [Haigh, 2001; DeCrevoisier, 2004]. None of the approaches to date provide sustainable duration of response.

Molecular signaling mechanisms such as activation of the RTK-RAS-RAF-MAPK pathway, p53 mutation, epidermal growth factor receptor (EGFR) expression and vascular epithelial growth factor (VEGF) secretion, are thought to be significant factors in the progression of ATC [Kojic, 2011]. Mutations of the RAS oncogene and p53, a well-known tumor-suppressor gene, are frequently seen in molecular studies of ATC [Papay, 2009; Pitt, 2010]. Recently alternations in the BRAF oncogene have been seen and may be involved in the progression of BRAF-mutated papillary thyroid cancer (PTC) to ATC. Specifically, BRAF V600E mutation has been found in both PTC and papillary- derived ATC in which it initiates follicular cell transformation [Caronia, 2011; Kojic, 2011]. The average frequency of BRAF mutations in ATC is 24% and tends to have more aggressive phenotypes that often become resistant to traditional therapies. BRAF mutations may lead to changes in the sodium-iodide symporter limiting adjunct radioactive iodine treatment by decreasing iodine uptake [Kojic, 2011].

Pre-clinical studies in human ATC cell lines have shown the importance of therapeutic agents that selectively target BRAF kinase, significantly inhibiting tumor growth [Caronia, 2011; Kojic, 2011]. Vemurafenib, a BRAFi, has shown decreased cell proliferation and aggressiveness and markedly inhibit tumor growth in animal models using human ATC cell lines [Nucera, 2011]. Rosove et al reported a significant response to vemurafenib in a patient with BRAF-mutated ATC with radiological assessment confirming nearly complete clearing of metastatic disease after 38 days [Rosove 2013]. [redacted]

1.5.2 Cohort 2: Biliary Tract Cancer (BTC)

Biliary tract cancers (BTC) include a spectrum of invasive adenocarcinomas encompassing both cholangiocarcinoma, which refers to cancers arising in the intrahepatic, peri-hilar, or

distal biliary tree, and gall bladder carcinoma. Gallbladder carcinoma is an uncommon cancer in developed Western countries, afflicts approximately 12,000 people in the US annually, with a recently appreciated increasing incidence of intrahepatic cholangiocarcinoma [Hezel, 2010]. Cholangiocarcinoma is an adenocarcinoma of the bile duct with a poor prognosis given the invasiveness of the disease and its resistance to conventional chemotherapy. About 5 to 10% of all primary liver cancers are cholangiocarcinoma. Only 10% of patients present with early-stage disease and are considered candidates for surgical resection, which offers the only chance for cure. The prognosis is poor for the majority of patients with locally advanced or metastatic BTC, with a median survival of <1 year. No standard post-operative treatment has been established. Gemcitabine with or without cisplatin is being studied for treatment in the adjuvant setting for gallbladder carcinoma, intra-hepatic and extra- hepatic bile duct. Results of a study by Valle et al concluded that gemcitabine plus cisplatin provided a significant survival advantage over gemcitabine alone. The median overall survival (OS) is 11.7 months with gemcitabine plus cisplatin vs. 8.1 months with gemcitabine monotherapy [Valle, 2010].

Tannapfel et al conducted the first study of mutational status of BRAF in liver tumors where the frequency of mutations of BRAF and KRAS oncogenes in hepatocellular carcinoma (HCC) and cholangiocarcinoma was evaluated [Tannapfel, 2003]. No BRAF mutations were detected in HCC; however, specific BRAF mutations were noted in approximately 21% of the cholangiocarcinoma cases. Saetta et al reported in the second year that the BRAF mutations were observed in 7 of 21 (33%) gallbladder carcinomas examined [Saetta, 2004]. In the Tannapfel et al study, mutations of KRAS (45% of cases) together with BRAF mutations (22%), the RAS/RAF/MEK/ERK/MAPK pathway had a high frequency of alteration in cholangiocarcinoma. The results of such studies support the significance of the role disruption of the MAPK cascade may have in the development of gallbladder cancer. The alterations in the RAS/RAF/MEK/ERK/MAPK pathway may also play a significant role in new treatment strategies for BTC and support the use of BRAFi and/or MEKi.

1.5.3 Cohort 3: Gastrointestinal Stromal Tumors (GIST)

Gastrointestinal stromal tumors (GISTs) are the most common mesenchymal tumors in the gastrointestinal (GI) tract, mainly within the stomach and small intestine. Most GIST cells are positive for c-kit. Malignant GIST cells are expected to harbor a mutation of c-kit that results in the activation of c-kit leading to cell division and tumor growth. Tyrosine receptor inhibitors (TKIs) such as imatinib selectively inhibit c-kit and therefore, are indicated for the treatment of GIST. Tyrosine kinase inhibitors (TKIs), imatinib and sunitinib, are currently the standard of care in treating metastatic or unresectable GIST. Imatinib is approved for first-line therapy of metastatic GIST with disease control achieved in approximately 85% of treated patients; median progression- free survival (PFS) is 20 to 24 months [Italiano, 2011]. Sunitinib is the only approved drug for imatinib-resistant GIST. Clinical benefit was reported in approximately 50% of the patients treated, with a PFS of 7 to 10 months [Demetri, 2006]. Mutational status of KIT and platelet-derived growth factor receptor alpha (PDGFRA) and albumin levels have been significantly associated with PFS [Italiano, 2011]. Low levels of albumin may be indicative of the nutritional status but also the presence of a systemic inflammatory response.

Following treatment with imatinib and/or sunitinib, patients with advanced GIST often develop drug-resistant tumors which drive the need for alternative treatment. Imatinib-resistance is observed in 10 to 15% of patients following initial treatment with secondary

resistance observed in almost all patients who initially have a clinical response from imatinib [Italiano, 2011]. Data has suggested that the resistance to imatinib in GISTs may be due to the activation of the MAPK pathway and may explain why some patients with GIST who are treated with imatinib fail to respond to treatment despite having KIT- sensitive mutations [Miranda, 2012]. While most of the GISTs carry mutations in KIT (80%) or the PDGFRA gene (8%), 3 to 13% of adult GIST cases have a wild-type genotype [Hostein, 2010; Agaram, 2008; Caram, 2011]. The MAPK pathway (RAS/RAF/MEK/ERK) is one that is activated by KIT and PDGFRA. The BRAF protein when activated transmits signaling through the MAPK pathway as well but only further downstream without the stimulation of KIT or PDGFRA. BRAF mutations have been detected in a limited number of patients with imatinib-naïve or –resistant GISTs, occurring preferentially in small intestinal and gastric high risk GISTs that lack the KIT and PDGFRA mutations [Agaram, 2008; Hostein, 2010]. Drugs that inhibit KIT and PDGFRA would be ineffective in treating GISTs with BRAF mutations. Patients with wild-type GIST have poor responses to TKIs and poor overall survival [Heinrich, 2008]. Therefore, these patients could benefit from having select BRAF inhibitors as an alternative treatment to imatinib and other tyrosine kinase inhibitors.

1.5.4 Cohort 4: WHO Grade 1 and 2 Glioma

According to the ACS, there were an estimated 22,910 new cases of malignant brain and other central nervous system (CNS) tumors in 2012, leading to over 13,700 deaths in the US alone [ACS, 2012]. Worldwide the number of new cases of brain and other CNS tumors diagnosed in 2008 was approximately 238,000 with an estimated 175,000 deaths [Ferlay, 2011]. Gliomas account for 32% of all the primary brain tumors and are historically classified by the World Health Organization (WHO) as either Grade 1 2, 3 or 4.

The most common pediatric brain tumor, pilocytic astrocytoma, occurs in 20% of brain tumors in patients under 20 years of age and is classified as a WHO Grade 1 glioma [Sadighi, 2013]. While the 10-year OS rate is high at 96%, recurrent and progressive disease occurs in 20% of patients [Sadighi, 2013]. In these patients, total resection remains the treatment of choice. For inoperable tumors, radiotherapy or chemotherapy is the first line treatment [Sadighi, 2013]. Recent findings have indicated that certain types of low grade and pediatric brain tumors, such as pilocytic astrocytoma, may have a higher rate of BRAF alterations [Schindler, 2011]. BRAF V600E mutations are also found in WHO Grade 1 gliomas including 18% of WHO Grade 1 gangliogliomas and 9% of pilocytic astrocytoma (especially extra-cerebellar pilocytic astrocytoma) [Dias-Santagata, 2011; Schindler, 2011]. Such aberrations are known to activate the MAPK pathway which provides the platform by which novel molecular targeted therapy can develop. Agents such as MEKi can serve as novel treatment options for patients with progressive pilocytic astrocytomas.

Currently there are Phase II studies with MEKi such as selumetinib underway. Preliminary results have indicated a favorable pharmacologic profile. However, drug- resistance when administered as a single agent has been seen in some tumor models [Sadighi, 2013]. One explanation for this resistance may be due to the activation of an alternative pathway such as MEK-1 mutations. Therefore, it may be more effective to offer a combination therapy than single agent to decrease the potential for resistance.

WHO Grade 2 tumors such as diffuse astrocytoma, oligodendroglioma and oligoastrocytoma are usually non-enhancing (>90%) and typically affect patients in their third and fourth decade of life. Although WHO Grade 2 tumors have a less aggressive course than WHO Grade 3 or 4 gliomas that are considered to be high grade, the outcome of these tumors is

ultimately fatal in most patients. BRAF V600E mutations have been identified in approximately 60% of WHO Grade 2 pleomorphic xanthoastrocytoma (PXA), (a similar mutation rate as seen in melanoma).

For adult WHO Grade 2 gliomas, treatment guidelines have been established. Surgical resection represents the first treatment option. For patients with unfavorable prognostic factors such as older age, incomplete or no resection and existing neurological symptoms, adjuvant radiotherapy is indicated. Chemotherapy is an option for patients with recurrence after surgery and radiation therapy and is also an option as initial treatment for patients with large residual tumors after surgery or unresectable tumors, especially when 1p/19q loss is present. Treatment with procarbazine, lomustine and vincristine (PCV) or temozolomide (TMZ) yield similar response rate and duration of response with a toxicity profile favoring TMZ. The prognosis of patients who have recurrent or progressive disease following radiotherapy and chemotherapy is usually poor.

The Response Assessment for Neuro-Oncology (RANO) Working Group proposed a response criteria for non-enhancing low grade tumors to be adapted in clinical trials when response rate or PFS is used as the primary endpoint. In addition to CR and PR categories in WHO Grade 3 or 4 glioma Modified RANO response criteria, minor response (MR) is proposed as an additional response category of the WHO Grade 1 or 2 Modified RANO response criteria. In contrast to WHO Grade 3 or 4 glioma, MR is assumed to be particular important for WHO Grade 1 or 2 gliomas for it is often the best achievable response category [van den Bent, 2011].

[redacted]

[redacted]

[redacted]

1.5.5 Cohort 5: WHO Grade 3 and 4 Glioma

Gliomas classified by the WHO as either Grade 3 or 4 are often referred to as high-grade. The incidence of adult high grade gliomas is 2 to 4 per 100,000 patients [Perry, 2004]. WHO Grade 3 or 4 gliomas are locally invasive and associated with a poor prognosis and include Grade 3 anaplastic astrocytoma (AA), anaplastic oligodendroglioma (AO), anaplastic mixed anaplastic oligoastrocytoma (MAO), anaplastic pleomorphic xanthroastrocytomas (APXA) and Grade 4 glioblastoma (GBM) and accounts for approximately 60 to 75% of all gliomas [Hadziahmetovic, 2011]. Metastasis outside of the CNS has been reported butis extremely

infrequent until multiple local relapses have occurred. Biologic markers, such as O6-methylguanine-deoxyribonucleic acid (DNA)-methyltransferase (MGMT) and mutation status, may be useful predictors of outcome in patients with WHO Grade 3 or 4 gliomas. WHO Grade 3 anaplastic gangiogliomas and WHO Grade 4 GBM have also been reported to have BRAF V600E mutations [Dias-Santagata, 2011; Schindler, 2011]. pERK was detected in all tumors independent of the BRAF mutation status [Dias-Santagata,2011].

Historically, the gold standard for treating WHO Grade 3 or 4 gliomas has been surgery followed by radiotherapy resulting in an estimated 5-year OS of 2 to 3 % [Hadziahmetovic, 2011]. Demonstrating efficacy with the addition of chemotherapy to the treatment standard has been more difficult. The treatment of WHO Grade 3 or 4 gliomas with carmustine or a combination regimen of PCV has recently shifted to the addition of concurrent or adjuvant TMZ with radiation. Until recently the treatment of all WHO Grade 3 or 4 gliomas has been much the same; however, ongoing clinical trials are segmenting the Grade 4 gliomas so the treatment modalities can focus on AA, AO and anaplastic MOA tumors [Hadziahmetovic, 2011]. The European Organization for Research and Treatment of Cancer (EORTC) conducted a randomized trial in AA, AO and anaplastic MOA tumors in adjuvant radiotherapy with or without PCV chemotherapy. The addition of PCV improved the PFS (1.9 vs. 1.1 years) but not OS (3.4 vs. 2.6 years) [Hadziahmetovic, 2011]. Seeking a more favorable regimen, studies with TMZ have reported objective response rates of 58% with 32% CRs, rate of progression of only 10% with TMZ administered prior to radiation.

Considerable interest in determining the frequency and distribution of BRAF V600E mutations in central and peripheral nervous system tumors has been generated recently to help identify the tumor entities that may potentially benefit from BRAF V600E targeted therapy. In study BRF113929, which was to evaluate the safety and efficacy of dabrafenib in subjects with BRAF V600E/K mutation-positive melanoma metastatic to the brain, response rates of 31% and 39% were observed in 74 subjects without prior brain treatment and 53 subjects with treatment. This suggests that the study treatment can have activity in brain tumors.

1.5.6 Cohort 6: Non-Seminomatous/Non-Germinomatous Germ Cell Tumors (NSGCT/NGGCT)

The worldwide incidence of germ cell tumors (GCT) has more than doubled in the past 40 years. Germ cell tumors (GCTs) are relatively uncommon and account for only 2% of all human malignancies. Non-seminomatous germ cell tumors (NSGCT) are malignant extragonadal germ cell tumors that generally form in the pineal gland of the brain, the mediastinum or the abdomen from cells meant to form sperm in the testicles or eggs in the ovaries and account for 48% of all GCTs. These tumors are managed in the same way as testicular GCTs. In the US, approximately 8,590 new cases of testicular cancer will be diagnosed in 2012 [Siegel, 2012]. Testicular cancer is the most common solid tumor in males between the ages of 15 and 35 years with 95% of the testicular cancers being testicular GCTs (evenly reported as seminoma and non-seminoma) [Le Gal, 2005; Mannuel, 2011].

Non-seminomatous GCTs (NSGCTs) tend to be more clinically aggressive and grow quicker than the seminoma-type of GCT. If untreated, NSGCTs can spread to the lungs, liver, lymph nodes, or bones. Common risk factors include being male, under the age of 20 and having Klinefelter syndrome [Nichols, 1987]. Chest pain and difficulty breathing are early signs of NSGCT. Other symptoms include: cough, fever, headache, and change in bowel habits, fatigue, difficulty walking and visual problems (difficulty seeing or moving eyes). Testicular

GCTs present with a painless, solid testicular mass and often discomfort or swelling; persistent conditions warrant further evaluation.

Serum tumor markers alpha-fetoprotein (AFP), lactate dehydrogenase (LDH) and beta-human chorionic gonadotropin (β-HCG) are used in determining the diagnosis and prognosis as well as assessing treatment in GCTs. These tumor markers are especially useful in monitoring NSGCT at all stages. Tumor markers AFP and β-HCG are more specific than LDH; AFP may be seen at any stage of disease. An elevation in AFP generally indicates the presence of an undetected focus of non-seminoma [Nazeer, 1998; Weissbach, 1997]. An elevated β-HCG should be interpreted with caution if the use of marijuana is suspected or the development of hypogonadism as benign elevation in β-HCG may result.

In 1997, the International Germ Cell Cancer Consensus Group (IGCCCG) developed a classification system based upon prognostic factors such as extent of disease and serum tumor markers. Post-orchiectomy markers are used to determine the IGCCCG risk classification [IGCCCG, 1997]. This classification categorizes patients into risk groups designated as good, intermediate or poor.

Following diagnosis, NSGCT patients with persistent elevation of serum tumor markers are typically treated with a cisplatin-based chemotherapy regimen consisting of 4 cycles of bleomycin, etoposide and cisplatin (BEP) considered the standard first-line therapy [Williams, 1987; Hinton, 2003]. Although the tumor volume tends to be very large, initial debulking surgery is rarely considered useful. Long-term disease-free survival may be achieved following post-chemotherapy surgery to resect residual disease following initial chemotherapy treatment [Schneider, 2004]. Patients with NSGCT who relapse following first-line chemotherapy generally have poor responses to salvage chemotherapy or bone marrow (BM) transplantation and are therefore candidates for new therapies [Saxman, 1994; Beyer, 1996]. Gemcitabine containing palliative chemotherapy provides 30 to 40% ORR with median survival only between 5 to 9 months [Bokemeyer, 2008; Einhorn, 2007].

The median OS is 6 to 9 months following treatment with best supportive care [Kollmannsberger, 2004; Einhorn, 2007]. The cure rate for intermediate risk (Stage IIIB) NSGCT is approximately 70% following 4 cycles of BEP [DeWit, 1998; Frohlich, 1998]. In the poor risk (Stage IIIC) patients, between 20 and 30% of all patients with metastatic NSGCT remain not cured following cisplatin-based chemotherapy. Less than 50% of patients experience a durable CR to BEP, leaving them with limited treatment options other than investigational therapy [Jones, 2003].

Of the patients with NSGCT who develop refractory disease, the BRAF V600E mutation is detected in 2 to 3% [Mayer, 2010; Honecker, 2009]. The BRAF V600E mutation is not detected in seminomatous GCT. The tumors with the positive mutation tend to be extragonadal, especially with an origin in the mediastinum. Furthermore, the BRAF V600E mutation has only been reported in adult NSGCT patients, excluding pediatric or adolescent patients [Masque-Soler, 2012]. As seen in melanoma, those patients with NSGCT and the BRAF positive-mutation show a trend to longer PFS.

1.5.7 Cohort 7: Adenocarcinoma of the Small Intestine (ASI)including Periampullary and Ampullary

Adenocarcinoma of the small intestine (ASI) is a rare cancer; less than 2.3% of GI cancers arise in the small intestine. According to the ACS, 6230 new cases and 1110 deaths due to small intestine cancers were estimated in 2009 in the US. Adenocarcinoma of the small

intestine (ASI) has historically been the most commonly diagnosed histologic subtype of small bowel tumors, but has recently been surpassed by carcinoid tumors according to the National Cancer Data Base (NCDB). There are approximately 2000 to 3000 new patients per year in the US [Bilimoria, 2009; Jemal, 2009]. Adenocarcinoma of the small intestine (ASI) typically includes those arising from the ampulla of Vater and the periampullary region; however, these were not included in the NCDB in the category of small bowel tumors. Tumors of amulla of Vater include tumors arising in the ampulla (intra-ampullary type), tumors arising in the periampullary region of the duodenum (periampullary type), or tumors involving both the intra- ampullary and periampullary region of the duodenum (mixed periampullary and intra- ampullary type). The rare nature of ASI has led to a poor understanding of disease pathogenesis, delay in diagnosis, and a paucity of information regarding treatment. Thirty-two percent of patients with ASI present with advanced disease [Overman, 2012]. The treatment of choice for ASI is curative surgical resection. However, no standard protocol has been defined for use when the disease is unresectable or relapsed. The majority of chemotherapy experience has been with 5-fluororuacil, alone or in combination with some of the newer chemotherapeutic agents such as irinotecan, platinum compounds and gemcitabine. Response rates with chemotherapy agents have ranged from 6% and 41%. The median OS was between 9 to 18.6 months [Speranzaa, 2010]. Mutations of BRAF V600E were detected in 4 out of 37 subjects with ASI by Warth et al [Warth, 2011] and in one periampullary adenocarcinoma by Schonleben et al [Schonleben,2009].

1.5.8 Cohort 8: Hairy Cell Leukemia (HCL)

Hairy cell leukemia (HCL) is a rare B-cell lymphoproliferative disease accounting for 2 to 3% of all leukemias in adults in Western world [Riccioni, 2007]. The incidence in the US was 0.33 per 100,000 in 2011 [Morton, 2006]. Virtually all patients with HCL carry the BRAF V600E mutation, the HCL variant, which typically presents with leukocytosis instead of leukopenia, and absence of CD25, TRAP or Annexin A1, is uniformly wild-type for BRAF [Xi, 2012; Tiacci, 2011; Matutes, 2003]. Sequencing the gene in 47 HCL patients disclosed BRAF V600E mutations in nearly all patients. pERK was also found in BM biopsies from these patients indicating that the BRAF mutation was activating. Incubation of primary hairy cells with a BRAFi led to reversal of ERK and MEK phosphorylation [Tiacci, 2011]. Recently, Dietrich et al reported that a refractory HCL patient had been successfully treated with a BRAFi validating that BRAF mutation is an appropriate therapeutic target in HCL [Dietrich, 2012].

Patients are categorized as having untreated HCL or progressive HCL for the purpose of determining treatment. Treatment is based on symptomatic cytopenias, massive splenomegaly or the presence of other complications. Ten percent of all patients will not require any treatment [Saven, 2001]. Untreated HCL is characterized by splenomegaly, leukopenia and/or pancytopenia, and BM infiltration by an atypical cell with prominent cytoplasmic projections (i.e., hairy cells). Bone marrow (BM) biopsy is required for diagnosis and to determine the degree of hairy cell infiltration. Asymptomatic disease is often not treated if blood counts remain in an acceptable range [Grever, 2011]. Replacement of the BM by hairy cells with pancytopenia refractory to treatment characterizes progressive HCL. The treatment of choice for patients with advanced HCL is cladribine (2-chlorodeoxyadenosine, 2-Cda) or pentostatin (2-deoxycoformycin); both with comparable response rates [Gidron, 2006; Grever, 2011]. The survival rate with systemic therapy is estimated to be >85% at 5 years following the initiation of systemic therapy [Estey, 1992; Frassoldati, 1994].

Consolidation or maintenance therapy with additional purine analog in preventing relapse or disease progression following purine analog treatment has not been formally evaluated, but the clinical impression is that repeated courses of purine analog have increasing cumulative toxicity with decreasing benefit, and should not be added unless patients require retreatment due to cytopenias. Moreover, cytopenias due to disease requiring retreatment must be differentiated from cytopenias due to toxicity, and it is very rare for retreatment within 6 months after prior purine analog therapy to be advisable. Most patients have relapsed disease by 16 years [Else, 2009]. There is no plateau on the disease-free survival curves to suggest that purine analogs can cure HCL [Goodman, 2003; Else, 2009]. At a median of 16 years after treatment, the Scripps population of 358 subjects contained 19 who were still being followed in continuous complete remission, nine of whom were still without evidence of minimal residual disease (MRD) [Sigal, 2010]. Thus, it is not clear if purine analogs have curative potential, but clearly they fail in most patients who live long enough. Multiple immune defects present in HCL appear to have a role in the development of second cancers. Skin cancers were found to be the most numerous before the availability of nucleoside analogs for the treatment of HCL [Dasanu, 2010]. Treatment with either nucleoside analogs can result in profound CD4 suppression that may last up to 4 years [Seymour, 1994; Seymour, 1997], and this may increase the risk of secondary malignancies [Goodman,2003; Hisada 2007]. A study of HCL survivors from the Surveillance Epidemiology and End Results (SEER) database confirmed the increased risk of second cancers, especially for Hodgkin lymphoma and non-Hodgkin's lymphoma (NHL), among those treated with cladribine but not with pentostatin [Morton, 2006; Hisada, 2007; Dasanu, 2010]. For those patients who experience severe thrombocytopenia, splenectomy may be considered, particularly if all other options have failed. However, while splenectomy derives a palliative, usually temporary benefit in blood counts, few patients have sustained remissions [Golomb, 1983], and disease may continue to worsen in the blood, lymph nodes, BM and visceral sites. Virtually all patients progress and the median OS in large retrospective studies were less than 6 years [Jansen, 1981; Chrobak, 1993; Haberman, 2006; Haberman, 2011]. Interferon-alpha is largely a drug of historical significance [Haberman, 2006; Haberman, 2011]. In fact, the response rate was 0% in subjects not responding to pentostatin who crossed over to interferon [Grever, 1995].

Patients who relapse after the first course of cladribine or pentostatin treatment often respond, but with less chance of CR, to repeated treatment with purine analog, with no benefit if a different purine analog is used [Saven, 1998; Else, 2009]. Rituximab, although not approved for HCL by the US Food and Drug Administatrion (FDA), remains a common treatment but most patients, particularly those with prior purine analog treatment and significant disease burden, do not respond and need alternative treatment. In a study of 24 subjects with prior therapy with purine analogs and need for retreatment due to cytopenias, the overall response rate (ORR) of rituximab was only 26% with only 13% CRs [Nieva, 2003]. Rituximab with cladribine or pentostatin may be an alternative, but the increased efficacy of this combination must be weighed against its toxicity, and it is not considered standard at this time [Else, 2007; Else, 2009; Ravandi, 2006]. In clinical practice, pentostatin is usually not considered for patients already resistant to cladribine, and vice versa. Thus, there is an unmet medical need for a non-chemotherapy option in HCL resistant to purine analogs.

1.5.9 Cohort 9: Multiple Myeloma (MM)

Multiple myeloma (MM) is a systemic malignancy of plasma cells in the BM that secretes all or part of a monoclonal antibody. It is the second most common hematologic malignancy,

accounting for 10% of all hematologic cancers and 2% of annual cancer- related deaths [Anderson, 2011]. The incidence of multiple myeloma in the US in 2008 was 5.579 per 100,000 [Ferlay, 2011] According to the ACS, it was expected that almost 21,700 new cases of MM and 10,700 MM related deaths would occur during 2012 [ACS, 2012].

Chapman et al and Walker et al genotyped MM patients and detected the BRAF V600E mutation in 2.4% and 4%, respectively [Chapman, 2011b; Walker, 2012]. The finding of BRAF mutations in patients with MM has important clinical implications because such patients may benefit from treatment with a BRAFi. These results also support the observation that inhibitors acting downstream of BRAF (i.e., MEK) may have activity in MM [Kim, 2010].

Multiple myeloma (MM) is further classified on the basis of the patient's characteristics. Patients who are younger than 65 years of age and without other severe co-morbidities are considered eligible for allogenic stem cell transplantation (alloSCT). Patients older than 65 years or with serious co-morbidities are usually not considered SCT candidates and a gentler approach is needed. Although MM is not curable, it is treatable. The median survival with chemotherapy is 24 to 30 months with a 10-year survival rate of 3%. With newer therapies of immunomodulatory drugs (IMiD: thalidomide, lenalidomide), proteosome inhibitor (bortezomib) and stem cell transplantation, the median OS has been prolonged 7 to 8 years [Kumar, 2008; Anderson, 2011].

In the bortezomib registration study of over 600 subjects, the response rate to bortezomib and dexamethasone for subjects with MM relapsing after at least one systemic therapy not containing bortezomib was 38% [Richardson, 2005]. The PFS for the bortezomib arm in this study was 6.2 months (median follow-up of 8.3 months). A subset analysis showed that the ORR and survival was higher for the less heavily pre-treated subjects. Amongst the IMids, both thalidomide and lenalidomide have been extensively investigated in the induction/consolidation/maintenance settings and both agents have been approved by the US FDA.

Despite these agents and along with other agents such as carfilzomib and pomalidomide, MM continues to remain incurable. In particular, patients resistant to novel agents represent a challenging group. Kumar et al. conducted an analysis in subjects who relapsed or were refractory to one of the IMiDs as well as bortezomib that determined the median event-free survival to be 5 months and the median OS was 8 months [Kumar, 2010]. Therefore, these patients require novel strategies.

2 RATIONALE

2.1 Rationale for the Combination of Dabrafenib and Trametinib

Pre-clinical studies have demonstrated that the combination of dabrafenib and trametinib is synergistic and/or enhances cell growth inhibition in majority of the BRAF V600E mutant melanoma, colon, thyroid, and lung cancer cells. The combination delayed dabrafenib resistance and reduced hyperproliferation skin adverse effect in pre-clinical models. The scientific rationale suggesting MEK-mediated re-activation of the MAPK pathway as a prominent mechanism of resistance to BRAFi single-agent therapy supports the combination study of trametinib with BRAFi in cancer [Alcala, 2012; Greger, 2012]. Because BRAF V600E mutated tumors have been shown to develop resistance to BRAF inhibitors, the combination of a BRAFi and MEKi provides a rational approach for dual vertical inhibition

within the MAPK pathway to address such drug resistance [Johannessen, 2010]. Both have demonstrated substantial clinical activity with ORR between 28 to 53% and PFS ranging between 4.8 to 5.1 months. Further, the combination of both dabrafenib and trametinib indicated increased efficacy over both monotherapies with ORR of 63% and an increase of PFS to approximately 10 to 11 months for the 150 mg BID dabrafenib and 2 mg once daily trametinib dose cohort [Flaherty, 2012; Hauschild, 2012; Weber, 2012]. Furthermore, the combination of dabrafenib and rametinib has demonstrated overall survival of a median of more than 25 months in advanced melanoma. This robust survival data for the combination is further supported by other efficacy measures such as progression free survival of >11 months , overall response rates of about 64-69% (13-16% of patients achieving a complete response) with duration of response of 12.9-13.8 months and a disease control rate of 94% across two randomized Phase-III studies. (Long GV ,2015; Robert C, 2015).

Substantial safety and efficacy data across these clinical development programs in metastatic melanoma demonstrated the scientific validity of dual inhibition targeting the MAPK pathway through dabrafenib and trametinib in human cancer. The combination therapy of dabrafenib + trametinib may offer greater benefit to BRAF V600E mutated cancers than either agent alone.

2.2 Study Rationale

Mutations of BRAF V600E have been identified at a high frequency in melanoma, PTC, colorectal and ovarian cancers. Such mutations have also been reported in some rare cancers such as ATC, HCL, GIST, NSGCT/NGGCT, BTC, MM, ASI (including periampullary and ampullary), WHO Grade 1 and 2 gliomas and WHO Grade 3 and 4 (high-grade) gliomas. These rare cancers have an incidence rate in the US ranging between 0.07 to 6.3 per 100,000 and have a frequencyof BRAF V600E mutations ranging between 3% in WHO Grade 3 and 4 glioma to 90% in HCL (refer to Table 1) [Caronia, 2011; Tiacci, 2011; Hostein, 2010; Mayer, 2010; Honecker, 2009; Borger, 2012; Chapman, 2011a; Chapman, 2011b; Schonleben, 2009; Warth, 2011; Schindler, 2011]. These BRAF-mutated rare cancers have no effective treatment available either in the first-line setting for ATC, ASI and BRAF V600E-mutated GIST or in the refractory disease setting for NSGCT/NGGCT, BTC, WHO Grade 1 or 2 glioma, WHO Grade 3 or 4 glioma, HCL and MM. Therefore, there is a high unmet medical need for these rare cancerindications.

Table 1 Overall Incidence Rate and BRAF V600E Mutation Rate of Rare Cancers

Tumor Type	Overall Incidence Rate per 100,000 in US (2011)	BRAF V600E Mutation Rate (%)
Anaplastic Thyroid Cancer (ATC)	0.10	24%
Biliary Tract Cancer (BTC)	0.6	7 to 30%
Gastrointestinal Stromal Tumor (GIST)	0.7 to 1.1	2 to 5%
Germ Cell Tumor (GCT)<br>(approximately 50% non-seminomatous)	6.31 (White males)<br>1.38 (Black males)	3%
World Health Organization (WHO) Grade 1 or 2 glioma	6.5	Approximately 7%
WHO Grade 3 or 4 glioma	2 to 4	Approximately 3% (glioblastoma [GBM])
Hairy Cell Leukemia (HCL)	0.33	90 to 100%
Multiple Myeloma (MM)	5.579	4%
Adenocarcinoma of the Small Intestine (ASI)	0.073	Approximately 10%

References: Caronia, 2011; Tiacci, 2011; Hostein, 2010; Mayer, 2010; Honecker, 2009; Borger, 2012;Chapman, 2011b; Schonleben, 2009; Warth, 2011; Schindler, 2011

It is expected that in some of these BRAF-mutated rare cancers substantial clinical activity of BRAF and MEK inhibition will be observed similar to that in metastatic melanoma (breakthrough efficacy). For most rare cancers, conventional drug development programs are not feasible due to the low incidence of disease and the limited commercial opportunities. These challenges may reduce the incentive to explore the potential benefit of new targeted therapies for rare cancers. An alternative and likely more feasible approach may be based on the detection of BRAF V600E mutations in these rare cancers as a common denominator for investigation of a BRAF + MEK inhibitor in such well-defined subject populations. Enriched Bayesian statistical designs and a high target effect size may enable meaningful clinical investigation of targeted therapies in these small patient groups.

A somewhat similar approach was taken with imatinib, where several label indications were granted by US FDA based on an open-label, Phase II clinical trial of imatinib in subjects with multiple rare tumor types associated with imatinib sensitive tyrosine kinases along with published reports. The results of this one Phase II study in 186 subjects provided the basis for approvals in myelodysplastic/ myeloproliferative diseases, aggressive systemic mastocytosis, hypereosinophilic syndrome/chronic eosinophilic leukemia, and dermatofibrosarcoma protuberans. There were three responders out of seven subjects with myelodysplastic/ myeloproliferative diseases, one in five subjects with aggressive systemic mastocytosis, four out of 14 in the hypereosinophilic syndrome/chronic eosinophilic leukemia cohort and 10 out of 12 in the dermatofibrosarcoma protuberans cohort of that particular study [Heinrich, 2008]. It repeatedly appeared in the US FDA review of the submission for the extension of indication for Gleevec (2006) that "the (US) FDA medical reviewers recommended approval based on 'long duration of responses' and the rarity of disease and the occurrence of associated gene arrangement makes randomized trials impractical".

While it is anticipated that several rare cancers with V600E mutations will have clinically substantial response rates of 50% or more to BRAF plus MEK inhibition, in some settings resistance mechanisms may prevent such high response rates. For example, the BRAFi vemurafenib and dabrafenib have shown response rates of greater than 50% in BRAF-mutant metastatic melanoma, but show substantially lower response rates in BRAF-mutant metastatic colorectal cancer based on resistance mechanisms through receptor tyrosine kinases (RTKs) [Prahallad, 2012]. This reaffirms that pathways of resistance may vary between histologic disease entities and underscores – in the absence of a full understanding of such mechanisms of resistance for all histologies – that individual assessment of histologic types remains a relevant component of the investigation together with adequate PD markers to understand them.

Therefore, a Phase II study is proposed to investigate BRAF-mutant rare cancers in individual cohorts defined by histology with the objective to demonstrate breakthrough clinical effects from combination therapy with dabrafenib + trametinib. To address the inevitable small sample sizes per histologic cohort, the study will utilize a Bayesian hierarchical statistical design, which increases the power of the overall investigation by borrowing information across histology cohorts based on the emerging rate of response in these cohorts. [redacted]

2.3 Dose Rationale

[redacted]

In this study, dabrafenib will be administered at the dose of 150 mg BID continuously that demonstrated significant benefit in PFS in a Phase III trial [Hauschild, 2012]. Trametinib administered at a dose of 2 mg once daily has demonstrated significant benefit in PFS (hazard ratio [HR] = 0.45), a confirmed response rate (22% vs. 8%), and OS (HR = 0.54) relative to chemotherapy [Flaherty, 2012]. **As determined in Phase III studies, the safety profile of the combination of dabrafenib and trametinib generally reflects the well established safety profiles of the individual approved agents, with toxicities that are manageable with appropriate intervention.**

2.4 Design Rational

Due to the common pathway activation with the BRAF V600E mutation, multiple histologies may demonstrate substantial response to BRAF plus MEK inhibition. However, pathways of resistance may vary between histologic diseases resulting in different responses to the targeted combination therapy.

This single-arm, multi-histology study incorporates analyses based on a hierarchical model that allows for the possibility that the response profile for the population of histologies may be homogenous or heterogeneous. There may be ‘clusters’, or subsets of histologies, some in which the combination therapy is effective, and others not. The design borrows information in a limited sense from histologies that demonstrate similar response rates based on the accumulated trial data. If response rates are sufficiently different across particular histologies, the design recognizes this and borrowing is minimal. The analysis is entirely data driven. The number of clusters used to characterize the distribution of histologies is solely based on the observed responses. At each interim analysis as well as the final analysis, the entire model including clustering specification is fit based on the available data.

Prospectively, the entire design including model specification and decision criteria is developed by evaluating its performance from simulated trials. Hence, the false-positive rate can be controlled and power assessed. The advantages of a hierarchical model, both due to borrowing, are 1) increased precision of estimates of ORR, and 2) more accurate estimates of ORR.

Borrowing via a hierarchical model analysis is a type of shrinkage estimation; it provides a formal mechanism by which extreme observations are shrunk toward the mean. Shrinkage estimators have long been supported by literature. In particular, Berry et al. [Berry, 2013] highlighted that in the 1950s, Charles Stein [Stein, 1956; James, 1961], under normality, demonstrated that when there are three or more groups, estimating the groups separately (without borrowing) is inadmissible in the sense that the mean squared error is improved uniformly by borrowing.

For all these reasons, borrowing information generally increases the power to detect a clinically meaningful ORR compared to a traditional analysis while controlling the type I error rate. Independent analyses do not consider the possibility that some of the histologic cohorts may respond similarly to the targeted combination therapy. And due to the rarity of the biomarker subtypes of the diseases being considered, a traditional approach will likely have difficulty accruing a sufficient number of subjects to have adequate power to detect even a very large treatment effect in several of the histologic cohorts.

3 OBJECTIVES AND ENDPOINTS

The study objectives and corresponding endpoints are presented in Table 2.

Table 2 Study Objectives and Endpoints

OBJECTIVES	ENDPOINTS
**Primary**	
• To determine the ORR of dabrafenib and trametinib anti-cancer combination therapy in subjects with selected rare BRAF V600E mutated solid tumors or hematologic malignancies.	• Tumor response as defined by: RECIST, v1.1 for solid tumor histologies, Modified RANO and RANO for glioma or established response criteria for specific hematologic malignancies
**Secondary**	
• To determine the duration of response of dabrafenib in combination with trametinib in subjects with selected rare BRAF-mutated cancers	• Duration of response
• To determine PFS of dabrafenib in combination with trametinib in subjects with selected rare BRAF-mutated cancers	• Investigator-assessed PFS
• To determine OS of dabrafenib in combination with trametinib in subjects with selected rare BRAF-mutated cancers	• OS
• To determine the safety of dabrafenib in combination with trametinib in subjects with selected rare BRAF-mutated tumors	• Change from baseline in physical examination findings, vital signs, AEs, laboratory values and cardiac assessments
**Exploratory**	
• To identify candidate predictive and prognostic molecular features for each histology; to characterize treatment-emergent malignancies and to characterize the mechanisms of underlying AEs of special interest	• Comprehensive characterization of molecular background (DNA, RNA, protein) in tumor tissue at baseline and disease progression
[redacted]	

Abbreviations: AE, adverse events; [redacted]; ORR, overall response rate; OS, overall survival; [redacted]; PFS, progression-free survival; PGx,

Pharmacogenetics; [redacted]; RANO, Response Assessment for Neuro-Oncology; RECIST, Response Evaluation Criteria in Solid Tumors; [redacted]

4 STUDY DESIGN

This is a Phase II, open-label, non-randomized, multi-center study of oral dabrafenib in combination with oral trametinib in subjects with rare cancers with the BRAF V600E mutation. The following histologies will be included in this study: ATC, BTC, GIST, WHO Grade 1 or 2 glioma, WHO Grade 3 or 4 (high-grade) glioma, NSGCT/NGGCT, ASI, HCL and MM (Cohorts 1 to 9, respectively). This study is designed to determine the ORR of dabrafenib in combination with trametinib in subjects with rare BRAF V600E mutated cancers. Further supporting secondary objectives include the evaluation of duration of response, PFS, OS, and safety of the combination treatment. For each cohort, up to 25 evaluable subjects will be enrolled in the primary analysis cohort as defined in Section 5.1. If a given cohort is stopped early for efficacy, a histology specific expansion cohort may be opened to allow for additional patient enrollment (see Section 5.2). Only subjects with histologically or cytologically confirmed advanced disease with no available treatment options as determined by locally or regionally available standards of care and by the treating physician's discretion will be eligible for enrollment. Subjects may be enrolled based on local BRAF V600E mutation results and mutation status will be confirmed by a central reference laboratory.

Subjects will undergo screening assessments prior to the start of treatment to determine their eligibility for study enrollment. Baseline disease assessment must be completed prior to the first dose of study treatment. For female subjects, a pregnancy test must be performed within 7 days prior to first dose of study treatment. No study procedures, including submission of tumor tissue, will be performed unless written informed consent has been obtained. Screening evaluations will include demography, medical history, review of prior treatment, physical, dermatologic and ophthalmic examinations, vital signs, 12-lead ECG, ECHO, clinical laboratory assessments including urinalysis and histologic-specific assessments. Screening assessments may be repeated at the discretion of the investigator.

Subjects will have radiologic tests to identify target lesions and to assess disease status per protocol-defined response assessment criteria. Subjects will be evaluated following the first dose of study treatment according to the assessment schedule (see Time and Events Table, Section 7.1). Assessments may be performed more frequently if medically necessary. A treatment cycle is 28 days. Subjects will be assessed for AEs, study treatment compliance, and use of concomitant medications.

Subjects will receive dabrafenib 150 mg BID orally plus trametinib 2 mg once daily orally on a continuous dosing schedule. Subjects will undergo disease assessment of response after receiving at least 8 weeks of treatment. Dose adjustments will be made as needed based on toxicity. Subjects will continue treatment until an unacceptable toxicity, disease progression, or death occurs. Subjects who continue on treatment will undergo disease assessment according to the applicable response assessment criteria for the specific histology (i.e., for solid tumors, radiologic assessment every 8 weeks following initial response assessment). Subjects who experience disease progression will discontinue study treatments and be withdrawn from the study, unless the investigator, with approval of the Medical Lead, indicates that the subject may receive clinical benefit from continued study treatment. Once a subject discontinues treatment and/or is withdrawn from the study, a post-treatment follow-up visit will be conducted within 28 days (+7 days) after the last dose of study treatment(s)

with extended follow-up visits conducted every 4 weeks (±7 days) for the first 6 months and then every 3 months (±14 days) thereafter.

A hierarchical model that borrows information across cohorts will be utilized to strengthen the overall evidence within each cohort. Using the hierarchical model, continuous monitoring for both futility and efficacy will be undertaken throughout the study. Reviews will take place in approximately 12-week intervals depending on the enrollment rate. Enrollment for each cohort may be stopped early for futility or efficacy should various criteria occur based on accrued data. Response data from a minimum of five subjects will be required in a histologic cohort before it may discontinue enrollment for futility, and response data from a minimum of 10 subjects are required before discontinuing a histologic cohort for efficacy. In addition, at the final analysis and after the study has been closed, response data from a minimum of 2 subjects will be required in a histologic cohort in order to meet statistical success at the final analysis.

Decisions are based on whether the posterior probability that the ORR exceeds its corresponding historical control is sufficiently low or high compared to fixed statistical thresholds. Historical controls vary across histologic cohorts, but are all very low due to a lack of effective treatment options (Section 18.3.2.1). If this posterior probability is sufficiently low within a given histology (≤0.30), then enrollment may be halted early for futility. Conversely, if this posterior probability is sufficiently high within a given histology (≥0.95), then enrollment may be halted early for success. At the final analysis and after the study has been closed, if the posterior probability is sufficiently high (≥0.92), then the combination will be declared efficacious for that histology. Separate from model estimates, the observed ORR will also be reported for each histology. Further details regarding the statistical study design and analysis along with simulated example trials to illustrate the study may run is provided in Section 18.4.

Protocol waivers or exemptions are not allowed with the exception of immediate safety concerns. Therefore, adherence to the study design requirements, including those specified in the Time and Events Table (Section 7.1) is essential and required for study conduct. Supplementary study conduct information not mandated to be present in this protocol is provided in the accompanying study procedures manual (SPM). The SPM will provide site personnel with administrative and detailed technical information that does not impact subject safety.

4.1 Discussion of Design

The design of this Phase II study allows for the simultaneous evaluation of clinical activity and early signals of efficacy in each of the histologies under study. In addition, the safety of dabrafenib in combination with trametinib will be evaluated to support the use of the combination treatment in subjects with selected rare tumor types. Study treatment will begin on Study Day 1 and will continue until protocol-defined treatment withdrawal criteria are met (Section 16). While on study treatment, subjects will be closely monitored for safety. Procedures to minimize or monitor potential risks, dose modification guidance, and supportive care recommendations are provided in Section 8.1.

In the event one of the combination agents is discontinued, subjects may still receive the remaining agent (either dabrafenib or trametinib) as monotherapy provided the investigator concludes that continued therapy will be in the best interest of the subject and will result in potential clinical benefit. Once subjects are permanently withdrawn from all study treatments,

a post-treatment follow-up assessment should be completed within approximately 28 days from the last dose of study treatment.

4.1.1 BRAF Mutation Test and Companion Diagnostic

Subjects will be enrolled in the study based on local BRAF V600 mutation test results.

- For subjects with solid tumors to be eligible for the study, archived tumor tissue sample(s) and/or fresh tumor tissue sample(s) must be available to allow for central confirmation of BRAF V600E mutation status.
- For subjects with HCL or MM, a fresh BM aspirate sample and a peripheral blood sample must be available to allow for central confirmation of BRAF V600E mutation status. See Section 7.3.1 for additional details.

A sponsor designated central reference laboratory will be employed for the central confirmation of BRAF V600 mutation status. For solid tumors or hematological malignancies where application of the sponsor designated test is not feasible, an alternative approach of BRAF V600E mutation testing by immunohistochemistry (IHC) or Sanger sequencing may be employed. The primary analysis to determine the efficacy of dabrafenib + trametinib therapy for the rare cancers will be based on the BRAF V600E mutation status determined by central reference laboratory testing.

5 SUBJECT SELECTION

5.1 Primary analysis cohort

The "primary analysis cohort" will be comprised of those patients enrolled within a histology-specific group prior to capping at 25 patients per cohort or prior to early stopping for efficacy or futility. The primary analysis cohort will form the basis of the Bayesian modelling.

5.2 Expansion cohorts

If a cohort closes early at an interim analysis because it meets the rules for early stopping for efficacy, an expansion cohort may be opened to allow additional patient enrollment for that particular histology. The patients in the expansion cohort will provide supportive efficacy data and will NOT contribute to the Bayesian modeling. The expansion cohort(s) will enroll subjects for the duration of trial enrollment. The same general and histology specific eligibility criteria and T&E tables and biomarker/histology confirmation analyses will apply to patients in the expansion cohort.

5.3 Number of Subjects

Subjects with BRAF V600E positive-mutations will be enrolled in separate cohorts based on histologic type. Once 16 weeks of response data is available from a minimum of 5 subjects in a particular cohort, interim analyses will occur every 12 weeks to assess whether enrollment in the particular cohort will be continued or closed. **For the primary analysis cohort,** enrollment will not exceed 25 subjects per histology cohort to ensure adequate subjects in the BRAF V600E confirmed population. After 25 subjects have been enrolled, the histology cohort will be closed to further enrollment for patients contributing to the Bayesian modeling. Histology specific expansion cohorts may be opened to allow additional patient enrollment. See Section 18.2 for sample size assumptions.

The study is expected to enroll subjects over an approximate 3 to 4 year time period. This flexibility is intended to accommodate recruitment rates in these rare histologic cohorts. Projected 3-year recruitment rates are described in Section 18.4.1.2, and are used for simulated trials to assess design operating characteristics.

5.4 Eligibility

Specific information regarding warnings, precautions, contraindications, AEs, and other pertinent information on the study treatment(s) that may impact subject eligibility is provided in the IB for dabrafenib + trametinib combination [GlaxoSmithKline Document Number CM2010/00010/05].

Deviations from inclusion/exclusion criteria are not allowed because they can potentially jeopardize the scientific integrity of the study, regulatory acceptability or subject safety. Therefore, adherence to the eligibility criteria as specified in the protocol is essential.

5.4.1 Inclusion Criteria

Subjects must meet all of the following inclusion criteria in order to be considered eligible for this study:

1. Signed, written informed consent
2. Sex: male or female
3. Age: ≥18 years of age at the time of providing informed consent
4. Eastern Cooperative Oncology Group (ECOG) performance status: 0, 1 or 2 (Appendix 1).
5. Must have advanced disease and no standard treatment options as determined by locally/regionally available standards of care and treating physician's discretion.
6. Must have a BRAF V600E mutation-positive tumor as confirmed by an approved local laboratory or a sponsor designated central reference laboratory.
7. **NOTE:** All subjects must provide an archived or fresh tumor sample (for solid tumors) or a fresh BM aspirate and peripheral blood sample (for HCL and MM) for confirmation testing of the BRAF V600E mutation by a sponsor designated central reference laboratory using a sponsor designated assay. See Section 7.3.1 for additional details.
8. ATC, BTC, GIST, NSGCT/NGGCT, and ASI ONLY: Must have at least one measurable lesion per RECIST 1.1 outside of a prior radiation field or within the field with evidence of progression.
9. Able to swallow and retain orally administered medication.
10. Female Subjects of Childbearing Potential: Subjects must have a negative serum pregnancy test within 7 days prior to the first dose of study treatment and agrees to use highly effective contraception, as defined in Section 9.1, from 7 days prior to enrollment, throughout the treatment period and for 16 weeks following discontinuation of trametinib when taken in combination with dabrafenib, or for 2 weeks following discontinuation of dabrafenib monotherapy.
11. Has adequate baseline organ function as outlined in Table 3

Table 3 Adequate Baseline Organ Function

SYSTEM Adequate Baseline Organ Function	TEST	Histology	LABORATORY VALUES[1]
Hematologic	ANC[3]	ATC, BTC, GIST, WHO Grade 1-4 Glioma, NSGCT/NGGCT, ASI	≥1.2 × 10^9/L
		MM	≥1 × 10^9/L
	Hemoglobin[3]	ATC, BTC, GIST, WHO Grade 1-4 Glioma, NSGCT/NGGCT, ASI	≥9 g/dL
	Platelets[3]	ATC, BTC, GIST, NSGCT/NGGCT, ASI	≥75 × 10^9/L
		MM	≥50 × 10^9/L
		WHO Grade 1-4 Glioma	≥100 × 10^9/L
	PT, PTT, INR[2]	ATC, BTC, GIST, WHO Grade 1-4 Glioma, NSGCT/NGGCT, ASI, HCL, MM	≤1.5 times ULN
Hepatic	Albumin	ATC, BTC, GIST, WHO Grade 1-4 Glioma, NSGCT/NGGCT, ASI, HCL, MM	≥2.5 g/dL
	Total Bilirubin[4]	ATC	≤1.5 times ULN
		BTC	≤3 times ULN if stable for 14 days prior to enrollment
		GIST, WHO Grade 1-4 Glioma, NSGCT/NGGCT, MM	≤2 times ULN
		HCL	<3 times ULN
		ASI	≤2 times ULN OR ≤3 times ULN if stable for 14 days prior to enrollment for ampullary and periampullary carcinoma
	ALT and AST[5]	ATC, GIST, NSGCT/NGGCT	≤2 times ULN without liver metastases ≤2.5 times ULN if documented liver metastases
		BTC, ASI, MM	≤3 times ULN without liver metastases ≤5 times ULN if documented liver metastases
		HCL	<5 times ULN
		WHO Grade 1, 2, 3 or 4 Glioma	≤3 times ULN
Renal	Creatinine	ATC, BTC, GIST, WHO Grade 1-4 Glioma, NSGCT/NGGCT, ASI, HCL, MM	≤1.5 mg/dL
	OR		
	Calculated Creatinine Clearance[6] or 24-hr Urine Creatinine Clearance	ATC, BTC, GIST, WHO Grade 1-4 Glioma, NSGCT/NGGCT, ASI, HCL, MM	≥50 mL/min
Cardiac	LVEF	ATC, BTC, GIST, WHO Grade 1-4 Glioma, NSGCT/NGGCT, ASI, HCL, MM	≥LLN[7] by ECHO

Abbreviations: ALT, alanine aminotransferase; ANC, absolute neutrophil count; ASI, adenocarcinoma of small intestine AST, aspartate aminotransferase; CBC, complete blood count; ECHO, echocardiogram; HCL, hairy cell leukemia; hr, hour; INR, international normalization n ratio; LLN, lower limit of normal; LVEF, left ventricular ejection fraction; PT, prothrombin time; PTT, partial thromboplastin time; ULN, upper limit of normal

Laboratory values are obtained within 14 days prior to first dose of study treatment.

PTT and PT/INR >1.5 times ULN will be acceptable in case of subjects receiving therapeutic anticoagulants such as warfarin as long as INR is monitored during the study according to clinical practice.

HCL: No minimum CBC criteria if the cytopenias are due to disease under study.

HCL: Subjects will be allowed to enroll with indirect hyperbilirubinemia; however, this condition must be confirmed due to hemolysis,

SYSTEM Adequate Baseline Organ Function	TEST	Histology	LABORATORY VALUES[1]
hypersplenism or direct bilirubin ratio <35%, and requires approval from the Medical Lead.<br>HCL: Subjects will be allowed to enrol with elevated ALT/AST values up to 5 times ULN; however this condition must be confirmed due to hemolysis and/or hypersplenism and not associated with hepatic dysfunction and required approval from the Medical Lead.<br>Calculated by the Cockcroft-Gault formula (see Appendix 3).<br>If LLN is not defined for a given institution, then ejection fraction must be ≥ 50%.<br>NOTE: Subjects with AST, ALT or total bilirubin values outside the range(s) in Table 3 due to Gilbert's syndrome or asymptomatic gallstones are not excluded. Laboratory results obtained during Screening should be used to determine eligibility criteria. In situations where laboratory results are outside the permitted range, the investigator may opt to retest the subject and the subsequent within-range screening result may be used to confirm eligibility			

12. French subjects: In France, a subject will be eligible for inclusion in this study only if either affiliated to or a beneficiary of a social security category.

5.4.1.1 Histology Specific Inclusion Criteria

5.4.1.1.1 ATC

1. Histologically or cytologically confirmed, unresectable, metastatic ATC including ATC originating from within well-differentiated thyroid cancers or an ATC as part of a thyroid carcinoma of another histologic type.
   NOTE: Squamoid differentiated subtype is not permitted. Diagnosis noted to be "consistent with ATC" with presence of thyroid mass is acceptable.
2. Has undergone evaluation via indirect or direct laryngoscopy to ensure patency of trachea/airway prior to enrollment if bulky thyroid/neck masses are present and/or airway obstruction is suspected.
3. Has undergone prior external beam radiotherapy and/or surgery to the primary tumor.
   NOTE: Subjects with small primary tumor that has been totally removed by surgical excision whereby no radiotherapy was indicated (only metastatic lesions are manifested) or subjects with metastatic disease who do not require radiation or surgery to the neck mass will be eligible for participation in the study.

5.4.1.1.2 BTC

1. Histologically or cytologically confirmed, unresectable, metastatic or locally advanced or recurrent adenocarcinoma of the biliary tract or gallbladder
2. Must have progressed on or demonstrated intolerance (despite standard measures of supportive care and dose reduction) to treatment with a gemcitabine-based chemotherapy regimen.

5.4.1.1.3 GIST

1. Histologically confirmed diagnosis of c-Kit and PDGFRA wild-type GIST.
2. Must have metastatic or locally advanced, unresectable, or recurrent post-surgical disease.
3. Must have progressed on or demonstrated intolerance (despite standard measures of supportive care and dose reduction) to treatment with a TKI (i.e., imatinib, sunitinib).

5.4.1.1.4 WHO Grade 1 or 2 Glioma

1. Histologically confirmed recurrent or progressive WHO Grade 1 or 2 glioma

2. **For WHO Grade 1 glioma ONLY:** To be considered eligible for study treatment, subject must:
    a. Present with adequate level of favourable risk/benefit ratio including, but not limited to, the exhibited initial symptoms, AND
    b. Be evaluated by a panel of neuro-oncologists and the Medical Lead prior to enrollment.
3. **For WHO Grade 2 glioma ONLY:** To be considered eligible for study treatment, subject must not be eligible for treatment with chemotherapy.
4. Must have measurable non-enhancing disease based on two-dimensional magnetic resonance imaging (MRI) with contrast assessments as per RANO response criteria (Appendix 13).
   NOTE: Enhancing disease is acceptable for pilocytic astrocytomas.
5. For WHO Grade 1 or 2 glioma subjects receiving corticosteroid treatment: Must be receiving a stable or decreasing dose of corticosteroid treatment for 7 days prior to first dose of study treatment.
   NOTE: Steroids dose is limited to up to 8 mg/day of dexamethasone or equivalent dose of steroid.
   NOTE: Subjects are not required to be on corticosteroids to be eligible.

5.4.1.1.5 WHO Grade 3 or 4 Glioma

1. Histologically confirmed recurrent or progressive WHO Grade 3 or 4 glioma.
2. Had prior treatment with radiotherapy and first-line chemotherapy or concurrent chemoradiation therapy.
   NOTE: Subjects who have a WHO Grade 3 or 4 glioma for which chemotherapy and/or radiotherapy is not considered standard of care may remain eligible for the study. Consult the Medical Lead to discuss and determine if subject is eligible for enrollment.
3. Must have measurable disease at least 1 cm x 1 cm as per RANO response criteria (Appendix 14).
4. **For WHO Grade 3 or 4 glioma subjects receiving corticosteroid treatment**: Must be receiving a stable or decreasing dose of corticosteroid treatment for 7 days prior to first dose of study treatments.
   NOTE: Steroids dose is limited to up to 8 mg/day of dexamethasone or equivalent dose of steroid.
   NOTE: Subjects are not required to be on corticosteroids to be eligible.

5.4.1.1.6 NSGCT/NGGCT

1. Histologically confirmed NSGCT/NGGCT.
2. Must have either:
    - ***Refractory disease*** defined as disease progression during or relapsed after salvage high-dose chemotherapy (HDCT), or disease progression during cisplatin-based salvage chemotherapy.
      OR
    - ***Relapsed disease*** and ineligible for cisplatin-based salvage chemotherapy or HDCT.

5.4.1.1.7 ASI

1. Histologically confirmed, metastatic or locally advanced ASI, adenocarcinoma of the ampulla, or adenocarcinoma of the peri-ampulla.
2. Must have progressed on or demonstrated intolerance (despite standard measures of supportive care and dose reduction) to one line of chemotherapy.

5.4.1.1.8 HCL

1. Histologically confirmed diagnosis of HCL according to morphological and immunophenotypic criteria of WHO classification [WHO, 2008] of lymphoid neoplasms.
2. Must have either:
   - ***Refractory*** disease defined as no response or disease progression in ≤1 year following first-line treatment with a purine analog (i.e., pentostatin, cladribine or fludarabine)
     OR
   - ***Relapsed*** disease defined as having relapsed following treatment with at least two prior treatments
3. Must have the presence of leukemic cells in the peripheral blood or BM aspirate or BM biopsy AND one or more of the following:
   - Bulky/symptomatic splenomegaly
   - Hemoglobin <10 g/dL
   - Platelets $<100 \times 10^9$/L
   - Absolute neutrophil count (ANC) $<1 \times 10^9$/L
4. For subjects with an opportunistic infection: the infection must be adequately managed and the subject must be clinically stable. The investigator may discuss this issue with the Medical Lead.MM

5.4.1.1.9 MM

1. Histologically confirmed secretory MM.
2. Has received at least 2 prior lines of therapy, such as prior treatment with a proteasome inhibitor and IMiD, and is now refractory or has demonstrated intolerance (despite standard measures of supportive care and dose reduction) to the most recent therapy received
3. Must have measurable disease of MM as defined by at least **ONE** of the following:
   - Serum M-protein ≥1 g/dL (≥10 g/L)
   - Urine M-protein ≥200 mg/24 hr
   - Serum free light chain (FLC) assay: involved FLC level ≥5 mg/dL (≥50 mg/L) and an abnormal serum FLC ratio (<0.26 or >1.65), or
   - Biopsy proven plasmacytoma (measured within 28 days of Screening)
4. **For subjects with an opportunistic infection:** the infection must be adequately managed and the subject must be clinically stable. The investigator may discuss this issue with the Medical Lead.

5.4.2 Exclusion Criteria

ANY subject who meets any of the following criteria is excluded from enrollment in this study:

1. Prior treatment with:
   - BRAF and/or MEK inhibitor(s)
   - Chemotherapy, immunotherapy, biologic therapy or chemoradiation with delayed toxicity within 21 days (or within 42 days if prior therapy contains nitrosourea or mitomycin C) prior to enrollment
   - Chemotherapy or biologic therapy without evidence of delayed toxicity within 14 days prior to enrollment
   - Investigational product(s) (IP) within 30 days or 5 half-lives, whichever is longer, prior to enrollment
     - **Subjects enrolled in France:** Subject has participated in any study using an IP(s) within 30 days prior to enrollment in this study.
2. History of malignancy with confirmed activating RAS mutation at any time.
   NOTE: Prospective RAS testing is not required. However, if the results ofprevious RAS testing are known, then those results must be used in assessing eligibility.
3. Prior radiotherapy less than 14 days prior to enrollment, except for WHO Grade 1-4 glioma and ATC. Treatment-related AEs must have resolved prior to enrollment.
   For WHO Grades 1, 2, 3, or 4 Glioma ONLY: Radiotherapy is not permitted within 3 months prior to enrollment (extended period of time of > 3 months needed to prevent subjects with pseudo-progression from radiotherapy from being enrolled in the study). Subjects may be ≥ 2 weeks from radiotherapy if a new lesion relative to the pre-radiation MRI develops outside the primary radiation field. Treatment-related AEs must have resolved prior to enrollment.
   For ATC Only: Radiotherapy is not permitted within 7 days prior to enrollment. Treatment-related AE(s) must have resolved prior to enrollment.
4. Prior major surgery less than 14 days prior to enrollment. Any surgery-related AE(s) must have resolved prior to enrollment.
5. Prior solid organ transplantation or allogenic stem cell transplantation (alloSCT)
   NOTE: Previous autologous BM transplant (autoSCT) or autologous peripheral blood stem cell transplant (PBSCT) is permitted.
6. History of another malignancy
   NOTE: Subjects with another malignancy are eligible if:
   a. disease-free for 3 years, or
   b. have a history of completely resected non-melanoma skin cancer, and/or
   c. have an indolent second malignancy(ies).
   Consult a Medical Lead if unsure whether second malignancies meet requirements specified above.
7. Presence of:
   - Brain metastases (*except* for subjects in the WHO Grade 1 or 2 Glioma or WHO Grade 3 or 4 Glioma histology cohorts) that are symptomatic or untreated or not stable for ≥ 3 months (must be documented by imaging) or requiring corticosteroids. Subjects on a stable dose of corticosteroids > 14 days and have not

required treatment with enzyme-inducing anticonvulsants for >30 days prior to enrollment can be enrolled with approval of the Medical Lead.

- Symptomatic or untreated leptomeningeal or spinal cord compression
  NOTE: Subjects who have been previously treated for these conditions and have stable CNS disease (documented by consecutive imaging studies) for >60 days, are asymptomatic and currently not taking corticosteroids, or have been on a stable dose of corticosteroids for at least 30 days prior to enrollment, are permitted.
- Interstitial lung disease or pneumonitis
- Any unresolved ≥Grade 2 (per Common Terminology Criteria for Adverse Events [CTCAE] version 4.0) toxicity from previous anti-cancer therapy at the time of enrollment, except alopecia or Grade 2 anemia
  NOTE: Subjects with MM who have ≤Grade 2 peripheral neuropathy (per CTCAE v4.0) are permitted.
- Any serious and/or unstable pre-existing medical disorder, psychiatric disorder, or other conditions that could interfere with subject's safety, obtaining informed consent or compliance to the study procedures

8. History of RVO
9. Clinically significant GI abnormalities that may alter absorption such as malabsorption syndrome or major resection of the stomach or bowels. For example, subjects should have no more than 50% of the large intestine removed and no sign of malabsorption (i.e., diarrhea).
   NOTE: If clarification is needed as to whether a condition will significantly affect the absorption of study treatments, contact the Medical Lead.
10. History or evidence of cardiovascular risk including any of the following:
    - Acute coronary syndromes (including myocardial infarction and unstable angina), coronary angioplasty, or stenting within 6 months prior to enrollment
    - Clinically significant uncontrolled arrhythmias
      NOTE: Subjects with controlled atrial fibrillation for >30 days prior to enrollment are eligible.
    - Class II or higher congestive heart failure as defined by the New York Heart Association (NYHA) criteria (Appendix 2)
    - Left ventricular ejection fraction (LVEF) below the institutional LLN
      NOTE: If a LLN does not exist at an institution, then use LVEF<50%.
    - Abnormal cardiac valve morphology (≥Grade 2) documented by ECHO
      NOTE: Subjects with Grade 1 abnormalities (i.e., mild regurgitation/stenosis) may be entered on study. Subjects with moderate valvular thickening should NOT be enrolled.
    - Corrected QT (QTc) interval for heart rate using Bazett-corrected QT interval (QTcB) ≥480 msec
    - Intracardiac defibrillator
    - Treatment-refractory hypertension defined as a blood pressure (BP) >140/90 mmHg which may not be controlled by anti-hypertensive medication(s) and/or lifestyle modifications
    - **Subjects enrolled in Germany:** Subjects with a left bundle branch block (LBBB) are NOT eligible for inclusion in this study.

11. Presence of hepatitis B surface antigen (HBsAg), positive hepatitis C antibody test result within 3 months prior to first dose of study treatment.
    NOTE: Subjects with positive Hepatitis C antibody due to prior exposure can be enrolled, only if a confirmatory negative Hepatitis C RNA polymerase chain reaction (PCR) test is obtained.
12. Current use of prohibited medication(s) or requirement for prohibited medications during study (see Section 10.2).
    NOTE: Use of anticoagulants such as warfarin is permitted; however, international normalization ratio (INR) must be monitored according to local institutional practice.
13. Clinically significant known immediate or delayed hypersensitivity reaction or idiosyncrasy to drugs chemically related to study treatment, or excipients, or to dimethyl sulfoxide (structural component of dabrafenib).
14. **Female subjects:** Pregnant, lactating or actively breastfeeding

5.4.2.1 Histology Specific Exclusion Criteria

5.4.2.1.1 ATC

1. Presence of thyroid lymphomas, sarcomas, or metastatic disease from other sites of origin to the thyroid.
2. Has potentially curable ATC by surgical excision alone or subjects who havenot received treatment that might be considered standard of care.

5.4.2.1.2 BTC

1. Has biliary duct obstruction, unless a treatable, clinically relevant obstruction has been relieved by internal endoscopic drainage/stenting, palliative by-pass surgery or percutaneous drainage prior to enrollment.

5.4.2.1.3 WHO Grade 1, 2, 3 or 4 Glioma

1. Prior treatment with enzyme-inducing anticonvulsants within 14 days prior to enrollment.
2. Radiotherapy treatment within 3 months prior to enrollment.
   NOTE: Extended period of time (>3 months) needed to prevent subjects with pseudo-progression from radiotherapy being enrolled in the study. **Subjects may be ≥2 weeks from radiotherapy if a new lesion relative to the pre-radiation MRI develops outside the primary radiation field. Treatment-related AEs must have resolved prior to enrollment.**

6 STUDY TREATMENTS

The term ‘study treatment’ is used throughout the protocol to describe any combination of IP received by the subject as per the protocol design. Study treatment may therefore refer to the individual study treatments or the combination of those study treatments.

6.1 Treatment Assignment

Subjects will be identified by a unique subject number that will remain consistent for the duration of the study. Subjects will be categorized by histology group based on tumor type and mutation status in a non-randomized manner.

Upon completion of all the required screening assessments, including signed informed consent; eligible subjects will be registered into the designated interactive voice response system (IVRS) by the investigator or authorized site staff.

6.2 Blinding

This is an open-labeled study.

6.3 Investigational Products

6.3.1 Dabrafenib (GSK2118436) Capsule

Dabrafenib capsules will be provided to sites by the sponsor. The contents of the label will be in accordance with all applicable regulatory requirements.

	**Investigational Product**	
**Product name :**	**Dabrafenib (GSK2118436) 50 mg**	**Dabrafenib (GSK2118436) 75 mg**
**Formulation description:**	Each capsule contains GSK2118436B equivalent to 50 mg or 75	
**Dosage form :**	Capsule	
**Unit dose strength(s):**	50 mg	75 mg
**Physical Description:**	50 mg: [redacted]	75 mg: [redacted]
**Route/Schedule/ Duration:**	Oral/ BID/Continuous	

Abbreviations: BID, twice daily; HPMC, hydroxypropyl methyl cellulose

6.3.1.1 Administration and Storage of Dabrafenib Capsules

Administration: Dabrafenib capsules must be dispensed only to subjects enrolled in the study and in accordance with the protocol. Dabrafenib should be administered under fasted conditions, either 1 hr before or 2 hrs after a meal with approximately 200 mL of water. Subjects should be encouraged to take their doses at 12 hr intervals and at similar times every day.

Missed Dose: If a subject misses a dose of dabrafenib, the subject may take the dose immediately if the next dose is scheduled for at least 6 hrs later. If the next scheduled dose is due in less than 6 hrs, the subject should skip the dose and resume dosing at the next scheduled dose.

Storage: Dabrafenib must be stored in a secure area under the appropriate physical conditions for the product. Access to and administration of the dabrafenib will be limited to the investigator and authorized site staff.

Dabrafenib is to be stored and dispensed in an opaque bottle. The recommended storage conditions for dabrafenib are stated on the product label. Maintenance of a temperature log (manual or automated) at the site is required.

6.3.2 Trametinib (GSK1120212) Tablet

Trametinib tablets will be provided to sites by the sponsor. The contents of the label will be in accordance with all applicable regulatory requirements.

	**Investigational Product**	
**Product name :**	**Trametinib (GSK1120212) 0.5 mg**	**Trametinib (GSK1120212) 2 mg**
**Formulation description:**	Each immediate-release oral tablet contains GSK1120212B equivalent to 0.5 mg or 2 mg of GSK1120212 [redacted]	
**Dosage form :**	Tablet	
**Unit dose strength(s):**	0.5 mg	2 mg
**Physical Description:**	[redacted]	[redacted]
**Route/Schedule/Duration:**	Oral/once-daily/Continuous	

6.3.2.1 Administration and Storage of Trametinib Tablets

Administration: Trametinib must be dispensed only to subjects enrolled in the study and in accordance with the protocol. Trametinib should be administered under fasted conditions, either 1 hr before or 2 hrs after a meal with approximately 200 mL of water. Subjectsshould be instructed to take their dose of trametinib concurrently with the morning dose of dabrafenib.

Vomiting: If a subject vomits after taking study treatment, the subject should be instructed not to retake the dose but should be instructed to take the next scheduled dose.

Missed Dose: If a subject misses a dose of trametinib, the subject may take the dose immediately if the next dose is scheduled for at least 12 hrs later. If the next scheduled dose is due in less than 12 hrs, the subject should skip the dose and resume dosing at the next scheduled dose.

Storage: Trametinib must be stored in a secure area under the appropriate physical conditions for the product. Trametinib is to be stored at the temperature specified on the label. Maintenance of a temperature log (manual or automated) is required. Access to and administration of trametinib will be limited to the investigator and authorized site staff. Refer to the SPM for further detailed instructions on IP storage.

6.4 Handling/Accountability of Investigational Products

Handling: Adequate precautions must be taken to avoid direct contact with the IP. The occupational hazards and recommended handling procedures are provided in the Material Safety Data Sheet (MSDS). A description of the occupational hazards and recommended handling precautions for each IP (MSDS) will be provided to the site staff and/or subjects if required by local laws or will otherwise be available from the sponsor upon request. In the case of unintentional occupational exposure, notify the study monitor, the Medical Lead, and/or study manager.**Accountability:** In accordance with local regulatory requirements, the

investigator, designated site staff, or head of the medical institution (where applicable) must document the amount of each IP dispensed and/or administered to study subjects, the amount returned by study subjects, and the amount received from and returned to the sponsor, when applicable. Accountability records for each IP must be maintained throughout the course of the study. Refer to the SPM for further detailed instructions on IP accountability.

6.5 Treatment Compliance

Subjects will be asked to maintain a dosing diary to record their daily administration of study treatments. At each visit, the subject should return the dosing diary; an evaluation of subject compliance with study treatments taken will be performed. The investigator will make every effort to bring non-compliant subjects into compliance. A record of the number of trametinib tablets and dabrafenib capsules dispensed to and taken by each subject must be maintained and reconciled with study treatment and compliance records. Compliance with dosing will be assessed through querying the subject during site visits and documented in the source documents and electronic case report form (eCRF) including treatment start and stop dates, dates for treatment delays and/or dose reductions.

6.6 Treatment of Overdose

6.6.1 Dabrafenib

In the event of a dabrafenib overdose (defined as administration of >300 mg as a single dose or 600 mg daily, the highest dose administered to date), the investigator should contact the Medical Lead immediately and closely monitor the subject for AEs/SAEs and laboratory abnormalities. The sponsor does not recommend specific treatment. The investigator will use clinical judgment to treat any overdose. Hemodialysis is not expected to enhance the elimination of dabrafenib as it is highly bound to plasma proteins.

[redacted]

Information regarding the quantity of the excess dose as well as the duration of the overdosing should be documented in the eCRF.

6.6.2 Trametinib

In the event of a trametinib overdose, defined as administration of more than 3.0 mg once daily (the maximum tolerated dose defined in the study MEK111054), the investigator should contact the Medical Lead immediately and closely monitor the subject for AEs/SAEs and laboratory abnormalities. The sponsor does not recommend specific treatment. The investigator will use clinical judgment to treat any overdose. Hemodialysis is not expected to enhance the elimination of trametinib as it is highly bound to plasma proteins.

Decisions regarding dose interruptions or modifications will be made by the investigator in consultation with the Medical Lead based on the clinical evaluation of the subject.

[redacted]

Information regarding the quantity of the excess dose as well as the duration of the overdosing should be documented in the eCRF.

6.7 Continuation of Treatment

In the absence of an unacceptable toxicity, disease progression or withdrawal of consent by subject, treatment may continue. However, if at the time of investigator-reported disease progression, the investigator determines that the subject is still clinically benefitting from study treatment(s) and the subject is willing to continue study treatment(s); consultation with the Medical Lead is required. If further progressive disease is noted at the next response assessment, the subject must discontinue study treatment(s).

If the study treatment(s) is continued beyond the initial disease progression, study procedures, including response assessments, will be continued according to the Time and Events Table (Section 7.1)

7 STUDY ASSESSMENTS AND PROCEDURES

Study assessments and procedures, including the timing of the assessments, are summarized in the Time and Events Table (Section 7.1).

A signed, written informed consent form (ICF) must be obtained from the subject or a legal representative prior to any study-specific procedures or assessments.

Procedures conducted as part of the subject's routine clinical management (e.g., blood count, imaging study) and obtained prior to signing of informed consent may be utilized for screening or baseline purposes provided the procedure meets the protocol-defined criteria and has been performed in the timeframe of the study.

Investigators may be requested to perform additional safety tests during the course of the study based on newly available data to ensure appropriate safety monitoring. Appropriate local regulatory and ethical approvals should be obtained before any additional testing is performed.

7.1 Time and Event Table

Table 4 Time and Event Table for All Cohorts

		Treatment[5]						Follow-up Visit[55]	Extended Follow-up[58]
	Screening	Day 1 [7]	Day 15 (±1 day)	Every 4 weeks (±3 days)	Every 8 weeks (±3 days)	Every 12 weeks (±3 days)	Every 16 weeks (±3 days)	Treatment or Study Withdrawal	Every 3 months (±14 days) *except when noted*
Clinical Assessments: ALL Cohorts									
Informed Consent[1]	X								
Demographics	X								
Medical History[2]	X	X[8]							
Disease Characteristics[3]	X								
BRAF V600E Mutation Testing (Mandatory)[4]	X								
ECOG Performance Status	X[6]	X[8]		X				X	
Safety Assessments: ALL Cohorts									
Physical Exam (including weight)	X (Complete)[6,9]	X (Brief)[10]		X (Brief)[10]				X (Complete)[9]	
Height	X[6,11]								
Dermatologic Exam	X (Full)[12]			X (Brief)[13]				X (Brief)[13]	X (Brief)[13]
Ophthalmic Exam[14]	X			Week 4 Only					
Vital Signs[15]	X[6]	X[8]		X				X	
12-lead ECG[16]	X[6]	X[8]		X				X[56]	
ECHO[17]	X			Week 4 Only		X		X[57]	

[redacted]

	Screening	Treatment[5]						Follow-up Visit[55]	Extended Follow-up[58]
		Day 1 [7]	Day 15 (±1 day)	Every 4 weeks (±3 days)	Every 8 weeks (±3 days)	Every 12 weeks (±3 days)	Every 16 weeks (±3 days)	Treatment or Study Withdrawal	Every 3 months (±14 days) *except when noted*
Adverse Events		Continuous							
Safety Assessments: ALL Cohorts continued									
Concomitant Medications	X[6]	X[8]	X	X				X	
Laboratory Assessments: ALL Cohorts									
Chemistry and Hematology	X[6]	X[8]		X				X[56]	
Pregnancy test[19]	X[20] (serum)				X[21] (serum or urine)	X[21] (serum or urine)		X[21,56] (serum or urine)	
HbA1c	X[6]					X			
Coagulation: PT, PTT, INR	X[6]								
Urinalysis	X[6]	X[8]		X				X[56]	
Monitoring for Non-Cutaneous Secondary/Recurrent Malignancy[22]: ALL Cohorts									
Head and neck exam[23]	X[6]					X			X[24]
Chest and Abdominal CT scan or MRI	X[6]	As clinically indicated							X[24]
Disease Assessment: ATC, ASI, BTC, GIST, NSGCT/NGGCT, and WHO Grade 1-4 Glioma Cohorts ONLY									
Disease Assessment: Imaging[25]	X[26]				X[27]	X[27]		X[27]	X[27]
TSH, Free T4 (ATC Only)	X[6]				X			X	
Histology Confirmation: ATC and WHO Grade 1-4 Glioma Cohorts ONLY									
Tissue Sample for Histology Confirmation[28]		X							
Disease Assessment: NSGCT/NGGCT Cohort ONLY									
AFP, β-HCG	X[6]			X[29]	Every 8 to 12 weeks[29]			At Disease Progression[29]	

	Screening	Treatment[5]						Follow-up Visit[55]	Extended Follow-up[58]
		Day 1 [7]	Day 15 (±1 day)	Every 4 weeks (±3 days)	Every 8 weeks (±3 days)	Every 12 weeks (±3 days)	Every 16 weeks (±3 days)	Treatment or Study Withdrawal	Every 3 months (±14 days) *except when noted*
Disease Assessment: BTC and ASI Cohorts ONLY									
CEA, CA19-9	X[6]			X[30]	Every 8 to 12 weeks[30]			At Disease Progression[30]	
Disease Assessment: HCL Cohort ONLY[31]									
Blood sample for CBC	X[6]			X[31]	X[31]				
Peripheral Blood Sample Staining for Hairy Cell count	X			X[31]	X[31]				
BM biopsy and aspirate with H/E stain and IHC	X[34]	When counts are consistent with CR or PR for 4 weeks; if evidence of response, then after every 6 months twice, then after a year twice and then every 2 years[32]						At time of relapse	
Flow cytometry: BM aspirate sample	X[34]	When counts are consistent with CR or PR for 4 weeks; if evidence of response, then after every 6 months twice, then after a year twice, and then every 2 years[33]						At time of relapse	
Flow cytometry: peripheral blood sample[33]	X[34]			X[31]	X[31]				
Chest CT scan and Abdominal CT scan w/contrast	X[35]	When counts are consistent with CR or PR for 4 weeks; then 6 months later						At time of relapse	
Disease Assessments: MM Cohort ONLY									
Skeletal surveys[36]	X						X	X	
Extramedullary Disease Assessment[37]	X				X			X	
BM aspirate	X[38]				At Week 8 and at time of achieving CR or best response[39]			At Disease Progression	
BM biopsy	X[38]				At Week 8 and at time of achieving CR or best response[39]			At Disease Progression	
SPEP[40]	X			X		X		X	
UPEP[41,42]	X			X		X		X	

	Screening	Treatment[5]						Follow-up Visit[55]	Extended Follow-up[58]
		Day 1 [7]	Day 15 (±1 day)	Every 4 weeks (±3 days)	Every 8 weeks (±3 days)	Every 12 weeks (±3 days)	Every 16 weeks (±3 days)	Treatment or Study Withdrawal	Every 3 months (±14 days) *except when noted*
CRP, β2 microglobulin; IgG, IgA and IgM[43]	X			X		X		X	
Serum FLC assay[43]	X			X		X		X	
[redacted]									
Exploratory Assessments: All Cohorts									
Optional PGx: blood sample[46]		X							
Exploratory Assessments: ATC, ASI, BTC, GIST, NSGCT/NGGCT, and WHO Grade 1-4 Glioma Cohorts only									
Optional tumor tissue sample for biomarker analysis	X[47]		X[48]					At Disease Progression[48]	
Optional peripheral blood samples for biomarker analysis[49]	X		X					At Disease Progression	
Exploratory Assessments: HCL Cohort ONLY									
Optional Ex Vivo Assay[50]	X							X	
Exploratory Assessments: HCL and MM Cohorts ONLY									
Optional BM aspirate sample for biomarker analysis	X[51]		X[52]					At Disease Progression[52]	
Optional peripheral blood samples for biomarker analysis[53]	X		X					At Disease Progression	

		**Treatment[5]**						**Follow-up Visit[55]**	**Extended Follow-up[58]**
	**Screening**	**Day 1 [7]**	**Day 15 (±1 day)**	**Every 4 weeks (±3 days)**	**Every 8 weeks (±3 days)**	**Every 12 weeks (±3 days)**	**Every 16 weeks (±3 days)**	**Treatment or Study Withdrawal**	**Every 3 months (±14 days)** ***except when noted***
Study Treatments: ALL Cohorts									
Dispensation of study treatments		X[54]		X					
Dabrafenib Dosing		Continuous Twice Daily Dosing							
Trametinib Dosing		Continuous Once Daily Dosing							
Compliance Assessment				X				X	

Abbreviations: AFP, alpha- fetoprotein; ANC, absolute neutrophil count; AP, anteroposterior; ASI, adenocarcinoma of small intestine; β-HCG, beta-human chorionic gonadotropin; BM, bone marrow ; CBC, complete blood count; CR, complete response; CRP, C-reactive protein; CT, computed tomography; ECG, electrocardiogram; ECHO, echocardiogram; ECOG, Eastern Cooperative Oncology Group;[redacted]; FFPE, formalin-fixed, paraffin- embedded; FISH, fluorescence in situ hybridization; FLC, free light chain; H/E, Hematoxylin and eosin; hr(s), hour(s); ICF, informed consent form; IHC, immunohistochemistry; INR, international normalization ratio; [redacted]; PGx, pharmacogenetics; [redacted]; PT, prothrombin time; PTT, partial thromboplastin time; SPEP, serum protein electrophoresis; TSH, thyroid stimulating hormone; UPEP, urine protein electrophoresis

1. **ICF:** Informed consent should be obtained prior to performing any study-related procedure(s).
2. **MEDICAL HISTORY:** Medical History will include past and current medical conditions, including cardiovascular medical history and risk factors
3. **DISEASE CHARACTERISTICS:** Disease characteristics will include date of initial diagnosis, primary tumor type, histology, stage, etc.
4. **MUTATION TESTING:** Subjects **may** be enrolled based on local determination of BRAF V600E mutation status. A subject may be tested for the BRAF V600E mutation greater than 28 days prior to enrollment. (Any past results of local BRAF V600E mutation testing may be used to determine eligibility regardless of when the results were obtained.) To enroll a subject, the result of mutation testing from a sponsor designated central reference laboratory is not required; however, if the subject does not have a local result available, the sponsor designated central reference laboratory mutation test result will be required to determine eligibility for enrollment. **NOTE:** ***For subjects with a solid tumor:*** An archived or fresh tumor tissue sample must be available to send to a sponsor designated central reference laboratory for confirmation testing. **NOTE:** ***For subjects with HCL or MM:*** A fresh BM aspirate sample and a peripheral blood sample are required for confirmation testing by a sponsor designated central reference laboratory. If a BM aspirate cannot be obtained due to a dry tap, please contact the Medical Lead. **In the event of a dry tap, fresh or archived tumor tissues such as lymph node or plasmacytoma biopsies may be acceptable pending medical lead approval.** An archived tissue sample (such as FFPE tissue sample from a BM biopsy), BM core samples and BM clot samples cannot be used. The same BM aspirate sample may be used for BRAFmutation testing, the baseline assessment[redacted]
5. Dose interruptions should not alter the assessment schedule.

6. **SCREENING:** All screening assessments must be completed within 14 days prior to first dose except where indicated differently. Disease Assessment (if MRI is used) can occur within 35 days prior to the first dose. Note: Procedures conducted as part of the subject's routine clinical management (e.g., blood counts, imaging study) and obtained prior to signing of informed consent may be utilized for screening or baseline purposes provided these procedures meet the protocol requirements.
7. **DAY 1:** Day 1 is defined as the date of first dose of study treatments.
8. If assessment is completed within_3 days prior to the first dose of study treatments, the assessment does not need to be repeated on Day 1 unless clinically indicated.
9. **COMPLETE PHYSICAL EXAM:** A complete physical exam, including pelvic and rectal exams, will be performed at Screening and at the Follow-Up Visit (see Section 7.4.1).
10. **BRIEF PHYSICAL EXAM:** A brief physical exam will be performed on Day 1 (if more than 72 hrs since the complete physical exam was performed for Screening) and every 4 weeks while on study treatment.
11. **HEIGHT:** Height will be measured as part of complete physical exam performed at Screening only.
12. **FULL DERMATOLOGICAL EXAM:** A full dermatological exam will be performed at Screening (or within 35 days prior to the first dose) only. A biopsy in and/or around a new skin lesion(s) or a lesion(s) that changes during the study is required if clinically indicated (refer to Section 7.4.2).
13. **BRIEF DERMATOLOGICAL EXAM:** A brief dermatological exam will be performed every 4 weeks thereafter unless otherwise required to be performed more frequently. A brief dermatological exam should be performed every 4 weeks (±7 days) for the first 6 months after discontinuation of study treatments. A biopsy in and/or around a new skin lesion(s) or a lesion(s) that changes during the study is required if clinically indicated (refer to Section 7.4.2).
14. **OPHTHALMGIC EXAM:** Ophthalmic exams will be performed at Screening (or within 35 days prior to the first dose), Week 4, and as clinically indicated thereafter.
15. **VITAL SIGNS:** *Vital signs* include systolic/diastolic BP, temperature, pulse rate and respiratory rate. Vital signs should be measured in a semi-supine position after a 5 minute rest. *Blood pressure:* Three readings of BP and pulse rate should be taken. The first reading should be rejected and the second and third readings averaged to give the measurement to be recorded in the eCRF. If persistent hypertension (see Section 8.1.1.3.1) is noted in 3 consecutive visits, clinic visits to monitor the increased BP should be scheduled independently from the per-protocol visits. Ideally, subsequent BP assessments should be performed within 7 days of initial finding.
16. **ECG:** A single 12-lead ECG should be performed after vital signs and before blood draws if assessments are planned at the same nominal time point. If clinically significant abnormality(ies) are seen, confirm with 2 additional ECGs taken at least 5 minutes apart. See Section 7.4.6 and SPM for additional information on ECG collection.
17. **ECHO:** ECHOs will be performed at Screening (or within 35 days prior to the first dose), Week 4, Week 12, and then every 12 weeks thereafter. See Section 7.4.7 and SPM for additional information on ECHO collection.

[redacted]

19. **PREGNANCY TEST:** Perform only in female subjects of childbearing potential.
20. **PREGNANCY TEST:** A **serum** pregnancy test should be performed no more than 7 days prior to the first dose of study treatments.
21. **PREGNANCY TEST:** A **serum or urine** pregnancy test will be performed every 8 weeks during the first 48 weeks of study treatment, then every 12 weeks thereafter, and at the Follow-up Visit.
22. **MONITORING for NON-CUTANEOUS SECONDARY/RECURRENT MALIGNANCY:** During study treatment, monitor as clinically appropriate which may include a head/neck exam every 3 months and a chest/abdominal CT scan or MRI every 6 months.
23. **MONITORING for NON-CUTANEOUS SECONDARY/RECURRENT MALIGNANCY:** This assessment is to be part of the physical exam and should include visual inspection of the oral mucosa and lymph node palpation.
24. **MONITORING for NON-CUTANEOUS SECONDARY/RECURRENT MALIGNANCY:** Following discontinuation of study treatments, monitoring (which may include a chest/abdominal CT scan or MRI) should continue for up to 6 months or until initiation of new anti-cancer therapy.
25. **DISEASE ASSESSMENTS (Solid Tumor Cohorts only):** See Section 7.5.1 for details on baseline and post-baseline disease assessments for the solid tumor cohorts: ATC, ASI, BTC, GIST, NSGCT/NGGCT and WHO Grade 1-4Glioma.

26. **DISEASE ASSESSMENTS (Solid Tumor Cohorts only):** Baseline disease assessments must be performed within 28 days prior to enrollment (or within 35 days if MRI is used for disease assessment).
27. **DISEASE ASSESSMENTS (Solid Tumor Cohorts only):** Disease assessments are every 8 weeks during the first 48 weeks of study treatment, then every 12 weeks thereafter unless otherwise noted, and at the Follow-up Visit. Confirmatory scan(s) are to be performed ≥28 days after documented CR or PR. If subject discontinues treatment for reasons other than disease progression, disease assessments should continue every 12 weeks until disease progression, initiation of new anti-cancer treatment, subject withdrawal of consent, or death.
28. **HISTOLOGY CONFIRMATION: (ATC and WHO grade 1-4 cohorts only)** A tissue specimen for possible retrospective histology confirmation should be obtained and submitted to a Novartis designated central reference laboratory. Please refer to the SPM for the requirements of the tissue specimen that is required for the histology confirmation.
29. **NSGCT/NGGCT ONLY: AFP, B-HCG and LDH are the disease assessment markers for the NSGCT/NGGCT cohort**. LDH result obtained from clinical chemistry panel can be used as a tumor marker result. Regardless of baseline values of AFP, β-HCG and LDH, a blood sample will be drawn every 4 weeks for first 48 weeks of study treatment, then every 8 to 12 weeks thereafter, and at the time of disease progression. Synchronize the collection of the blood sample with the imaging schedule.
30. **BTC/ASI ONLY**: A blood sample will be drawn to assess CEA and CA 19-9 every 4 weeks for first 48 weeks of study treatment, then every 8 to 12 weeks thereafter, and at the time of disease progression. Synchronize the collection of the blood sample with the imaging schedule.
31. **HCL ONLY: All required disease assessments necessary for a post-baseline response determination should be performed every 4 weeks (+/- 3 days) for the first 48 weeks of study treatment. Beyond 48 weeks, patients tolerating study drug in the judgement of the treating investigator may transition to a response assessment evaluation interval of at least every 8 weeks (+/- 3 days). Patients that are not tolerating study drug in the judgement of the treating investigator must continue (or return to) response assessment evaluation every 4 weeks (+/- 3 days).** Assessments must be collected in agreement with those required in the response criteria (please consult Appendix 12)**.**
32. **HCL ONLY: BM biopsy and aspiration with H/E stain and IHC will be performed when blood counts are consistent with CR or PR for 4 weeks (please consult** Appendix 12**). If a patient has a PR, and then blood counts become consistent with a CR for 4 weeks, a repeat BM aspirate and biopsy is required to assess a response of CR. Following best response, a bone marrow biopsy and aspirate must be performed every 6 months for two time intervals, then after a year for two time intervals, then every two years.**
33. **HCL ONLY:** Recommend that flow cytometry panel consists of CD19, CD20, CD22, SmIg, CD11c, CD25, CD103 and CD123. **Flow cytometry of the bone marrow aspirate sample will be performed when blood counts are consistent with CR or PR for 4 weeks (please consult** Appendix 12). **If a patient has a PR, and then blood counts become consistent with a CR for 4 weeks, a repeat flow cytometry of the BM aspirate is required to assess a response of CR. Following best response, a flow cytometry of the bone marrow aspirate must be performed every 6 months for two time intervals, then after a year for two time intervals, then every two years.**
34. **HCL ONLY: BM biopsy and aspirate (with H/E stain and IHC), BM aspirate (with flow cytometry) and peripheral blood sample (with flow cytometry) will be assessed within 28 days prior to enrollment.**
35. **HCL ONLY:** A chest CT scan and an abdominal CT scan with contrast will be performed **within 28 days prior to enrollment**. If an abdominal CT scan with contrast cannot be performed, then a MRI may be performed **within 35 days prior to enrollment**. If lymph nodes or spleen are enlarged at baseline, then the abnormal organ needs to be followed on post-baseline assessments using the same modality of imaging.
36. **MM ONLY:** A skeletal survey should be performed within 28 days prior to enrollment. If myeloma associated bony lesions are present at baseline, skeletal surveys should be performed every 16 weeks or more frequently in order to assess response or upon clinical suspicion of progressive disease. Skeletal surveys typically consist of lateral radiographs of the skull, AP and lateral views of the spine, and AP views of the humeri, ribs, pelvis, and femurs.
37. **MM ONLY:** Each patient should be clinically examined for soft tissue plasmacytomas/extramedullary disease at baseline and at each visit for response assessment. If extramedullary disease is present at baseline, then Chest/Abdominal/Pelvis CT scan with contrast should be performed to confirm and to determine size and location of the soft tissue plasmacytoma.If no disease is identified at baseline, then subsequent scans are only required if clinically indicated. Alternative imaging modality (i.e., MRI) may be used if it is better to assess the lesion and approved by a Medical Lead. The same imaging modality should be used for all subsequent assessments.
38. **MM ONLY:** BM aspirate and BM biopsy samples will be assessed by IHC, flow cytometry, FISH and cytogenetics **within 90 days prior enrollment**.

39. **MM ONLY:** Flow cytometry for plasma cell enumeration and phenotyping will be performed on BM aspirate and BM biopsy samples at Week 8 and then at the time of achieving a CR or best response. **NOTE:** Density gradient is not recommended.
40. **MM ONLY:** SPEP should be performed **within 28 days prior to enrollment**, every 4 weeks for the first 48 weeks of study treatment, then every 12 weeks thereafter, and at the Follow-up Visit.
41. **MM ONLY:** UPEP must be performed using a 24-hr urine sample.
42. **MM ONLY:** If UPEP is positive at Screening (**or within 28 days prior to enrollment**), then a UPEP should be performed every 4 weeks for first 48 weeks of study treatment, then every 12 weeks thereafter, and at the Follow-up Visit.
43. **MM ONLY:** CRP, β2 microglobulin; immunoglobulins (IgG, IgA and IgM) and serum FLC assay should be performed **within 28 days prior to ernollment**, every 4 weeks for the first 48 weeks of study treatment, then every 12 weeks thereafter, and at the Follow-up Visit.

[redacted]

46. **PGx:** (Optional) If informed consent for PGx research has been obtained, a 6 mL blood sample for PGx analysis will be collected and sent for central testing on or after Day 1.
47. **PD Biomarkers** (Optional): *For ACT, ASI, BTC, GIST, NSGCT/NGGCT and WHO Grade 1- 4 Glioma:* A pre-dose FFPE tumor tissue sample is to be obtained during Screening if consent is provided. If a fresh FFPE tumor tissue for BRAF mutation testing is obtained within 28 days of Screening, it can also be used for this pre-dose sample.
48. **PD Biomarkers** (Optional): *For ACT, ASI, BTC, GIST, NSGCT/NGGCT, and WHO Grade 1-4 Glioma:* On Day 15 (±3 days) and at the time of disease progression, a post-dose FFPE tumor tissue sample will be collected if a pre-dose tumor biopsy was obtained during Screening.
49. **PD Biomarkers** (Optional): *For ACT, ASI, BTC, GIST, NSGCT/NGGCT, and WHO Grade 1-4 Glioma:* If a tumor biopsy is performed at a given time point (see Footnotes 47 and 48), a 7.5 mL peripheral blood sample for cfDNA as well as a 10mL peripheral blood sample for PD plasma and PBMC will be collected at the corresponding time point: Screening, on Day 15 (±3 days), and at the time of disease progression.
50. **Exploratory Assessments** (Optional): *For HCL Cohort only:* Blood samples (approximately 25 mL) will be collected from HCL subjects (see Section 13.2 for further details) **at the corresponding time point: Screening and at the Follow-up Visit**. The investigator should use their clinical judgment in determining whether it is appropriate for a subject enrolled in the HCL cohort to have this additional blood drawn. In addition, if the IEC or IRB does not approve the site for participation in the sub-study, no subjects at those sites will be included in the sub-study.
51. **PD Biomarkers** (Optional): *For HCL and MM Cohorts:* A pre-dose BM aspirate sample is to be obtained during Screening if consent is provided. If a fresh BM aspirate sample was taken for BRAF mutation testing within 28 days of Screening, it can be used for this pre-dose sample.
52. **PD Biomarkers** (Optional): *For HCL and MM Cohorts*: On Day 15 (±3 days) and at the time of disease progression, a post-dose BM aspirate sample will be collected if a pre- dose BM aspirate sample was obtained duringScreening.
53. **PD Biomarkers** (Optional): *For HCL and MM Cohorts:* If a BM aspirate is performed at a given time point (see Footnote 51 and Footnote 52), a single 7.5 mL peripheral blood sample will be collected at the corresponding time point: Screening, on Day 15 (±3 days), and at the time of disease progression.

54. **STUDY TREATMENT:** Subjects must receive their supply of study treatments within 3 days of enrollment.
55. **FOLLOW-UP VISIT:** The Follow-up Visit should take place within 28 days (+7 days) of the last dose of study treatments. If the subject is unable to return to clinic, site staff is encouraged to call subject for assessment of AEs.
56. **FOLLOW-UP VISIT:** All safety and laboratory assessments should be performed unless the assessments were performed within 4 weeks prior to the Follow-up Visit.
57. **FOLLOW-UP VISIT**: If the last cardiac assessment was >12 weeks prior to the Follow-up Visit, an ECHO should be performed.
58. **EXTENDED FOLLOW-UP:** The following evaluations will be performed for all patients in all cohorts.
    a) Dermatologic exam. Exams will be performed every 4 weeks (+/-7 days) for the first 6 months. The skin will be carefully examined and pictures and biopsy will be taken of any new or changed skin lesions.
    b) If study treatments are permanently discontinued prior to the subject having confirmed disease progression, the subject will continue to have disease assessments every 12 weeks (+/-14 days) until disease progression or initiation of new anti-cancer treatment, or death, whichever comes first.
    c) Subjects will be followed every 12 weeks (+/-14 days) for survival until the subject dies or withdraws consent or is lost to follow up, or up to a time period as defined in Section 16.3. Survival follow up may be performed via clinic visit, a telephone contact, an email or by mail. The initiation of any new anti-cancer treatment(s) and date of last contact should also be documented.
    d) Subjects will have a head and neck exam performed every 12 weeks for the first 6 months to monitor for secondary or recurrent malignancy.
    e) Subjects may have CT and/or MRI scans of the chest and abdomen every 6 months to monitor for secondary or recurrent malignancy.

7.2 Demographic/Medical History Assessments

The following demographic parameters will be captured: date of birth, gender, race and ethnicity. Medical/medication history will also be assessed as related to the eligibility criteria.

7.3 Critical Baseline Assessments

7.3.1 Baseline Confirmation of BRAF-Mutation Status

In order to be eligible for the study, subjects must also have archived tumor sample material available or be willing to undergo a biopsy in order to meet eligibility for the study.

If multiple results are available, eligibility should be based on the most recent test result. If the subsequent central laboratory testing is negative and, in the opinion of the investigator, the subject is deriving benefit, the subject may continue in the study.

For Solid Tumor Histologies:

For solid tumor histologies, archived tumor tissue sample or fresh tumor tissue sample obtained by biopsy must be submitted to the sponsor designated central reference laboratory for central confirmatory testing of the BRAF V600E mutation.

If sufficient archived tumor tissue is not available for central confirmation of BRAF mutation status and the subject cannot undergo a repeat biopsy to obtain an additional tumor tissue sample(s), the subject is NOT eligible for the study.

Table 5 BRAF V600E Mutation Testing Scenarios for Solid Tumors

	Scenario		Decision		
	**Local BRAF V600E MutationTest Performed? Result?**	**Archived Tumor Tissue Sample?**	**Tumor Sample to be Obtained**	**Eligible for Enrollment?**	**BRAF V600E Mutation Test to be Performed?**
**1**	Yes. Positive	Not available	Fresh tumor tissue obtained by biopsy	Yes, only after submission of fresh tumor tissue sample for central testing	Yes, Central
**2**	Yes. Positive	Available	Archived tumor tissue	Yes, only after submission of archived tumor tissue sample for central testing	Yes, Central
**3**	No. No result.	Not available	Fresh tumor tissue obtained by biopsy	Yes, only If central test result on fresh tumor tissue is positive for a BRAF V600E mutation	Yes, Central
**4**	No. No result.	Available	Archived tumor tissue	Yes, only if central test result on archived tumor tissue is positive for a BRAF V600E mutation.	Yes, Central
**5**	Yes. Negative	Subject is not eligible for participation.			

For Hematologic Histologies:

For the HCL and MM cohorts, a fresh BM aspirate sample and a corresponding peripheral blood sample are required.

NOTE: For subjects in the HCL and MM cohorts, an archived tissue sample (such as formalin-fixed paraffin-embedded [FFPE] tissue sample from a BM biopsy), BM core samples and BM clot samples are NOT acceptable for BRAF V600E testing. If a BM aspirate cannot be obtained due to a dry tap, please contact the Medical Lead.

Details on sample collection, processing, storage and shipping procedures are provided in the lab manual.

7.3.2 Baseline Documentation of Target and Non-Target Lesions for Solid Tumor Histologies

All baseline lesion assessments must be performed within 28 days of enrollment **except where indicated differently in the T&E table (Section 7.1)**. Guidance on baseline documentation of target and non-target lesions will be provided in the SPM.

7.4 Safety

Safety assessments and procedures, including the timing of the assessments, are summarized in the Time and Events Table (Section 7.1). Additional time points for safety tests (such as vital signs, physical examinations, and laboratory safety tests) may be added during the course of the study based on newly available data to ensure appropriate safety monitoring.

7.4.1 Physical Examinations

Complete Physical Examination: A complete physical examination will be performed by a qualified physician, physician assistant or nurse practitioner under the supervision of a qualified physician, or another designee as permitted by local practices or laws. At minimum, the examination should include assessments of the head and neck, eyes, ears, nose, throat, skin, thyroid, CNS, lungs, cardiovascular system, abdomen (liver and spleen), lymph nodes, extremities and genitalia. Height (measured only at Screening) and weight will be measured and recorded. Complete physical examinations will be performed at Screening and at the post-treatment follow-up visit.

Complete physical examinations will also include thorough rectal and genitourinary (pelvic) examinations to assess secondary malignancies.

- **Rectal Examinations:** Including digital rectal examination and visual inspection of the anus and perianal area.
- **Pelvic Examinations (for female subjects only):** Including visualization of cervix. At Screening, pelvic examinations may be completed up to 24 weeks prior to date of first dose; thus, a subject’s most recent gynecological examination may be used to satisfy the screening requirements. (Pap smear and/or colposcopy are not required unless clinically indicated.)
- **Brief Physical Examination:** A brief physical examination will include assessments of the lymph nodes, skin, lungs, cardiovascular system and abdomen (including liver and spleen assessment). Weight will be measured and recorded. Brief physical examination will be performed on Day 1 and every 4 weeks while on study treatment.

7.4.2 Dermatological Examinations

Dermatological examinations will be performed by an investigator or the subject may be referred to a dermatologist, at the discretion of the investigator. **A complete or full body**

dermatological examination will be performed at the time points indicated in the Time and Event Table (Section 7.1) to identify any abnormal skin lesions. All abnormal findings (i.e., suspicious for proliferative skin lesions) will be identified and photographed during the Screening examination. Brief dermatological examinations should be performed every 4weeks or more frequently if clinically indicated while on study treatment and every 4 weeks (±7 days) for the first 6 months following treatment discontinuation. Wherever possible, the same individual should perform all of the examinations. Follow-up skin examinations by a referral dermatologist should be conducted if clinically indicated.

Biopsy in or around new skin lesions or lesions that change during the study is required if clinically indicated. Testing of these biopsy specimens may include analysis of proteins related to the action of dabrafenib. DNA analysis of these biopsy specimens may be performed, and would be restricted to the analysis of pathway mutations known to be associated with, and relevant to, BRAF mutant tumors. A summary of the results should be submitted to the sponsor.

Skin photography of new lesions or lesions that change during the study must be obtained and forwarded to the sponsor. Refer to the SPM for details regarding skin lesion documentation by photography.

7.4.3 Ophthalmic Examinations

Subjects are required to have a standard ophthalmology exam performed by an ophthalmologist at the time points indicated in the Time and Event Table (Section 7.1). The exam will include best corrected visual acuity, tonometry, slit lamp biomicroscopic examination, visual field examination, and dilated indirect fundoscopy with special attention to retinal abnormalities. Optical coherence tomography is strongly recommended at scheduled visits, and if retinal abnormalities are suspected. Other types of ancillary testing including color fundus photography and fluorescein angiography are also recommended if clinically indicated. Refer to Section 8.2.7 for additional details on management of visual changes.

7.4.4 Vital Signs

Vital sign measurements will include systolic and diastolic BP, temperature, pulse rate and respiratory rate. Vital signs will be measured at the time points indicated in the Time and Event Table (Section 7.1). Vital signs may be measured more frequently if warranted by the clinical condition of the subject.

- Vital sign measurements to be measured in semi-supine position after 5 minutes rest
- Three readings of BP and pulse rate should be taken. The first reading should be rejected and the second and third readings averaged to give the measurement to be recorded in the eCRF.

Refer to Section 8.1.1.3.1 and Section 8.1.1.3.2 for guidance on the monitoring and management of hypertension.

If subject develops a fever, refer to Section 8.2.3 for management guidelines for pyrexia. Refer to the SPM for details regarding measurement of vital signs.

7.4.5 Performance Status

The performance status will be assessed using the ECOG performance status scale (Appendix 1).

7.4.6 Electrocardiogram

Electrocardiogram (12-Lead ECGs) will be obtained using an ECG machine that automatically calculates the heart rate and measures PR, QRS, QT, and QTc intervals. Results of the ECG will be transmitted to a central storage facility. At each assessment, a 12-lead ECG will be performed by qualified site personnel after the subject has rested at least 5 minutes in a semi-recumbent or supine position.

Those QTc values >480 msec as calculated by the machine must be confirmed manually using Bazett's formula given below:

$$\mathbf{QTcB = QT \times (1/\sqrt{RR})}$$

If there are any clinically significant abnormalities including, but not limited to, a QTcB >500 msec, confirm with two additional ECGs taken at least 5 minutes apart.

Refer to Section 8.1.1.1 for QTc withdrawal criteria and additional QTc readings that may be necessary.

7.4.7 Echocardiogram

Echocardiograms (ECHOs) will be performed to assess cardiac ejection fraction andcardiac valve abnormalities. **ECHOs will be performed at the time points indicated in the Time and Event Table (Section 7.1)**. The evaluation by the echocardiographer should include an evaluation for LVEF and both right and left-sided valvular lesions.

The procedure (ECHO) to document the subject's baseline LVEF status must be used consistently throughout the study. If possible, it is also preferred that interpretation of LVEF status be performed consistently by the same reviewer throughout the study. Copies of all ECHOs performed on ALL subjects will be required by the sponsor for review. Instructions for submission of ECHOs are provided in the SPM.

7.4.8 Clinical Laboratory Assessments

General laboratory assessments and histology-specific laboratory assessments will be required during the study and should be performed by a local laboratory. Laboratory tests may be done 7 days prior to the scheduled visit in order to have results available for review by investigator prior to the scheduled visit date.

Urine tests for protein/creatinine may be done up to 3 days prior to the scheduled visit date. If a 24-hr urine collection for protein has been completed within 5 days prior to scheduled visit date, a urine sample for protein/creatinine is not necessary.

If additional non-protocol specified laboratory assessments are performed at the institution's local laboratory and result in a change in subject management or are considered clinical significant by the investigator (i.e., AE, SAE or dose modification) the results must be recorded in the subject's eCRF.

All laboratory tests with values that are significantly abnormal during study participation or within 28 days after the last dose of study treatment(s) should be repeated until the values

return to within normal range or baseline. All subjects who have a Grade 3 or 4 laboratory abnormality at time of study withdrawal must be followed until resolution to Grade 2 or less, unless it is unlikely to improve due to underlying disease. If such values do not return to within normal range within a period judged reasonable by the investigator, the etiology should be identified and the Medical Lead notified.

Laboratory requisition forms must be completed and samples must be clearly labeled with the subject number, protocol number, site/center number, and visit date. Refer to the lab manual for appropriate processing and handling of samples to avoid duplicate and/or additional blood draws.

Refer to Appendix 17 for an estimation of the volume of blood samples collected during the study.

Table 6 Clinical Laboratory Assessments

Hematology

**RBC Count**	**RBC Indices (at Screening and if hemoglobin decreases**	**Automated WBC Differential:**
Platelets	MCV	Lymphocytes
WBC Count	MCH	Monocytes
Hemoglobin	MCHC	Eosinophils
Hematocrit	Reticulocyte Count	Basophils
Absolute neutrophil count (ANC)		

Clinical Chemistry

BUN	Potassium	Total Protein	Total and direct bilirubin[1]
Creatinine[3]	Chloride[2]	Phosphorus	AST
Glucose	Uric Acid[2]	Magnesium	ALT
Sodium	Calcium	LDH	Alkaline Phosphatase
Albumin	HbA1c[6]	**GGT**	

Coagulation

PT
PTT
INR

Urinalysis[4]

Color	Specific gravity	Ketones	Urobilinogen
Appearance	Glucose	Blood	
pH	Protein[5]	Leukocyte esterase	

Other Tests

Amylase and lipase [monitor via local laboratory when clinically indicated to evaluate certain AEs (i.e., abdominal pain, pancreatitis, etc.)
Serum or urine β-hCG[6]
For subjects with a history of chronic HBV and/or HCV, the following tests will be performed at Screening:<br>● Viral hepatitis serology;<br>● Hepatitis B surface antigen and Hepatitis B core antibody (IgM); and/or<br>● Hepatitis C RNA

Abbreviations: ALT, alanine aminotransferase; ANC, absolute neutrophil count; AST, aspartate aminotransferase; β- hCG, Beta-human chorionic gonadotropin; BUN, blood urea nitrogen; GGT, gamma glutamyl transferase; INR, international normalization ratio; HbA1C, hemoglobin A1C; HBV,

hepatitis B virus; HCV, hepatitis C virus; LDH, lactate dehydrogenase; MCV, mean corpuscular volume; MCH, mean corpuscular hemoglobin; MCHC, mean corpuscular hemoglobulin concentration; PT, prothrombin time; PTT, partial thromboplastin time; RBC, red blood cells; TSH, thyroid stimulating hormone; ULN, upper limit of normal; WBC, white blood cells

1. Direct bilirubin is required only if the total bilirubin is elevated (≥2 times the ULN)
2. Chemistry evaluation of chloride or uric acid is not required where there are logistical constraints.
3. If serum creatinine is >2.0 mg/dL, calculate creatinine clearance using standard Cockcroft-Gault method (Appendix 3).
4. If urine protein result is not available, urine albumin result may be used.
5. For female subjects of childbearing potential only, unless subject has had a hysterectomy, undergone a tubal ligation within 1 year prior to Screening or has been post-menopausal for at least 1 year prior to Screening.

HbA1c testing to be performed every 12 weeks (+/-3 days)

7.4.8.1 Additional Histology Specific Assessments and Histology Confirmation

For ATC Cohort ONLY: Thyroid stimulating hormone (TSH) and Free T4: Serum TSH suppression (lowering TSH below the LLN) through the use of thyroid hormone replacement (e.g., levothyroxine) should be considered prior to the first dose of study treatment and throughout the study. However, the decision to suppress TSH is optional and dependent upon the investigator weighing the risk and benefits for each individual subject. If the investigator is unsure about suppressing the TSH, it is recommended that an endocrinologist be consulted. Free T4 will also be evaluated.

For NSGCT/NGGCT Cohort ONLY: β-HCG and AFP: These laboratory assessments will be completed as indicated in the Time and Event Table (Section 7.1).

For BTC and ASI Cohorts ONLY: CEA and CA19-9: These laboratory assessments will be completed as indicated in the Time and Event Table (Section 7.1).

Histology Confirmation for ATC and WHO Grade 1-4 Glioma Cohorts ONLY:

A tissue specimen from the biopsy of a primary or metastatic site must be submitted to a Novartis designated central reference laboratory as soon as possible after enrollment for possible retrospective confirmation of histologic type. Refer to the SPM and the Lab Manual for details regarding sample submission.

7.4.9 Monitoring for Non-Cutaneous Secondary/Recurrent Malignancy

Prior to initiation of treatment subjects should undergo a head and neck examination with minimally visual inspection of oral mucosa and lymph node palpitation, as well as chest/abdomen CT scan. Subjects should be monitored as clinically indicated during treatment and for up to 6 months after treatment discontinuation or until a new anti- cancer therapy is initiated. Anal examinations and pelvic examinations (for women) are required before and at the end of treatment or when considered clinically indicated. Complete blood cell counts should be performed as clinically indicated. Abnormal findings should be managed according to standard clinical practice.

Any new non-cutaneous secondary/recurrent malignancy should be reported as an SAE and treated according to standard clinical practice.

7.4.10 Pregnancy Testing and Reporting

7.4.10.1 Pregnancy Testing

The need for a screening pregnancy test depends on whether a female subject is of childbearing or non-childbearing potential.

A female of non-childbearing potential (i.e., physiologically incapable of becoming pregnant) is defined as any female who has had a hysterectomy, bilateral oophorectomy (ovariectomy) or bilateral tubal ligation, or is post-menopausal defined as 1 year without menses with an appropriate clinical profile, e.g., age appropriate, >45 years in the absence of hormone replacement therapy (HRT). In questionable cases, the subject must have a follicle stimulating hormone (FSH) value >40 mIU/mL and an estradiol value <40 pg/mL (<140 pmol/L).

A female of child-bearing potential is defined as any female who does not meet the criteria of non-childbearing potential as described in the previous paragraph.

If a female subject is of childbearing potential, she must have a serum β-HCG pregnancy test performed within 7 days prior to the first dose of study treatment. Subjects with a positive pregnancy test result must be excluded from the study. Subjects with a negative pregnancy test result must agree to use an effective contraception method (see Section 9.1) during the study and for 16 weeks following discontinuation of trametinib when taken in combination with dabrafenib, or for 2 weeks following discontinuation of dabrafenib monotherapy.

7.4.10.2 Pregnancy Reporting

Any pregnancy that occurs during study participation must be reported using a clinical trial pregnancy form. To ensure subject safety, each pregnancy must be reported to the sponsor within 24 hours of learning of its occurrence. The pregnancy must be followed up to determine outcome (including premature termination) and status of mother and child. Pregnancy complications and elective terminations for medical reasons must be reported as an AE or SAE. Spontaneous abortions must be reported as an SAE. Any SAE occurring in association with a pregnancy brought to the investigator's attention after the subject has completed the study and considered by the investigator as possibly related to the study treatment, must be promptly reported to the sponsor.

7.4.10.3 Time period for collecting pregnancy information

All pregnancies in female subjects and/or female partners of male subject will be collected after the start of dosing until the follow-up visit.

7.4.10.4 Action to be taken if pregnancy occurs in a female partner of a male study subject

The investigator will attempt to collect pregnancy information on any female partner of a male study subject who becomes pregnant while participating in this study. The investigator will record pregnancy information on the appropriate form and submit it to the sponsor within 24 hours of learning of the partner's pregnancy. The partner will also be followed to determine the outcome of the pregnancy. Information on the status of the mother and child will be forwarded to the sponsor. Generally, follow-up will be no longer than 6 to 8 weeks following the estimated delivery date. Any premature termination of the pregnancy will be reported.

7.5 Disease Assessment

If CT scan with contrast is contraindicated (except for chest CT scan), a MRI may be used as an alternative method of baseline disease assessment. If a chest CT scan with contrast is contraindicated, a chest CT scan without contrast should be performed.

The method used to document baseline status should be used consistently throughout study to facilitate comparison of scan results. Assessments must be performed on a calendar schedule and should not be affected by dose interruptions or delays. Refer to the Time and Events Table (Section 7.1) for the schedule of disease assessments.

7.5.1 Disease Assessment for Solid Tumor Histologies

Disease assessment for solid tumor types (ATC, BTC, GIST, NSGCT/NGGCT, WHO Grade 1 and 2 gliomas, WHO Grade 3 and 4 gliomas and ASI) will be conducted as noted below. For subjects whose disease may be followed by well-characterized tumor markers, disease assessment should also include results of tumor marker measurements.

Disease assessments will be completed as indicated in the Time and Events Table (Section 7.1).

Please refer to the IAG for additional details on imaging assessments for this study.

Baseline Assessments: The following baseline disease assessments are required for all subjects within 28 days prior to enrollment (or within 35 days if MRI is used for disease assessment:

Histology	Area(s) of Assessment	Imaging to be Performed	Alternative Imaging Methods if Contraindications are Present
ATC[1]	Brain	MRI with contrast	1st alternative: MRI without contrast<br>2nd alternative: CT scan with contrast
	Neck	CT scan with contrast	1st alternative: a MRI with contrast 2nd alternative: CT scan without contrast (if 1st alternative is contraindicated)
	Chest/Abdomen (including liver and adrenal glands)	CT scan[2]	
BTC/ASI	Chest/Abdomen/Pelvis	CT scan with contrast	CT scan without contrast of the Chest and MRI with contrast of Abdomen/Pelvis
NSGCT/NGGCT	Chest/Abdomen/Pelvis	CT scan with contrast	CT scan without contrast of the Chest and MRI with contrast of Abdomen/Pelvis
GIST	Chest/Abdomen/Pelvis	CT scan with contrast	CT scan without contrast of the Chest and MRI with contrast of Abdomen/Pelvis
ATC[1] BTC GIST NSGCT/NGGCT ASI WHO Grade 1-4 Glioma	Regions with known or suspected metastatic lesions	CT scan, MRI, or bone scan as appropriate	
WHO Grade 1- 4 Glioma	Brain	MRI with contrast[3]	N/A
NSGCT/NGGCT[4]	Brain (for pituitary GCT)	MRI with contrast	1st alternative: MRI without contrast<br>2nd alternative: CT scan with contrast

Abbreviations: ASI, adenocarcinoma of small intestine; ATC, anaplastic thyroid carcinoma; BTC, biliary tract carcinoma; CNS, central nervous system; CR, complete response; CT, computed tomography; GCT, germ cell tumor; GIST,

gastrointestinal stromal tumor; MRI, magnetic resonance imaging; NGGCT, non-geminomatous germ cell tumor; NSGCT, non-seminomatous germ cell tumor; PET, positron emission tomography; SPM, Study Procedures Manual; WHO, World Health Organization

1. For ATC subjects without CNS and neck disease at baseline, subsequent brain and neck imaging should only be performed as clinically indicated.
2. Optional: Whole body PET/CT scan
3. MRI of the brain with contrast is mandated in this cohort; CT scan is not permitted. Please refer to imaging details in the SPM.
4. For NSGCT/NGGCT subjects *without* CNS disease at baseline, subsequent MRI of the brain with contrast should only be performed as clinically indicated (e.g., symptoms suggestive of CNS progression). For subjects with CNS disease at baseline, a MRI of the brain with contrast is required every 4 to 8 weeks or as clinically indicated. In addition, in order to confirm a CR in a subject with brain disease at baseline, a MRI of the brain with contrast must be performed 1 week prior to the first set of images showing CR to 4 weeks after the next protocol specified assessment.

Post-Baseline Assessments: All post-baseline assessments require imaging of disease sites identified by baseline scans. The same imaging modality used at baseline should be utilized for all post-baseline assessments. If subject discontinues treatment prior to Week 48 for reasons other than disease progression, disease assessments (imaging) should continue every 12 weeks until a new treatment is initiated.

Histology	Area(s) of Assessment	Imaging to be Performed	Alternative Imaging Methods if Contraindications are Present
ATC	Chest/Abdomen (including liver and adrenal glands)	CT scan[1]	
BTC, ASI	Chest/Abdomen/Pelvis	CT scan with contrast	CT scan without contrast of the Chest and MRI with contrast of Abdomen/Pelvis
WHO Grade 1-4 Glioma	Brain	MRI with contrast[2]	N/A
NSGCT/NGGCT, GIST	Chest/Abdomen/Pelvis	CT scan with contrast	CT scan without contrast of the Chest and MRI with contrast of Abdomen/Pelvis

Abbreviations: ASI, adenocarcinoma of small intestine; ATC, anaplastic thyroid carcinoma; BTC, biliary tract carcinoma; CT, computed tomography; GIST, gastrointestinal stromal tumor; MRI, magnetic resonance imaging; NGGCT, non-geminomatous germ cell tumor; NSGCT, non-seminomatous germ cell tumor; SPM, Study Procedures Manual; WHO, World Health Organization

1. Optional: Whole body PET/CT scan
2. MRI with contrast is mandated in this cohort; CT scan is not permitted. Please refer to imaging details in SPM.

7.5.1.1 Response Criteria for Solid Tumor Histologies

For solid tumor types (ATC, BTC, GIST, NSGCT/NGGCT, WHO Grade 1 and 2 gliomas, WHO Grade 3 and 4 gliomas and ASI), response evaluations will be determined according to the response assessment criteria listed in Table 7.

Table 7 Response Criteria for Solid Tumor Histologies

Histology	Response Criteria	Response Subcategories defined as 'Response[4]
ATC, BTC, GIST, ASI, NSGCT/NGGCT	RECIST v 1.1[1]	CR, PR
WHO Grade 1 and 2 Glioma	RANO Response Criteria: Response Assessment Criteria for WHO Grade 1 and 2 Glioma[2]	CR, PR and MR

WHO Grade 3 and 4 Glioma	RANO Response Criteria: Updated Response Assessment Criteria for WHO Grade 3 and 4 Glioma[3]	CR, PR

Abbreviations: ATC, anaplastic thyroid carcinoma; BTC, biliary tract cancer; CR, complete response; GIST, gastrointestinal stromal tumor; MR, minor response; NGGCT, non-geminomatous germ cell tumor; NSGCT, non- seminomatous germ cell tumor; PR, partial response; RANO, Response Assessment in Neuro-Oncology; RECIST, Response Evaluation Criteria in Solid Tumors; SPM, Study Procedures Manual; WHO, World Health Organization

1. Refer to Appendix 10 for details responsecriteria.
2. Refer to Appendix 13 for details response criteria
3. Refer to Appendix 14 for details response criteria
4. Serum tumor markers normalization will not be considered as part of response criteria.

7.5.2 Disease Assessment for Histologies of Hematologic Malignancies

For hematologic malignancies (HCL and MM), disease assessment will be conducted as noted below. For subjects whose disease may be followed by well-characterized tumor markers, disease assessment should also include results of tumor marker measurements. Disease assessments will be completed as indicated in the Time and Events Table (Section 7.1).

7.5.2.1 Laboratory and Disease Assessments: HCL

Baseline Assessments: The following baseline disease assessments will be performed at the time points indicated in the Time and Event Table (Section 7.1):

- Complete blood count
- Routine stain of peripheral blood sample
- **BM aspirate** and biopsy sample with H/E stain and IHC
- Flow cytometry on BM aspirate sample and peripheral blood sample
- Chest CT scan and abdominal CT scan with contrast

NOTE: MRI of the abdomen may be performed if abdominal CT cannot be performed. The method used to document baseline status is used consistently throughout the study to facilitate comparison of results.

If lymph nodes and spleen were enlarged at baseline, they must be followed by post- baseline assessments using a consistent imaging modality.

Post-Baseline Assessments: A response is to be determined every 4 weeks (+/-3 days) for the first 48 weeks of study treatment. Beyond 48 weeks, patients tolerating study drug in the judgement of the treating investigator may transition to a response assessment evaluation interval of at least every 8 weeks (+/-3 days). Patients that are not tolerating study drug in the judgement of the treating investigator must continue (or return to) response assessment evaluation every 4 weeks (+/-3 days). Assessments must be collected in agreement with those required in the response criteria (please consult Appendix 12).

- Complete blood count
- Routine stain of peripheral blood sample
- BM aspirate and biopsy sample with H/E stain and IHC
- Flow cytometry on BM aspirate sample will be performed **as indicated in the Time and Event Table (Section 7.1).**

- Flow cytometry on peripheral blood sample will be performed as indicated in the Time and Event Table (Section 7.1)
- If lymph nodes and/or spleen were enlarged at baseline, they must be followed by post-baseline assessments using a consistent imaging modality as indicated in the Time and Event Table (Section 7.1).

7.5.2.2 Laboratory and Disease Assessment: MM

Baseline Assessments: The following baseline disease assessments will be performed at the time points indicated in the Time and Event Table (Section 7.1):

- Skeletal surveys
- Extramedullary disease assessment BM aspirate and biopsy samples: Samples will be assessed by IHC, flow cytometry, fluorescence *in situ* hybridization (FISH) and cytogenetics.
- UPEP
- SPEP
- CRP, β-2 microglobulin, serum FLC assay, levels of immunoglobulin (Ig)G, IgA, and IgM

Post-baseline Assessments: All post-baseline assessments require imaging of disease sites identified by baseline scans. The same imaging modality used at baseline should be utilized for all post-baseline assessments.

- BM aspirate and biopsy samples will be performed and assessed as indicated in the Time and Event Table (Section 7.1)
- Laboratory tests (SPEP, UPEP , CRP, β-2 microglobulin, serum FLC assay, levels of IgG, IgA, and IgM will be performed as indicated in the Time and Event Table (Section 7.1).
- Skeletal surveys: Post-baseline assessments only need to be performed if disease is noted on the baseline assessment. If no disease is identified at baseline, then subsequent skeletal surveys are only required if clinically indicated.
- Extramedullary disease assessment: Post-baseline assessments only need to be performed if disease is noted on the baseline assessment. If no disease is identified at baseline, then subsequent assessments are only required if clinically indicated.

7.5.2.3 Response Criteria for Hematologic Histologies

For hematologic malignancies (HCL and MM), response evaluations will be determined according to the response assessment criteria listed in Table 8.

Table 8 Response Criteria for Hematologic Histologies

Histology	Response Criteria	Response Subcategories defined as 'Response'
HCL	Adapted from NCCN guidelines, Consensus Resolution Criteria and previous studies definition[1]	CR±MRD, PR
MM	IMWG Uniform Response Criteria for MM[2]	sCR, CR, PR, VGPR

Abbreviations: CR, complete response; HCL, hairy cell leukemia; IMWG, International Myeloma Working Group; MM, multiple myeloma; MRD, minimal residual disease; NCCN, National Comprehensive Cancer Network; PR, partial response; sCR, stringent complete response; SPM, Study Procedures Manual; VGPR, very good partial response

1. Refer to Appendix 12 for details on response criteria.
2. Refer to Appendix 15 for details on response criteria.

8 DOSE MODIFICATIONS AND STOPPING CRITERIA

8.1 Dose Modification

General guidelines regarding management and dose reduction for AEs that are considered by the investigator to be related to study treatment and for which specific guidelines do not apply are provided in Table 11. A maximum of two trametinib dose level reductions are allowed. If a third dose level reduction is required, study treatment should be permanently discontinued.

If a dose reduction of trametinib is required, but the toxicity resolves and no additional toxicities are seen after **one** treatment cycle (**4 weeks**), the dose of trametinib may be re-escalated but should not exceed 2 mg once daily.

If a dose reduction of dabrafenib is required, but the toxicity resolves and no additional toxicities are seen after one treatment cycle (4 weeks), the dose of dabrafenib may be re-escalated but should not exceed 150 mg BID daily.

These guidelines are intended primarily for toxicities not easily managed with routine supportive care. For example, alopecia is not an indication for dose modification, nor is Grade 2 nausea and vomiting that can be easily managed with anti-emetics.

These are general guidelines and investigators should always use clinical judgment in determining dose adjustments for any individual subject. Some toxicities may require hospitalization for stabilization, additional work-up, and consultation with a specialist before treatment can be restarted. Investigators should always err on the side of caution in these settings if treatment-related toxicity is a possibility.

Dose delay: Treatment with dabrafenib and trametinib may be delayed for up to 21 days to allow resolution of toxicity, or based on investigator discretion (e.g., scheduling issues). If the investigator and the Medical Lead conclude that continued treatment will benefit a subject who has experienced a treatment delay >21 days, then the subject may continue treatment with the approval of the Medical Lead. If a dose delay continues for 60 or more days, then both study treatments (dabrafenib and trametinib) will be permanently discontinued. Subjects should complete the Follow-Up Visit and then proceed to the extended follow-up phase as indicated in the Time and Events Table (Section7.1).

Dose reduction: A dose reduction below 75 mg BID for dabrafenib or below 1 mg once daily for trametinib is not allowed. If a dose reduction below 75 mg BID for dabrafenib is required, dabrafenib will be permanently discontinued, but the subjects will be allowed to continue treatment with trametinib. If a dose reduction below 1 mg once daily for trametinib is required, then trametinib will be permanently discontinued, but these subjects will be allowed to continue treatment with dabrafenib.

If both study treatments are reduced below the minimum dose levels (75 mg BID for dabrafenib and 1 mg once daily for trametinib), then both study treatments will be permanently discontinued. Subjects should complete the Follow-Up Visit and then proceed to the extended follow-up phase as indicated in the Time and Events Table (Section 7.1).

For Grade 2 or 3 laboratory abnormalities that are considered unrelated to the study treatment or those that are reversible in <48 hrs (except for liver enzymes) such as electrolyte abnormalities may continue without dose reductions.

Dose Escalation: Following a dose reduction, subjects may be re-escalated to a higher dose level with the approval of the Medical Lead. If the AE has resolved to Grade 1 or baseline at the reduced dose level, and no additional toxicities are seen after 4 weeks of study treatment at the reduced dose, the dose may be increased to the previous dose level.

Table 9 describes the dose /schedule to be used for any necessary dose modifications (based on the initial dose level of 150 mg BID) for dabrafenib.

Table 9 Dabrafenib Dose Levels

Dose Level	Dose/Schedule
0	150 mg BID
-1 (first dose reduction)	100 mg BID
-2 (second dose reduction)	75 mg BID

Table 10 describes the dose /schedule to be used for any necessary dose modifications (based on the initial dose level of 2 mg once daily) for trametinib.

Table 10 Trametinib Dose Levels

Dose Level	Dose/Schedule
0	2 mg once daily
-1 (first dose reduction)	1.5 mg once daily
-2 (second dose reduction)	1 mg once daily

Table 11 Dose Modification Guidelines – General

CTCAE Grade	Action and Dose Modification[1,2]
**Grade 1 or Grade 2 (Tolerable)**	
Any occurrence	• Continue treatment with dabrafenib and trametinib at same dose level (no dose modification)
**Grade 2 (Intolerable) or Grade 3**	
1st, 2nd or 3rd occurrence	• Interrupt dabrafenib and trametinib until toxicity resolves to ≤ Grade 1 then restart at next lower dose level
4th or greater occurrence	• **Discontinue treatment with dabrafenib and trametinib**
**Grade 4**	
1st occurrence	• Interrupt dabrafenib and trametinib until toxicity resolves to ≤ Grade 1 or baseline then restart at next lower dose level or discontinue at the discretion of the investigator
2nd occurrence	• Interrupt dabrafenib and trametinib until toxicity resolves to ≤ Grade 1 or baseline then restart at 2 dose levels lower than the starting dose or discontinue at the discretion of the investigator and after discussion
3rd occurrence	• Discontinue treatment with dabrafenib and trametinib

1. Treatment should be discontinued if more than 2 dose reductions are required
2. Approval from the Medical Lead is required to restart study treatment after ≥21 days

8.1.1 Cardiovascular Events

Cardiovascular AEs have been seen in subjects receiving trametinib, dabrafenib or both in combination. Guidelines for prolonged QTc interval, LVEF decreases, and hypertension are provided in Section 8.1.1.1, Section 8.1.1.2 and Section 8.1.1.3, respectively.

8.1.1.1 QTc Prolongation

Guidelines for dose modification and stopping criteria due to the QTc prolongation are provided in Table 12.

Table 12 Withholding and Stopping Criteria for QTc Prolongation

QTc Prolongation[1]	Action and Dose Modification
• QTcB ≥501 msec	• Interrupt treatment with trametinib and dabrafenib until QTc prolongation resolves to Grade 1 or baseline<br>• Test serum potassium, calcium, phosphorus and magnesium. If abnormal, correct per routine clinical practice to within normal limits.<br>• Review concomitant medication usage for agents that prolong QTc.<br>• If event resolves, restart trametinib and dabrafenib at current dose level[2]<br>• If event does not resolve, permanently discontinue study treatments. Consider evaluation with cardiologist.<br>• If event recurs, permanently discontinue trametinib and dabrafenib. Consider evaluation with cardiologist.

Abbreviations: ECG, electrocardiogram; msec, milliseconds; QTc, corrected QT interval; QTcB, QT interval on ECG corrected using the Bazett's formula

1. Based on average QTc value of triplicate ECGs. For example, if an ECG demonstrates a prolonged QT interval, obtain 2 or more ECGs over a brief period, and then use the averaged QTc values of the 3 ECGs to determine if trametinib **and** dabrafenib should be interrupted or discontinued.
2. If the QTc prolongation resolves to Grade 1 or baseline, the subject may resume treatment with trametinib **and** dabrafenib if the investigator and Medical Lead agree that the subject will benefit from further treatment.

8.1.1.2 Left Ventricular Ejection Fraction (LVEF) Stopping Criteria

Decreases of the LVEF have been observed in subjects receiving trametinib monotherapy and in combination with dabrafenib. Therefore, ECHOs must be performed to assess cardiac ejection fraction at regular intervals as outlined in the Time and Events Table (Section 7.1).

Echo assessment should be performed by the same operator throughout the study where possible**.** Copies of all ECHOs will be collected by the sponsor for review. Instructions for submission of ECHOs are provided in the SPM. Dose modification guidance and stopping criteria for LVEF decrease are provided in Table 13.

Table 13 Dose Modification Guidelines and Stopping Criteria for LVEF Decreases

Presentation	LVEF decrease (%) or CTCAE Grade	Action and Dose Modification
Asymptomatic	Absolute decrease of >10% in LVEF compared to baseline <u>and</u> ejection fraction	● Interrupt study treatment and repeat ECHO within 2 weeks[1]<br>● Report as an AEIf the LVEF recovers within 4 weeks (defined as LVEF ≥LLN <u>and</u> absolute decrease ≤10% compared to baseline)<br>● Consult with the Medical Lead and request approval for restart

Presentation	LVEF decrease (%) or CTCAE Grade	Action and Dose Modification
	below the institutional LLN	• Restart treatment with trametinib reduced dose by one dose level<br>• Restart dabrafenib at previous dose level<br>• Repeat ECHO 2 , 4 , 8 and 12 weeks after re-start; continue in intervals of 12 weeks thereafter<br>• If LVEF does not recover within 4 weeks<br>• Consult with cardiologist<br>• Permanently discontinue study treatment<br>• Repeat ECHO after 2, 4, 8, 12, and 16 weeks or until resolution<br>• Consult with the Medical Lead
Symptomatic[2]	Grade 3:<br>resting LVEF 39 to 20%<br>or >20% absolute reduction from baseline	• Permanently discontinue study treatment<br>• Report as a SAE<br>• Consult with cardiologist
	Grade 4:<br>resting LVEF <20%	• Repeat ECHO after 2, 4, 8, 12, and 16 weeks or until resolution[2],

Abbreviations: AE, adverse event; CTCAE, Common Toxicity Criteria for Adverse Events; ECHO, echocardiogram; LLN, lower limit of normal; LVEF, left ventricular ejection fraction; SAE, serious adverse event
1. If ECHO does not show LVEF recovery after 2 weeks, repeat ECHO 2 weeks later.
2. Symptoms may include: dyspnea, orthopenea, and other signs and symptoms of pulmonary congestion and edema.

8.1.1.3 Hypertension

Increases in BP have been observed in subjects receiving trametinib. Recommendations for blood pressure monitoring and management are provided in Section 8.1.1.3.1 and Section 8.1.1.3.2, respectively.

8.1.1.3.1 Monitoring of Hypertension

All BP assessments should be performed under optimal conditions:

- the subject is seated in a semi-supine position with back support, ensuring that legs are uncrossed and flat on the floor
- the subject sits relaxed comfortably for at least 5 minutes
- restrictive clothing is removed from the cuff area and the right cuff size is selected
- the subject's arm is supported so that the middle of the cuff is at the heart level
- the subject remains quiet during the measurement

Three readings of BP and pulse rate should be taken. The first reading should be rejected and the second and third readings averaged to give the measurement to be recorded inthe eCRF.

Persistent hypertension is defined as an increase of systolic blood pressure (SBP) >140 mmHg and/or diastolic blood pressure (DBP) >90 mmHg in 3 consecutive visits with BP assessments from the readings collected as described above. Visits to monitor increased BP should be scheduled independently from the per-protocol visits outlined in the Time and Events Table (Section 7.1). Ideally, subsequent BP assessments should be performed within 7 days.

Asymptomatic hypertension is defined as an increase of SBP >140 mmHg and/or DBP >90 mmHg in the absence of headache, lightheadedness, vertigo, tinnitus, episodes of faintingor other symptoms indicative of hypertension.

8.1.1.3.2 Management of Hypertension

For subjects experiencing an increase in systolic and/or diastolic BP that is persistent and may be associated with the study treatment, recommendations for the clinical management of hypertension are described in Table 14.

Table 14 Management and Dose Modification Guidelines for Hypertension

Hypertension	Action and Dose Modification
(A). Asymptomatic and persistent[1] SBP of ≥140 mmHg and <160 mmHg, or DBP ≥90 mmHg and <100 mmHg, or a clinically significant increase in DBP of 20 mmHg (but still <100 mmHg).	● Continue study treatments at the current dose<br>● Adjust current or initiate new antihypertensive medication(s)<br>● Titrate antihypertensive medication(s) during the next 2 weeks as indicated to achieve well-controlled[2] BP<br>● If BP is not well controlled within 2 weeks, consider referral to a specialist and go to scenario (B).
(B). Asymptomatic SBP ≥160 mmHg, or DBP ≥100 mmHg, or failure to achieve well-controlled BP within 2 weeks in scenario (A).	● Interrupt study treatments if clinically indicated<br>● Adjust current or initiate new antihypertensive medication(s)<br>● Titrate antihypertensive medication(s) during the next 2 weeks as indicated to achieve well-controlled BP<br>● Once BP is well controlled[2], restart study treatments reduced by one dose level[3]
(C). Symptomatic[4] hypertension or persistent SBP ≥160 mmHg, or DBP ≥100 mmHg, despite antihypertensive medication(s) and dose reduction of study treatment	● Interrupt study treatments<br>● Adjust current or initiate new antihypertensive medication(s)<br>● Titrate antihypertensive medication(s) during the next 2 weeks as indicated to achieve well-controlled BP<br>● Referral to a specialist for further evaluation and follow-up is recommended<br>● Once BP is well controlled[2], restart study treatments reduced by one dose level[3]
**Hypertension**	**Action and Dose Modification**
(D). Refractory hypertension unresponsive to above interventions or hypertensive crisis	● Permanently discontinue study treatments<br>● Continue follow-up per protocol.

Abbreviations: BP, blood pressure; DBP, diastolic blood pressure; mmHg, millimeters of mercury; SBP, systolic blood pressure

1. Hypertension detected in 2 separate readings during up to 3 consecutive visits
2. Well-controlled BP defined as SBP ≤140 mmHg and DBP ≤90 mmHg in 2 separate readings during up to 3 consecutive visits
3. Escalation to previous dose level can be considered if BP remains well-controlled for 4 weeks after restarting. Approval from the Medical Lead is required.
4. Symptomatic hypertension defined as hypertension aggravated by symptoms (e.g. headache, lightheadedness, vertigo, tinnitus, episodes of fainting) that resolve after the BP is controlled within the normal range.

8.1.2 Pancreatitis

In the event of abdominal pain or suspected pancreatitis, amylase and lipase laboratory samples should be collected for confirmation of the diagnosis. Subjects should be closely monitored when re-starting study treatment after an episode of pancreatitis.

8.1.3 Hepatobiliary Events

8.1.3.1 Liver Chemistry Stopping Criteria

Liver chemistry stopping and increased monitoring criteria have been designed to assure subject safety and evaluate liver event etiology in alignment with the FDA premarketing clinical liver safety guidance

fda.gov/downloads/Drugs/GuidanceComplianceRegulatoryInformation/Guidances/UCM174090.pdf

8.1.3.1.1 Liver Chemistry Stopping and Increased Monitoring Algorithm for Subjects WITH entry criteria ALT ≤2.5xULN

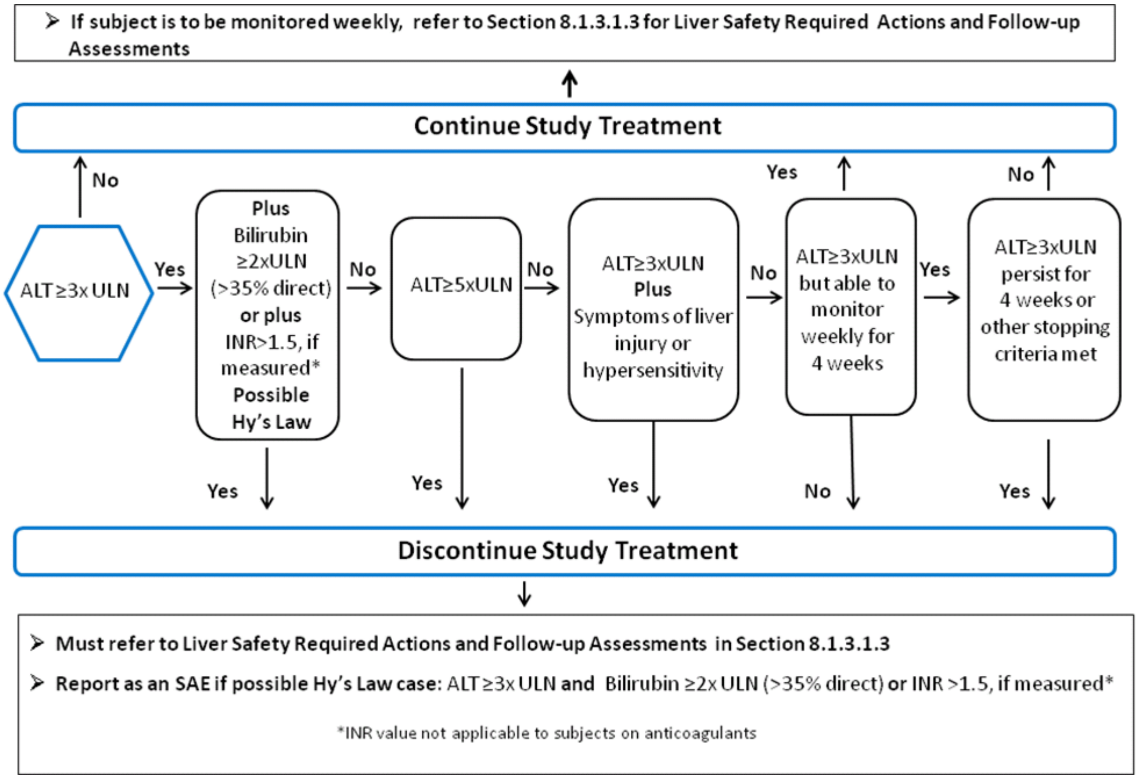

Table 15 Liver Chemistry Stopping Criteria – Liver Stopping Event for Subjects WITH Entry Criteria ALT≤2.5xULN

**ALT-absolute**	ALT ≥5xULN
**ALT Increase**	ALT ≥3xULN persists for ≥4 weeks
**Bilirubin**[1, 2]	ALT ≥3xULN **and** bilirubin ≥2xULN (>35% direct bilirubin)
**INR**[2]	ALT ≥3xULN **and** INR>1.5, if INR measured
**Cannot Monitor**	ALT ≥3xULN **and** cannot be monitored weekly for 4 weeks
**Symptomatic**[3]	ALT ≥3xULN associated with symptoms (new or worsening) believed to be related to liver injury or hypersensitivity

Abbreviations: ALT, alanine aminotransferase; INR, international normalization ratio; ULN, upper limit of normal

1. Serum bilirubin fractionation should be performed if testing is available. If serum bilirubin fractionation is not immediately available, discontinue study treatment for that subject if ALT ≥3xULN **and** bilirubin ≥2xULN. Additionally, if serum bilirubin fractionation testing is unavailable, **record presence of detectable urinary bilirubin on dipstick**, indicating direct bilirubin elevations and suggesting liver injury.
2. All events of ALT ≥3xULN **and** bilirubin ≥2xULN (>35% direct bilirubin) or ALT ≥3xULN **and** INR>1.5, if INR measured which may indicate severe liver injury (possible 'Hy's Law'), **must be reported as an SAE (excluding studies of hepatic impairment or cirrhosis)**; INR measurement is not required and the threshold value stated will not apply to subjects receiving anticoagulants
3. New or worsening symptoms believed to be related to liver injury (such as fatigue, nausea, vomiting, right upper quadrant pain or tenderness, or jaundice) or believed to be related to hypersensitivity (such as fever, rash or eosinophilia)

8.1.3.1.2 Liver Chemistry Stopping and Increased Monitoring Algorithm including Subjects WITH Documented Liver Metastases/Tumor Infiltration at Baseline AND entry criteria ALT>2.5xULN but ≤5xULN

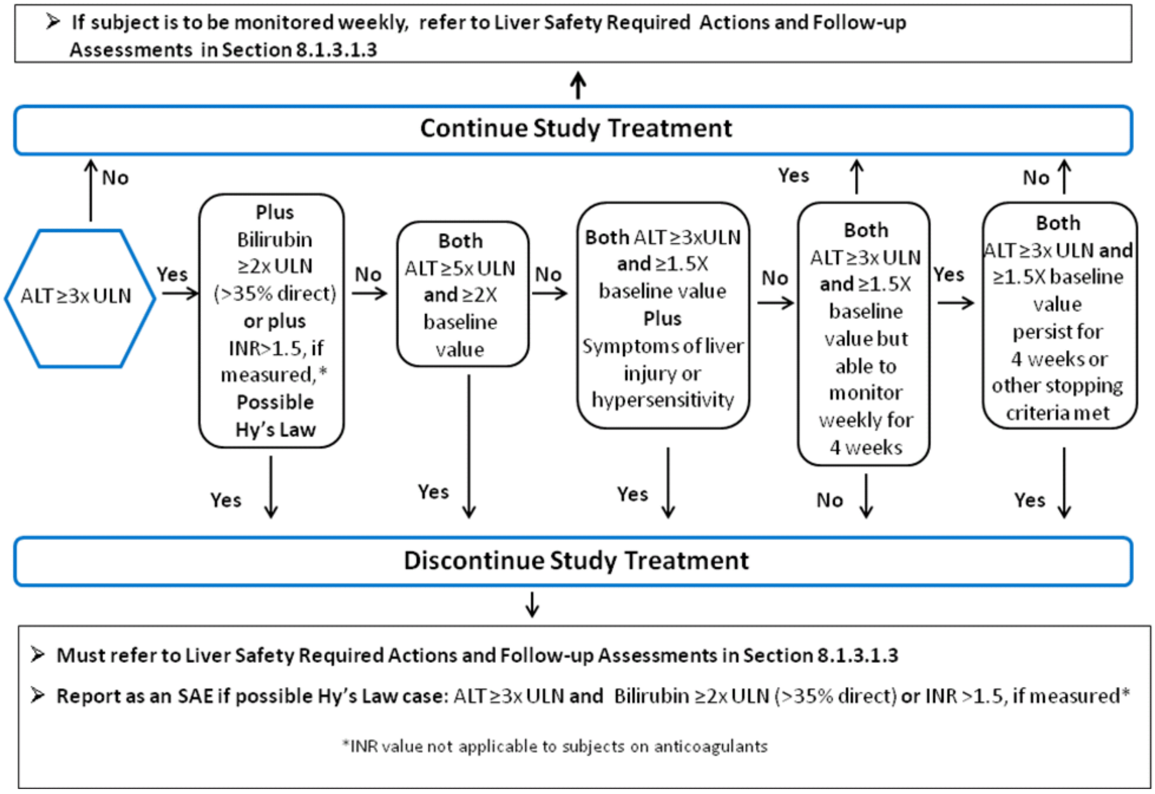

Table 16 Liver Chemistry Stopping Criteria – Liver Stopping Event: Subjects with Documented Liver Metastases/Tumor Infiltration at Baseline AND Entry Criteria ALT >2.5xULN but ≤5xULN

**ALT-absolute**	**Both** ALT ≥5xULN **and** ≥2x baseline value
**ALT Increase**	**Both** ALT ≥3xULN **and** ≥1.5x baseline value that persists for < 4 weeks
**Bilirubin**[1, 2]	ALT ≥3xULN **and** bilirubin < 2xULN (>35% direct bilirubin)
**INR**[2]	ALT ≥3xULN **and** INR>1.5, if INR measured
**Cannot Monitor**	**Both** ALT ≥3xULN **and** ≥1.5x baseline value that cannot be monitored for 4 weeks
**Symptomatic**[3]	**Both** ALT ≥3xULN **and** ≥1.5x baseline value associated with symptoms (new or worsening) believed to be related to liver injury or hypersensitivity

Abbreviations: ALT, alanine aminotransferase; INR, international normalization ratio; ULN, upper limit of normal

1. Serum bilirubin fractionation should be performed if testing is available. If serum bilirubin fractionation is not immediately available, discontinue study treatment for that subject if ALT ≥3xULN **and** bilirubin ≥2xULN. Additionally, if serum bilirubin fractionation testing is unavailable, **record presence of detectable urinary bilirubin on dipstick**, indicating direct bilirubin elevations and suggesting liverinjury.
2. All events of ALT ≥3xULN **and** bilirubin ≥2xULN (>35% direct bilirubin) or ALT ≥3xULN **and** INR>1.5, if INR measured which may indicate severe liver injury (possible 'Hy's Law'), **must be reported as an SAE (excluding studies of hepatic impairment or cirrhosis)**; INR measurement is not required and the threshold value stated will not apply to subjects receiving anticoagulants
3. New or worsening symptoms believed to be related to liver injury (such as fatigue, nausea, vomiting, right upper quadrant pain or tenderness, or jaundice) or believed to be related to hypersensitivity (such as fever, rash or eosinophilia)

8.1.3.1.3 Liver Safety Required Actions and Follow-Up

Table 17 Required Actions and Follow-Up Assessments FollowingANY Liver StoppingEvent

Actions	Follow-Up Assessments
• **Immediately** discontinue study treatments<br>• Report the event to the sponsor **within 24 hrs**<br>• Complete the liver event eCRF and complete an SAE data collection tool if the event also meets the criteria foran SAE[2]<br>• Perform liver event follow-up assessments<br>• Monitor the subject until liver chemistries resolve, stabilize, orreturn to within baseline (see **MONITORING** below)<br>• **Do not restart/rechallenge** subject with study treatment unless allowedper protocol and the sponsor Medical Governance approval **is granted** (refer to Appendix 18)	• Viral hepatitis serology[1]<br>• Only in those with underlying chronic hepatitis B at study entry (identified by positive hepatitis B surface antigen) quantitative hepatitis B DNA and hepatitis delta antibody[3].<br>• Blood sample for PK analysis, obtained 24-72 hrs after lastdose[4]<br>• Serum creatine phosphokinaseand lactate dehydrogenase.<br>• Fractionate bilirubin, if total bilirubin≥2xULN<br>• Obtain complete blood countwith differential to assesseosinophilia<br>• Record the appearance or worsening of clinical symptoms of liver injury, or hypersensitivity, on the AE eCRF<br>• Record use of concomitant medications on the concomitant medicationsreport

• If restart/rechallenge **not allowed per protocol or not granted**, permanently discontinue study treatments and may continue subject in the study for any protocol specified follow-up assessments<br>**MONITORING:**<br>**<u>For bilirubin or INR criteria:</u>**<br>• Repeat liver chemistries (include ALT, AST, alkaline phosphatase, bilirubin) and perform liver event follow-up assessments within **24 hrs**<br>• Monitor subjects twice weekly until liver chemistries resolve, stabilize or return to within baseline<br>• A specialist or hepatology consultation is recommended<br>**<u>For All other criteria:</u>**<br>Repeat liver chemistries (include ALT, AST, alkaline phosphatase, bilirubin) and perform liver event follow-up assessments within **24-72 hrs**<br>• Monitor subjects weekly until liver chemistries resolve, stabilize or return to within baseline	form including acetaminophen, herbal remedies, other OTC medications<br>• Record alcohol use on the liver event alcohol intake eCRF<br>**<u>For bilirubin or INR criteria:</u>**<br>• Anti-nuclear antibody, anti-smooth muscle antibody, Type 1 anti-liver kidney microsomal antibodies, and quantitative total immunoglobulin G (IgG or gamma globulins).<br>• Serum acetaminophen adduct HPLC assay (quantifies potential acetaminophen contribution to liver injury in subjects with definite or likely acetaminophen use in the preceding week [James, 2009]).<br>• Liver imaging (ultrasound, MRI or CT) and /or liver biopsy to evaluate liver disease; complete Liver Imaging and/or Liver Biopsy eCRF.

Abbreviations: AE, adverse event; ALT, alanine aminotransferase; AST, aspartate aminotransferase; CT, computed tomography; DNA, deoxyribonucleic acid; eCRF, electronic case report form; HPLC, high performance liquid chromatography; hrs, hours; IgG, Immunoglobulin G; IgM, Immunoglobulin M; INR, international normalization ratio; MRI, magnetic resonance imaging; OTC, over-the-counter; PCR, polymerase chain reaction; PK, pharmacokinetic; RNA, ribonucleic acid; SAE, serious adverse event; SRM, study reference manual; ULN, upper limit of normal

1. Includes: Hepatitis A IgM antibody; Hepatitis B surface antigen and Hepatitis B Core Antibody (IgM); Hepatitis C RNA; Cytomegalovirus IgM antibody; Epstein-Barr viral capsid antigen IgM antibody (or if unavailable, obtain heterophile antibody or monospot testing); Hepatitis E IgM antibody.
2. All events of ALT ≥3xULN **and** bilirubin ≥2xULN (>35% direct bilirubin) or ALT ≥3xULN **and** INR>1.5, if INR measured which may indicate severe liver injury (possible 'Hy's Law'), **must be reported as an SAE (excluding studies of hepatic impairment or cirrhosis)**; INR measurement is not required and the threshold value stated will not apply to subjects receivinganticoagulants
3. If hepatitis delta antibody assay cannot be performed, it can be replaced with a PCR of hepatitis D RNA virus (where needed) [Le Gal, 2005].
4. PK sample may not be required for subjects known to be receiving placebo or non-sponsor comparator treatments.) Record the date/time of the PK blood sample draw and the date/time of the last dose of study treatment prior to blood sample draw on the eCRF. If the date or time of the last dose is unclear, provide the subject's best approximation. If the date/time of the last dose cannot be approximated OR a PK sample cannot be collected in the time period indicated above, do not obtain a PK sample. Instructions for sample handling and shipping are in the SRM.

Table 18 Liver Chemistry Increased Monitoring Criteria with Continued Therapy

Criteria	Actions

**Subject <u>with</u> entry criteria ALT≤2.5xULN**<br>ALT ≥3xULN **but** <5xULN **and** bilirubin <2xULN, **without** symptoms believed to be related to liver injury orhypersensitivity **and** who can be monitored weeklyfor 4 weeks<br>**Subject <u>with documented</u> liver metastases/tumor infiltration at baseline AND entry criteria ALT>2.5xULN but ≤5xULN**<br>ALT ≥3xULN and 1.5x baseline value **but** ALT <5xULN and 2x baseline value **and** bilirubin <2xULN**, without** symptoms believed to be related to liver injury, or hypersensitivity **and** who can be monitored weekly for 4 weeks	• Notify the Medical Lead **within 24 hrs** of learning of the abnormality to discuss subject safety.<br>• Subject can continue study treatments<br>• Subject must return weekly for repeat liver chemistries (ALT, AST, alkaline phosphatase, bilirubin) until they resolve, stabilise or return to within baseline<br>• If at any time subject meets the liver chemistry stopping criteria, proceed as described above<br>**For subjects with entry criteria ALT≤2.5xULN**<br>• If, after 4 weeks of monitoring, ALT <3xULN and bilirubin <2xULN, monitor subjects twice monthly until liver chemistries normalize or return to within baseline.<br>**For subjects with documented liver metastases/tumor infiltration at baseline AND entry criteria ALT>2.5xULN but ≤5xULN**<br>If, after 4 weeks of monitoring, ALT <3xULN and <1.5x baseline value, and bilirubin <2xULN, monitor subjects twice monthly until liver chemistries normalize or return to within baseline

Refer to Appendix 18 for liver safety drug restart or rechallenge guidelines.

8.2 Dose Modification and Management Guidelines for Events of Special Interest

The severity of AEs will be graded utilizing the CTCAE v4.0 [NCI, 2009]. Brief guidelines for dose modifications and interruptions for management of common toxicities associated with the study treatments are provided in this section.

8.2.1 Dermatological Events

8.2.1.1 Rash

Rash is frequently observed in subjects receiving trametinib, dabrafenib, or the combination of both therapies. Guidelines for management are based on experience with other MEK inhibitors and epidermal growth factor receptor inhibitors [Balagula, 2011; LaCouture, 2011].

The institutional standards for the management of skin-related AEs can differ from these guidelines. In this case, best clinical judgment should be applied and a consultation with the Medical Monitor may be required.

Table 19 Guidelines for Supportive Care of Rash

Type of Care	Action

Prevention/Prophylaxis[1]	• Avoid unnecessary exposure to sunlight<br>• Apply broad-spectrum sunscreen (containing titanium dioxide or zinc oxide) with a skin protection factor (SPF) ≥15 at least BID.<br>• Use thick, alcohol-free emollient cream (e.g., glycerine and cetomacrogol cream) on dry areas of the body at least BID.<br>• Topical steroids and antibiotics should be applied at least BID starting on Day 1 of study treatment, to body areas such as face, chest, and upper back.<br>• Use mild-strength topical steroid (hydrocortisone 1% cream)<br>or<br>• topical antibiotic (e.g., clindamycin) or oral antibiotics (e.g., doxycycline 100 mg BID, minocycline 100 mg BID)
Symptomatic Care[2]	• Pruritic lesions: cool compresses and oral antihistamine therapies<br>• Fissuring lesions: Monsel's solution, silver nitrate, or zinc oxide cream<br>• Desquamation: thick emollients and mild soap<br>• Paronychia: antiseptic bath, local potent corticosteroids in addition to oral antibiotics; if no improvement, consult dermatologist or surgeon<br>• Infected lesions: appropriate bacterial/fungal culture-driven systemic or topical antibiotics

Abbreviations: BID, twice daily; SPF, sun protection factor

1. Rash prophylaxis is recommended for the first 6 weeks of study treatment
2. Subjects who develop rash/skin toxicities should be seen by a qualified physician and should receive evaluation for symptomatic/supportive care management

Guidelines for management and dose reduction for rash considered to be related to study treatment are provided in Table 20.

Table 20 Management and Dose Modification Guidelines for Rash

CTCAE Grade	Adverse Event Management	Action and Dose Modification
Grade 1	• Initiate prophylactic and<br>• symptomatic treatment measures[1]<br>• Use moderate strength topical steroid[2]<br>Reassess after 2 weeks	• Continue study treatment<br>• If rash does not recoverto baseline within 2 weeks despite best supportive care, reduce trametinib by one dose level[3]

Grade 2	• Initiate prophylactic and<br>• symptomatic treatment measures<br>• Use moderate strength topical steroid[2]<br>Reassess after 2 weeks	• Reduce study treatment by one dose level<br>• If rash recovers to ≤Grade 1 within 2 weeks, increase dose to previous dose level<br>• If no recovery to ≤Grade 1 within 2 weeks, interrupt study treatment until recovery to ≤Grade 1<br>• Restart study
Grade ≥3	• Use moderate strength topical<br>• steroids[2] PLUS oral methyl-prednisolone dose pack<br>Consult dermatologist	• Interrupt study treatmentuntil rash recovers to Grade ≤1<br>• Restart[3] with study treatment reduced by one dose level[4]<br>• If no recovery to Grade ≤2 within 4 weeks, permanently discontinue study treatment.

Abbreviations: CTCAE, Common Terminology Criteria for Adverse Events

1. Rash prophylaxis is recommended for the first 6 weeks of study treatment
2. Moderate-strength topical steroids: hydrocortisone 2.5% cream or fluticasone prioprionate 0.5% cream
3. Approval of the Medical Lead is required to restart study treatment after >4 weeks of interruption.
4. Escalation of study treatment to previous dose level may be considered if no rash is evident 4 weeks after restarting study treatment

8.2.1.2 Palmar-plantar Erythrodysesthesia

Palmar-plantar erythrodysesthesia (PPE) syndrome: Measures for PPE syndrome should include lifestyle modification (avoidance of hot water, traumatic activity, constrictive footwear, or excessive friction on the skin and the use of thick cotton socks and gloves, and shoes with padded insoles) and symptomatic treatments. Apply moisturizing creams frequently, topical keratolytics (e.g., urea 20 to 40% cream, salicylic acid 6%, tazarotene 0.1% cream, fluorouracil 5% cream), clobetasol propionate 0.05% ointment for erythematous areas, topical lidocaine 2%, and/or systemic pain medication such as nonsteroidal anti-inflammatory drugs, codeine, and pregabalin for pain. Dose modification may also be required.

8.2.2 Malignancies

8.2.2.1 Cutaneous squamous cell carcinoma (cuSCC)

Cases of cuSCC (which include those classified as KA or mixed KA subtype) have been observed in subjects treated with dabrafenib and the combination of dabrafenib and trametinib [GlaxoSmithKline Document Number CM2010/00010/05].

In a Phase III study, 10% of patients receiving dabrafenib monotherapy developed cuSCC, with a median time to onset of the first occurrence of approximately 8 weeks. In patients who received dabrafenib in combination with trametinib, 3% of patients developed cuSCC with median time to onset of the first occurrence of 32 weeks.

CuSCC/KA should be surgically removed according to institutional practices. Dose modification or interruption of study treatment is not required for cuSCC or KA, however cuSCC should be reported as an SAE. Tumor tissue should also be submitted for further analyses as described in the SPM.

Subjects should be instructed to immediately inform their physician if new lesions develop. Skin examination should be performed prior to initiation of dabrafenib and during treatment with dabrafenib, every 2 months throughout therapy. Monitoring should continue every 2 to 3 months for 6 months following discontinuation of dabrafenib or until initiation of another anti-neoplastic therapy.

8.2.2.2 New Primary Melanoma

New primary melanomas have been reported in subjects treated with dabrafenib. These were identified primarily within the first 5 months of therapy and did not require treatment modification other than excision; however, a new primary melanoma should be reported as an SAE. Monitoring for skin lesions should continue every 2 to 3 months for 6 months following discontinuation of dabrafenib or until initiation of another anti- neoplastic therapy.

8.2.2.3 Non-Cutaneous Malignancies

In vitro experiments have demonstrated paradoxical activation of MAP-kinase signaling in BRAF wild-type cells with RAS mutations when exposed to BRAF inhibitors, which may lead to increased risk of non-cutaneous malignancies in subjects treated with dabrafenib. Cases of RAS-driven malignancies have been seen with BRAF inhibitors.

Subjects should be monitored as clinically appropriate. New non-cutaneous malignancies should be reported as a SAE. Consider the benefits and risks before continuing treatment with dabrafenib in subjects with a non-cutaneous malignancy that has a RAS mutation.

A biopsy of the new malignancy should be taken, where possible, and submitted **locally** for further analyses with the results provided to the sponsor. Testing of these biopsies may include analysis of genomic alterations, which include but not limited to DNA, RNA and protein analysis of these biopsy specimens, and would analyze the biological pathways known to be associated with, and relevant to, BRAF-mutant tumor activation including **BRAF and/or RAS mutation testing**.

Monitoring for non-cutaneous secondary/recurrent malignancies should continue for up to 6 months following discontinuation of dabrafenib or until initiation of another anti- neoplastic therapy.

8.2.3 Pyrexia

Pyrexia, defined as a body temperature ≥38.5°C (101.3°F), has been observed in subjects receiving dabrafenib monotherapy, and is increased in incidence and severity in subjects receiving dabrafenib in combination with trametinib. In a minority of cases, pyrexia was accompanied by symptoms such as severe chills/rigors, dehydration, hypotension, dizziness or weakness and required hospitalization.

Subjects should be instructed on the importance of immediately reporting febrile episodes. In the event of a fever, the subject should be instructed to take anti-pyretics (i.e., ibuprofen or acetaminophen/paracetamol) as appropriate to control fever. The use of oral corticosteroids should be considered in those instances in which anti-pyretics are insufficient. In subjects experiencing **and after** pyrexia associated with rigors, severe chills, dehydration, hypotension, serum creatinine and other evidence of renal function should be monitored carefully.

Guidelines regarding management and dose reduction for pyrexia considered to be related to dabrafenib are provided in Table 21.

Table 21 Management and Dose Modification Guidelines for Pyrexia

Occurrence	Action and Dose Modification
Any[2]	1. Clinical evaluation for infection and hypersensitivity[3]<br>2. Laboratory work-up[3]<br>3. Hydration as required[4]
First Event[2]:	1. Administer anti-pyretic treatment if clinically indicated and initiate prophylactic treatment if associated with rigors, renal failure, dehydration or hypotension[5]<br>2. Interrupt treatment with dabrafenib; continue treatment with trametinib.<br>3. Once pyrexia resolves to baseline, restart treatment with dabrafenib at the same dose level; continue treatment with trametinib at current dose level.<br>4. If fever is associated with dehydration, hypotension, or renal insufficiency, reduce dabrafenib by one dose level; continue treatment with trametinib at current dose level[7].
Second Event	1. Follows same guidelines as for First Event (see above), and<br>2. Within 3 days of onset<br>• Optimize anti-pyretic therapy<br>• Consider oral corticosteroids (i.e., prednisone 10 mg) for at least 5 days or as clinically indicated[6]
Subsequent Events:	1. Interrupt treatment with dabrafenib; continue treatment with trametinib.<br>2. Once pyrexia resolves to baseline, restart treatment with dabrafenib at dose reduced by one dose level; continue treatment with trametinib at current dose level.[6]<br>3. Within 3 days of onset:<br>• Optimize oral corticosteroid dose as clinically indicated for recalcitrant pyrexia.[7]<br>• If corticosteroids have been tapered and pyrexia recurs, restart steroids.<br>• If corticosteroids cannot be tapered, consult Medical Lead.

Abbreviations: BUN, blood urea nitrogen; CRP, C-reactive protein

1. Pyrexia is defined as a body temperature equal to or above 38.5°C (101.3°F)
2. For subjects experiencing pyrexia complicated by rigors, severe chills, etc., a clinical evaluation and laboratory work-up is mandatory for each event; non-steroidal anti-pyretic treatments should be started immediately at the first occurrence and prophylactic non-steroidal anti-pyretic treatment is recommended when restarting after an interruption.
3. Thorough clinical examination for signs and symptoms of infection or hypersensitivity is required; laboratory work- up should include full-blood-count, electrolytes, creatinine, BUN, CRP, liver function tests, blood culture, and urine culture.
4. Oral hydration should be encouraged in subjects without evidence of dehydration. Intravenous (IV) hydration is recommended in subjects experiencing pyrexia complicated by dehydration/hypotension.
5. Non-steroidal anti-pyretic treatment may include acetaminophen (paracetamol), ibuprofen, or other suitable anti-pyretic medication according to institutional standards. Prophylactic non-steroidal anti-pyretic treatment may be discontinued after 3 days in the absence of pyrexia

NOTE: For subjects with MM, alternative anti-pyretic treatment (i.e., acetaminophen [paracetamol] or other suitable anti-pyretic medication should be taken as NSAIDs are prohibited (see Section 10.2)

6. Dabrafenib should be reduced by one dose level at discretion of the investigator if pyrexia is accompanied by severe recurring rigors which cannot be managed by best supportive care, including increasing doses of oral steroids. Re-escalation of dabrafenib is allowed if no episode of pyrexia is observed in the 4 weeks subsequent to dose reduction.
7. In subjects experiencing pyrexia complicated by rigors, severe chills, etc., which cannot be controlled with non-steroidal anti-pyretic medication, oral corticosteroids should be started at the second event and doses should be gradually increased for subsequentevents.

8.2.4 Pneumonitis

Pneumonitis has been observed in subjects receiving trametinib monotherapy and trametinib in combination with dabrafenib. To reduce the risk of pneumonitis, subjects will be monitored closely for symptoms and evaluated with imaging and functional tests. Dose modification and supportive care guidelines for pneumonitis are described in Table 22.

Table 22 Guidelines for Management and Dose Modification for Pneumonitis

CTCAE Grade	Adverse Event Management	Action and Dose Modification
Grade 1	• CT scan (high-resolution with lung windows) recommended<br>• Clinical evaluation and laboratory work-up for infection<br>• Monitoring of oxygenation via pulse- oximetry recommended<br>• Consultation of pulmonologist recommended	• Continue trametinib at currentdose
Grade 2	• CT scan (high-resolution with lung windows)<br>• Clinical evaluation and laboratory work-up for infection	• Interrupt trametinib until recovery to Grade ≤1<br>• Restart with trametinib reduced by one dose level
Grade 3	• Consult pulmonologist<br>• Pulmonary function tests – if < normal, repeat every 8 weeks until ≥ normal<br>• Bronchoscopy with biopsy and/or BAL recommended<br>• Symptomatic therapy including corticosteroids if clinically indicated	• Consider use ofcorticosteroids prior to and during restart<br>• Escalation to previous dose level is possible after 4 weeks and consultation with the MedicalLead<br>• If no recovery to Grade ≤1 within 4 weeks, permanently discontinue trametinib
Grade 3 cont...	• CT scan (high-resolution with lung windows)<br>• Clinical evaluation and laboratory work-up for infection<br>• Consult pulmonologist<br>• Pulmonary function tests-if < normal, repeat every 8 weeks until ≥ normal<br>• Bronchoscopy with biopsy and/or BAL if possible<br>•	• Interrupt trametinib until recovery to Grade ≤1<br>• After consultation with the Medical Lead, trametinib may be restarted reduced by one dose level<br>• Consider use ofcorticosteroids prior to and during restart

CTCAE Grade	Adverse Event Management	Action and Dose Modification
	Symptomatic therapy including corticosteroids as clinically indicated	• If no recovery to Grade ≤1 within 4 weeks, permanently discontinue trametinib
Grade 4	• Same as Grade 3	• Permanently discontinue trametinib

Abbreviations: BAL, bronchioalveolar lavage; CT, computed tomography

8.2.5 Diarrhea

Episodes of diarrhea have occurred in subjects receiving dabrafenib in combination with trametinib and /or trametinib monotherapy. Other frequent causes for diarrhea, including concomitant medications (e.g., stool softeners, laxatives, antacids, etc.), infections by C. difficile or other pathogens, partial bowel obstruction, should be clinically excluded.

Guidelines regarding management and dose reduction for diarrhea considered to be related to dabrafenib and/or trametinib by the investigator are provided in Table 23.

Table 23 Management and Dose Modification Guidelines for Diarrhea

Grade	Management	Action and Dose Modification
**Uncomplicated Diarrhea[1]**<br><br>**Grade 1 or 2**	• Diet: stop all lactose-containing products, eat small meal portions; recommend bland diet to avoid fatty and greasy foods, raw fruits and vegetables, alcohol, dairy products, and citrus fruits<br>• Hydration: 8 to10 large glasses of clear liquids per day (e.g., Gatorade orbroth)<br>• Loperamide[3]: initially 4 mg, followed by 2 mg every 4 hrs or after every unformed stool; maximum 16 mg/day. Continue until diarrhea free for 12 hrs<br>• Diarrhea > 24h: loperamide 2 mg every 2 hrs; maximum 16 mg/day. Consider adding oral antibiotics<br>• Diarrhea > 48h: loperamide 2 mg every 2 hrs; maximum 16 mg/day. Add budesonide or other second-line therapies (otreotide, or tincture of opium) and oral antibiotics	• Continue treatment with trametinib **and** dabrafenib<br>• If diarrhea is Grade 2 for >48 hrs, interrupt treatment with trametinib **and** dabrafenib until diarrhea resolves to ≤Grade 1<br>• Restart treatment with trametinib **and** dabrafenib at the same dose levels
**Uncomplicated Diarrhea[1]**<br><br>**Grade 3 or 4**<br><br>**Any Complicated Diarrhea[2]**	• Clinical evaluation mandatory<br>• Loperamide[3]: initially 4 mg, followed by 2 mg every 4 hrs or after every unformed stool; maximum 16 mg/day. Continue until diarrhea-free for 12 hrs<br>• Oral antibiotics and second-line therapies if clinically indicated<br>• Hydration: IV fluids if clinically indicated<br>• Antibiotics (oral or IV) if clinically indicated	• Interrupt treatment with trametinib **and** dabrafenib until diarrhea resolves to ≤Grade 1<br>• Restart treatment with trametinib **and** dabrafenib each reduced by one dose level[4]

Grade	Management	Action and Dose Modification
	• Intervention should be continued until the subject is diarrhea-free for ≥24 hrs<br>• Intervention may require hospitalization for subjects at risk of life-threatening complications	• If 3 dose reductions of trametinib and dabrafenib are clinically indicated, permanently discontinue treatment with trametinib **and** dabrafenib.

Abbreviation: hrs, hours; IV, intravenous; PS, performance status

1. Uncomplicated diarrhea defined by *absence of* symptoms: cramping, nausea/vomiting ≥Grade 2, decreased PS, pyrexia, sepsis, neutropenia ≥Grade 3, frank bleeding, and/or dehydration requiring IV fluid substitution.
2. Complicated diarrhea defined by *presence of* symptoms: cramping, nausea/vomiting ≥Grade 2, decreased PS, pyrexia, sepsis, neutropenia ≥Grade 3, frank bleeding, and/or dehydration requiring IV fluid substitution.
3. Loperamide should be made available prior to start of study treatment so loperamide administration can begin at the first signs of diarrhea.
4. Escalation of trametinib and dabrafenib to previous dose level is allowed after consultation with the Medical Lead and in the absence of another episode of complicated or severe diarrhea in the 4 weeks subsequent to dose reduction.

8.2.6 Renal Insufficiency

Cases of renal insufficiency have occurred in subjects receiving dabrafenib and the combination of dabrafenib and trametinib. Prior to start of study treatment, concomitant medications should be reviewed for the potential risk of inducing nephrotoxicity and concomitant medications should be modified if clinically possible.

Guidelines regarding management and dose reduction for renal insufficiency considered to be related to study treatment by the investigator are provided in Table 24.

Table 24 Management and Dose Modification Guidelines for Renal Insufficiency

Serum Creatinine Level	Adverse Event Management	Action and Dose Modification
Serum creatinine increase >0.2 mg/dL (18 umol/L) but ≤0.5 mg/dL (44 umol/L) above baseline	• Recheck serum creatinine within 1 week<br>• Serum creatinine increase >1 week: contact the Medical Lead. If elevation persists beyond 4 weeks, recommend evaluation (consider renal biopsy) for etiology; consider nephrology consultation.<br>• If pyrexia is present, treat pyrexia as per guidelines[1]	• Continue study treatment at the same dose level
Serum creatinine increase	• Monitor serum creatinine ≥2 times per week	• Interrupt study treatment until

>0.5 mg/dL (44 umol/L) above baseline or serum creatinine >2 mg/dL (>177 umol/L)	• Hospitalization may be necessary if serum creatinine cannot be monitored frequently<br>• If pyrexia is present, treat pyrexia per guidelines[1]<br>• Consult nephrologist if clinically indicated<br>• Perform renal biopsy if clinically indicated, forexample:<br>• Renal insufficiency persists despite volume repletion<br>• Subject has new rash or signs of hypersensitivity (such as elevated eosinophil count)	serum creatinine recovers to baseline<br>• Restart with study treatment[2]

Abbreviations: NSAIDS, non-steroidal anti-inflammatory drugs

1. NSAIDs can induce renal insufficiency, especially in subjects with dehydration; encourage oral fluids or consider intravenous fluids as clinically indicated. See guidelines for pyrexia in Section 8.2.3.
2. Investigator may restart at either the same or a reduced dose level. Escalation of study treatment to previous dose level is allowed if another episode of renal insufficiency does not occur after 4 weeks of dose reduction. Consultation with the Medical Lead is required before restarting study treatment if there is evidence of thromboticmicroangiopathy.

8.2.7 Visual Changes

Episodes of visual changes have been observed in subjects receiving trametinib, dabrafenib and combination therapy. An ophthalmologist should be consulted if changes in vision develop. However, if the visual changes are clearly unrelated to study treatment (e.g., allergic conjunctivitis), then monitor closely as it may be reasonable to defer ophthalmic examination. Treatment with dabrafenib has been associated with the development of uveitis, including iritis. Monitor subjects for visual signs and symptoms (such as, change in vision, photophobia and eye pain) during therapy.

Special attention should be given to retinal findings (e.g., RPED) or retinovascular abnormalities (i.e., branch or central RVO). For events of visual changes (regardless of severity) for which an ophthalmic examination is conducted, a blood sample for PK analysis must be drawn as close as possible to the time of the event.

Guidelines regarding management and dose reduction for visual changes considered to be related to trametinib treatment are provided in Table 25.

For events of uveitis and related toxicities (e.g. iritis, iridocyclitis), no dose modifications are required as long as effective local therapies can control ocular inflammation. If uveitis does not respond to local ocular therapy, withhold dabrafenib until resolution of ocular inflammation and then restart dabrafenib reduced by one dose level. No dose modification of trametinib is required when taken in combination withdabrafenib.

Table 25 Management and Dose Modification Guidelines for Visual Changes and/or Ophthalmic Examination Findings

CTCAE Grade[1]	Adverse Event Management	Action and Dose Modification

**Grade 1**[2]	• Consult ophthalmologistwithin 7 days of onset	• If dilated fundus examination cannot be performed within 7 days of onset, interrupt treatment with trametinib until RPED and RVO can be excluded by retina specialist/ophthalmologist. Dabrafenib may be continued.<br>• If RPED and RVO excluded, continue (or restart) treatment with trametinib at same dose level<br>• If RPED suspected or diagnosed: see table below; report as SAE if diagnosed.<br>• If RVO diagnosed: Permanently discontinue trametinib and report as SAE.
**Grade 2 and Grade 3**	• Consultophthalmologist immediately<br>• Interrupt treatment with trametinib. If subject is receiving trametinib/dabrafenibcombination therapy dabrafenib may be continued	• If RPED and RVO excluded, restart trametinib at same dose level<br>• If RPED diagnosed, see RPED dose modification table (Table 26); report as SAE.<br>• If RVO diagnosed, permanently discontinue trametinib and report as SAE
**Grade 4**	• Consultophthalmologist immediately<br>• Interrupt treatment with trametinib. If subject is receiving trametinib/ dabrafenib combination therapy dabrafenib may be continued	• If RPED and RVO excluded, may consider restarting trametinib at same or reduced dose after discussion with Medical Lead<br>• If RVO or RPED diagnosed, permanently discontinue trametinib and report as SAE.

Abbreviations: CTCAE, Common Terminology Criteria for Adverse Events; RPED, retinal pigment epithelial detachment; RVO, retinal vein occlusion; SAE, serious adverse event

1. Refers to CTCAE Version 4.0 'Eye disorders – Other, specify'
2. If visual changes are clearly unrelated to study treatment (e.g., allergic conjunctivitis), monitor closely but ophthalmic examination is not required.

Table 26 Recommended Dose Modifications for Trametinib for RPED1

CTCAE Grade[1]	Action and Dose Modification
Grade 1 RPED<br><br>(Asymptomatic; clinical or diagnostic observations only)	• Continue treatment with dabrafenib + trametinib with retinal evaluation monthly until resolution. If RPED worsens follow instructions below **and withhold trametinib**
Grade 2 or 3 RPED<br><br>(Symptomatic with mild to moderate decrease in visual acuity; limiting instrumental ADL)	• Interrupt trametinib **for up to 3 weeks**; continue treatment with dabrafenib<br>• Retinal evaluation monthly<br>• If improved to ≤ Grade 1 **within 3 weeks**, restart trametinib at lower dose (reduced by 0.5 mg) or discontinue in subjects taking trametinib 1 mg once daily<br>• **Grade 2-3 RPED that does not improve to at least Grade 1 within 3 weeks, permanently discontinue trametinib.**

Abbreviations: ADL, activities of daily living; CTCAE, Common Terminology Criteria for Adverse Events; RPED, retinal pigment epithelial detachment

1. Refers to retinopathy in CTCAE v4.0

9 LIFESTYLE AND/OR DIETARY RESTRICTIONS

9.1 Contraception Requirements

9.1.1 Female Subjects

Women of child-bearing potential, defined as all women physiologically capable of becoming pregnant, must use highly effective methods of contraception during dosing and for 16 weeks after stopping treatment.

Highly effective contraception methods include:

a. Total abstinence (when this is in line with the preferred and usual lifestyle of the subject). Periodic abstinence (e.g., calendar, ovulation, symptothermal, post-ovulation methods) and withdrawal are not acceptable methods of contraception.
b. Female sterilization (have had surgical bilateral oophorectomy with or without hysterectomy), total hysterectomy, or tubal ligation at least six weeks before taking study treatment. In case of oophorectomy alone, only when the reproductive status of the woman has been confirmed by follow up hormone level assessment.
c. Sterilization (at least 6 months prior to screening) for male partners. The vasectomized male partner should be the sole partner for that subject.
d. Placement of a hormonal or non-hormonal intrauterine device (IUD) or intrauterine system (IUS) with a documented failure rate of less than 1% per year.

Notes:

a. Double-barrier contraception: condom and occlusive cap (diaphragm or cervical/vault caps) with a vaginal spermicidal agent (foam/gel/cream/suppository) are not considered highly effective methods of contraception.
b. Hormonal based methods (e.g. oral contraceptives) are not considered as highly effective methods of contraception due to potential drug-drug interactions with dabrafenib and/or trametinib (see Section 10.1).
c. Women are considered post-menopausal and not of child bearing potential if they have had 12 months of natural (spontaneous) amenorrhea with an appropriate clinical profile (i.e. age appropriate, history of vasomotor symptoms) or have had surgical bilateral oophorectomy (with or without hysterectomy), total hysterectomy, or tubal ligation at least six weeks ago. In the case of oophorectomy alone, only when the reproductive status of the woman has been confirmed by follow up hormone level assessment is she considered not of child bearing potential.

All menstruating female subjects (as required per local requirements and/or regulations) must have a negative serum pregnancy test within 7 days prior to the first dose of study treatment(s), preferably as close to the first dose as possible. Subjects must have used highly effective contraception since the pregnancy test and agree to use adequate contraception during the study and for 16 weeks following discontinuation of trametinib when taken in combination with dabrafenib, or for 2 weeks following discontinuation of dabrafenib monotherapy.

These allowed methods of contraception are only effective when used consistently, correctly and in accordance with the product label. The investigator is responsible for ensuring subjects understand how to properly use these methods of contraception.

If a subject becomes pregnant during the treatment period of the study, the study treatments should be stopped immediately.

9.1.2 Male Subjects

Male subjects (including those that have had a vasectomy) must use a condom during intercourse while taking dabrafenib and/or trametinib treatment. Once dabrafenib and trametinib are both permanently discontinued, the patient must use a condom for 16 weeks after the last dose of the study treatments. The patient should not father a child during the study treatment period or during the specified time frames after discontinuation of study treatment.

9.2 Lactation

Female subjects who are lactating must discontinue nursing prior to the first dose of study treatments and must refrain from nursing throughout the treatment period and for 4 months following the last dose of study treatments.

9.3 Caffeine and Alcohol

On PK sampling days, subjects will abstain from ingesting caffeine- or xanthine- containing products (e.g., coffee, tea, cola drinks, energy drinks) and alcohol for 24 hours prior to the start of collection of the samples and until collection of the last sample.

9.4 Activity

Subjects will abstain from strenuous exercise (e.g., competitive sports) for 48 hrs prior to each blood collection for clinical laboratory tests. Subjects may participate in light recreational activities during the study.

10 CONCOMITANT MEDICATIONS AND NON-DRUG THERAPIES

The investigator must be informed as soon as possible about any medication taken from the time of screening until 30 days after the last dose of study treatment. Any concomitant medication(s), including dietary supplements, taken during the study will be recorded in the eCRF. The minimum requirement is that drug name, dose, and the dates of administration are to be recorded. Additionally, a complete list of all prior surgical procedures will be recorded in the eCRF.

If emerging data results in a change to the list of permitted/prohibited medications, formal documentation will be provided to the investigative site by the sponsor. Any such changes will be communicated to the investigative sites in the form of a letter. Sites may also refer to any updates to the SPM regarding changes to the list of permitted/prohibited medications.

Questions regarding concomitant medications should be directed to the Medical Lead for clarification.

10.1 Permitted Medications and Non-Drug Therapies

Subjects should receive full supportive care during the study, including transfusions of blood and blood products, and treatment with antibiotics, anti-emetics, anti-diarrheals, and

analgesics, and other care as deemed appropriate, and in accordance with their institutional guidelines. Use of anticoagulants such as warfarin is permitted however, caution should be exercised and additional INR monitoring is recommended when dabrafenib is used concomitantly with warfarin.

Oral (hormonal) contraceptives: While the use of oral contraceptives is not prohibited, the co-administration of dabrafenib and oral contraceptives may decrease the concentration and effectiveness of oral contraceptives and, therefore, the oral contraceptives should not be used as an effective means of birth control by female subjects in this study (see Section 9.1.1). Dabrafenib is an inducer of CYP3A4- and CYP2C9-mediated metabolism and may induce other enzymes including CYP2B6, CYP2C8, and CYP2C19, UDP glucuronosyl transferase (UGT) and transporters; oral contraceptives are affected by the induction of these enzymes. Oral contraceptives, when taken for reasons other than birth control (i.e., acne), may be administered with dabrafenib although there is a risk of lower efficacy.

Radiation: While subjects are on study treatment, palliative radiation therapy is permitted for non-target lesions that are either new or present at baseline. Radiation skin injury has been reported with concurrent use of dabrafenib and radiation. It is recommended that dabrafenib be held for 7 days before and 2 days after radiotherapy in subjects receiving dabrafenib monotherapy or in combination with trametinib. These recommendations can be modified based on the physician's assessment of the risk of radiation skin injury.

10.2 Prohibited Medications and Non-Drug Therapies

The use of illicit drugs and the following medications within 28 days or 5 half-lives, whichever is shorter, prior to **start of treatment** will not be allowed:

- **Antiretroviral drugs**
- **Herbal remedies (e.g., St. John's wort)**
- **Drugs that are strong inhibitors or inducers of CYP2C8 or CYP3A4 (Table27)**

The following medications or non-drug therapies (excluding topical formulations) are also prohibited while on treatment in this study:

- Other anti-cancer therapies
- Other investigational drugs
- Antiretroviral drugs
- Herbal remedies (e.g., St. John's wort)
- ***For MM cohort only***: non-steroidal anti-inflammatory drugs (NSAIDS)

NOTE: Use of NSAIDS by subjects with MM is prohibited as it will worsen renal insufficiency.

- Drugs that are strong inhibitors or inducers of CYP2C8 or CYP3A4 (Table 27)

NOTE: Dabrafenib is metabolized primarily by CYP2C8 and CYP3A4. Co- administration of dabrafenib with ketoconazole, a CYP3A4 inhibitor, or gemfibrozil, a CYP2C8 inhibitor, increased the area under the concentration-time curve (AUC) of dabrafenib by 71% and 47%, respectively. Drugs that are strong inhibitors or inducers of CYP2C8 or CYP3A4 are likely to increase or decrease, respectively, dabrafenib concentrations. Strong inhibitors **or inducers** should only be used under special circumstances (e.g., as a single use for a procedure) while study treatment is interrupted as they may alter dabrafenib concentrations;

consider therapeutic substitutions for these medications. Approval from the Medical Lead is required in these situations.

Table 27 Prohibited Medications

PROHIBITED[1] – strong inducers of CYP3A4 or CYP2C8, since concentrations of dabrafenib may be decreased	
**Class/Therapeutic Area**	**Drugs/Agents**
Antibiotics	Rifamycin class agents (e.g., rifampin, rifabutin, rifapentine),
Anticonvulsant	Carbamazepine, oxcarbazepine phenobarbital, phenytoin, s-mephenytoin
Miscellaneous	Bosentan, St-John's wort
**PROHIBITED[1] – Strong inhibitors of CYP3A4, or CYP2C8 since concentrations of dabrafenib may be increased**	
**Class/Therapeutic Area**	**Drugs/Agents**
Antibiotics	Clarithromycin, telithromycin, troleandomycin
Antidepressant	Nefazodone
Antifungals	Itraconazole, ketoconazole, posaconazole, voriconazole
Hyperlipidemia	Gemfibrozil
Antiretroviral	Ritonavir, saquinavir, atazanavir
Miscellaneous	Conivaptan

1. Topical formulations of prohibited medication(s) are permitted.

10.3 Medications to be used with Caution

The following medications should be used with caution as their concentrations may be altered by dabrafenib or they may alter dabrafenib concentrations:

- Drugs that are moderate inhibitors or inducers of CYP3A and CYP2C8 as they may alter concentrations of dabrafenib.
- Dabrafenib has been shown to induce CYP3A4 and CYP2C9 in vivo using midazolam (CYP3A4 substrate) and S-warfarin (CYP2C9 substrate). Dabrafenib is an in vitro inducer of CYP2B6 and may affect other enzymes such as CYP2C8, CYP2C19, UDP-glucuronyl transferases, and transporters. Co-administration of dabrafenib and medicinal products which are affected by the induction of these enzymes such as **hormonal contraceptives**, warfarin, dexamethasone, antiretroviral agents, or Immunosuppressants may result in decreased concentrations and loss of effectiveness. Where possible consider substitutions of these medicinal products if therapeutic effects cannot be monitored.
- Onset of induction is likely to occur after 3 days of repeat dosing with dabrafenib. Transient inhibition of CYP3A4 may be observed during the first few days of treatment. Upon discontinuation of dabrafenib, concentrations of these sensitive substrates may increase and subjects should be monitored for toxicity and dosage of these agents may need to be adjusted.
- Where possible consider substitutions of these medicinal products if therapeutic effects cannot be monitored. If co-administration of these medications is necessary, investigators should monitor subjects for loss of efficacy or consider substitutions of these medications. A partial list of these medications is provided in Table 28 and in the SPM.

- Therapeutic level dosing of warfarin can be used with approval by the Medical Lead and close monitoring of PT/INR by the site. Exposure decreased by 37% due to enzyme induction when on treatment, thus warfarin dosing may need to be adjusted based upon PT/INR. Consequently, when discontinuing dabrafenib, warfarin exposure may be increased and thus close monitoring via PT/INR and warfarin dose adjustments must be made as clinically appropriate. Prophylactic low dose warfarin may be given to maintain central catheter patency.
- Drugs known to induce QT prolongation interval should be used with caution. Refer to the following link for a list of drugs known to have a risk of QT prolongation: crediblemeds.org/everyone/composite-list-all-qtdrugs/

Table 28 Medications to be used with Caution

USE WITH CAUTION: Moderate inhibitors of CYP3A4, or CYP2C8 since concentrations of dabrafenib may be increased	
**Class/Therapeutic Area**	**Moderate CYP3A4 and CYP2C8 Inhibitors**
Antiarrhythmics	Diltiazem, verapamil
Antibiotic	Erythromycin
Antifungal	Fluconazole
Miscellaneous	Aprepitant
**USE WITH CAUTION: Co-administration of these drugs with study treatment may result in loss of efficacy. Monitor subjects for loss of efficacy or substitute with another medication.**	
**Class/Therapeutic Area**	**CYP3A4, CYP2B6, CYP2C8, CYP2C9, CYP2C19 or Transporter Substrates that May be Affected by Induction**
Analgesics	Alfentanil, buprenorphine, celecoxib, codeine, fentanyl, methadone, oxycodone
Antiarrhythmics	Disopyramide, dronedarone, mexiletine, propafenone, quinidine
Antibiotics	Chloramphenicol, doxycycline, erythromycin, moxifloxacin
Anticoagulants/ Antiplatelets	Cilostazole, warfarin
Anticonvulsants	Divalproex, lamotrigine, valproate, zonisamide
Antidepressants and Antipsychotics	Aripiprazole, bupropion, buspirone, desipramine, haloperidol, mirtazapine, pimozide, quetiapine, trazodone, amitriptyline, clomipramine, imipramine
Antidiabetics	Glyburide, saxagliptin, tolbutamide, nateglinide, pioglitazone, repaglinide, rosiglitazone
Antifungals	Caspofungin, fluconazole, terbinafine
Antihistamines	Astemizole, chlorpheniramine, ebastine
**USE WITH CAUTION: Moderate inhibitors of CYP3A4, or CYP2C8 since concentrations of dabrafenib may be increased**	
Antihypertensives	Amlodipine, diltiazem, felodipine, nifedipine, nilvadipine, nisoldipine, verapamil
Antimigraine Agents	Diergotamine, eletriptan, ergotamine
Corticosteroids	Dexamethasone, methylprednisolone, oral budesonide
Erectile Dysfunction Agents	Sildenafil, tadalafil, vardenafil
HMG-CoA Reductase Inhibitors	Atorvastatin, lovastatin, simvastatin, rosuvastatin, pravastatin

Hypnotics and Sedatives	Alprazolam, brotizolam, diazepam, estazolam, midazolam, triazolam, zolpidem, zopiclone
Immunosuppressants	Everolimus, sirolimus, tacrolimus
Miscellaneous	Aprepitant, cisapride, darifenacin, disopyramide, leflunomide, methohexital, oral contraceptives, quinine, ranitidine, solifenacin, sulfasalazine, tramadol, tolvaptan, chloroquine, zopiclone
Selective Aldosterone Blockers	Eplerenone

Abbreviations: CYP = cytochrome P450; HMG-CoA = 3-hydroxy-3-methylglutaryl-coenzyme A.

11 ADVERSE EVENTS AND SERIOUS ADVERSE EVENTS

The investigator or site staff will be responsible for detecting, documenting and reporting events that meet the definition of an AE or SAE as outlined in Section 11.1 and Section 11.2, respectively.

11.1 Definition of Adverse Event

Any untoward medical occurrence in a subject or clinical investigation subject, temporally associated with the use of a medicinal product, whether or not considered related to the medicinal product.

Note: An AE can therefore be any unfavorable and unintended sign (including an abnormal laboratory finding), symptom, or disease (new or exacerbated) temporally associated with the use of a medicinal product. For marketed medicinal products, this also includes failure to produce expected benefits, abuse, or misuse. Examples of events meeting the definition of an AE include:

- Exacerbation of a chronic or intermittent pre-existing condition including either an increase in frequency and/or grade of the condition
- New conditions detected or diagnosed after study treatment administration even though it may have been present prior to the start of the study
- Signs, symptoms, or the clinical sequelae of a suspected interaction
- Signs, symptoms, or the clinical sequelae of a suspected overdose of either study treatment or a concomitant medication (overdose per se will not be reported as an AE/SAE unless this is an intentional overdose taken with possible suicidal/self-harming intent. This should be reported regardless of sequelae).
- “Lack of efficacy” or “failure of expected pharmacological action” per se is not to be reported as an AE or SAE. However, any signs and symptoms and/or clinical sequelae resulting from “lack of efficacy” will be reported as an AE or SAE, if they fulfill the definition of an AE or SAE.

Events that **do not** meet the definition of an AE include:

- Medical or surgical procedure (e.g., endoscopy, appendectomy); the condition that leads to the procedure is an AE.
- Situations where an untoward medical occurrence did not occur (social and/or convenience admission to a hospital).
- Anticipated day-to-day fluctuations of pre-existing disease(s) or condition(s) present or detected at the start of the study that do not worsen.

- The disease/disorder under study, or expected progression, signs, or symptoms of the disease/disorder being studied, unless more severe than expected for the subject's condition.

11.2 Definition of Serious Adverse Event

A SAE is any untoward medical occurrence that, at any dose:

a. Results in death

NOTE: Death due to disease under study is to be recorded in the Death eCRF and does not need to be reported as an SAE.

b. Is life-threatening

NOTE: The term 'life-threatening' in the definition of 'serious' refers to an event in which the subject was at risk of death at the time of the event. It does not refer to an event, which hypothetically might have caused death, if it were more severe.

c. Requires hospitalization or prolongation of existing hospitalization

NOTE: In general, hospitalization signifies that the subject has been detained (usually involving at least an overnight stay) at the hospital or emergency ward for observation and/or treatment that would not have been appropriate in the physician's office or outpatient setting. Complications that occur during hospitalization are AEs. If a complication prolongs hospitalization or fulfills any other serious criteria, the event is serious. When in doubt as to whether "hospitalization" occurred or was necessary, the AE should be considered serious.

Hospitalization for elective treatment of a pre-existing condition that did not worsen from baseline is not considered an AE.

d. Results in disability/incapacity, or

NOTE: The term disability means a substantial disruption of a person's ability to conduct normal life functions. This definition is not intended to include experiences of relatively minor medical significance such as uncomplicated headache, nausea, vomiting, diarrhea, influenza, and accidental trauma (e.g. sprained ankle) which may interfere or prevent everyday life functions but do not constitute a substantial disruption.

e. Is a congenital anomaly/birth defect.

f. Medical or scientific judgment should be exercised in deciding whether reporting is appropriate in other situations, such as important medical events that may not be immediately life-threatening or result in death or hospitalization but may jeopardize the subject or may require medical or surgical intervention to prevent one of the other outcomes listed in the above definition. These should also be considered serious.

Examples of such events are invasive or malignant cancers, intensive treatment in an emergency room or at home for allergic bronchospasm, blood dyscrasias or convulsions that do not result in hospitalization, or development of drug dependency or drug abuse.

g. All events of possible treatment-induced liver injury with hyperbilirubinemia defined as:

- ALT ≥5 times ULN and bilirubin ≥3 times ULN (>35% direct) <u>or</u> ALT ≥5 times ULN and INR >1.5, if INR is measured, with no existing liver disease
  OR

- ALT ≥8 times ULN **and** bilirubin ≥5 times ULN (>35% direct) or INR >1.5, if INR is measured, with pre-existing liver disease

NOTE: INR measurement is not required and the threshold value stated will not apply to subjects receiving anticoagulants.

NOTE: Bilirubin fractionation is performed if testing is available. If testing is unavailable, record presence of detectable urinary bilirubin on dipstick indicating direct bilirubin elevations and suggesting liver injury. If testing is unavailable and a subject meets the criterion of total bilirubin ≥3 times ULN, if no existing liver disease or ≥3 times ULN if pre-existing liver disease; the event is still reported as an SAE. If INR is obtained, include values on the SAE form. Elevations in INR >1.5 suggest severe liver injury.

h. Protocol-specific SAEs:

- cuSCC (Section 8.2.2.1), new primary melanomas (Section 8.2.2.2) **and non-cutaneous malignancies (Section 8.2.2.3)** with the exception of basal cell carcinoma (BCC). BCC should be reported as an AE or SAE based on the discretion of the investigator.
- LVEF that meets the dose interruption criteria (Section 8.1.1.2): absolute LVEF decreases of >10% from baseline (not relative decrease) and below the LLN
- Liver chemistry events of ALT ≥3xULN **and** bilirubin ≥2xULN (>35% direct bilirubin) or ALT ≥3xULN **and** INR>1.5, if INR measured which may indicate severe liver injury (possible 'Hy's Law') (Section 8.1.3.1)
- RPED also referred to as central serous retinopathy (CSR) or RVO (Section 8.2.7)
- Pyrexia accompanied by hypotension or dehydration requiring IV fluids or renal insufficiency or severe rigors/chills in the absence of an obvious infectious cause (Section 8.2.3).

11.3 Laboratory and Other Safety Assessment Abnormalities Reported as AEs and SAEs

Any abnormal laboratory test results (hematology, clinical chemistry, or urinalysis), or other safety assessments (e.g., ECGs, radiological scans, vital signs measurements) including those that worsen from baseline, and events felt to be clinically significant in the medical and scientific judgment of the investigator are to be recorded as an AE or SAE, in accordance with the definitions provided.

In addition, an associated AE or SAE is to be recorded for any laboratory test result or other safety assessment that led to an intervention, including permanent discontinuation of study treatment(s), dose reduction, and/or dose interruption/delay.

However, any clinically significant safety assessments that are associated with the underlying disease, unless judged by the investigator to be more severe than expected for the subject's condition, are not to be reported as AEs or SAEs.

11.4 Cardiovascular Events

Investigators will be required to fill out event specific data collection tools for the following AEs and SAEs:

- Myocardial infarction/unstable angina

- Congestive heart failure
- Arrhythmias
- Valvulopathy
- Pulmonary hypertension
- Cerebrovascular events/stroke and transient ischemic attack
- Peripheral arterial thrombosis
- Deep Venous Thrombosis
- Revascularization

This information should be recorded within 1 week of when the AE/SAE(s) is first reported.

11.5 Death Events

In addition, all deaths, whether or not they are considered SAEs, will require a specific death data collection tool to be completed. The death data collection tool includes questions regarding cardiovascular (including sudden cardiac death) and non- cardiovascular death.

This information should be recorded within 1 week of when the death is first reported.

11.6 Disease-Related Events and/or Disease-Related Outcomes Not Qualifying as SAEs

An event which is part of the natural course of the disease under study (i.e., disease progression or hospitalization due to disease progression) does not need to be reported as an SAE. Death due to disease under study is to be recorded on the Death eCRF form. However, if the underlying disease (i.e., progression) is greater than that which would normally be expected for the subject, or if the investigator considers that there was a causal relationship between treatment with study treatment(s) or protocol design/procedures and the disease progression, then this must be reported as an SAE.

11.7 Time Period and Frequency of Detecting AEs and SAEs

The investigator or site staff is responsible for detecting, documenting and reporting events that meet the definition of an AE or SAE.

Adverse events (AEs) will be collected from the time the first dose of study treatment(s) is administered until 30 days following discontinuation of study treatment(s) regardless of initiation of a new cancer therapy or transfer to hospice.

Serious adverse events (SAEs) will be collected over the same time period as stated above for AEs. In addition, any SAE assessed as related to study participation (e.g., protocol-mandated procedures, invasive tests, or change in existing therapy), study treatment or concomitant medication(s) must be recorded from the time a subject consents to participate in the study up to and including any follow-up contact.

After discontinuation of study treatment(s), the investigator will monitor all AEs/SAEs that are ongoing until resolution or stabilization of the event or until the subject is lost to follow-up. At any time after 30 days the investigator may report any AE that they believe possibly related to study treatment(s).

11.8 Method of Detecting AEs and SAEs

Care will be taken not to introduce bias when detecting AEs and/or SAEs. Open-ended and non-leading verbal questioning of the subject is the preferred method to inquire about AE occurrence. Appropriate questions include:

- "How are you feeling?" or "How does your child seem to feel?"
- "Have you had any (other) medical problems since your last visit/contact?" or "Has your child had any (other) medical problems or seem to act differently in any way since his/her last visit/contact?"
- "Have you taken any new medicines, other than those provided in this study, since your last visit/contact?" or "Has your child needed to take any medicines, other than those provided in this study, since his/her last visit/contact?"

11.9 Recording of AEs and SAEs

When an AE/SAE occurs, it is the responsibility of the investigator to review all documentation (e.g., hospital progress notes, laboratory, and diagnostics reports) relative to the event. The investigator will then record all relevant information regarding an AE/SAE in the appropriate data collection tool.

It is not acceptable for the investigator to send photocopies of the subject's medical records to the sponsor in lieu of completion of the AE/SAE data collection tool.

However, there may be instances when copies of medical records for certain cases are requested by the sponsor. In this instance, all subject identifiers, with the exception of the subject number, will be blinded on the copies of the medical records prior to submission of to the sponsor.

The investigator will attempt to establish a diagnosis of the event based on signs, symptoms, and/or other clinical information. In such cases, the diagnosis will be documented as the AE/SAE and not the individual signs/symptoms.

Subject-completed health outcomes questionnaires and the collection of AE data are independent components of the study. Responses to each question in the health outcomes questionnaire will be treated in accordance with standard scoring and statistical procedures detailed by the scale's developer. The use of a single question from a multidimensional health survey to designate a cause-effect relationship to an AE is inappropriate.

11.10 Evaluating AEs and SAEs

11.10.1 Assessment of Intensity

The investigator will make an assessment of intensity for each AE and SAE reported during the study and will assign it to one of the following categories:

- Mild: An event that is easily tolerated by the subject, causing minimal discomfort and not interfering with everyday activities.
- Moderate: An event that is sufficiently discomforting to interfere with normal everyday activities.
- Severe: An event that prevents normal everyday activities.

An AE that is assessed as severe will not be confused with an SAE. Severity is a category utilized for rating the intensity of an event; and both AEs and SAEs can be assessed as severe.

An event is defined as 'serious' when it meets at least one of the pre- defined outcomes as described in the definition of an SAE.

11.10.2 Assessment of Causality

The investigator is obligated to assess the relationship between study treatment and the occurrence of each AE/SAE. A "reasonable possibility" is meant to convey that there is facts/evidence or arguments to suggest a causal relationship, rather than a relationship cannot be ruled out. The investigator will use clinical judgment to determine the relationship. Alternative causes, such as natural history of the underlying diseases, concomitant therapy, other risk factors, and the temporal relationship of the event to the study treatment will be considered and investigated. The investigator will also consult the IB and/or Product Information, for marketed products, in the determination of his/her assessment.

For each AE/SAE the investigator must document in the medical notes that he/she has reviewed the AE/SAE and has provided an assessment of causality.

11.11 Follow-Up of AEs and SAEs

After the initial AE/SAE report, the investigator is required to proactively follow each subject at subsequent visits/contacts. All AEs and SAEs will be followed until resolution, until the condition stabilizes, until the event is otherwise explained, or until the subject is lost to follow-up.

The investigator is obligated to perform or arrange for the conduct of supplemental measurements and/or evaluations as may be indicated or as requested by the sponsor to elucidate as fully as possible the nature and/or causality of the AE or SAE. The investigator is obligated to assist. This may include additional laboratory tests or investigations, histopathological examinations or consultation with other health care professionals. If a subject dies during participation in the study or during a recognized follow-up period, the investigator will provide the sponsor with a copy of any post- mortem findings, including histopathology.

New or updated information will be recorded in the originally completed data collection tool. The investigator will submit any updated SAE data to the sponsor within the designated reporting time frames.

11.12 Prompt Reporting of SAEs to Sponsor

Once the investigator determines that an event meets the protocol definition of an SAE, the SAE will be reported to the sponsor within 24 hours. Any follow-up information on a previously reported SAE will also be reported to the sponsor within 24 hours.

If the investigator does not have all information regarding an SAE, he/she will not wait to receive additional information before notifying the sponsor of the event and completing the appropriate data collection tool. The investigator will always provide an assessment of causality at the time of the initial report as described in Section 11.10.2, Assessment of Causality.

The primary mechanism for reporting SAEs to the sponsor will be the electronic data collection tool. If the electronic system is unavailable for greater than 24 hrs, the site will use the paper SAE data collection tool and fax it to the Medical Lead. Then the site will enter the SAE data into the electronic system as soon as it becomes available. After the study is

completed at a given site, the electronic data collection tool (e.g., InForm system) will be taken off-line to prevent the entry of new data or changes to existing data. If a site receives a report of a new SAE from a study participant or receives updated data on a previously reported SAE after the electronic data collection tool has been taken off-line, the site can report this information on a paper SAE form or to their sponsor protocol contact by telephone.

The sponsor contacts for SAE receipt can be found at the beginning of this protocol on the Sponsor/Medical Lead Contact Information page.

11.13 Regulatory Reporting Requirements for SAEs

Prompt notification of SAEs by the investigator to the sponsor is essential so that legal obligations and ethical responsibilities towards the safety of subjects are met.

The sponsor (currently Novartis) has a legal responsibility to notify both the local regulatory authority and other regulatory agencies about the safety of a product under clinical investigation. The sponsor will comply with country specific regulatory requirements relating to safety reporting to the regulatory authority, IRB/IEC and investigators.

Investigator safety reports are prepared for suspected unexpected serious adverse reactions according to local regulatory requirements and Novartis policy and are forwarded to investigators as necessary. An investigator who receives an investigator safety report describing a SAE(s) or other specific safety information (e.g., summary or listing of SAEs) from the sponsor will file it with the IB and will notify the IRB/IEC, if appropriate according to local requirements.

[redacted]

[redacted]

13 PREDICTIVE AND PHARMACODYNAMIC BIOMARKERS

13.1 Tumor Tissue for BRAFV600E Mutation Pre-Screening

Only those subjects whose samples test positive for the BRAF V600E mutation will be eligible for this study. Subjects may be enrolled and started on study treatment if the local BRAF result is positive for the BRAF V600E mutation. If a local test result is not available, a tumor sample reflective of the current disease will be collected at pre- screening to test for BRAF V600E mutation status.

Confirmatory Testing: All local test results for BRAF V600E mutation will be subject to central confirmation using a sponsor designated central reference lab.

1. ***For the solid tumor histology cohorts***, an archived or fresh tumor tissue sample must be submitted to the central reference laboratory.
2. ***For the HCL and MM cohorts***, a fresh BM aspirate and a corresponding peripheral blood sample are required to be submitted to the central reference laboratory.

   NOTE: An archived tissue sample (such as a FFPE tissue sample from a BM biopsy), BM core samples, and BM clot samples are **NOT** acceptable for BRAF V600E testing. If a BM aspirate cannot be obtained due to a dry tap, please contact the Medical Lead.

Details on sample collection, processing, storage and shipping procedures are provided in the lab manual.

13.2 Predictive Biomarker Research

Biomarker analyses may be conducted to identify predictive markers indicating sensitivity to the IP or characterize resistance acquired upon exposure to the IP. These may include testing for DNA changes including gene mutations and copy number variations that may predict response (e.g., mutations in MAPK, PI3K or related pathway genes) by comparing quantities of protein or RNA before and after treatment.

Analysis of novel candidate biomarkers of the biological/clinical response may include:

- Analysis of additional archival tumor or tumor biopsy specimens to identify genetic aberrations (e.g., DNA mutations, amplifications, and deletions).
- RNA or miRNA quantification from archival or fresh tumor biopsies.
- Analysis of circulating biomarkers related to the MAPK pathway.
- Proteome and protein analysis of archival tumor specimens, fresh tumor biopsy specimen or blood sample.

The analysis of markers may depend on emerging pre-clinical and clinical biomarker data. Pre- and post-dose tumor tissue samples or BM aspirate samples may be analyzed for the PD markers of the MAPK pathway such as pERK, pMEK p27 and Ki67 among others characterize a PD response or correlate with a response to treatment. Once preliminary PK

and PD data have been reviewed, the planned tumor biopsy collection times may be revised and provided to sites in writing. Any changes in the sample collection times will not constitute a protocol amendment.

If the subject provides consent, tumor biopsy samples or BM aspirate samples will be collected as indicated in the Time and Events Table (Section 7.1). Baseline tumor tissue samples should be obtained from lesion(s) not required for disease assessment. Sample collection requirements may be changed once sufficient evaluable paired samples have been obtained.

Note: A fresh tumor tissue sample submitted for BRAF testing or any tumor sample remaining after central confirmation of BRAF V600E testing may substitute for baseline sample for exploratory analysis if the subject provides consent.

Details on the collection of tumor tissue samples or BM aspirate samples, processing, storage and shipping procedures are provided in the lab manual.

All samples will be retained for a maximum of 15 years after the last subject completes the study.

13.2.1 Peripheral Blood Sample Collection and Analysis

If the subject provides consent, peripheral blood samples will be used as a surrogate tissue to explore mutation profiles (circulating free DNA [cfDNA] and RNA), protein levels and markers of the immune system.

A 7.5 mL peripheral blood sample for cfDNA as well as a 10 mL peripheral blood sample for PD plasma and PBMC will be collected from subjects to match the predictive biomarker tumor tissue sample or BM aspirate sample collected as indicated in the Time and Events Table (Section 7.1). The timing of the collections may be adjusted on the basis of new information in order to ensure optimal evaluation of the PD endpoints. The decision to analyze for specific mutations will depend upon the type of tumor.

Details on the blood sample collection, processing, storage and shipping procedures are provided in the lab manual.

13.3 Exploratory Ex Vivo Assay: HCL Cohort Only

Blood samples (approximately 25 mL) will be collected from HCL subjects at the time point indicated in the Time and Events Table (Section 7.1). The investigator should use their clinical judgment in determining whether it is appropriate for a subject enrolled in the HCL Cohort to have this additional blood drawn. In addition, if the IEC or IRB does not approve the site for participation in the sub-study, no subjects at the sites will be included in the sub-study.

The HCL cells will be isolated from these samples and tested using two exploratory ex vivo assays. One assay will identify the HCL sensitivity to dabrafenib and trametinib as determined by cytotoxicity assay. The second assay will determine the ability of dabrafenib and trametinib to block phosphorylation of BRAF and MEK, respectively, using a western blot assay. Analysis will identify the utility of these assays as a potential predictive assay for these drugs in subjects with HCL.

14 PHARMACOGENETICS

Information regarding optional pharmacogenetic (PGx) research is included in Appendix 4.

[redacted]

16 COMPLETION OR WITHDRAWAL OF SUBJECTS

16.1 Screen Failures

Screen failures are defined as subjects who consent to participate in the clinical trial but are never subsequently enrolled. In order to ensure transparent reporting of screen failure subjects, meet the Consolidated Standards of Reporting Trials (CONSORT) publishing requirements, and respond to queries from Regulatory authorities, a minimal set of screen failure information is required including Demography, Screen Failure details, Eligibility Criteria, and any SAEs.

16.2 Criteria for Permanent Discontinuation of Study Treatments

Subjects will receive study treatment(s) until disease progression, death or unacceptable toxicity (AE or SAE), including meeting the stopping criteria for liver chemistry defined in Section 8.1.3.1 or for other toxicities described in Section 8.2.

However, cases in which the subject has met the criteria for disease progression (e.g., a mixed response or slow progression) and the investigator determines that the subject may still clinically benefit from continuing study treatment(s) beyond radiographic disease progression, study treatment(s) may continue if:

- the subject is willing to continue study treatment(s), and
- consultation and approval of the Medical Lead is obtained.

Once a subject has permanently discontinued from study treatment, the subject will not be allowed to be retreated.

Study treatment(s) may be discontinued if any of the following occurs:

- Deviation(s) from the protocol
- Request of the subject or their proxy
- Investigator's discretion
- Disease progression (unless subject is receiving clinical benefit and continued treatment is determined to be appropriate)
- Unacceptable toxicity (AE or SAE) that is considered by the investigator and/or Medical Lead to warrant permanent discontinuation of study treatment(s)
- Intercurrent illness that prevents further administration of study treatment(s)
- Subject withdrawal of consent for further treatment and/or data collection
    - If subject withdraws consent for further treatment, the subject should return for Post-Treatment Follow-up Visit as indicated in the Time and Events Table (Section 7.1).
    - If subject withdraws consent for further treatment and data collection, then no additional study visits or data collection should occur.
- Death
- Subject is lost to follow-up
- Study is closed or terminated by the Sponsor

The primary reason study treatment(s) were permanently discontinued must be documented in the subject's medical record and the eCRF. If the subject voluntarily discontinues from

treatment due to toxicity, 'AE/SAE' will be recorded as the primary reason for permanently discontinuation in the eCRF.

16.3 Follow-Up Assessments after Discontinuation of Study Treatment or Study Withdrawal

If study treatment is permanently discontinued **without** disease progression:

- the subject will complete the Post-Treatment Follow-Up Visit (see Time and Events Table) within 28 days (+7 days) after last dose of study treatment and prior to starting a new anti-cancer treatment or treatment with an investigational agent.
- the subject will continue to have disease assessments performed every 12 weeks until disease progression, initiation of a new anti-cancer treatment, or death, whichever comes first.

If study treatment is permanently discontinued **with** disease progression and/or subject is withdrawn from the study:

- The subject must make every effort to complete the Post-Treatment Follow-Up Visit (see Time and Events Table [Section 7.1]) within 28 days (+7 days) after last dose of study treatment and prior to starting any other anti-cancer treatment or treatment with an investigational agent.

All subjects permanently discontinuing study treatment will have a dermatologic exam performed monthly (±7 days) for the first 6 months following discontinuation of study treatment. Subjects will be followed concurrently for survival and subsequent anti-cancer treatment every 3 months (±14 days) until they die, withdraw consent, are lost to follow, or up to a time period that is dependent on the criteria defined in Section 16.4. When subjects are no longer required to be followed for survival, they will be notified accordingly.

If subjects are unable to attend clinic visits during survival follow-up, contact may be made via another form of communication (e.g., phone, mail, etc.).

16.4 Subject Completion

A subject is considered to have discontinued the study if the subject is lost to follow-up or withdraws consent or another reason exists that prevents additional data from being collected on the subject.

A subject is considered to have completed the study if they die while receiving study treatment(s) or during the extended follow-up period. The cause of death should be documented in the eCRF.

Data collection will continue until a subject has completed or discontinued the study.

16.5 Study Completion

The study will be considered complete at a date that would represent a minimum follow up of approximately 2 years for all subjects enrolled.

Subjects who continue to benefit from study treatment at the time of study completion or closure may transition to an alternative supply of MEK and/or BRAF inhibitor through commercial supply or post trial access mechanism such as a rollover study, if available, at the time of study completion. In the event subjects are transitioned to a rollover study, only safety will be followed.

16.6 Subject Withdrawal Procedures

If subject is withdrawn from the study due to an AE or SAE, subject should be followed until:

- AE or SAE stabilizes or resolves
- AE or SAE is otherwise explained
- Subject is lost to follow-up

If subject is withdrawn from the study with an ongoing Grade 3 or 4 laboratory abnormality:

- Subject should be followed until laboratory abnormality improves to Grade 2 or less, unless condition is unlikely to improve due to underlying disease, or resolves

Should a subject fail to attend the clinic for a required study visit, the site should attempt to contact the subject and re-schedule the missed visit as soon as possible. The site should also counsel the subject on the importance of maintaining the assigned visit schedule and ascertain whether or not the subject wishes to and/or should continue in the study based on previous non-compliance. In cases where the subject does not return for the rescheduled visit or cannot be reached to reschedule the missed visit, the site should make every effort to regain contact with the subject (three telephone calls and if necessary a certified letter to the subject's last known mailing address) so that they can appropriately be withdrawn from the study. These contact attempts should be documented in the subject's medicalrecord. Should the subject continue to be unreachable, then and only then will he/she be considered to have withdrawn from the study with a primary reason of "Lost to Follow-up". For all other subjects withdrawing from the study, an alternative reason for discontinuation should be recorded in the eCRF.

17 DATA MANAGEMENT

For this study, subject data will be entered into the sponsor-defined eCRFs, transmitted electronically to the sponsor or designee and combined with data provided from other sources in a validated data system.

Management of clinical data will be performed in accordance with applicable Novartis standards and data cleaning procedures to ensure the integrity of the data, e.g., removing errors and inconsistencies in the data. Adverse events (AEs) and concomitant medications terms will be coded using Medical Dictionary for Regulatory Activities (MedDRA) and a custom drug dictionary. An appropriate medical dictionary that covers all approved drugs in studies where Japan is participating will be referenced. Electronic CRFs (eCRFs), including queries and audit trails, will be retained by the sponsor and copies will be sent to the investigator to maintain as the investigator copy.

[redacted]

[redacted]

18 DATA ANALYSIS AND STATISTICAL CONSIDERATIONS

18.1 Hypotheses and Treatment Comparisons

Subjects with rare BRAF V600E mutation-positive cancers will be enrolled, with the primary objective of evaluating clinical activity according to ORR. Response assessment for most solid tumors (ATC, BTC, GIST, NGGCT/NSGCT and adenocarcinoma of small intestine) will be evaluated by RECIST version 1.1 while all other tumors (HCL, MM and WHO Grade 1 or 2 glioma or WHO Grade 3 or 4 glioma) will use the appropriate established response criteria.

To further investigate clinical activity and compensate for the small sample sizes across the histologies, an adaptive design utilizing a Bayesian hierarchical model will be employed. Multiple interim evaluations of the accumulating data to determine if one or morehistologic cohorts should discontinue enrollment early due to either success or futility will be incorporated. Interim and final evaluations will be based on a hierarchical model that borrows information in a limited way from histologies that demonstrate similar treatment effects based on the accumulated trial data. Traditional estimates based on independent analyses will also be provided.

π_j is the true response rate for histology *j,* where *j = 1,…, 9* and indexes the nine histologies. C_j is the historical control response rate for the jth histology. The historical controls vary by histology and are provided in Table 29. The study is powered to detect a high clinically meaningful response rate and is based on the hierarchical model assessment of whether there is sufficiently high probability that π_j exceeds C_j. The posterior probability that the ORR for a given histology is greater than C_j will be computed according to the following comparison:

$$P\ (\pi_j > C_j \mid \text{current data}) \text{ for the j-th histology}$$

If this posterior probability is sufficiently low within a given histology, then this will provide insufficient evidence to suggest that the ORR is greater than its respective historical control. Conversely, if a sufficiently high posterior probability is observed, this will provide evidence that the ORR is greater than the historical control, and the combination arm will be declared efficacious in that histology. Thresholds for decision- making are defined in Section 18.3.

Historical control and clinically meaningful response rate varies by histology and is provided in Table 29.

Table 29 Historical Control and Clinically Meaningful Response Rates for Each Histology

Histology	Historical Control (C_j)	Clinically meaningful ORR
ATC	15%	60%
BTC	10%	50%
GIST	10%	50%
HCL	10%	50%
WHO Grade 1 or 2 Glioma	10%	50%
WHO Grade 3 or 4 Glioma (high-grade)	10%	50%
MM	15%	60%
NSGCT/NGGCT	25%	75%
Small Intestine Adenocarcinoma	10%	50%

Abbreviations: ATC, anaplastic thyroid cancer; BTC, biliary tract cancer; C, control; GIST, gastrointestinal stromal tumor; HCL, hairy cell leukemia; MM, multiple myeloma; NGGCT, non-

geminomatous germ cell tumor; NSGCT, non- seminomatous germ cell tumor; WHO, World Health Organization

Full details regarding the hierarchical modeling framework are in Appendix 6.

18.2 Sample Size Considerations

18.2.1 Sample Size Assumptions

Each cohort of BRAF V600E mutation-positive tumor type of a given histology will enroll a maximum of 25 subjects **in the primary analysis cohort as defined in Section 5.1.** If all histologies enroll the maximum of 25 subjects, this will result in no more than 225 subjects in the primary analysis cohort.

Enrollment into specific histology cohorts may be halted early based on results from interim analyses incorporating emerging response data. Response data from a minimum of 5 subjects will be required in a histologic cohort before it may discontinue enrollment for futility and response data from a minimum of 10 subjects will be required before discontinuing a histologic cohort for efficacy. **If a cohort closes early for efficacy at an interim analysis, a histology specific expansion cohort (see Section 5.2) may be opened to allow additional enrollment. At** the final analysis and after the study has been closed, a minimum of 2 subjects will be required in a histologic cohort in order to meet statistical success at the final analysis. See Section 18.3.2 for more details.

Simulation studies were conducted to evaluate the performance of the design under various assumptions for the distribution of true ORRs across the histologic cohorts and accounting for the anticipated small sample sizes (refer to Section 18.4.1.2). Operating characteristics including power, type I error, estimation of the ORR, and the probability of halting enrollment at interim analyses were assessed.

When the treatment effects are similar across all histologies, the design maintains power 84% to 98% and type I error rate $\leq$0.04. Estimation efficiencies due to borrowing result in very good operating characteristics in these situations, even in histologies with very low sample sizes.

In mixed scenarios where some histologies show large treatment effects and others do not, the type I error can range from 0.03 to 0.14 while the power ranges from 55%- 96%. This variation is hugely dependent upon the expected sample sizes of the individual histologies as well as the overall distribution of treatment effects across the histologies.

Full presentation and discussion of the simulation parameters and corresponding simulation results are in Section 18.4.

18.2.2 Sample Size Sensitivity

Sample size sensitivities are included in Section 18.4.2.

18.2.3 Sample Size Re-estimation

No sample size re-estimation will be performed.

18.3 Data Analysis Considerations

Data will be listed and summarized according to Novartis Integrated Data Standard Library (IDSL) reporting standard where applicable. Complete details will be provided in the Reporting and Analysis Plan (RAP).

18.3.1 Analysis Populations

As the design for the study calls for repeated interim analyses to evaluate the accumulating efficacy data, it is necessary to shift the focus of the analysis population for efficacy depending upon the time at which the data are being analyzed. Table 30 presents the population of interest based on the timing of the analysis for both efficacy and safety; accompanying definitions are provided beneath the table.

Table 30 Definition of Population for Specific Analysis

		Analysis Period	
	**Analysis Intent**	**Interim Analyses**	**Final Analysis**
**Efficacy**	**Primary**	**ITT/Evaluable**	**BRAF V600E**
	**Supportive**	**BRAF V600/ Evaluable**	**ITT**
**Safety**	**Primary**	**ATS**	**ATS**

The BRAF V600E Population is defined as all enrolled subjects regardless of whether or not treatment was administered, who obtain positive verification of the BRAF V600E mutation from a certified central reference laboratory.

The Intent-to-Treat (ITT) population is defined as all enrolled subjects regardless of whether or not treatment was administered.

The All-Treated Subjects (ATS) population is defined as all subjects who receive at least one dose of dabrafenib (GSK2118436) or trametinib (GSK1120212).

The Intent-to-Treat (ITT)/Evaluable population is defined as all ITT subjects in the primary analysis cohort who are also evaluable according to the evaluability defined in the interim analysis RAP.

The BRAF V600E /Evaluable Population is defined as all BRAF V600E subjects in the primary analysis cohort who are also evaluable according to the evaluability defined in the interim analysis RAP.

18.3.2 Interim Analysis

Interim data will be evaluated to monitor efficacy and safety. Enrollment may be stopped early for futility or efficacy, should various criteria occur based on accrued data. The decision criteria for futility and efficacy are described below.

18.3.2.1 Early Stopping for Futility/Harm

For each histology, the therapeutic effect of the combination therapy will be declared insufficient and enrollment terminated if a minimum of 5 subjects have response data available and the posterior probability that the ORR is greater than its corresponding historical control (C_j) is sufficiently low (<30%) based on the hierarchical model. That is,

$$P(\pi_j > C_j \mid \text{current data}) < 30\%, \text{ for the j-th histology.}$$

18.3.2.2 Early Stopping for Efficacy

For each histology, the combination therapy will be declared efficacious and enrollment terminated if a minimum of 10 subjects have response data available and the posterior probability that the ORR exceeds its corresponding historical control (C_j) is sufficiently high (>95%) based on the hierarchical model. That is,

$$P(\pi_j > C_j \mid \text{current data}) > 95\%, \text{ for the j-th histology.}$$

18.3.3 Final Analyses

The final primary analysis for ORR will occur once all subjects in the primary analysis cohort have either discontinued treatment or have received treatment for at least 8 months, whichever comes first. Secondary efficacy endpoints will likely not be mature at this time, but will still be reported. Subsequent analyses for secondary endpoints will occur at a date that would represent a minimum follow up of approximately 2 years for all subjects enrolled.These latter analyses will provide mature estimates/incidence for duration of response, PFS, OS, andsafety.

18.3.3.1 Safety Analyses

Safety data will be presented in tabular and/or graphical format and summarized descriptively according to Novartis' IDSL standards.

The ATS population will be used for the analysis of safety data. In general, safety data will be summarized by histology and across all cohorts

Complete details of the safety analyses will be provided in the RAP.

18.3.3.1.1 Adverse Events

Adverse events (AEs) will be coded using the standard MedDRA and grouped by system organ class. Adverse events (AEs) will be graded by the investigator according to the CTCAE, version 4.0.

Events will be summarized based on frequency and proportion of total subjects, by system organ class and preferred term. Separate summaries will be given for all AEs, treatment-related AEs, AEs by toxicity grade, SAEs and AEs leading to discontinuation of study treatment and dose modification.

Adverse events (AEs) of special interest will be identified and additional summaries provided.

The incidence of deaths and the primary cause of death will be summarized.

18.3.3.1.2 Clinical Laboratory Evaluations

Hematology and clinical chemistry data will be summarized by each scheduled assessment according to CTCAE, version 4.0, grade. The proportion of values lying outside the reference range will also be presented for laboratory tests that are not graded because there are no associated CTCAE grading criteria. Summaries will include data from scheduled assessments only, and all data will be reported according to the nominal visit date for which it was recorded (i.e., no visit windows will be applied). Unscheduled data will be included

in "overall" and "any post-screening" summaries which will capture a worst case across all scheduled and unscheduled visits post first dose of study treatment.

18.3.3.1.3 Other Safety Measures

The results of scheduled assessments of vital signs, 12-lead ECG, ECHO and ECOG performance status will be summarized. Summaries will include data from scheduled assessments only. All data will be reported according to the nominal visit date for which it was recorded; no visit windows will be applied. All data will be listed.

18.3.3.2 Efficacy Analyses

ORR will be analyzed using an integrated analysis with the hierarchical model as well as analyzed independently **for each histology**.

At the final analysis a histology will be declared active if the posterior probability that the ORR exceeds its corresponding historical control (10%) is sufficiently high (>92%)

based on the hierarchical model. That is,

$$P(\pi_j > C_j \% \mid \text{current data}) > 92\%, \text{ for the j-th histology.}$$

The observed ORR and the estimated ORR based on the hierarchical model will be reported for each histology. Confirmed ORR will be calculated as the proportion of subjects that have a confirmed response relative to the total number of subjects enrolled. Standard response assessments vary by histology; the following subcategories for each histology will be considered a 'response' to therapy when computing confirmed ORR.

Table 31 Response Subcategories Defined as 'Response' by Histology

Histology	Response Criteria	Response Subcategories defined as 'Response'
ATC, BTC, GIST, Small Intestine Adenocarcinoma	RECIST 1.1	CR, PR
NSGCT/NGGCT	RECIST 1.1.<br>Tumor Markers: β-HCG and AFP	Marker-Negative CR, Marker-Positive CR, Marker-Negative PR, Marker-Positive PR
MM	IMWG Uniform Response Criteria for MM	sCR, CR, PR, VGPR
HCL	Adapted from NCCN guidelines, Consensus Resolution Criteria and previous studies definition	CR±MRD, PR
WHO Grade 1 and 2 Gliomas	Response Assessment Criteria for WHO Grade 1 or 2 Gliomas: RANO Working Group	CR, PR, MR
WHO Grade 3 and 4 Gliomas	Updated Response Assessment Criteria for WHO Grade 3 or 4 Gliomas: RANO Working Group	CR, PR
Abbreviations: AFP, alpha-fetoprotein; ATC, anaplastic thyroid cancer; β-hCG, beta-human chorionic gonadotropin; BTC, biliary tract cancer; CR, complete response; GIST, gastrointestinal stromal tumor; HCL, hairy cell leukemia; IMWG, International Myeloma Working Group; MM, multiple myeloma; MR, minor response; NCCN, National Comprehensive Cancer Network; NGGCT, non-geminomatous germ cell tumor; NSGCT, non-seminomatous germ cell tumor; PR, partial response; RANO, Response Assessment for Neuro-Oncology; RECIST, Response Evaluation Criteria in Solid Tumors; sCR, stringent complete response; VGPR, very good partial response; WHO, World Health Organization		

Further details on response confirmation criteria for each histological cohort will be provided in the RAP. Subjects with unknown or missing response will be treated as non- responders; that is, they will be included in the denominator when calculating the percentage.

The observed confirmed ORR will be reported at the final analysis for each histology. The estimates along with 95% confidence interval (CI) based on a normal approximation will be provided.

Bayesian inference based on summary statistics from the posterior distributions of each ORR will be reported. The posterior mean and posterior 2.5% and 97.5% percentiles of the ORR will be calculated for each histology. In addition, the posterior probability that the ORR exceeds its corresponding historical control will be reported for each histology.

Duration of response for the subset of subjects who have a confirmed response (See Table 31) is defined as the time from first documented evidence of response until the first documented sign of disease progression or death. Duration of response will be summarized descriptively for each histology, if data warrant, using Kaplan-Meier medians and quartiles. Details on rules for censoring will be provided in the RAP.

Progression-free survival (PFS) is defined as the time from the date of enrollment to the earliest date of progression or death. Progression-free survival (PFS) will be summarized by histology, if data warrant, using Kaplan-Meier quartile estimates along with 2-sided 95% CIs. If the subject does not have a documented date of progression or death, PFS will be censored at the date of the last adequate assessment. Further details on rules for censoring will be provided in the RAP. Progression-free survival (PFS) will be reported at the time of final primary analysis for overall response rate (ORR), regardless of the maturity of the data, and then updated at the final analysis.

Overall Survival (OS) is defined as the time from the date of enrollment to the date of death due to any cause. Censoring will be performed using the date of last known contact for those who are alive at the time of analysis. Overall survival (OS) will be summarized by histology, if data warrant, at the time of final primary analysis for overall response rate (ORR), regardless of the maturity of the survival data, and then updated at the final analysis.

Further details including possible sensitivity analyses will be provided in the RAP.

[redacted]

18.3.3.4 Tumor Biopsy/Blood Biomarkers

The study will analyze blood from subjects and any tumor tissue obtained to understand the genetic and proteomic mechanism(s) of response and/or resistance to dabrafenib and trametinib. As data warrant, correlation analysis may be performed to explore the relationship between biomarker data (e.g., change from baseline) and tumor response.

[redacted]

[redacted]

18.3.3.6 Pharmacogenetic Analyses

Further details on PGx analyses are addressed in Appendix 4.

[redacted]

18.4 Simulations and Design Operating Characteristics

18.4.1 Simulation Description

Extensive simulations have been conducted to develop and understand the performance of the adaptive design; the hierarchical model including clustering mechanism, interim monitoring, and decision criteria. Subject enrollments into the expansion cohorts as defined in Section 5.2 are not taken into consideration in the simulations presented in this section.

18.4.1.1 Software Details

Simulations were conducted by Berry Consultants, with post-processing in R 2.15.2. For each assumed scenario, 1,000 sets of trials were simulated. Posterior distributions were estimated via Markov chain Monte Carlo methods using 50,000 iterations for each analysis, discarding the first 1,000 iterations for each analysis as burn-in.

18.4.1.2 Trial Sample Sizes

Sample size requirements for halting enrollment at interim analyses for simulations is based on the number of subjects enrolled; while in practice, they will be based on the number of subjects with available response data. This discrepancy is due to software feasibility, but should not have a significant impact on operating characteristics of the design. Simulations assumed interim analyses occurred every 12 weeks, with the first interim analysis occurring once 10 subjects have been enrolled. The time from subject entry until the response assessment was performed is assumed to be 8 weeks. The maximum length of time for each study is 156 weeks ±6 weeks (roughly 3 years).

Although actual enrollment may vary, predicted enrollment during a 3-year time period is incorporated into the simulations. Because enrollment varies by histology, a numbering scheme (1 through 9) is used in simulations and corresponding results to index the nine histologies. Histology indexing, projected enrollments and the historical control and clinically meaningful response rates used in simulations are shown in Table 32.

Table 32 Projected Enrollment and Indexing by Histology

Histology	Histology Index	Projected enrollment in a 3-year time period				Historical Control/ Clinically Meaningful RRs
		Case 1	Case 2	Case 3	Case 4	
ATC	3	10	20	25	25	15% / 60%
BTC	2	10	16	16	10	10% / 50%
GIST	7	5	5	5	2	10% / 50%
HCL	1	15	17	20	20	10% / 50%
WHO Grade 1 or 2 Glioma	6	5	8	8	5	10% / 50%
WHO Grade 3 or 4 Glioma (high-grade)	5	5	16	16	8	10% / 50%
MM	8	5	17	20	20	15% / 60%
NSGCT/NGGCT	9	5	5	5	2	25% / 75%
ASI	4	8	5	5	2	10% / 50%

Abbreviations: ASI, adenocarcinoma of small intestine; ATC, anaplastic thyroid cancer; BTC, biliary tract cancer; GIST, gastrointestinal stromal tumor; HCL, hairy cell leukemia; MM, multiple myeloma; NGGCT, non-geminomatous germ cell tumor; NSGCT, non-seminomatous germ cell tumor; RR, response rate; WHO, World Health Organization

18.4.1.3 Overall Response Rate Scenarios

Due to the ‘borrowing’ nature of the model, the design is evaluated across a variety of scenarios for the distribution of true ORRs across the histologic cohorts. These are shown in Table 33.

The “Alternative ” scenario assumes all histologies are responsive at the defined clinically meaningful RR levels. The “Null” case assumes all histologies are non- responsive at the historical control RRs. The “All Moderate” scenario assumes all histologies show moderate RR levels inbetween the historical control and clinically meaningful RR levels. The “Mixed (0.1-0.5)” assumes a range of response rates across the nine histologies. The other three scenarios consider various situations where histologies are either responsive (at the clinically meaningful RR level) or not (at the historical control RRlevel).

Table 33 Simulation Scenarios: Various Assumptions for the True ORR for each Histology

Scenario	Histology								
	1	2	3	4	5	6	7	8	9
Alternative	50%	50%	60%	50%	50%	50%	50%	60%	75%
Null	10%	10%	15%	10%	10%	10%	10%	15%	25%
All Moderate	30%	30%	40%	30%	30%	30%	30%	40%	50%
Mixed	50%	10%	30%	40%	10%	20%	30%	45%	75%
5 Greats: Mixed	50%	10%	60%	10%	50%	10%	10%	60%	75%
5 Greats: Low	10%	10%	15%	10%	50%	50%	50%	60%	75%
2 Greats	10%	50%	15%	10%	50%	10%	10%	15%	25%

Abbreviations: ORR = overall response rate

NOTE: Color indicates responsiveness of the true ORR for each histology. Red indicates non-responsive and is equal to the assumed historical control rate, green indicates responsive and is equal to the clinically meaningful ORR, and blue indicates moderate responsiveness and is in-between the historical control rate and clinically meaningful ORR.

18.4.1.4 Output from Simulated Trials

For each scenario specified in Table 33, overall descriptive measures are reported for each histology and sample sizes specified in Case 1 and include:

- Proportion of studies that declare the histology efficacious at the final analysis
- Proportion of studies that halt enrollment to each histology early for futility
- Proportion of studies that halt enrollment to each histology early for efficacy
- For each possible combination of sample size and observed number of responses, the proportion and number of times the model declares such data efficacious at the final analysis (by scenario, combining across histologic cohorts)

In-depth information is also provided for a small number of simulated example trials. These illustrate the flow and outcome of possible trials based on the pre-specified statistical decision rules.

18.4.2 Operating Characteristics

18.4.2.1 Power and Type I Error Rate

Power and type I error rate are examined across scenarios for the distribution of assumed true ORRs for each histology. The power is the probability of declaring efficacy within a histology when the true underlying ORR is greater than its corresponding historical control. Type 1 error rate is the probability of declaring efficacy within a histology when the true underlying response rate is equal to the historical control. Figure 1 illustrates the proportion of trials that declare each histology efficacious at the final analysis across simulation scenarios. Supporting tables for the simulation results are provided in Appendix 7.

The power and type I error rate are dependent upon the sample sizes and response rates across the distribution of histologies. When the treatment effects are the same across all histologies (either all responsive or all non-responsive), estimation efficiencies, due to borrowing, result in very good operating characteristics, even in histologies with very low sample sizes. Under sample size Case 1, the type I error rate is controlled to ≤0.04 across all histologies (All Null scenario). The design maintains power 93% to 98% for the higher enrolling cohorts, and

84% to 90% for the smallest enrolling 5 cohorts (All Great scenario). Also, the design exhibits reasonable power to detect a moderate level of activity (All moderate scenarios).

When the treatment effects vary across histologies, the amount of appropriate borrowing also varies, impacting the operating characteristics. The ‘2 Greats: Mixed’ investigates design performance where most histologies are not responsive. In this situation, the type I error rate is controlled to ≤0.06 and the power is 82% for the higher enrolling cohort and 55% for the low enrolling cohort. The ‘5 Greats: Mixed’, ‘5 Greats: Low’ and ‘Mixed’ scenarios investigate the design performance in situations where roughly half the histologies are responsive. The type I error rate ranges from 0.09 to 0.14 while the power ranges from 93% to 96% for the higher enrolling cohorts and ranges from 67% to 84% for the lower enrolling cohorts. Generally, improvement in the overall distribution of treatment effects tends to coincide with slight increases in type I error rate and moderate increases inpower.

Figure 1 Proportion of Studies that Declare each Histology Efficacious at the Final Analysis under Sample Size Case 1

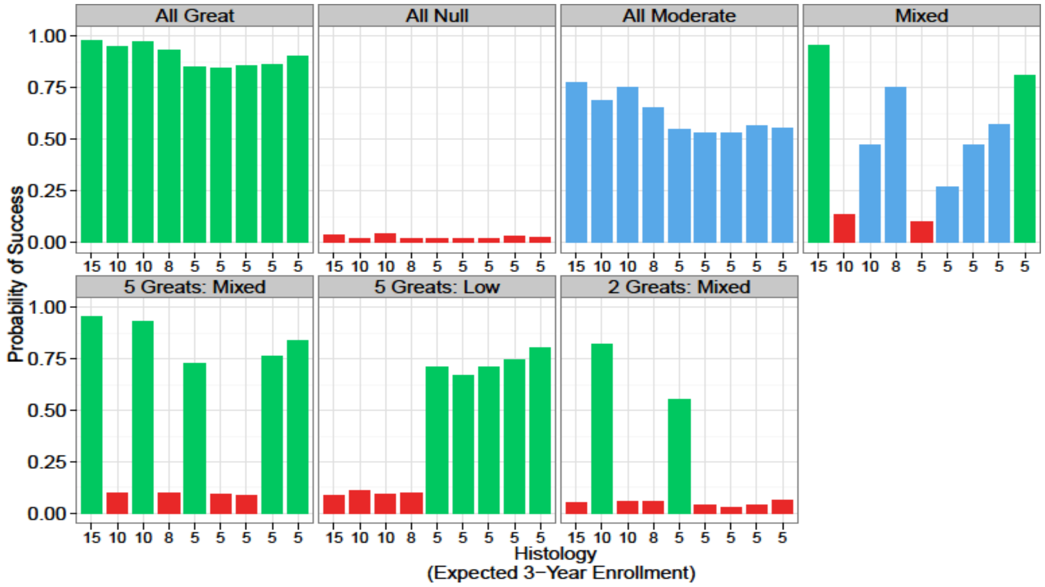

18.4.2.2 Probability that the Model Declares Success Across Different Sample Sizes and Numbers of Observed Responses

No pre-determined number of responses or response rate will necessarily dictate model success or model failure. In addition to a particular histology response data, their corresponding historical control response rate and the overall distribution of treatment effects across the histologies impacts the model decision in that particular histology. To further understand the likelihood of model success, for each possible combination ofsample size (≤15) and number of observed responses, the proportion of times the model declares such data efficacious at the final analysis is assessed by scenario but combining across histologic cohorts with the same historical control response rate. Hence, the likelihood of declaring success is explored for histologies with historical control response rates of 0.10 (histologies 1, 2, 4, 5, 6, and 7), 0.15 (histologies 3 and 8), and 0.25 (histology 9). Actual histologies, their simulation indexing number, and the appropriate historical control response rates are in Table 29 in Section 18.4.1.3.

Figure 1 illustrate the proportion of trials that declare success across all possible combinations of sample sizes and number of observed responses and for each historical control response rate. Figure 2 illustrates a subset of this data, more clearly describing the probability of declaring success when 1, 2, 3, or 4 responses are observed.

Supporting tables for these simulation results are provided in Appendix 7. Some variability is expected in these estimates due to low sample sizes in some of these observed data combinations.

Should statistical success be met while only a moderate response rate observed, it will be critical that the entirety of the data, including secondary efficacy endpoints, support a meaningful high level of activity to demonstrate evidence of a very large treatment effect. Based on the decision criteria, data from at least two subjects must be available to declare success. Hence, if data is only available for 1 subject, the success criteria will not be met, regardless of that 1 subject's response status. When the observed ORR is ≤10%, success is never declared for any histology across all seven scenarios. Hence, if zero responses are observed, a histology will never meet the success criteria. And, at least two responses are needed if response data is available for more than 10 subjects within a particular histology.

When one response is observed within a histology, there is positive probability of meeting success only in mixed situations when the sample size is small (roughly ≤5). When 2, 3 or 4 responses are observed, there is increasing probability of meeting success, but this is largely dependent upon the overall distribution of treatment effects as well as the final sample size. Also, the probability of declaring success for a particular dataset is higher for histologies with lower historical control response rates. Five responses are almost always sufficient to meet statistical success regardless of the sample size, scenario, and historical control response rate. Further details for all situations can be visually explored in Figure 3.

18.4.2.3 Probability that the Model Declares Success Across Different Sample Sizes

To evaluate the model performance for various sample sizes, simulations are also performed under the following four scenarios for sample sizes specified in Case 2, 3, and 4 with the maximum sample size for a given histology can be as high as 25.

Table 34 Simulation Scenarios for Enrollment Sensitivity: Various Assumptions for the True ORR for Each Histology

Scenario	Histology								
	1	2	3	4	5	6	7	8	9
Mixed1	20%	30%	60%	30%	40%	40%	10%	15%	75%
5 Greats: Mixed1	10%	50%	60%	50%	10%	50%	10%	15%	75%
4 Greats: Low	10%	10%	15%	50%	10%	50%	50%	15%	75%
2 Greats: Mixed1	10%	10%	60%	10%	10%	10%	50%	15%	25%

When the sample size for some histology increases from 15 to 25, the largest type I error increases from 0.14 to 0.18. Under sample size Case 2, the type I error rate is ranges from 0.04-0.17; the design maintains power 94% to 98% for the higher enrolling cohorts (>10 subjects enrolled per cohort), and 61% to 87% for the smallest enrolling three cohorts (5 subjects enrolled per cohort). Under sample size Case 3, the type I error rate is ranged from 0.04 to 0.18; the power is ranged from 92% to 98% for high enrolling cohorts (>10 subjects enrolled per cohort) and ranged from 62% to 86% for low enrolling cohorts (5-8 subjects

enrolled per cohort). Under sample size case 4, the type I error ranges from 0.02 to 0.16; the power ranges from 93% to 96% for high enrolling cohorts (>10 subjects enrolled per cohort), 59% to 71% for low enrolling cohorts (5-8 subjects enrolled per cohort) and 26% to 44% for ultra low enrolling cohorts (2 subjects enrolled per cohort). Figure 4 illustrates the proportion of trials that declare success across all possible combinations of sample sizes and number of observed responses and for each historical control response rate. Supporting tables for the simulation results are provided in Appendix 7.

Also, the design exhibits reasonable power to detect a moderate level of activity. The type I error rate maintains well under different sample size scenarios and reasonable power are achived for cohorts which enroll no less than 5 subjects per cohort. The powers for cohorts that have super low enrollment (n=2 or less) can be lower than 50%. Overall the design performance is robust to overenrolling up to 25 subjects for a given histology.

Figure 2 For Each Combination of Sample Size and Observed Number of Responses, the Probability of Model Success at the Final Analysis (by scenario, combined across histologies with the same historical control RR)

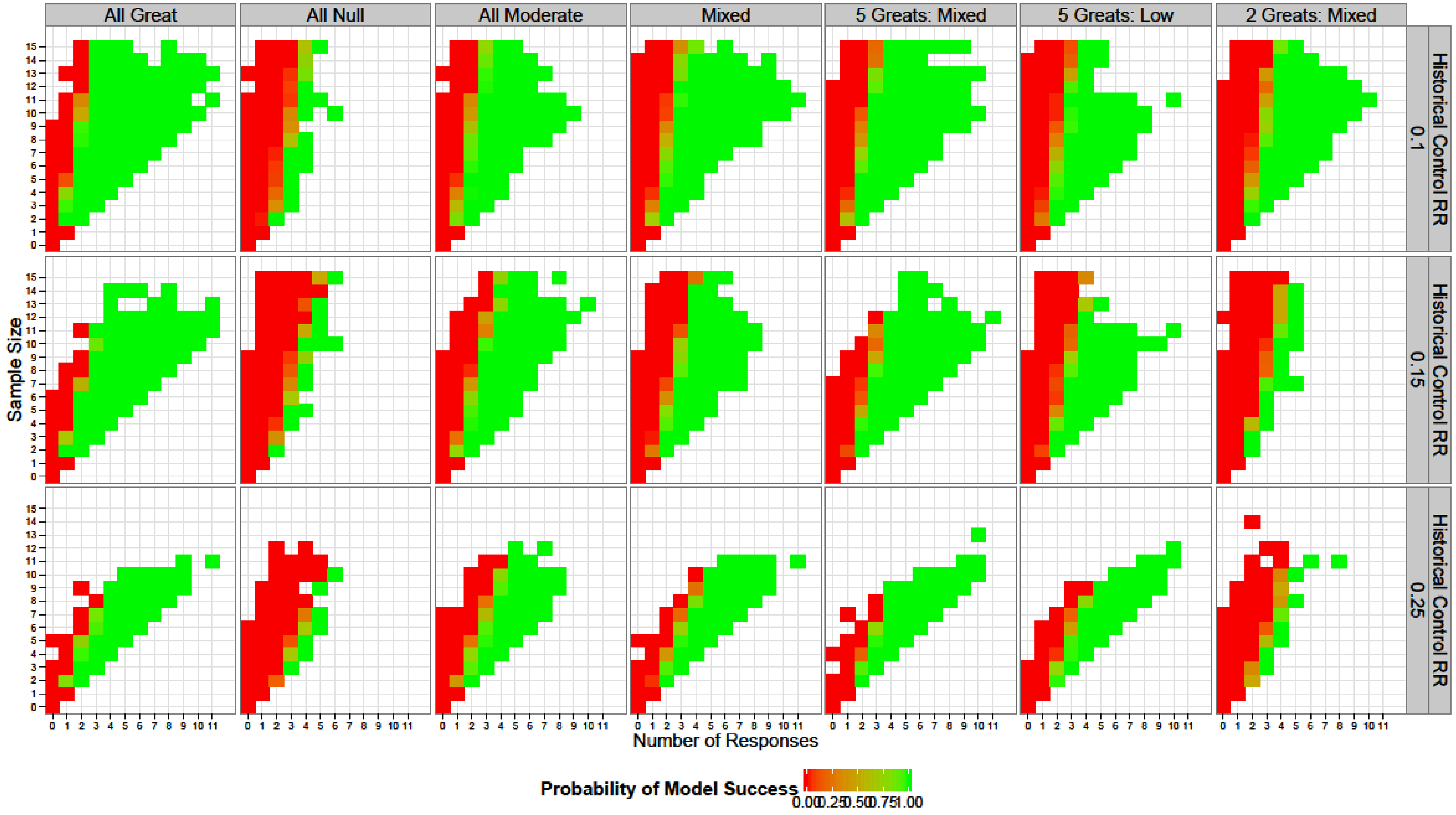

Figure 3 For All Sample Sizes and When the Number of Observed Responses is 1, 2, 3, or 4, the Probability of Model Success at the Final Analysis (by scenario, combinated across histologies with the same historical control RR)

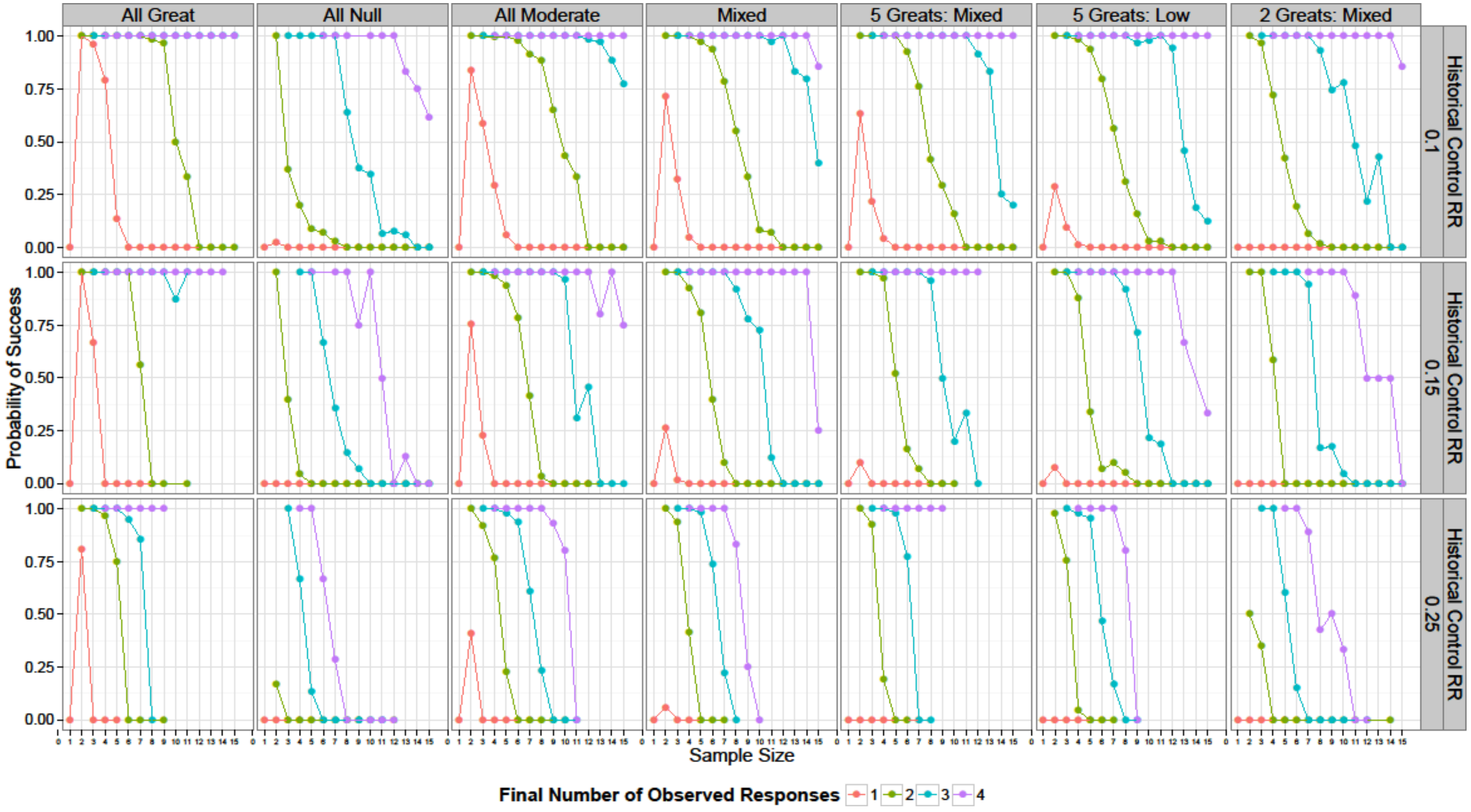

Figure 4 Proportion of Studies that Declare Each Histology Efficacious at the Final Analysis under Sample Size Case 2, 3 and 4

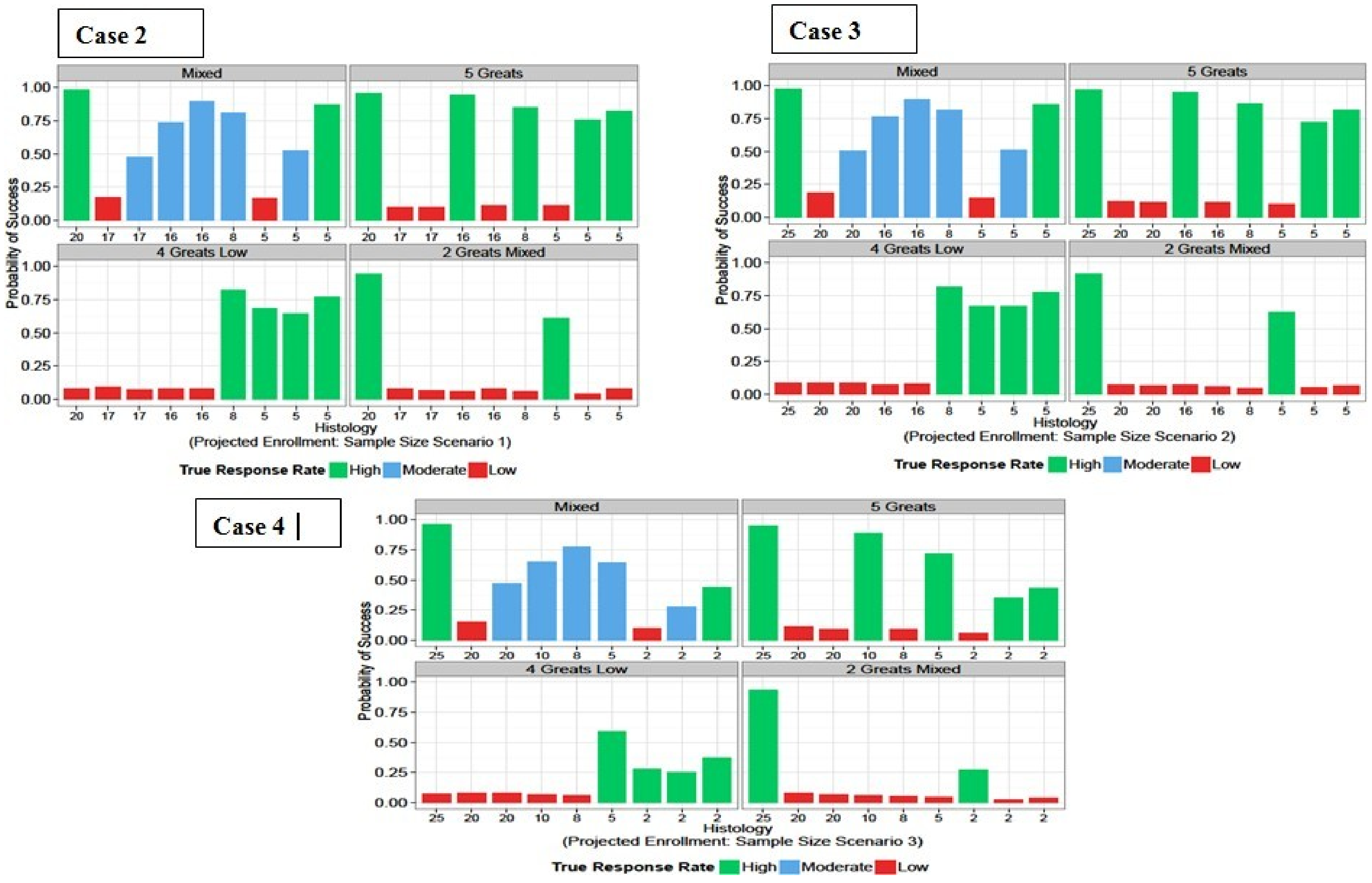

Stopping Early Figure 5 and Figure 6 illustrate the proportion of trials that halt enrollment early for futility and efficacy across simulation scenarios. Supporting tables for the simulation results are provided in Appendix 7.

Since the study requires at least five subjects in a particular histology prior to stopping early for futility and 10 subjects in a histology prior to stopping early for efficacy, the ability for histologies to stop early is largely dependent upon the projected maximum sample size per histology. The overall distribution of treatment effects across histologies also impacts the likelihood of halting enrollment early.

When the expected 3-year enrollment is greater than 5, non-responsive histologies stop early for futility between 32% and 66% of the time. When the expected 3-year enrollment is five subjects, non-responsive histologies stop early for futility between 8% and 35% of the time. When the expected 3-year enrollment is at least 10, responsive histologies stop early for futility between 41% and 90% of the time. When the expected 3-year enrollment is less than 10, the probability of stopping early for success is very low. Across simulation scenarios, non-responsive histologies generally do not stop early for efficacy while responsive histologies generally do not stop early for futility.

Figure 5 Proportion of Trials that Halt Enrollment Early for Futility for Each Histology

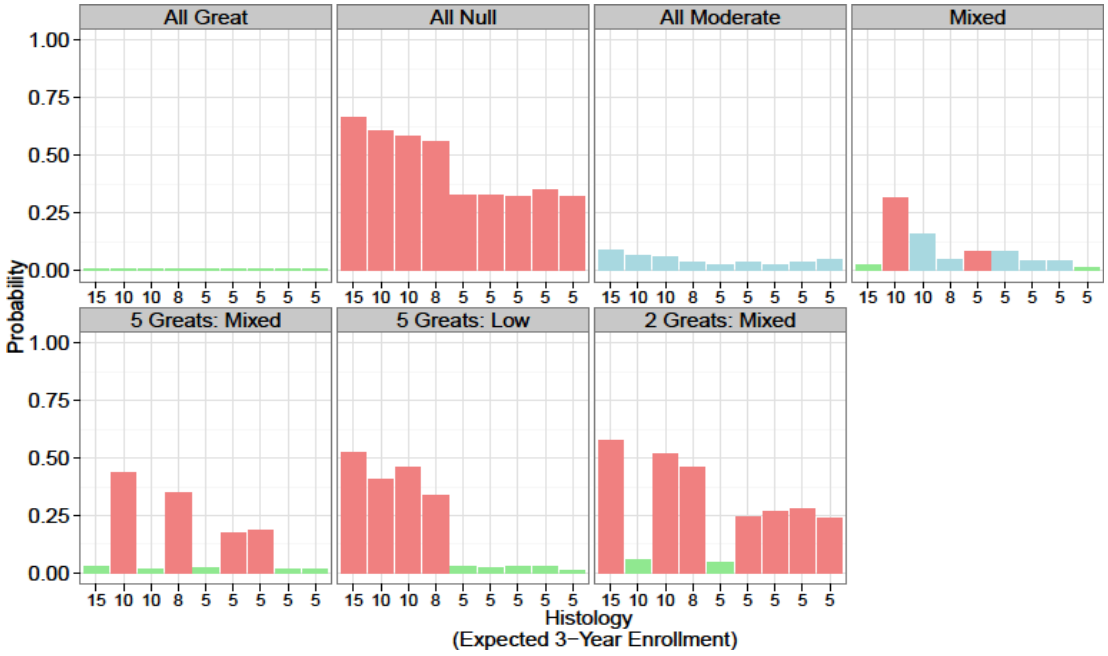

Figure 6 Proportion of Trials that Halt Enrollment Early for Success for each Histology

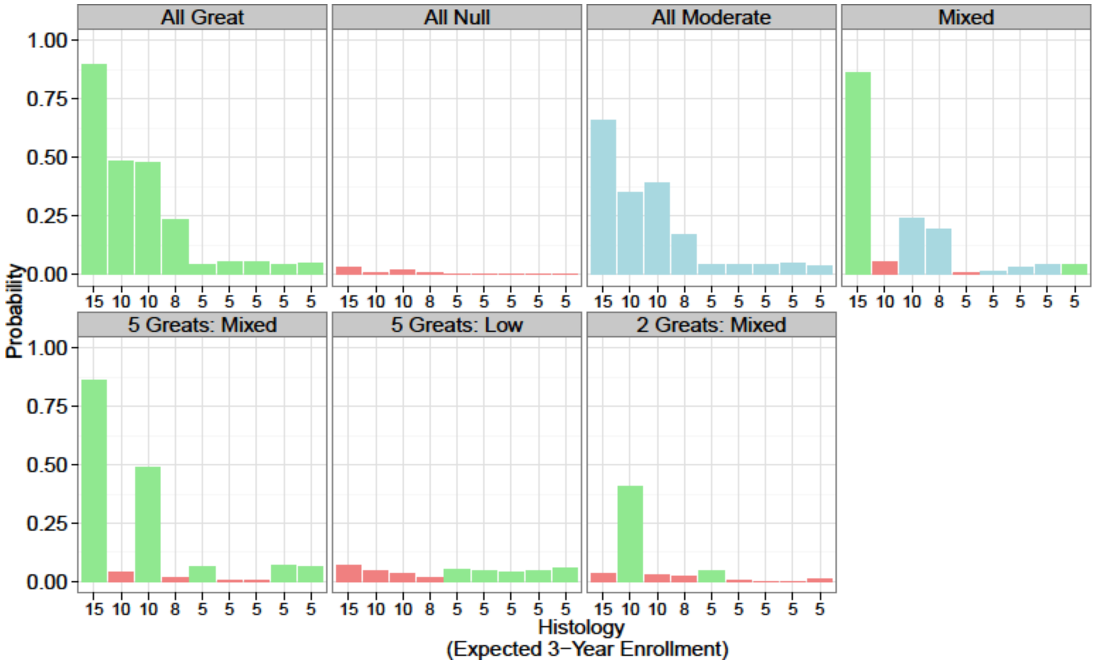

18.4.3 Example Trials

Select interim and final analysis results are presented for six simulated trials in Appendix 8. The simulated trials were chosen to represent a variety of potential data outcomes that may be observed. These illustrate the flow and outcome of possible trials based on the pre-specified statistical decision rules.

Because the overall distribution of treatment effects across the histologies impacts the model decision, no pre-determined number of responses or response rate will necessarily dictate model success or model failure at interim or final analyses. The above simulation results describe the likelihood of different model outcomes based on different scenarios for the assumed distribution of treatment effects. The example trials provide additional insight into how the model will perform and the level of evidence required to meet statistical success for possible trial outcomes.

19 STUDY CONDUCT CONSIDERATIONS

19.1 Posting of Information on Publicly Available Clinical Trial Registers

Study information from this protocol will be posted on publicly available clinical trial registers before enrollment of subjects begins.

19.2 Regulatory and Ethical Considerations, Including the Informed Consent Process

Prior to initiation of a study site, the sponsor will obtain favourable opinion/approval from the appropriate regulatory agency to conduct the study in accordance with International

Conference on Harmonization (ICH) Good Clinical Practice (GCP) and applicable country-specific regulatory requirements.

The study will be conducted in accordance with all applicable regulatory requirements.

The study will be conducted in accordance with ICH GCP, all applicable subject privacy requirements, and the ethical principles that are outlined in the Declaration of Helsinki 2008, including, but not limited to:

- IRB /IEC review and favorable opinion/approval of study protocol and any subsequent amendments
- Subject informed consent
- Investigator reporting requirements

The sponsor will provide full details of the above procedures, either verbally, in writing, or both.

Written informed consent must be obtained from each subject prior to participation in the study.

In approving the clinical protocol the IEC/IRB and, where required, the applicable regulatory agency are also approving the optional assessments (e.g., PGx assessments described in Appendix 4), unless otherwise indicated. Where permitted by regulatory authorities, approval of the optional assessments can occur after approval is obtained for the rest of the study. If so, then the written approval will clearly indicate approval of the optional assessments is being deferred and the study, except for the optional assessments, can be initiated. When the optional assessments are not approved, then the approval for the rest of the study will clearly indicate this and therefore, the optional assessments will not be conducted.

19.3 Data Monitoring Committee

A Data Monitoring Committee (DMC) will be utilized in this study and will be comprised of individuals who are not members of the clinical study team. At least one independent hematologist and one independent oncologist (external to the sponsor) will serve on the committee. The objective of the DMC will be to ensure objective medical and/or statistical review of safety and/or efficacy issues in order to protect the ethical and safety interests of subjects and to protect the scientific validity of the study. The schedule of any planned interim analyses and the analysis plan for DMC review will be described in the committee's charter.

19.4 Quality Control (Study Monitoring)

In accordance with applicable regulations, GCP, and Novartis procedures, Novartis personnel (or designated Clinical Research Organization [CRO]) will contact the site prior to the start of the study to review with the site staff the protocol, study requirements, and their responsibilities to satisfy regulatory, ethical, and the sponsor's requirements. When reviewing data collection procedures, the discussion will include identification, agreement and documentation of data items for which the eCRF will serve as the source document.

The investigator and the head of the medical institution (where applicable) agrees to allow the monitor direct access to all relevant documents and to allocate their time and the time to their staff to monitor to discuss findings and any issues.

Monitoring visits will be conducted in a manner to ensure that the:

- Data are authentic, accurate, and complete.
- Safety and rights of subjects are being protected.
- Study is conducted in accordance with the currently approved protocol and any other study agreements, ICH GCP, and all applicable regulatory requirements.

19.5 Quality Assurance

To ensure compliance with GCP and all applicable regulatory requirements, the sponsor may conduct a quality assurance assessment and/or audit of the site records, and the regulatory agencies may conduct a regulatory inspection at any time during or after completion of the study. In the event of an assessment, audit or inspection, the investigator (and institution) must agree to grant the advisor(s), auditor(s) and inspector(s) direct access to all relevant documents and to allocate their time and the time of their staff to discuss the conduct of the study, any findings/relevant issues and to implement any corrective and/or preventative actions to address any findings/issues identified

19.6 Study and Site Closure

The end of the study is defined as the date of the last subject, last visit.

Upon completion or termination of the study, Novartis personnel (or designated CRO) will conduct site closure activities with the investigator or site staff (as appropriate), in accordance with applicable regulations, ICH GCP, and Novartis Standard Operating Procedures.

The sponsor reserves the right to temporarily suspend or terminate the study at any time for reasons including (but not limited to) safety issues, ethical issues, or severe noncompliance. If the sponsor determines that such action is required, the sponsor will discuss the reasons for taking such action with the investigator or head of the medical institution (where applicable). When feasible, the sponsor will provide advance notice to the investigator or head of the medical institution of the impending action.

If a study is suspended or terminated for safety reasons, the sponsor will promptly inform all investigators, heads of the medical institutions (where applicable),and/or institutions conducting the study. The sponsor will also promptly inform the relevant regulatory authorities of the suspension/termination along with the reasons for such action. Where required by applicable regulations, the investigator or head of the medical institution must inform the IRB/IEC promptly and provide the reason(s) for the suspension/termination.

Study sites and/or the study may be closed by the sponsor if the site/study fails to recruit within a pre-defined timeframe, as defined within the SPM.

19.7 Records Retention

Following closure of the study, the investigator or head of the medical institution (where applicable) must maintain all site study records (except for those required by local regulations to be maintained elsewhere) in a safe and secure location. The records must be easily accessible when needed (e.g., for a sponsor audit or regulatory inspection) and must be available for review in conjunction with assessment of the facility, supporting systems, and relevant site staff.

Where permitted by local laws/regulations or institutional policy, some or all of the records may be maintained in a format other than hard copy (e.g., microfiche, scanned, electronic); however, caution must be exercised before such action is taken. The investigator must ensure that all reproductions are legible and are a true and accurate copy of the original. In addition, they must meet accessibility and retrieval standards, including regeneration of a hard copy, if required. The investigator must also ensure that an acceptable back-up of the reproductions exists and that there is an acceptable quality control procedure in place for creating the reproductions.

Essential documents (written and electronic) should be retained for a period of not less than fifteen (15) years from the completion of the Clinical Trial unless the Sponsor provides written permission to dispose of them or, requires their retention for an additional period of time because of applicable laws, regulations and/or guidelines.

The investigator must notify the sponsor of any changes in the archival arrangements, including, but not limited to archival of records at an off-site facility or transfer of ownership of the records in the event that the investigator is no longer associated with the site.

19.8 Provision of Study Results to Investigators, Posting of Information on Publicly Available Clinical Trials Registers and Publication

Where required by applicable regulatory requirements, an investigator signatory will be identified for the approval of the clinical study report. The investigator will be provided reasonable access to statistical tables, figures, and relevant reports and will have the opportunity to review the complete study results at a sponsor site or other mutually- agreeable location.

The sponsor will also provide the investigator with the full summary of the study results. The investigator is encouraged to share the summary results with the study subjects, as appropriate.

Novartis aims to post a results summary to the Novartis Clinical Trial Results website (www.novartisclinicaltrials.com) and other publicly available registers no later than twelve (12) months after the last subject's last visit (LSLV). In addition, upon study completion and finalization of study report, Novartis aims to submit results of the study for publication.

When publication is not feasible, please refer to the Novartis Clinical Trial Results website (novartisclinicaltrials.com) for a summary of the trial results.

20 REFERENCES

[redacted]

Agaram NP, Wong GC, Guo T, et al. Novel V600E BRAF mutations in imatinib-naïve and imatinib-resistant gastrointestinal stromal tumors. *Genes Chromosomes Cancer.* 2008;47(10):853-859.

Ain KB, Egorin MJ, DeSimone PA. Treatment of anaplastic thyroid carcinoma with paclitaxel: Phase 2 trial using 96-hour infusion. Collaborative Anaplastic Thyroid Cancer Health Intervention Trials (CATCHIT) Group. *Thyroid*. 2000;10(7):587-594.

Alcala AM, Flaherty K. BRAF inhibitors for the treatment of metastatic melanoma: Trials and mechanisms of resistance. *Clin Cancer Res*. 2012;18:33-39.

American Cancer Society (ACS): Cancer Facts and Figures 2012. Atlanta, Ga: American Cancer Society, 2012. Last accessed July 31, 2012.

Anderson KC, Carrasco RD. Pathogenesis of myeloma. *Annu.Rev.Pathol.Mech.Dis.* 2011;6:249-274.

Balagula Y, Garbe C, Myskoski PL, et al. Clinical presentation and management of dermatological toxicities of epidermal growth factor receptor inhibitors. *Int J ofDerm*. 2011;50:129-146.

Berry SM, Broglio MS, Groshen S, Berry DA. Bayesian Hierarhical Modeling ofPatient Subpopulations: Efficient Design of Phase II Oncology Clinical Trials. *Clinical Trials*. 2013;10:720-734.

Beyer J, Kramar A, Mandanas R, et al.: High-dose chemotherapy as salvage treatment in germ cell tumors: a multivariate analysis of prognostic variables. *J Clin Oncol.* 1996;14(10):2638-45.

Bilimoria KY, Bentrem DJ, Wayne JD, et al. Small bowel cancer in the United States: changes in epidemiology, treatment, and survival over the last 20 years. *Ann Surg*. 2009;249:63–71.

Bokemeyer C, Oechsle K, Honecker F, et al. Combination chemotherapy with gemcitabine, oxaliplatin, and paclitaxel in patients with cisplatin-refractory or multiple relapsed germ-cell tumors: a study of the German Testicular Cancer Study Group. *Annals of Oncology.* 2008;19:448-453.

Borger DR, Tanabe KK, Fan KC, et al. Frequent mutation of isocitrate dehydrogenase (IDH)1 and IDH2 in cholangiocarcinoma identified through broad-based tumor genotyping. *Oncologist*. 2012;17(1):72-79.

Caram, MV, Schuetze SM. Advanced or Metastatic Gastrointestinal Stromal Tumors: Systemic Treatment Options. *J Surg Onc*. 2011;104:888-895.

Caronia LM, Phay JE, Shah MH. Role of BRAF in Thyroid Oncogenesis. *Clin Cancer Res.* 2011;17:7511-7517.

Catovsky D, Golomb HM, Golde DW. Consensus Resolution: Proposed Criteria for Evaluation of Response to Treatment in Hairy Cell Leukemia. *Leukemia*. 1987;4(1):405-406.

Chapman MA, Lawrence MS, Keats JJ, et al. Initial genome sequencing and analysis of multiple myeloma. *Nature.* 2011b;471(7339):467-472.

Chapman P, Hauschild A, Robert C, et al. Improved survival with vemurafenib in melanoma with BRAF V600E mutation. *N Eng J Med*, 2011a;364:2507-2516.

Chrobak L, Podzimek K, Kerekes Z, Spacek J, Voglova. Long-term results in hairy cell leukemia treated by splenectomy. *Neoplasma*. 1993;40(2):133-136. clinical trials and mechanisms of resistance. *Clin Cancer Res.* 2012;18(1):33-39.

Dasanu CA, Alexandrescu DT. Risk of additional cancers in untreated and treated hairy cell leukemia patients. *Expert Opin Pharmacother*. 2010;11(1):41-50.

Davies H, Bignell GR, Cox C, Stephens P, Edkins S, et al. Mutations of the BRAF gene in human cancer. *Nature.* 2002;417:949-954.

DeCrevoisier R, Baudin E, Bachelot A, et al. Combined treatment of anaplastic thyroid carcinoma with surgery, chemotherapy, and hyperfractionated accelerated external radiotherapy. *Int J Radiat Oncol Biol Phys*. 2004;60(4):1137-1143.

Demetri GD, van Oosterom AT, Garrett CR, et al. Efficacy and safety of sunitinib in patients with advanced gastrointestinal stromal tumor after failure of imatinib: a randomized controlled trial. *Lancet.* 2006;368:1329-1338.

DeWit R, Stoter G, Sleijfer DT, et al. Four cycles of BEP vs four cycles of VIP in patients with intermediate-prognosis metastatic testicular non-seminoma: a randomized study of the EORTC Genitourinary Tract Cancer Cooperative Group. *Br J Cancer.* 1998;78:828-832.

Dias-Santagata D, Lam Q, Vernovsky K, et al. BRAF V600E Mutations are Common in Pleomorphic Xanthoastrocytoma: Diagnostic and Therapeutic Implications. PLoS ONE. 2011;6(3):1-9.

Dietrich S,Glimm H, Andrulis M, et al. BRAF Inhibition in Refractory Hairy Cell Leukemia. *N Engl J Med*. 2012;366(21):2038-2039.

Durie BGM, Harousseau J-L, Miguel JS, et. al. International uniform response criteria for multiple myeloma. *Leukemia.* 2006:20:1467-73.

Einhorn LH, Brames MJ, Juliar B, Williams SD. Phase II Study of Paclitaxel plus Gemcitabine Salvage Chemotherapy for Germ Cell Tumors after Progression Following High-Dose Chemotherapy with Tandem Transplant. *J Clin Oncol.* 2007;25(5):513-516.

Else M, Dearden CE, Matutes E, et al. Long-term follow-up of 233 patients with hairy cell leukemia, treated initially with pentostatin or cladribine, at a median of 16 years from diagnosis. *Br J Hematol*. 2009;145(6):733-740.

Else M, Osuji N, Forconi F, et al. The role of rituximab in combination with pentostatin or cladribine for the treatment of recurrent/refractory hairy cell leukemia. *Cancer*. 2007;110(10):2240-2247.

Escobar MD, West M. Bayesian Density Estimation and Inference Using Mixtures. *J of American Statistical Association*. 1995;90(430):577-588.

Estey, EH, Kurzrock R, Kantarijan HM, et al. Treatment of hairy cell leukemia with 2-chlorodeoxyadenosine (2-CdA). *Blood.* 1992;79(4):882-887.

Ferlay J, Shin HR, Bray F, et al.: GLOBOCAN 2008: Cancer Incidence and Mortality Worldwide in 2008. Lyon, France: IARC Cancer Base No. 10. Last accessed December 1, 2011.

Flaherty KT, Robert C, Hersey P, et al. Improved survival with MEK inhibition in BRAF-Mutated Melanoma. *N Engl J Med.* 2012;367(2):107-114.

Frassoldati A, Lamparelli T, Federico M, et al. Hairy cell leukemia: a clinical review based on 725 cases of the Italian Cooperative Group (ICGHCL). *Leuk Lymphoma*. 1994;13(3-4):307-316.

Frohlich MW, Small EJ. Stage II non-seminomatous testis cancer: the roles of primary and adjuvant chemotherapy. *Urol Clin North Am.* 1998;24:451-459.

Gidron A. Tallman MS. 2-Cda in the treatment of hairy cell leukemia: a review of long-term follow-up. *Leuk Lymphoma*. 2006;47(11):2301-2307.

Gilmartin AG, Bleam MR. Groy R, et al. GSK1120212 (JTP-74057) is an inhibitor of MEK activity and activation with favourable pharmacokinetic properties for sustained in vivo pathway inhibition. *Clin Cancer Res*, 2011;17:989-1000.

GlaxoSmithKline Document Number CM2010/00010/05. Dabrafenib (GSK2118436) Monotherapy and Dabrafenib+Trametinib (GSK1120212) Combination Therapy Investigator's Brochure. Version 07. Report dated 09-Oct-2015.

GlaxoSmithKline Document Number HM2009/00151/05. GSK1120212 (trametinib) Investigator's Brochure. Version 06. Report dated 28-Jan-2015.

Goldman JM, Goren EN, Cohen BL, Webber M, Brennan F, Robbins J. Anaplastic thyroid carcinoma: long term survival after radical surgery. *J of Surg Onc*. 1980;14(4):389-394.

Golomb HM, Vardiman JW. Response to splenectomy in 65 patients with hairy cell leukemia: an evaluation of spleen weight and bone marrow involvement. *Blood.* 1983;61(2):349-352.

Goodman GR, Burian C, Koziol Ja, et al. Extended follow-up of patients with hairy cell leukemia after treatment with cladribine. *J Clin Oncol*. 2003;21(5):891-896.

Goutsouliak V, Hay JH. Anaplastic thyroid cancer in British Columbia 1985 – 1999: a population-based study. *Clin Oncol (R Coll Radiol)*. 2005;17:75-78.

Greger JG, Eastman SD, Zhang V, et al. Combinations of BRAF, MEK, and PI3K/mTOR inhibitors overcome acquired resistance to the BRAF inhibitor GSK2118436 dabrafenib, mediated by NRAS or MEK mutations. *Mol Cancer Ther* 2012;11(4):909-920.

Grever M, Kepecky K, Foucar MK, et al. Randomizzed comparison of pentostatin versus interferon alfa-2a in previously untreated patients with hairy cell leukemia: an intergroup study. *J Clin Oncol*. 1995;13(4):974-982.

Grever MR, Lazanski G. Modern strategies for hairy cell leukemia. *J Clin Oncol*. 2011;29(5):483-590.

Haberman TM, Rai K. Historical treatments of hairy cell leukemia, splenectomy and interferon: past and current uses. *Leukemia & Lymphoma*. 2011(6);52(S2):18-20.

Haberman TM. Splenectomy, interferon, and treatments of historical interest in hairy cell leukemia. *Hematol Oncol Clin North Am*. 2006;20(5):1075-1086.

Hadziahmetovic M, Shirai K Chakravarti A. Recent advancements in multimodality treatment of gliomas. *Future Oncol.* 2011;7(10):1169-1183.

Haigh PI, Ituarte PHG, Wu HS, et al. Completely resected anaplastic thyroid carcinoma combined with adjuvant chemotherapy and irradiation is associated with prolonged survival. *Cancer.* 2001;91:2335-2342.

Hauschild, A, Jacques JG, Demidov LV, et al. Phase III, randomized, open-label, multicenter trial (BREAK-3) comparing the BRAF kinase inhibitor dabrafenib (GSK2118436) with dacarbazine (DTIC) in patients with BRAF V600E-mutated melanoma. *J Clin Oncol.* 2012;30:(suppl;abstr8500).

Heinrich MC, Corless CL, Demetri GD, et al. Kinase mutations and imatinib response in patients with metastatic gastrointestinal stromal tumors. *J Clin Oncol*. 2003;21(23):4342-4349.

Heinrich MC, Joensuu H, Demetri GD, et al. Phase II, open-label study evaluating the activity of imatinib in treating life-threatening malignancies known to be associated with imatinib-sensitive tyrosine kinases. *Clin Cancer Res.* 2008;14(9):2717-2725.

Hezel AF, Deshpande V, Zhu AX. Genetics of Biliary Tract Cancers and Emerging Targeted Therapies. *J Clin Oncol.* 2010;28(10):3531-3540.

Hinton S, Catalano PJ, Einhorn LH, et al. Cisplatin, etoposide and either bleomycin or ifosfamide in the treatment of disseminated germ cell tumors: final analysis of an intergroup trial. *Cancer* 2003;97(8):1869-1875.

Hisada M, Chen BE, Jaffe ES, Travis LB. Second cancer incidence and cause-specific mortality among 3104 patients with hairy cell leukemia: a population-based study. *J Natl Cancer Inst*. 2007;99(3):215-222.

Honecker F, Wermann H, Mayer F, et al. Microsatellite Instability, Mismatch Repair Deficiency and BRAF Mutation in Treatment-Resistant Germ Cell Tumors. *J Clin Oncol.* 2009;27:2129-2136.

Hostein I, Faur N, Primois C, et al. BRAF Mutation Status in Gastrointestinal Stromal Tumors. *Am J Clin Pathol.* 2010;133:141-148.

Hunt, CM. Mitochondrial and immunoallergic injury increase risk of positive drug rechallenge after drug-induced liver injury: A systematic review. Hepatol. 2010;52:2216-2222.

International Germ Cell Cancer Collaborative Group (IGCCCG): International Germ Cell Consensus Classification: A Prognostic Factor-Based Staging System for Metastatic Germ Cell Cancers. *J Clin Oncol.* 1997;15(2):594-603.

Italiano A, Cioffi A, Coco P, et al. Patterns of care, prognosis, and survival in patients with metastatic gastrointestinal stromal tumors (GIST) refractory to first-line imatinib and second-line sunitinib. *Ann Surg Oncol.* 2011;19(5):1551-1559.

James LP, Letzig L, Simpson PM, et al. Pharmacokinetics of Acetaminophen-Adduct in Adults with Acetaminophen Overdose and Acute Liver Failure. *Drug Metab Dispos.* 2009; 37:1779-1784.

James W, Stein C. Estimation with Quadratic Loss. *Proc Fourth Berkeley Symp on Math Statist and Prob*. 1961;1:361–379.

Jansen J, Hermans J. Splenectomy in hairy cell leukemia: a retrospective multicenter analysis. *Cancer*. 1981;47:2066-2076.

Jemal A, Siegel R, Ward E, et al. Cancer Statistics 2009. CA Cancer J Clin. 2009;59:225-249.

Johannessen CM, Boehm JS, Kim SY, et al. Rationale of duak therapy COT drives resistance to RAF inhibition through MAP kinase pathway reactivation. Nature. 2010;468:968-972.

Jones RH, Vasey PA. Part II: testicular cancer – management of advanced disease.

Lancet Oncol. 2003;4:738-747.

Kim, K, Kong SY, Fulciniti M, et al. Blockade of the MEK/ERK signalling cascade by AS703026, a novel selective MEK1/2 inhibitor, induces pleiotropic anti-myeloma activity in vitro and in vivo. *Br. J. Haematol*. 2010;149:537–549.

Kojic SL, Strugnell SS, Wiseman SM. Anaplastic thyroid cancer: a comprehensive review of novel therapy. *Expert Rev. Anticancer Ther.* 2011;11(3):387-402.

Kollmannsberger C, Beyer J, Liersch R, et al. Combination chemotherapy with gemcitabine plus oxaliplatin in patients with intensively pretreated or refrctory germ cell cancer: A Study of the German Testicular Cancer Study Group. *J Clin Oncol.* 2004;22:108-114.

Kreitman RJ, Fitzgerald DJ, Pastan I. Approach to the patient after relapse of hairy cell leukemia. *Leuk Lymphoma.* 2009a;50(suppl 1):32-37.

Kreitman RJ, Tallman MS, Robak T, et al. Phase I trial of anti-CD22 recombinant immunotoxin moxetumomab pasudotox (CAT-8015 or HA22) in patients with hairy cell leukemia. *J Clin Oncol*. 2012;30:1822-1828.

Kreitman, RJ, Stetler-Stevenson, M, Margulies, I, et al. Phase II trial of recombinant immunotoxin RFB4(dsFv)-PE38 (BL22) in patients with hairy cell leukaemia. *J Clin Oncol.* 2009b;27:2983–2990.

Kumar S, Blade J, Crowley J, Goldschmidt H, Hoering A, Jagannath S. Outcome of patients with myeloma relapsing after IMiD and bortezomib therapy: a multicentre study from the IMFWG. *Haematologica.* 2010;95(suppl.2):151, abstract 0376.

Kumar SK, Rajkumar SV, Dispenzieri A, et al. Improved survival in multiple myeloma and the impact of novel therapies. *Blood*. 2008;111(5):2516-2520. Prepublished on 2007/11/03 as DOI 10.1182/blood-2007-10-116129.

LaCouture ME, Anadkat MJ, Bensadoun RJ, et al. Clinical Practice guidelines for the prevention and treatment of EGFR inhibitor-associated dermatologic toxicities. *Support Care Cancer.* 2011;19:1079-1095.

Le Gal F, Gordien E, Affolabi D, et al. Quantification of Hepatitis Delta Virus RNA in Serum by Consensus Real-Time PCR Indicates Different Patterns of Virological Response to Interferon Therapy in Chronically Infected Patients. *J Clin Microbiol*. 2005;43(5):2363–2369.

Long GV et al. Dabrafenib and trametinib versus dabrafenib and placebo for Val600 BRAF-mutant melanoma: a multicentre, double-blind, phase 3 randomised controlled trial. *Lancet.* 2015; 386(9992): 444-51

Mannuel H, Mitikiri N, Hussain A. Update on testicular germ cell tumors. *Current Opin in Oncology.* 2011;23:265-270.

Masque-Soler N, Szczepanowski M, Leuschner I, et al. Brief Report: Absence of BRAF Mutation in Pediatric and Adolescent Germ Cell Tumors Indicate Biological Difference to Adult Tumors. *Pediatr Blood Cancer*. 2012;59:732-735.

Matutes E, Wotherspoon A, Catovsky D. The variant form of hairy cell leukemia. *Best Pract Res Clin Hematol*. 2003;16(1):41-56.

Mayer F, Wermann H, Albers P, et al. Histopathological and molecular features of late relapses in non-seminomas. *BJUI.* 2010;107:936-943.

Miranda C, Nucifora M, Molinari F, et al. KRAS and BRAF mutations predict primary resistance to imatinib in gastrointestinal stromal tumors. *Clin Cancer Res.* 2012;18(6):1769-1776.

Morton LM, Wang SS, Devesa SS, Hartge P, Weisenburger DD, Linet MS. Lymphoma incidence patterns by WHO subtype in the United States, 1992-2001. *Blood*. 2006;107:265-276.

Nagaiah G, Hossain A, Mooney CJ, Parmentier J, Remick SC. Anaplastic Thyroid Cancer: A review of epidemiology, pathogenesis and treatment. *J of Oncology.* 2011; Article ID 542358. Doi:10.1155/2011/542358.

Nazeer T, Ro JY, Amato RJ, et al. Histologically pure seminoma with elevated alpha-fetoprotein: a clinicopathologic study of 10 cases. *Oncol Rep.* 1998;5:1425-1429.

NCI Common Terminology Criteria for Adverse Events, Version 4, DCTD, NCI, NIH, DHHS, 28 May 2009.

Neal RM. Markov Chain Sampling Methods for Dirichlet Process Mixture Models. *J of Computational and Graphical Statistics*. 2000;9(2):249-265.

Niault TS, Baccarini M. Targets of RAF in tumorigenesis. *Carcinogenesis*. 2010;31(7):1165-1174.

Nichols CR, Heerema NA, Palmer C, et al.: Klinefelter's syndrome associated with mediastinal germ cell neoplasms. *J Clin Oncol* 1987;5(8):1290-1294.

Nieva J, Bethel K, Saven A. Phase 2 study of rituximab in the treatment of cladribine-failed patients with hairy cell leukemia. *Blood* 2003;102:810-813.

Nucera C, Neha MA, Nagarkatti SS, et al. Targeting BRAF V600E with PLX4720 Displays Potent Antimigratory and Anti-invasive Activity in Pre-Clinical Models of Human Thyroid Cancer. *The Oncologist*. 2011;16:296-309.

Overman MJ, Hu CYH, Kopetz S, et al. A Population-Based Comparison of Adenocarcinoma of the Large and Small Intestine: Insights Into a Rare Disease. *Ann Surg Oncol*. 2012;19(5):1439-1445.

Papay JI, Clines D, Rafi R, et al. Drug-induced liver injury following positive drug rechallenge. *Regul Tox Pharm*. 2009;54:84-90.

Perry A, Aldape KD, Goerge DH, Burger PC. Small Cell Astrocytoma: An Aggressive Variant that is Clinicopathologically and Genetically Distinct from Anaplastic Oligodendroglioma. *Cancer.* 2004;101(10):2318-2326.

Pitt SC, Moley JF. Medullary, Anaplastic and Metastatic Cancers of the Thyroid. *Semin Oncol.* 2010;37:567-579.

Posner MD, Quivey JM, Akazawa PF, et al. Dose Optimization for the Treatment of Anaplastic Thyroid Carcinoma: A Comparison of Treatment Planning Techniques. *Int J Radiation Oncology Biol. Phys.* 2000;48(2):475-483.

Prahallad A, Sun C, Huang S, et al. Unresponsiveness of colon cancer to BRAF(V600E) inhibition through feedback activation of EGFR. *Nature.* 2012;483(7387):100-103.

Rajkumar SV, Harousseau JL, Durie B, et al. Consensus recommendations for the uniform reporting of clinical trials: Report of the International Myeloma Workshop Consensus Panel 1. *Blood*. 2011;117:4691-4695.

Ravandi F, Jorgensen JL, O’Brien SM, et al. Eradication of minimal residual disease in hairy cell leukemia. *Blood*. 2006;107(12):4658-4662.

Riccioni R, Galimberti S, Petrini M. Hairy Cell Leukemia. *Curr Trmt Options in Onc*. 2007;8:129-134.

Richardson PG, Sonneveld P, Schuster MW, et al. Bortezomib or High-Dose Dexamethasone for Relapsed Multiple Myeloma. *N Engl J Med*. 2005;352:2487-2498.

Robert C et al. Improved overall survival in melanoma with combined dabrafenib and trametinib. *N Engl J Med*. 2015; 372(1): 30 - 9

Rosove MH, Peddi PF, Glaspy JA. BRAF V600E Inhibition in Anaplastic Thyroid Cancer – Letter to the Editor. *N Engl J Med.* 2013;368 (7):684-685.

Sadighi Z, Slopis J. Pilocytic Astrocytoma: A Disease with Evolving Molecular Heterogeneity. *J Child Neurol*. 2013;28:625-632.

Saetta AA, Papanastasiou P, Michalopoulos NV, Gigelou F, Korkolopoulou P, Bei T, Patsouris E. Mutational analysis of BRAF in gallbladder carcinomas in association with K-ras and p53 mutations and microsatellite instability. *Virchows Arch.* 2004;445:179- 182.

Saven A, Burian C, Koziol JA, Piro LD. Long-term follow-up of patients with hairy cell leukemia after cladribine treatment. *Blood.* 1998;92(6):1918-1926.

Saven A. Treatment of hairy-cell leukemia. *N Engl J Med*. 2001;345(20):1500-1501.

Saxman SB, Nichols CR, Einhorn LH. Salvage chemotherapy in patients with extragonadal nonseminomatous germ cell tumors: the Indiana University experience. *J Clin Oncol.* 1994;12(7):1390-1393.

Schindler G, Capper D, Meyer J, et al. Analysis of BRAF V600E mutation in 1,320 nervous system tumors reveals high mutation frequencies in pleomorphic xanthoastrocytoma, ganglioglioma and extra-cerebellar pilocytic astrocytoma. *Acta Neuropathol*. 2011;121:397-405.

Schneider BP, Kesler KA, Brooks JA, et al. Outcome of patients with residual germ cell or non-germ cell malignancy after resection of primary mediastinal nonseminomatous germ cell cancer. *J Clin Oncol* 2004;22(7):1195-1200.

Schonleben F, Qui W, Allendorf JD, Chabot JA, Remotii HE, Su GH. Molecular analysis of PIK3CA, BRAF, and RAS oncogenes in periampullary and ampullary adenomas and carcinomas. *J Gastrointest Surg*. 2009;13(8):1510-1516.

Seymour JF, Kurzrock R, Freireich EJ, et al. 2-chlorodeoxyadenosine induces durable remissions and prolonged suppression of CD4_ lymphocyte counts in patients with hairy cell leukemia. *Blood*. 1994;83:2906-2911.

Seymour JF, Talpaz M, Kurzrock R. Response duration and recovery of CD4_ lymphocytes following deoxycoformycin in interferon-alpha-resistant hairy cell leukemia: 7-Year follow-up. *Leukemia.* 1997;11:42-47.

Shimaoka K, Schoenfeld DA, Dewys WD, et al. A randomized trial of doxorubicin versus doxorubicin plus cisplatin in patients with advanced thyroid carcinoma. *Cancer.* 1985;56(9):2155-2160.

Siegel R, Naishadham D, Jemal A. Cancer Statistics, 2012. Cancer Journal for Clinicians. 2012;10-29. Doi:10.3322/caac.20138)

Sigal DS, Sharpe R, Burian C, Saven A. Very long term eradication of minimal residual disease in patients with hairy cell leukemia after a single course of cladribine. *Blood*. 2010;115:1893-1896.

Speranzaa G, Doroshow JH, Kummar S. Adenocarcinoma of the small bowel: changes in the landscape? Current Opin in Oncology. 2010;22:387-393.

Stein C. Inadmissibility of the Usual Estimator for the Mean of a Multivariate Normal Distribution. *Proc Third Berkeley Symp on Math Statist and Prob.*1956;1:197–206.

Su F, Viros A, Milagre C, et al. RAS-mutaions in cutaneous squamous-cell carcinomas in patients treated with BRAF inhibitors. *N Engl J Med;* 2012;366:207-215.

Swerdlow SH, Campo E, Harris NL, Jaffe ES, Pileri SA, Stein H, Thiele J, Vardiman JW. WHO Classification of Tumours of Haemotopoietic and Lymphoid Tissues. 4th ed. Geneva, Switzerland: IARC; 2008.

Tannapfel A, Sommerer F, Benicke M, et al. Mutations of the BRAF gene in cholangiocarcinoma but not in hepatocellular carcinoma. *Gut.* 2003;52:706-712.

The Criteria Committee of the New York Heart Association (NYHA). Nomenclature and Criteria for Diagnosis of Diseases of the Heart and Great Vessels. 9th Ed. Boston, Mass: Little, Brown & Co.; 1994:253-256.

Tiacci E, Trifonov V, Schiavoni G, et al. BRAF Mutations in Hairy Cell Leukemia. *N Engl J Med.* 2011;364:2305-2315.

Trials and mechanisms of resistance. *Clin Cancer Res.* 2012;18:33-39.

Valle J, Wasan H, Palmer DH, et al. Cisplatin plus Gemcitabine versus Gemcitabine for Biliary Tract Cancer. *N Engl J Med.* 2010;362:1273-1281.

van den Bent MJ, Wefel JS, Schiff D, et al. Response assessment in neuro-oncology (a report of the RANO group): assessment of outcome in trials of diffuse low grade gliomas. The Lancet. 2011;12:583-593.

Walker BA, Wardell CP, Melchor L, et al. Intraclonal heterogeneity and distinct molecular mechanisms characterize the development of t(4;14) and t(11:14) myeloma. *Blood*. 2012;120(5):1077-1086

Warth A, Kloor M, Schirmacher P, Blaker H. Genetics and epigenetics of small bowel adenocarcinoma: the interactions of CIN, MSI, and CIMP. *Modern Pathology*. 2011;24(4):564–570.

Weber JS, Flaherty KT, Infante JR, et al. Updated safety and efficacy results from a Phase I/II study of the oral BRAF inhibitor dabrafenib (GSK2118436) combined with the oral MEK 1/2 inhibitor trametinib (GSK1120212) in patients with BRAF-naive metastatic melanoma. *J Clin Oncol.* 2012;30:(suppl;abstr8510).

Weissbach L, Bussar-Maatz R, Mann K. The value of tumor markers in testicular seminomas: Results of a prospective multicenter study. *Eur Urol.* 1997;32:16-22.

Wellbrock C, Karasaides M, Marais R. The RAF proteins take center stage. *Nature Reviews Mol Cell Biol.* 2004;5:875-885.

Wen PY. Updated Response Assessment Criteria for High-Grade Gliomas: Response Assessment in Neuro-Oncology Working Group. *J Clin Oncol*. 2010;28(11):1963-1972.

Williams SD, Birch R, Einhorn LH, et al. Treatment of disseminated germ-cell tumors with cisplatin, bleomycin, and either vinblastine or etoposide. *N Engl J Med*. 1987;316(23):1435-1440.

Xi L, Arons E, Navarro W, et al. Both variant and IGHV4-34-expressing hairy cell leukemia lack the BRAF V600E mutation. Blood. 2012;119:3330-3332

21 APPENDICES

21.1 Appendix 1: ECOG Performance Status

Assessment of Eastern Cooperative Oncology Group (ECOG) performance status to evaluate daily living abilities is required at Screening as well as routinely throughout the treatment and at treatment discontinuation.

0 Fully active, able to carry on all pre-disease performance without restriction

1 Restricted in physically strenuous activity but ambulatory and able to carry out work of a light or sedentary nature, e.g., light house work, office work

2 Ambulatory and capable of all self care but unable to carry out any work activities; up and about more than 50% of waking hours

3 Capable of only limited self care, confined to bed or chair more than 50% of waking hours

4 Completely disabled; cannot carry on any self care; totally confined to bed or chair

Reference:

Oken MM, Creech RH, et al. Toxicity and response criteria of the Eastern Cooperative Oncology Group. *American J of Clinical Oncology.* 1982;5:649-655.

21.2 Appendix 2: NYHA Functional Classification System

The **New York Heart Association (NYHA) Functional Classification: Class I, II, III or IV Heart Failure** provides a simple way of classifying the extent of heart failure. It places subjects in one of 4 categories based on the level of limitation experienced during physical activity.

Class	Symptoms
Class I<br>(Mild)	No limitation of physical activity. Ordinary physical activity does not cause undue fatigue, palpitation or dyspnea (shortness of breath).
Class II<br>(Mild)	Slight limitation of physical activity. Comfortable at rest, but ordinary physical activity results in fatigue, palpitation or dyspnea.
Class III<br>(Moderate)	Marked limitation of physical activity. Comfortable at rest, but less than ordinary physical activity results in fatigue, palpitation or dyspnea.
Class IV<br>(Severe)	Unable to carry out any physical activity without discomfort. Symptoms of cardiac insufficiency at rest. If any physical activity is undertaken, discomfort is increased.

Reference:

The Criteria Committee of the New York Heart Association (NYHA). Nomenclature and Criteria for Diagnosis of Diseases of the Heart and Great Vessels. 9th Ed. Boston, Mass: Little, Brown & Co.; 1994:253-256.

21.3 Appendix 3: Cockcroft-Gault Formula forCreatinine Clearance

$$\text{Creatinine Clearance (mL/min)}^2 = \frac{\text{Q x (140 - age[yr]) x ideal body wt[kg]}^1}{\text{72 x serum creatinine [mg/dL]}}$$

Q = 0.85 for females

Q = 1.0 for males

OR

$$\text{Creatinine Clearance (mL/min)}^2 = \frac{\text{K x (140 - age[yr]) x ideal body wt[kg]}^1}{\text{serum creatinine [µmol/L]}}$$

K = 1.0 for females

K = 1.23 for males

1. Use ideal body weight (IBW) if body weight >30% of IBW. Otherwise, use body weight.
2. Creatinine clearance has a maximum value of 125 mL/min. Clearance values >125 mL/min by this calculation must use 125 mL/min in the Calvert formula to determine carboplatin dosing.

Calculation of IBW using the Devine Formula [Devine, 1974]:

Males = 50.0 kg + (2.3 kg x each inch over 5 ft) or 50.0 kg + (0.906 kg x each cm over 152.4 cm)

Females = 45.5 kg + (2.3 kg x each inch over 5 ft) or 45.5 kg + (0.906 kg x each cm over 152.4 cm)

Example:

- Male
- Actual Body Weight = 90.0 kg,
- Height = 68 inches

IBW = 50.0 + (2.3) (68 - 60) = 68.4 kg

This subject's actual body weight is >30% over IBW. Therefore, in this case, the subject's IBW of 68.4 kg should be used in calculating estimated creatinine clearance.

Reference:

Devine BJ. Case Number 25 Gentamicin Therapy: Clinical Pharmacy Case Studies. *Drug Intelligence and Clinical Pharmacy.* 1974;8:650-655.

21.4 Appendix 4: Pharmacogenetic (PGx) Research

Pharmacogenetics – Background

Pharmacogenetics (PGx) is the study of variability in drug response due to hereditary factors in populations. There is increasing evidence that an individual's genetic composition (i.e., genotype) may impact the pharmacokinetics (PK; absorption, distribution, metabolism, elimination), pharmacodynamics (PD; relationship between concentrations and pharmacologic effects or the time course of pharmacologic effects) and/or clinical outcome (in terms of efficacy and/or safety and tolerability). Some reported examples of PGx associations with safety/adverse events (AEs) include:

Drug	Disease	Gene Variant	Outcome
Abacavir	HIV [Hetherington, 2002; Mallal, 2002; Mallal, 2008]	HLA-B* 5701 *(Human Leukocyte Antigen B)*	Carriage of the HLA-B*5701 variant has been shown to increase a patient's risk for experiencing hypersensitivity to abacavir. Prospective HLA-B*5701 screening and exclusion of HLA-B*5701 positive patients from abacavir treatment significantly decreased the incidence of abacavir hypersensitivity. Treatment guidelines and abacavir product labeling in the United States (US) and Europe (EU) now recommend (US) or require (EU) prospective HLA-B*5701 screening prior to initiation of abacavir to reduce the incidence of abacavir hypersensitivity. HLA-B*5701 screening should supplement but must never replace clinical risk management strategies for abacavir hypersensitivity.

Drug	Disease	Gene Variant	Outcome
Carbamazepine	Seizure, Bipolar disorders & Analgesia Chung, 2010; Ferrell, 2008	HLA-B*1502	Independent studies indicated that patients of East Asian ancestry who carry *HLA-B*15:02* are at higher risk of Stevens-Johnson Syndrome and toxic epidermal necrolysis. Regulators, including the US Food and Drug Administration (FDA) and the Taiwanese FDA (TFDA), have updated the carbamazepine drug label to indicate that patients with ancestry in genetically at risk populations should be screened for the presence of HLA-B*1502 prior to initiating treatment with carbamazepine.
Irinotecan	Cancer [Innocenti, 2004; Liu, 2008; Schulz, 2009]	UGT1A1*28	Variations in the UGT1A1 gene can influence a patient's ability to break down irinotecan, which can lead to increased blood levels of the drug and a higher risk of side effects. A dose of irinotecan that is safe for one patient with a particular UGT1A1 gene variation might be too high for another patient without this variation, raising the risk of certain side-effects that include neutropenia following initiation of Irinotecan treatment. The irinotecan drug label indicates that individuals who have two copies of the UGT1A1*28 variant are at increased risk of neutropenia. A genetic blood test is available that can detect variations in the gene.

A key component to successful PGx research is the collection of samples during the conduct of clinical studies.

Collection of whole blood samples, even when no *a priori* hypothesis has been identified, may enable PGx analysis to be conducted if at any time it appears that there is a potential unexpected or unexplained variation in response to dabrafenib and/or trametinib treatment.

Pharmacogenetic Research Objectives

The objective of the PGx research (if there is a potential unexpected or unexplained variation) is to investigate a relationship between genetic factors and response to dabrafenib and/or trametinib treatment. If at any time it appears there is potential variability in response in this clinical study or in a series of clinical studies with dabrafenib and/or trametinib treatment that may be attributable to genetic variations of subjects, the following objectives may be investigated:

- The relationship between genetic variants and the pharmacokinetics and/or PD of study treatment.
- The relationship between genetic variants and safety and/or tolerability ofstudy treatment.
- The relationship between genetic variants and efficacy of study treatment.

Study Population

Any subject who is enrolled in the clinical study, can participate in PGx research. Any subject who has received an ABMT must be excluded from the PGx research.

Subject participation in the PGx research is voluntary and refusal to participate will not indicate withdrawal from the clinical study or result in any penalty or loss of benefits to which the subject would otherwise be entitled.

Study Assessments and Procedures

Blood samples can be taken for deoxyribonucleic acid (DNA) extraction and used in PGx assessments.

In addition to any blood samples taken for the clinical study, a whole blood sample (approximately 6 mL) will be collected from subjects who provide specific consent for the PGx research using a tube containing ethylenediamine tetraacetic acid (EDTA). It is recommended that the blood sample be taken at the first opportunity after a subject has been randomized and provided informed consent for PGx research, but may be taken at any time while the subject is participating in the clinical study.

- The PGx sample is labelled (or "coded") with a study specific number that can be traced or linked back to the subject by the investigator or site staff. Coded samples do not carry personal identifiers (such as name or social security number). The blood sample is taken on a single occasion unless a duplicate sample is required due to inability to utilise the original sample.

The DNA extracted from the blood sample may be subjected to sample quality control analysis. This analysis will involve the genotyping of several genetic markers to confirm

the integrity of individual samples. If inconsistencies are noted in the analysis, then those samples may be destroyed.

The need to conduct PGx analysis may be identified after a study (or set of studies) of dabrafenib and/or trametinib have been completed and the clinical study data reviewed. In some cases, the samples may not be studied. e.g., no questions are raised about how people respond to dabrafenib and/or trametinib treatment.

Samples will be stored securely and may be kept for up to 15 years after the last subject completes the study or Novartis may destroy the samples sooner. Novartis or those working with Novartis (for example, other researchers) will use samples collected from the study for the purpose stated in this protocol and in the informed consent form.

Subjects can request their sample to be destroyed at any time.

Subject Withdrawal from Study

If a subject who has consented to participate in PGx research and has a sample taken for PGx research withdraws from the clinical study for any reason other than lost to follow- up, the subject will be given the following options:

- The same is retained for PGx research.
- Any PGx sample is destroyed.

If a subject withdraws consent for PGx research or requests sample destruction for any reason, the investigator must complete the appropriate documentation to request sample destruction within the timeframe specified by Novartis and maintain the documentation in the site study records. The investigator should forward the PGx Sample Destruction Request Form to Novartis as directed on the form. This can be done at any time when a subject wishes to withdraw from the PGx research or have their sample destroyed whether during the study or during the retention period following close of the main study. In either case, Novartis will only keep study information collected/generated up to that point.

Screen and Baseline Failures

If a blood sample for PGx research has been collected and it is determined that the subject does not meet the entry criteria for participation in the clinical study, then the investigator

should instruct the participant that their PGx sample will be destroyed. No forms are required to complete this process as it will be completed as part of the consent and sample reconciliation process. In this instance a sample destruction form will not be available to include in the site files.

Pharmacogenetics Analyses

The aim of PGx analyses will be to investigate the relationship between genetic variations in germline DNA and response to study treatment. The specific type of genetic investigation to be applied will be dependent on the most scientifically feasible approach available to address understanding of the response to the study treatment.

Generally, two approaches may be utilized to explore the association of genetic variation:

- Candidate gene analysis where variations in specific genes may be studied that encode the drug targets, or drug mechanism of action pathways, drug metabolizing enzymes, drug transporters, or which may underpin adverse events, disease risk or drug response. These candidate genes may include a common set of ADME (Absorption, Distribution, Metabolism and Excretion) genes that are studied to determine the relationship between gene variants and treatment response and/or tolerance.

In addition, continuing research may identify other enzymes, transporters, proteins or receptors that may be involved in response to treatment with dabrafenib and/or trametinib. The genes that may code for these proteins may also be studied.

- Genome-wide scans involving a large number of polymorphic markers (e.g., single nucleotide polymorphisms) at defined locations in the genome, often correlated with a candidate gene, may be studied to determine the relationship between genetic variants and treatment response or tolerance. This approach is often employed when a definitive candidate gene(s) does not exist and/or the potential genetic effects are not well understood.

If applicable and PGx research is conducted, appropriate statistical analysis methods will be used to evaluate PGx data in the context of the other clinical data. Results of PGx investigations will be reported either as part of the main clinical study report or as a separate report. Endpoints of interest from all comparisons will be descriptively and/or graphically summarized as appropriate to the data. A detailed description of the analysis to be performed will be documented in the study reporting and analysis plan (RAP) or in a separate pharmacogenetics RAP, as appropriate.

Informed Consent

Subjects who do not wish to participate in the PGx research may still participate in the clinical study. PGx informed consent must be obtained prior to any blood being taken for PGx research.

Provision of Study Results and Confidentiality of Subject's PGx Data

Novartis may summarize the PGx research results in the clinical study report, or separately, and may publish the results in scientific journals.

Novartis does not inform the investigator, subject, or anyone else (e.g., family members, study investigators, primary care physicians, insurers, or employers) of individual genotyping results that are not known to be relevant to the subject's medical care at the time of the study, unless required by law. This is due to the fact that the information generated from PGx

studies is generally preliminary in nature, and therefore the significance and scientific validity of the results are undetermined

References

Chung WH, Hung SL, Chen YT. Genetic predisposition of life-threatening antiepileptic-induced skin reactions. Expert Opin. Drug Saf. 2010; 9: 15-21.

Ferrell PB, McLeod HL. Carbamazepine, HLA-B*1502 and risk of Stevens-Johnson syndrome and toxic epidermal necrolysis: US FDA recommendations. Pharmacogenomics. 2008; 9: 1543-1546.

Hetherington S, Hughes AR, Mosteller M, Shortino D, Baker KL, Spreen W, Lai E, Davies K, Handley A, Dow DJ, Fling ME, Stocum M, Bowman C, Thurmond LM, Roses AD. Genetic variations in HLA-B region and hypersensitivity reactions to abacavir. *Lancet*. 2002; 359:1121-1122.

Innocenti F, Undevia SD, Iyer L, Chen PX, Das S, Kocherginsky M, Karrison T, Janisch L, Ramirez J, Rudin CM, Vokes EE, Ratain MJ. Genetic variants in the UDP-glucuronosyltransferase 1A1 gene predict the risk of severe neutropenia of irinotecan. J Clin Oncol 2004; 22: 1382-1388.

Liu CY, Chen PM, Chiou TJ, Liu JH, Lin JK, Lin TC, Chen WS, Jiang JK, Wang HS, Wang WS. UGT1A1*28 polymorphism predicts irinotecan-induced severe toxicities without affecting treatment outcome and survival in patients with metastatic colorectal carcinoma. Cancer. 2008; 112: 1932-1940.

Mallal S, Nolan D, Witt C, Masel G, Martin AM, Moore C, Sayer D, Castley A, Mamotte C, Maxwell D, James I. Association between presence of HLA-B*5701, HLA-DR7, and HLA-DQ3 and hypersensitivity to HIV-1 reverse-transcriptase inhibitor abacavir. *Lancet*. 2002; 359:727-32.

Mallal S, Phillips E, Carosi G, Molina JM, Workman C, Tomazic J, Jägel-Guedes E, Rugina S, Kozyrev O, Cid JF, Hay P, Nolan D, Hughes S, Hughes A, Ryan S, Fitch N, Thorborn D, Benbow A; PREDICT-1 Study Team. HLA-B*5701 screening for hypersensitivity to abacavir. N Engl J Med. 2008; 358; 568-579

Schulz C, Heinemann V, Schalhorn A, Moosmann N, Zwingers T, Boeck S, Giessen C, Stemmler HJ. UGT1A1 gene polymorphism: Impact on toxicity and efficacy of irinotecan-based regimens in metastatic colorectal cancer. World J. Gastroenterol. 2009; 15: 5058-5066.

[redacted]

[redacted]

[redacted]

21.6 Appendix 6: Details of Bayesian Hierarchical Model

Y_i is the response indicator for the ith subject, and $\pi_j = P(Y_i = 1 \mid j_i = j)$ is the true response rate for histology j. In the logic scale, θ_j is the mean log odds treatment effect of response:

$$\theta_j = \log\left(\frac{\pi_j}{1-\pi_j}\right) - \log\left(\frac{r_{,j}}{1-r_{,j}}\right)$$

Basic Hierarchical Model

A basic hierarchical model structure is described here and is incorporated within the clustered hierarchical model framework used in this study.

Borrowing occurs to the extent indicated by the data using a hierarchical normal model on the treatment effects:

$$\theta_j \sim N(\mu, \tau^2).$$

Were only this basic hierarchical model used for analysis, the prior distributions would be:

$$\mu \sim N(0,3) \text{ and } \tau^2 \sim IG(0.5, 0.125),$$

where $IG(\alpha,\beta)$ is the inverse gamma distribution defined by:

$$f(x|\alpha,\beta) = \frac{\beta^{\alpha} e^{-\beta/x}}{x^{\alpha+1}\,\Gamma(\alpha)},$$

This distribution is non-informative in the sense that its parameters are analogous to having a single observation. The variance component τ is a key parameter that plays a role in the degree of borrowing among histologies. Small values of τ result in a greater degree of borrowing while large values of τ correspond to less borrowing. The distribution of τ is based on the variability of the observations, and the observed between- histology variation is an important component of the model's performance. The effects of any particular prior distribution for τ can be assessed via simulations in evaluating a design's operating characteristics, type I error rate and statistical power.

Clustered Hierarchical Model

A clustered hierarchical model considers the possibility that "clusters" exist among the collection of histologies. A therapy may be effective for some histologies but not for others. Histologies within the same cluster have greater influence on each other than they do on histologies in other clusters. On the other hand, histologies in different clusters are conditionally independent given any particular configuration of clusters. There is borrowing at the cluster level and borrowing across histologies within a particular cluster.

Borrowing across clusters depends only on similarities between clusters and not of individual histologies.

The number of clusters is unknown in advance but information will be gleaned from the data using Dirichlet Process Mixtures (DPM). The full prior is constructed in 2 stages. In thefirst

stage, the histologies are assigned to particular clusters, with the number of clusters unspecified. Conditional on this particular clustering, the hierarchical model is fit within each cluster. A difference from the basic hierarchical model is in the parameter τ. Each cluster has its own τ. And the prior distribution on τ is now different:

$$rr^2 \sim \mathrm{llll}(3, 0.5).$$

Without the clustering, an informative prior on τ that places high probability on small values of τ could yield conclusions similar to simple pooling across histologies. This is not desirable. Instead its preferred to preserve the integrity of individual histologies to the extent that they give dissimilar results. With the clustering, a prior such as the one selected above meets this desideratum. When τ is small, as evinced by the data, histologies will only be placed in the same cluster if the data for those histologies are sufficiently similar to other histologies in the cluster. When τ is large, the histologies will be assigned to separate clusters and response rates estimated separately. This clustering approach provides further protection against borrowing between dissimilar histologies while enhancing the borrowing between similar histologies.

The prior distribution in a DPM is governed by the parameter α. When α is small, the prior favors large clusters. As α tends to zero, the prior tends to place all its mass on a single cluster containing all the histologies. As α increases, the prior places more mass on clustering with a large number of clusters. As α becomes large, the prior places all of its mass on having a separate cluster for each histology (thus, no borrowing across histologies). A value of $\alpha = 0$ corresponds to the usual hierarchical model with no clustering, while a very large α treats all the histologies separately. Common values of α might be between 0.5 and 5. We have selected $\alpha = 2$ for the final model. This value has been used for the final algorithm and the operating characteristics including type I error and power.

The key aspect of the clustering portion of the prior involves which histologies are clustered together. This is accomplished by creating a sequence of bins (in theory there are infinitely many bins, but the number of bins actually used cannot exceed the number of histologies). Thus imagine a sequence of bins C1,C2,C3,… with associated probabilities p_{C1},p_{C2},p_{C3} and so forth. In the prior, each histology is placed in a bin according to the p_{Ck} probabilities. Thus, if one p_{Ck} is near 1, then all histologies will be likely to randomly fall in the same bin. If all the p_{Ck} values are small, it will be likely that all the histologies will randomly fall in separate bins. Histologies that fall within the same bin are defined as being in the same cluster. More details may be found in Escobar 1995 or Neal 2000.

The p_{Ck} probabilities are determined through a stick breaking process [Sethuraman, 1994]. Let p_{C1}~Beta(1,α). This leaves 1-p_{C1} probability to be allocated to the remaining bins. Let p_{C2} be a Beta(1,α) proportion of that remaining mass, so p_{C2}~Beta(1,α)*[1-p_{C1}]. There is now 1-p_{C1}-p_{C2} mass unallocated. Let p_{C3} be a Beta(1,α) proportion of the remaining mass, so p_{C3}~Beta(1,α)*[1-p_{C1}-p_{C2}] and so on for p_{C4}, p_{C5}, and so forth. This process generates an infinite sequence of p_{Ck} values, but generally the first few contain most of the probability. In terms of the posterior distribution, only the bins which contain the histologies are of interest, and thus only 9 of the infinite bins will actually be used.

The role of α can be seen from this construction. If α is very small, then the Beta(1,α) proportions will tend to be close to 1. Thus, the first p_{C1} is quite likely to be near 1, and thus all the histologies will tend to lie in the first bin. If α is very large, all the Beta(1,α) values will tend to be small, and thus each p_{Ck} will tend to be small. This creates a situation where

each histology is likely to be in a separate bin. Our proposed value of α=2 avoids these forced extremes and allows the data to drive the posterior distribution.

After the histologies have been assigned to bins in the prior, histologies within the same bin will tend to have more similar data than histologies in separate bins. Thus, in the posterior distribution, histologies with similar data are viewed as more likely to be from the same bin (and hence the same cluster). Histologies with very different data, in contrast, will have high posterior probability of being in separate bins/clusters, and thus will be estimated separately.

The posterior distribution also can be thought of in 2 stages, the first being a posterior distribution on the clustering, and then conditional on the clustering the posterior distribution from the hierarchical model. Conditional on the clustering, borrowing happens within clusters, but not across clusters. In situations where the clustering is uncertain (always in practice), one will see a proportional amount of borrowing between histologies, proportional on the posterior probability the histologies are within the same cluster. The aim of the clustering is to identify situations where the drug generally works for some histologies and generally does not work for others, so that these two disparate effects are not averaged together through the borrowing.

Reference:

Sethuraman J. A Constructive Definition of Dirichlet Priors. *Statistica Sinica*. 1994;4:639-650.

21.7 Appendix 7: Supporting SimulationResults

Operating Characteristics for the Clustered Hierarchical Design for Different Sample Sizes: Power, Type I Error Rate

	Histology								
Scenario	1	2	3	4	5	6	7	8	9
	Proportion of Trials that Declare each Histology Efficacious (Measure of Power or Type I Error Rate)								
Sample size Case 2	17	16	20	5	16	8	5	17	5
Mixed1	0.17	0.74	0.98	0.87	0.9	0.81	0.17	0.48	0.53
5 Greats: Mixed1	0.1	0.94	0.96	0.82	0.11	0.85	0.12	0.1	0.75
4 Greats: Low	0.09	0.08	0.08	0.77	0.08	0.82	0.68	0.07	0.65
2 Greats: Mixed1	0.08	0.06	0.94	0.08	0.08	0.06	0.61	0.07	0.04
Sample size Case 3	20	16	25	5	16	8	5	20	5
Mixed1	0.18	0.77	0.98	0.85	0.9	0.82	0.15	0.5	0.51
5 Greats: Mixed1	0.12	0.95	0.97	0.82	0.12	0.86	0.1	0.11	0.72
4 Greats: Low	0.09	0.08	0.09	0.78	0.08	0.82	0.67	0.08	0.67
2 Greats: Mixed1	0.07	0.07	0.92	0.06	0.06	0.04	0.62	0.06	0.05
Sample size Case 4	20	10	25	2	8	5	2	20	2
Mixed1	0.16	0.65	0.96	0.44	0.78	0.64	0.1	0.47	0.28
5 Greats: Mixed1	0.12	0.89	0.95	0.43	0.09	0.71	0.06	0.1	0.35
4 Greats: Low	0.08	0.06	0.07	0.37	0.06	0.59	0.28	0.08	0.26

**2 Greats: Mixed1**	0.08	0.06	0.93	0.04	0.06	0.04	0.27	0.07	0.02

21.8 Appendix 8: Hypothetical Example Trial Results

For each example trial, a table describing the observed number of responses, sample size, and any stopping decisions are presented at each interim analysis. A second table describes the final analysis. Rows indicate individual histologies. Columns indicate the assumed historical control and clinically meaningful response rates for that particular histology, the number of responders out of the total number of subjects enrolled within the particular histology, the observed ORR, the estimated ORR, the probability of declaring success at the final analysis based on the hierarchical analysis, and the final outcome based on the statistical decision.

Additionally, for each example trial a trellis plot is provided, whereby sequentially over time, each interim analysis as well as the final analysis is presented. Each analysis contains a scatter plot with histology on the x-axis (1 to 9) and observed ORR on the y- axis. The size of the bubble reflects the number of subjects included in that analysis at that time (the larger the bubble, the more subjects). If the bubble is blue, then the histology reached no interim decision and continued on. If the bubble is red, then that histology met the futility boundary and ceased enrollment at that interim. If the bubble is green, then that histology met the threshold for stopping enrollment due to efficacy. If enrollment is stopped early at an interim analysis, all future interims continue to reflect that early decision. At the final analysis, histologies must either meet criteria for success orfutility.

Example 1: Responsive Scenario, Most Histologies Declared Successful

In this example, all histologies demonstrate relatively high ORR's. At the final analysis, the ORRs for eight of the histologies range from 29% to 67%. All these histologies meet the defined statistical success criteria. To demonstrate evidence of meaningful high levels of activity in these eight histologies, it will be critical that in addition to statistical success, the totality of the data support clinically relevant results. Two of these histologies (histologies 1 and 3) halted enrollment early for success. One histology (histology 9) had one responders out of three subjects, and was not declared successful.

Example 1: Responses/N at each Interim Analysis

	Interim Analysis										
**Histology**	**1**	**2**	**3**	**4**	**5**	**6**	**7**	**8**	**9**	**10**	**11**
1	2/4	4/7	4/7	4/8	4/8	4/10	4/11	4/11	4/11	4/11	4/11
2	0/1	0/1	0/2	1/3	1/3	1/3	2/4	2/4	2/4	2/5	2/5
3	1/2	1/2	1/5	2/6	2/6	2/6	3/7	3/7	3/9	3/10	3/10
4	0/1	0/1	0/1	0/1	0/1	0/1	0/2	0/2	0/2	0/2	2/4
5	1/1	1/1	1/2	1/2	1/2	1/2	1/2	1/2	1/2	1/2	2/3
6	0/1	0/1	0/1	0/1	1/2	1/4	1/4	1/4	1/4	1/4	2/5
7	0/1	0/1	0/1	0/1	0/1	0/2	0/2	1/3	1/3	2/5	3/6
8	0/1	1/2	1/2	1/2	1/2	1/3	2/4	2/4	2/5	3/6	5/8
9	0/1	0/2	0/2	0/2	0/2	0/2	1/3	1/3	1/3	1/3	1/3

Note: Color indicates enrollment status for each histology. Green indicates stopping enrollment for efficacy, red indicates stopping enrollment for futility, clear indicates continued enrollment.

Example 1: Information at the Final Analysis

Histology	Historical Control/ Clinically Meaningful RRs	Responses/N	Observed RR	Estimated RR	Probability of Success	Final Decision
1	10% / 50%	4/11	0.36	0.37	1	Success
2	10% / 50%	2/7	0.29	0.33	0.98	Success
3	15% / 60%	3/10	0.30	0.38	0.96	Success
4	10% / 50%	3/5	0.60	0.44	1	Success
5	10% / 50%	2/3	0.67	0.43	1	Success
6	10% / 50%	2/5	0.40	0.38	0.99	Success
7	10% / 50%	3/6	0.50	0.41	1	Success
8	15% / 60%	5/8	0.62	0.54	1	Success
9	25% / 75%	1/3	0.33	0.51	0.88	Futility

Abbreviation: RR = response rate

Note: Color indicates the model-based statistical decision for each histology. Green indicates success while red indicates futility.

Example 1: Observed ORR at each Interim Analysis and the Final Analysis

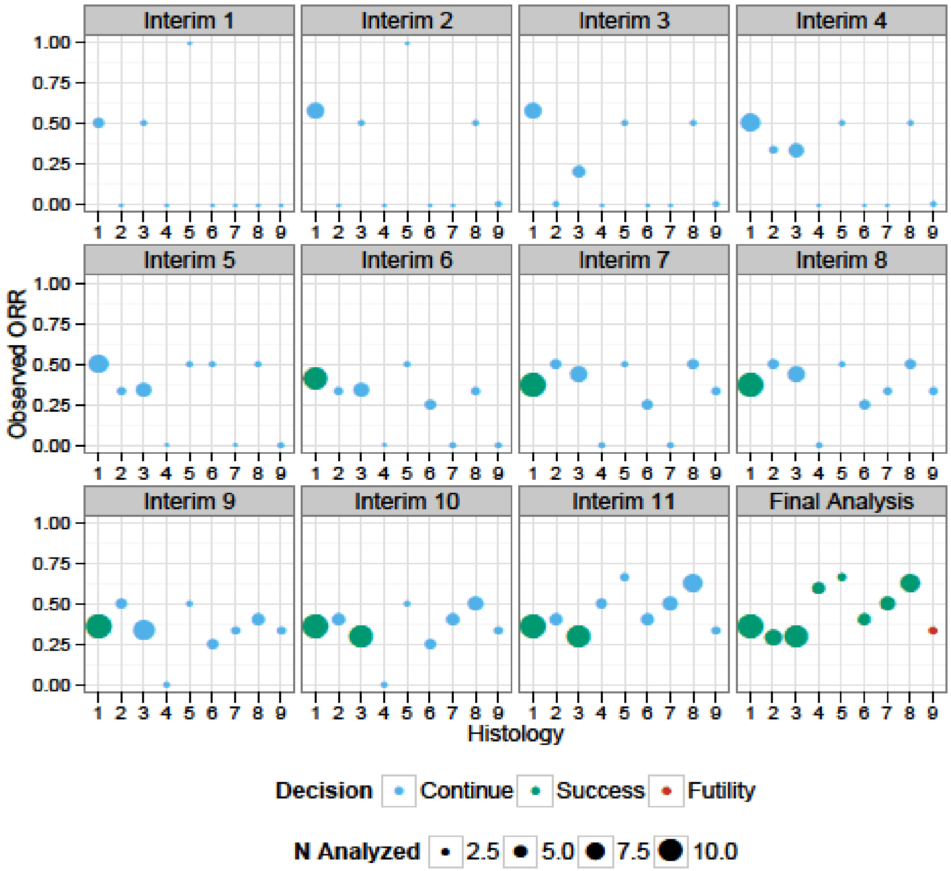

Example 2: Non-responsive Scenario: No Histologies Declared Successful

In this example, histologies demonstrate relatively low ORRs. At the final analysis, most the ORRs range from 0 to 33%. The only exception is histology nine with a 67% ORR (2/3). No histologies declare success. Three of the nine histologies halted enrollment early for futility during interim analyses.

Example 2: Responses/N at each Interim Analysis

	Interim Analysis									
Histology	1	2	3	4	5	6	7	8	9	10
1	0/4	0/6	0/6	0/6	0/6	0/6	0/6	0/6	0/6	0/6
2	0/2	0/2	0/3	0/5	0/6	0/6	0/6	0/6	0/6	0/6
3	1/2	1/4	1/6	1/6	1/6	1/7	1/7	1/9	1/9	1/9
4	0/2	0/2	0/3	0/4	0/5	0/5	0/5	0/5	0/5	0/5
5	0/1	0/2	0/2	0/2	0/2	0/2	0/2	0/3	0/3	0/3
6	0/0	0/0	0/1	0/1	0/2	0/2	0/2	1/3	1/3	1/4
7	0/0	0/0	0/1	0/1	0/1	0/3	0/3	0/3	0/3	0/3
8	0/0	0/0	0/0	0/0	0/0	1/1	1/3	1/3	1/3	1/3
9	1/1	1/1	1/1	2/2	2/2	2/2	2/2	2/2	2/2	2/3

Note: Color indicates enrollment status for each histology. Green indicates stopping enrollment for efficacy, red indicates stopping enrollment for futility, clear indicates continued enrollment.

Example 2: Information at the Final Analysis

Histology	Historical Control/ Clinically Meaningful RRs	Responses/N	Observed RR	Estimated RR	Probability of Success	Final Decision
1	10% / 50%	0/6	0	0.07	0.21	Futility
2	10% / 50%	0/6	0	0.06	0.20	Futility
3	15% / 60%	1/10	0.10	0.12	0.28	Futility
4	10% / 50%	0/5	0	0.07	0.23	Futility
5	10% / 50%	0/3	0	0.08	0.28	Futility
6	10% / 50%	1/4	0.25	0.16	0.59	Futility
7	10% / 50%	0/3	0	0.08	0.29	Futility
8	15% / 60%	1/3	0.33	0.22	0.59	Futility
9	25% / 75%	2/3	0.67	0.42	0.77	Futility

Abbreviation: RR = response rate

Note: Color indicates the model-based statistical decision for each histology. Red indicates futility.

Example 2: Observed ORR at each Interim Analysis and the Final Analysis

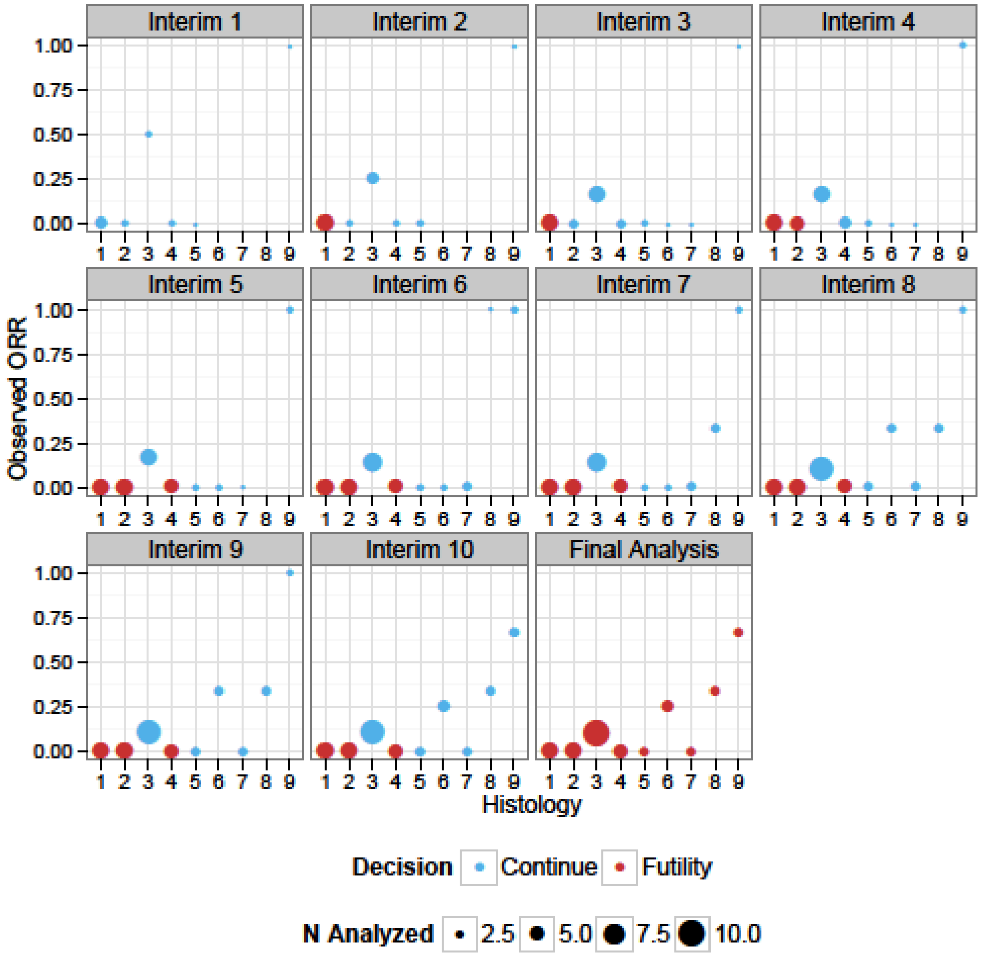

Example 3: Mixed Scenario, Most Histologies Declared Successful

In this example, the nine histologies demonstrate varying levels of responsiveness. Most histologies meet the criteria for statistical success at the final analysis.

Six histologies have ORRs ranging from 30% to 67%. All of these histologies declare success. Due to moderate ORRs in some of these histologies, it will be particularly critical that the entirety of the data, including secondary efficacy endpoints, support clinically relevant results to demonstrate evidence of meaningful high levels of activity.

The ORR for the remaining three histologies is 0, 14% and 29%, none of which is sufficient to meet the pre-specified success criteria. Two histologies halted enrollment early for success and one for futility.

Example 3: Responses/N at each Interim Analysis

	Interim Analysis									
**Histology**	**1**	**2**	**3**	**4**	**5**	**6**	**7**	**8**	**9**	**10**
1	2/4	3/7	3/10	3/10	3/10	3/10	3/10	3/10	3/10	3/10
2	1/4	2/5	2/5	2/5	2/5	2/5	2/6	4/10	4/11	4/11
3	0/4	0/4	0/5	0/5	0/5	0/5	0/5	0/6	0/7	0/9
4	0/1	0/2	0/2	0/2	0/3	0/3	0/5	0/5	0/5	0/5
5	0/0	0/0	0/0	0/0	0/1	0/2	0/2	1/4	1/4	2/6
6	1/1	1/1	1/1	1/2	1/3	1/4	2/5	2/5	2/6	2/6
7	1/1	1/2	1/2	1/2	2/5	2/6	2/6	2/6	2/8	2/8
8	1/1	1/1	1/1	1/1	2/2	2/2	2/2	2/2	2/3	2/3
9	0/1	0/2	1/4	1/4	1/5	2/7	2/7	2/7	2/7	2/7

Note: Color indicates enrollment status for each histology. Green indicates stopping enrollment for efficacy, red indicates stopping enrollment for futility, clear indicates continued enrollment.

Example 3: Information at the Final Analysis

Histology	Historical Control/ Clinically Meaningful RRs	Responses/N	Observed RR	Estimated RR	Probability of Success	Final Decision
1	10% / 50%	3/10	0.30	0.28	0.97	Success
2	10% / 50%	4/11	0.36	0.31	0.99	Success
3	15% / 60%	0/9	0	0.11	0.28	Futility
4	10% / 50%	1/7	0.14	0.21	0.83	Futility
5	10% / 50%	2/6	0.33	0.28	0.96	Success
6	10% / 50%	2/6	0.33	0.29	0.96	Success
7	10% / 50%	3/9	0.33	0.29	0.98	Success
8	15% / 60%	2/3	0.67	0.43	0.98	Success
9	25% / 75%	2/7	0.29	0.37	0.77	Futility

Abbreviation: RR = response rate

Note: Color indicates the model-based statistical decision for each histology. Green indicates success while red indicates futility.

Example 3: Observed ORR at each Interim Analysis and the Final Analysis

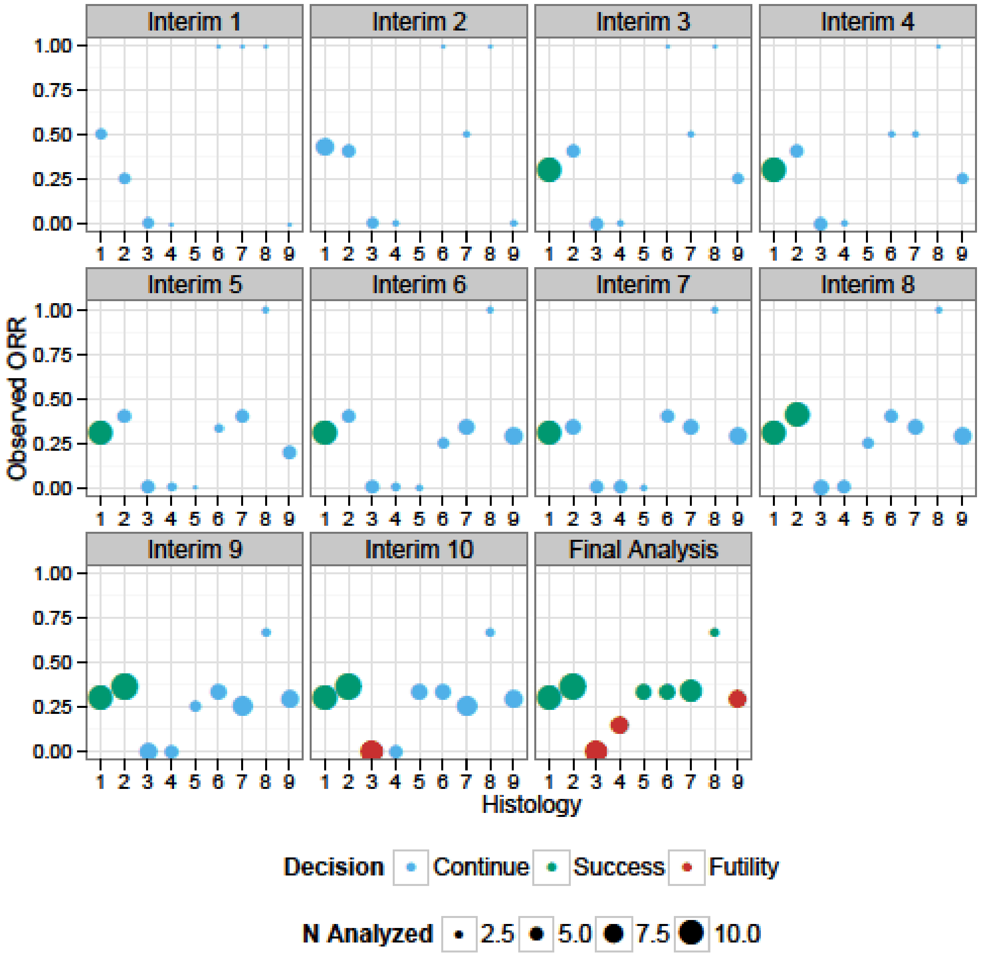

Example 4: Mixed Scenario, Most Histologies not Declared Successful

In this example, seven of the histologies demonstrate a range of ORRs from 0 to 31%. The remaining two histologies (histologies 7 and 9) have ORRs of 71% and 80% respectively. Three of the histologies (histologies 1, 7, and 9) which demonstrated the highest ORRs of 31%, 71%, and 80%, respectively, meet statistical success. To demonstrate evidence of meaningful high levels of activity in these three histologies, it will be critical that in addition to statistical success, the totality of the data support clinically relevant results.

The remaining size histologies with ORRs of ≤29% do not meet the pre-specified success criteria. Histology 1 stopped early for success while two histologies (2 and 3) stopped early for futility.

Example 4: Responses/N at each Interim Analysis

	Interim Analysis									
Histology	1	2	3	4	5	6	7	8	9	10
1	1/4	1/5	2/7	2/7	2/8	2/8	3/10	4/13	4/13	4/13
2	0/3	0/3	0/4	0/4	0/5	0/7	0/8	0/8	0/8	0/8
3	0/2	0/3	0/3	0/3	0/4	0/4	0/4	0/5	0/5	0/5
4	0/0	0/0	0/0	0/0	1/2	1/3	2/4	2/6	2/6	2/7
5	1/1	1/2	1/3	1/5	1/7	1/8	1/10	1/10	1/10	1/10
6	0/0	0/0	0/0	0/0	0/1	0/2	0/2	0/3	0/3	0/3
7	0/1	0/1	0/1	1/2	3/5	4/6	4/6	4/6	5/7	5/7
8	0/1	0/1	0/2	1/4	1/4	1/4	1/4	1/4	1/4	1/4
9	1/1	1/1	1/1	1/1	1/2	2/3	3/4	4/5	4/5	4/5

Note: Color indicates enrollment status for each histology. Green indicates stopping enrollment for efficacy, red indicates stopping enrollment for futility, clear indicates continued enrollment.

Example 4: Information at the Final Analysis

Histology	Historical Control/ Clinically Meaningful RRs	Responses/N	Observed RR	Estimated RR	Probability of Success	Final Decision
1	10% / 50%	4/13	0.31	0.29	0.97	Success
2	10% / 50%	0/8	0	0.07	0.27	Futility
3	15% / 60%	0/5	0	0.11	0.28	Futility
4	10% / 50%	2/7	0.29	0.26	0.88	Futility
5	10% / 50%	1/10	0.1	0.13	0.53	Futility
6	10% / 50%	0/5	0	0.09	0.35	Futility
7	10% / 50%	5/7	0.71	0.52	1	Success
8	15% / 60%	1/4	0.25	0.27	0.73	Futility
9	25% / 75%	4/5	0.80	0.65	0.99	Success

Abbreviation: RR = response rate

Note: Color indicates the model-based statistical decision for each histology. Green indicates success while red indicates futility.

Example 4: Observed ORR at each Interim Analysis and the Final Analysis

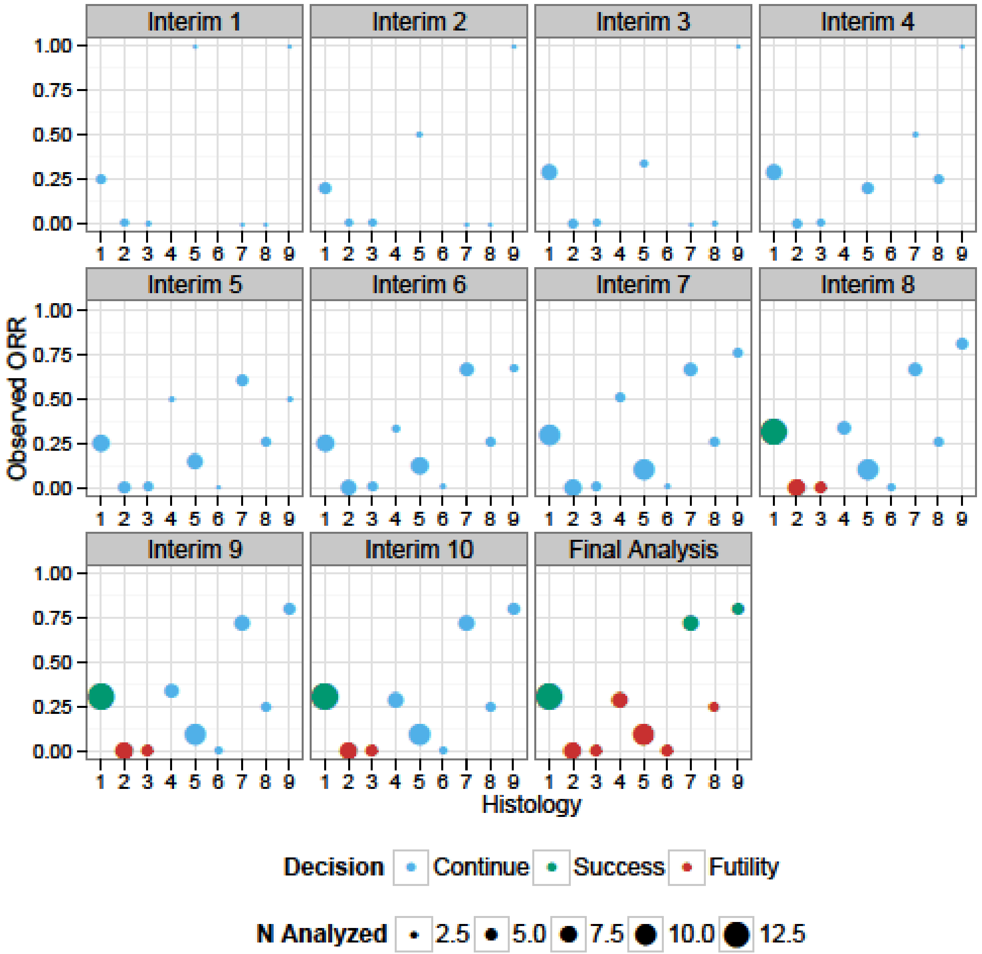

Example 5: Mixed Scenario where Roughly Half the Histologies are Declared Successful

In this example, there is a broad range of ORRs across the histologies, from 0 to 83%. Four histologies (histologies 1, 3, 7, and 9) have ORRs of 42%, 38%, 33%, and 83% respectively, and statistical success is declared in each. To demonstrate evidence of meaningful high levels of activity in these four histologies, it will be critical that in addition to statistical success, the totality of the data support clinically relevant results. Histology 1 had met the criteria for early stopping for efficacy.

The remaining five histolgies (histology 2, 4, 5, 6 and 8) have ORRs ranging from 0 to 20%. The pre-specified success criteria are not met in any of these. No histologies stopped early for futility

Example 5: Responses/N at each Interim Analysis

		Interim Analysis										
**Histology**	**1**	**2**	**3**	**4**	**5**	**6**	**7**	**8**	**9**	**10**	**11**	**12**
1	2/3	3/7	4/8	4/8	4/8	4/10	5/12	5/12	5/12	5/12	5/12	5/12
2	0/0	0/0	0/1	0/1	1/3	1/3	1/5	1/5	1/6	1/10	1/11	1/11
3	0/0	0/0	0/0	1/2	1/2	2/4	2/4	2/4	2/4	2/5	2/5	2/6
4	0/0	0/0	0/0	0/0	0/0	0/0	0/2	0/2	0/2	0/5	0/6	0/7

5	0/1	0/3	0/3	0/3	0/3	0/3	0/3	0/3	0/4	0/5	0/6	0/6
6	0/0	0/1	0/1	1/2	1/2	1/2	1/3	1/4	1/4	1/4	1/5	1/5
7	1/1	2/2	2/2	2/2	2/3	2/3	2/3	2/4	2/6	2/6	2/6	2/6
8	0/0	0/1	0/1	0/1	0/1	0/1	0/1	0/1	0/1	0/2	0/2	0/2
9	0/1	1/2	1/2	1/2	1/2	2/3	2/3	2/3	2/3	3/4	5/6	5/6

Note: Color indicates enrollment status for each histology. Green indicates stopping enrollment for efficacy, clear indicates continued enrollment.

Example 5: Information at the Final Analysis

Histology	Historical Control/ Clinically Meaningful RRs	Responses/N	Observed RR	Estimated RR	Probability of Success	Final Decision
1	10% / 50%	5/12	0.42	0.35	1	Success
2	10% / 50%	1/11	0.09	0.13	0.56	Futility
3	15% / 60%	3/8	0.38	0.36	0.94	Success
4	10% / 50%	0/7	0	0.09	0.34	Futility
5	10% / 50%	0/6	0	0.09	0.36	Futility
6	10% / 50%	1/5	0.20	0.21	0.77	Futility
7	10% / 50%	2/6	0.33	0.29	0.94	Success
8	15% / 60%	0/2	0	0.19	0.49	Futility
9	25% / 75%	5/6	0.83	0.65	1	Success

Abbreviation: RR = response rate

Note: Color indicates the model-based statistical decision for each histology. Green indicates success while red indicates futility.

Example 5: Observed ORR at each Interim Analysis and the Final Analysis

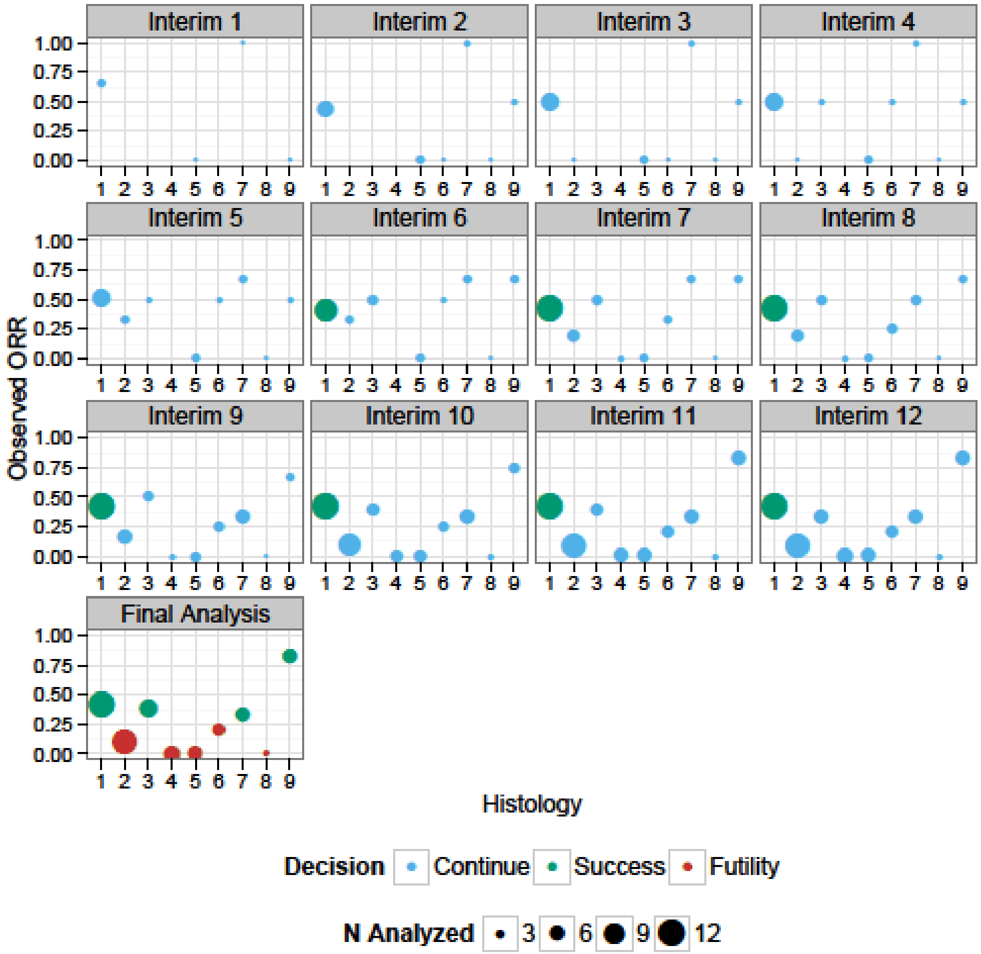

Example 6: Mixed Scenario, Most of the Low-enrolling Histologies Declare Success while the Higher-enrolling Histologies Do Not

In this example, most of the lower enrolling cohorts (three to six subjects each) have relatively high ORRs while the higher enrollment cohorts demonstrate low ORRs.

Four low-enrolling histologies (histology 6, 7, 8 and 9) have ORRs of 50%, 50%, 67%, and 75% respectively, and statistical success is declared in each. To demonstrate evidence of meaningful high levels of activity in these three histologies, it will be critical that in addition to statistical success, the totality of the data support clinically relevant results.

The four histologies (histologies 1, 2, 3, and 4) expected to enroll higher numbers of patients demonstrate much lower ORRs, ranging from 0-12% and none declare success. Though the actual sample sizes aren't very large (five to eight subjects), three of these histologies stopped early due to futility and larger sample sizes would likely have been possible had they not halted enrollment early. The remaining histology (histology 5) has an ORR of 33% (1/3) which also does not declare success.

Example 6: Responses/N at each Interim Analysis

	Interim Analysis							
Histology	1	2	3	4	5	6	7	8
1	0/0	0/0	0/2	0/3	0/5	0/6	0/6	0/6
2	1/3	1/3	1/4	1/6	1/6	1/6	1/7	1/8
3	0/2	0/6	0/7	0/7	0/7	0/7	0/7	0/7
4	0/3	0/5	0/5	0/5	0/5	0/5	0/5	0/5
5	1/2	1/2	1/2	1/2	1/2	1/3	1/3	1/3
6	0/0	0/0	0/0	0/0	1/2	2/4	2/4	2/4
7	1/2	1/2	1/2	1/2	1/3	2/4	2/4	2/5
8	2/2	3/3	3/3	4/4	4/5	4/5	4/5	4/5
9	2/2	2/2	2/2	2/2	2/2	2/2	3/3	3/4

Note: Color indicates enrollment status for each histology. Green indicates stopping enrollment for efficacy, red indicates stopping enrollment for futility, clear indicates continued enrollment.

Example 6: Information at the Final Analysis

Histology	Historical Control/ Clinically Meaningful RRs	Responses/N	Observed RR	Estimated RR	Probability of Success	Final Decision
1	10% / 50%	0/6	0	0.08	0.30	Futility
2	10% / 50%	1/8	0.12	0.17	0.64	Futility
3	15% / 60%	0/7	0	0.09	0.19	Futility
4	10% / 50%	0/5	0	0.10	0.36	Futility
5	10% / 50%	1/3	0.33	0.31	0.87	Futility
6	10% / 50%	2/4	0.50	0.38	0.97	Success
7	10% / 50%	3/6	0.50	0.41	0.99	Success
8	15% / 60%	4/6	0.67	0.54	1.00	Success
9	25% / 75%	3/4	0.75	0.63	0.98	Success

Abbreviation: RR = response rate

Note: Color indicates the model-based statistical decision for each histology. Green indicates success while red indicates futility.

Example 6: Observed ORR at each Interim Analysis and the Final Analysis

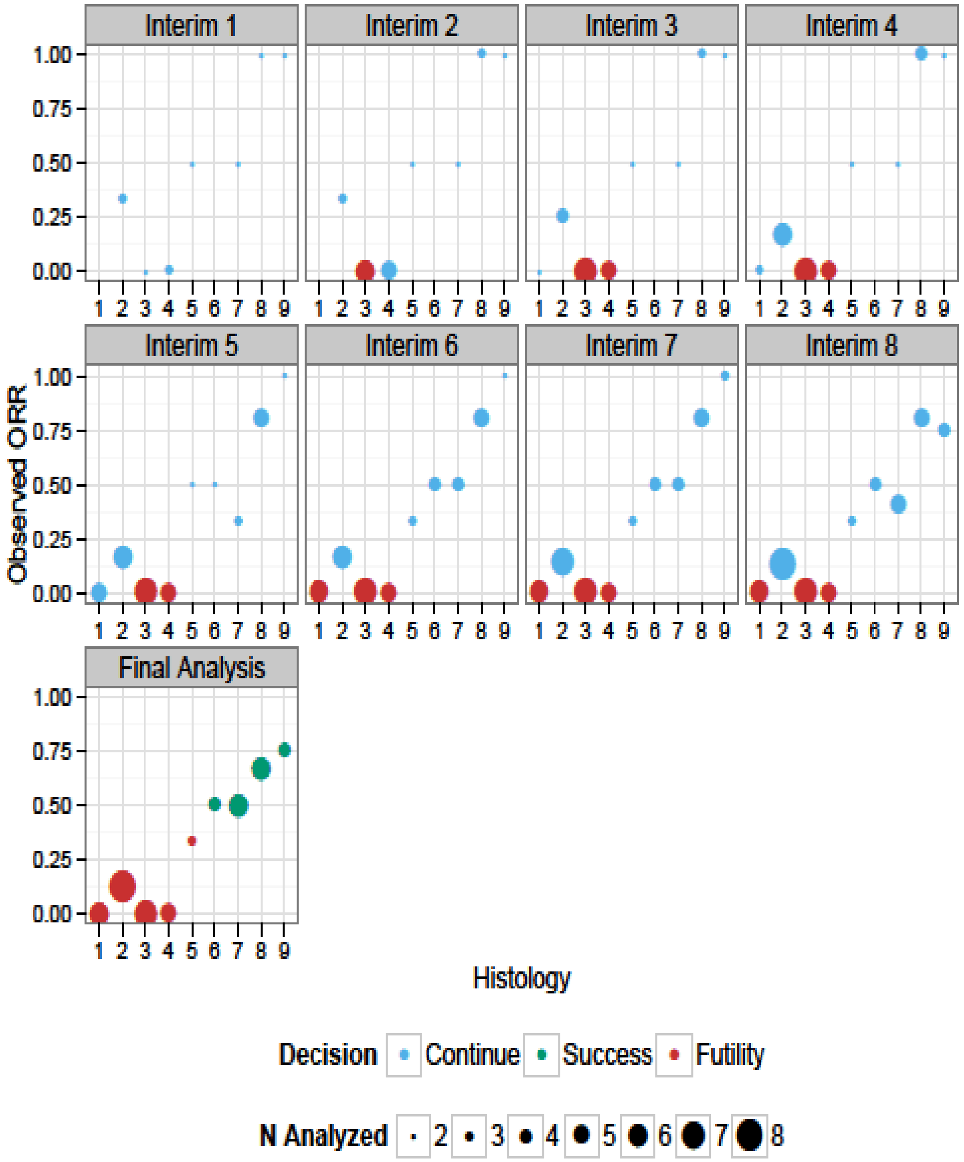

21.9 Appendix 9: Country-Specific Requirements

AUSTRIA:

In compliance with the Austrian Drug Law Amendment Act of 2004, the following regulatory requirements will be applied to all study centers in Austria:

Pregnancy: §30. The clinical testing of drugs may only be carried out or continued in women of childbearing potential, with the exception of cases as stated in §44, if non-pregnancy is confirmed prior to and at adequate intervals during the clinical trial.

Clinical Trials in Emergency Situations:

§43a. (1) If a clinical trial, by its very nature, can only be conducted in emergency situations in which the consent of the legal representative cannot be obtained within a reasonable length of time, then a clinical trial may be conducted on a person who is not in a position to give his or her consent if:

1. there is no informative basis that the subject has objected to or would object to the clinical trial,

2. such research is implicitly required for the validation of data that were obtained from clinical trials on persons able to give their informed consent or by means of other research methods and which can only be conducted in emergency situations,

3. the drug to be tested is intended to identify, heal, mitigate or prevent diseases in an emergency situation,

4. the application of the drug to be tested is indicated according to the latest findings in medical science in order to identify, heal or to mitigate a disease in emergency patients or to protect them from further diseases and the benefit of being included in the clinical trial outweighs the risk for the trial participant,

5. the conduct of the trial and the protocol have been approved by an ethics committee that has knowledge of the area of the disease in question and of the emergency situation concerned and the patient population involved or that has been advised on the clinical and ethical questions in the area of the disease in question and on the emergency situation concerned and the patient population involved, specifically for the conduct of clinical trials in emergency situations on persons who are unable to give their consent personally, and

6. in case of doubt the patient's interests, at all times, stand above public interests and the interest of science.

(2) The general public is to be informed about this circumstance in an appropriate manner at the clinical trial centre where a clinical trial is conducted on persons who are not in a position to give their consent.

(3) Should the patient recover and be in a position to give consent, he or she is to be informed immediately that a clinical trial has been or will be conducted in an emergency situation. He or she is to be informed as defined in §§38 and 39. The continuation of the clinical trial is only then allowed if informed consent has been given.

ITALY:

It is mandatory that any genetic and genomic research that is explorative in nature must be optional for the subject participating in the study.

21.10 0 Appendix 10: RECIST, version 1.1

MEASURABILITY OF TUMOR LESIONS AT BASELINE

Measurable lesion:

A non-nodal lesion that can be accurately measured in at least one dimension (longest dimension) of:

- ≥10 mm with magnetic resonance imaging (MRI) or computed tomography (CT) when the scan slice thickness is no greater than 5 mm. If the slice thickness is greater than 5 mm, the minimum size of a measurable lesion must be at least double the slice thickness (e.g., if the slice thickness is 10 mm, a measurable lesion must be ≥20 mm)

- ≥10 mm calliper/ruler measurement by clinical examination or medical photography

- ≥20 mm by chest x-ray

Additionally, lymph nodes can be considered pathologically enlarged and measurable if:

- ≥15 mm in the short axis when assessed by CT or MRI (slice thickness recommended to be no more than 5 mm). At baseline and follow-up, only the short axis will be measured

Non-measurable lesion:

All other lesions including lesions too small to be considered measurable (longest diameter <10 mm or pathological lymph nodes with ≥10 mm and <15 mm short axis) as well as truly non-measurable lesions, which include: leptomeningeal disease, ascites, pleural or pericardial effusions, inflammatory breast disease, lymphangitic involvement of the skin or lung, abdominal masses/abdominal organomegaly identified by physical exam that is not measurable by reproducible imaging techniques.

Measurable disease: The presence of at least one measurable lesion. Palpable lesions that are not measurable by radiologic or photographic evaluations may not be utilized as the only measurable lesion.

Non-Measurable only disease: The presence of only non-measurable lesions.

Specifications by methods of measurements:

- The same diagnostic method, including use of contrast when applicable, must be used throughout the study to evaluate a lesion.

- All measurements should be taken and recorded in millimeters (mm), using a ruler or calipers.

- Ultrasound is not a suitable modality of disease assessment. If new lesions are identified by ultrasound, confirmation by CT or MRI is required.

- Fluorodeoxyglucose-positron emission tomography (FDG-PET) is generally not suitable for ongoing assessments of disease. However, FDG-PET can be useful in confirming new sites of disease where a positive FDG-PET scans correlates with the new site of disease present on CT/MRI or when a baseline FDG-PET was previously negative for the site of the new lesion. FDG-PET may also be used in lieu of a standard bone scan providing coverage allows interrogation of all likely sites of bone disease and FDG-PET is performed at all assessments.

- If FDG-PET/CT is performed then the CT component can only be used for standard response assessments if performed to diagnostic quality, which includes the required anatomical coverage and prescribed use of contrast. The method of assessment should be noted as CT on the case report form.

Clinical Examination: Clinically detected lesions will only be considered measurable when they are superficial (e.g., skin nodules). In the case of skin lesions, documentation by color photography, including a ruler/calipers to measure the size of the lesion, is required.

CT and MRI: **<u>Contrast enhanced CT with 5 mm contiguous slices is recommended.</u>** Minimum size of a measurable baseline lesion should be twice the slice thickness, with a minimum lesion size of 10 mm when the slice thickness is 5 mm. MRI is acceptable, but when used, the technical specification of the scanning sequences should be optimised for the evaluation of the type and site of disease and lesions must be measured in the same anatomic plane by use of the same imaging examinations. Whenever possible the same scanner should be used.

X-ray: Should not be used for target lesion measurements owing to poor lesion definition. Lesions on chest X-ray may be considered measurable if they are clearly defined and surrounded by aerated lung; however, chest CT is preferred over chest X-ray.

EVALUATION OF TARGET LESIONS

Definitions for assessment of response for target lesion(s) are as follows:

- **Complete Response (CR):** Disappearance of all target lesions. Any pathological lymph nodes must be <10 mm in the short axis.

- **Partial Response (PR):** At least a 30% decrease in the sum of the diameters of target lesions, taking as a reference, the baseline sum of the diameters (e.g. percent change from baseline).

- **Stable Disease (SD):** Neither sufficient shrinkage to qualify for PR nor sufficient increase to qualify for PD.

- **Progressive Disease (PD):** At least a 20% increase in the sum of the diameters of target lesions, taking as a reference, the smallest sum of diameters recorded since the treatment started (e.g. percent change from nadir, where nadir is defined as the smallest sum of diameters recorded since treatment start). In addition, the sum must have an absolute increase from nadir of 5 mm.
- **Not Applicable (NA):** Target lesions not present at baseline.
- **Not Evaluable (NE):** Unable to classify using one of the five preceding definitions.

NOTE:

- If lymph nodes are documented as target lesions the short axis is added into thesum of the diameters (e.g. sum of diameters is the sum of the longest diameters for non- nodal lesions and the short axis for nodal lesions). When lymph nodes decrease to non-pathological size (short axis <10 mm) they should still have a measurement reported in order not to overstate progression.

- If at a given assessment time point all target lesions identified at baseline are <u>not</u> assessed, sum of the diameters <u>cannot</u> be calculated for purposes of assessing CR, PR, or SD, or for use as the nadir for future assessments. However, the sum of the diameters of the assessed lesions and the percent change from nadir should be calculated to ensure that progression has not been documented. If an assessment of PD cannot be made, the response assessment should be NE.

- All lesions (nodal and non-nodal) should have their measurements recorded even when very small (e.g., 2 mm). If lesions are present but too small to measure, 5 mm should be recorded and should contribute to the sum of the diameters, unless it is likely that the lesion has disappeared in which case 0 mm should be reported.

- If a lesion disappears and reappears at a subsequent time point it should continue to be measured. The response at the time when the lesion reappears will depend upon the status of the other lesions. For example, if the disease had reached a CR status then PD would be documented at the time of reappearance. However, if the response status was PR or SD, the diameter of the reappearing lesion should be added to the remaining

diameters and response determined based on percent change from baseline and percent change from nadir.

EVALUATION OF NON-TARGET LESIONS

Definitions for assessment of response for non-target lesions are as follows:

- **Complete Response (CR):** The disappearance of all non-target lesions. All lymph nodes identified as a site of disease at baseline must be non-pathological (e.g., <10 mm short axis).

- **Non-CR/Non-PD**: The persistence of 1 or more non-target lesion(s) or lymph nodes identified as a site of disease at baseline ≥10 mm short axis.
- **Progressive Disease (PD):** Unequivocal progression of existing non-targetlesions.
- **Not Applicable (NA):** Non-target lesions not present atbaseline.
- **Not Evaluable (NE):** Unable to classify using one of the four precedingdefinitions.

NOTE:

- In the presence of measurable disease, progression on the basis of solely non-target disease requires substantial worsening such that even in the presence of SD or PR in target disease, the overall tumour burden has increased sufficiently to merit discontinuation of therapy.
- In the presence of non-measurable only disease consideration should be given to whether or not the increase in overall disease burden is comparable in magnitude to the increase that would be required to declare PD for measurable disease.

- Sites of non-target lesions, which are not assessed at a particular time point based on the assessment schedule, should be excluded from the response determination (e.g. non-target response does not have to be "NE").

Frequency of tumor re-evaluation

Target and non-target lesions will be re-evaluated every 8 weeks.

Confirmation of Response

- To be assigned a status of PR or CR, a confirmatory disease assessment should be performed no less than 4 weeks (28 days) after the criteria for response are first met.

OVERALL RESPONSE CRITERIA

The table presents the overall response at an individual time point for all possible combinations of tumor responses in target and non-target lesions with or without the appearance of new lesions for subjects with measurable disease at baseline.

Evaluation of Overall Response for Subjects with Measurable Disease at Baseline

Target Lesions	Non-Target Lesions	New Lesions	Overall Response
CR	CR or NA	No	CR
CR	Non-CR/Non-PD or NE	No	PR
PR	Non-PD or NA or NE	No	PR
SD	Non-PD or NA or NE	No	SD
NE	Non-PD or NA or NE	No	NE
PD	Any	Yes or No	PD
Any	PD	Yes or No	PD
Any	Any	Yes	PD

Abbreviations: CR = complete response; NA = not applicable; NE = not evaluable; PD = progressive disease; PR = partial response; SD = stable disease

NOTE:

- Subjects with a global deterioration of health status requiring discontinuation of treatment without objective evidence of PD at that time should be classified as having "symptomatic deterioration." Objective response status is determined by evaluations of disease burden. Every effort should be made to document the objective progression even after discontinuation of treatment.

- In some circumstances, it may be difficult to distinguish residual disease from normal tissue. When the evaluation of CR depends on this determination, it is recommended that the residual lesion be investigated (fine needle aspirate/biopsy) to confirm the CR.

Reference:

Eisenhauer EA, Therasse P, Bogaerts, J, et al. New response evaluation criteria in solid tumors: revised RECIST guideline (version 1.1). *Eur J Cancer.* 2009 Jan;45(2):228-247.

21.11 Appendix 11: Response Criteria for NSGCT/NGGCT with Elevated Tumor Markers (AFP and β-HCG) at Baseline

Serum tumor markers alpha-fetoprotein (AFP) and beta-human chorionic gonadotropin (β-HCG) will be measured as indicated in the Time and Events Table

- **Marker-Negative Complete Response (CR-)** is defined as meeting the Response Evaluation Criteria in Solid Tumors (RECIST), v1.1 criteria for CR plus normalization of previously elevated tumor markers for a minimum of 4 weeks.
- **Marker-Positive Complete Response (CR+)** is defined as meeting the RECIST, v1.1 criteria for CR plus a tumor marker decrease for at least 4 weeks, but without complete normalization of previously elevated tumor markers.
- **Marker-Negative Partial Remission (PR-)** is defined as meeting the RECIST, v1.1 criteria for partial response (PR) plus normalization of previously elevated tumor markers for a minimum of 4 weeks.
- **Marker-Positive Partial Remission (PR+)** is defined as meeting the RECIST, v1.1 criteria for PR plus a tumor marker decrease for at least 4 weeks, but without complete normalization of previously elevated tumor markers.
- **Progressive Disease (PD)** is defined as a significant elevation (≥50%) in tumor markers after 4 weeks of therapy; treatment will be stopped despite of radiological response.

References:

Bokemeyer C, Oechsle K, Honecker F, et al. Combination chemotherapy with gemcitabine, oxaliplatin, and paclitaxel in patients with cisplatin-refractory or multiple relapsed germ-cell tumors: a study of the German Testicular Cancer Study Group. *Annals of Oncology.* 2008;19:448-453.

Einhorn LH, Brames MJ, Juliar B, Williams SD. Phase II Study of Paclitaxel plus Gemcitabine Salvage Chemotherapy for Germ Cell Tumors after Progression Following High-Dose Chemotherapy with Tandem Transplant. *J Clin Oncol.* 2007;25(5):513-516.

Kollmannsberger C, Beyer J, Liersch R, et al. Combination chemotherapy with gemcitabine plus oxaliplatin in patients with intensively pretreated or refrctory germ cell cancer: A Study of the German Testicular Cancer Study Group. *J Clin Oncol.*

2004;22:108-114.

21.12 Appendix 12: Response Criteria forHCL

Response status for hairy cell leukemia (HCL) will be assessed according to the following criteria adapted from the NCCN guideline - Consensus Resolution, and previous HCL studies.

Complete response (CR) with or without Minimal Residual Disease (±MRD):

- No evidence of leukemic cells by routine and H/E stains of the peripheral blood and bone marrow (BM).

- MRD: CR with HCL evident in blood by flow cytometry (FACS) or in BM biopsyby immunohistochemistry (IHC)

- No hepatomegaly, splenomegaly, or abnormal (< 2cm minimum length) lymphadenopathy by appropriate radiographic techniques.

- **NOTE:** Enlarged liver or spleen associated with a negative nuclear medicine study will not prevent consideration of CR for those subjects meeting all other criteria.

- Normal complete blood count (CBC) as exhibited by: absolute neutrophil count (ANC) $\geq 1.5 \times 10^9/L$, platelets $\geq 100 \times 10^9/L$, and hemoglobin ≥11 g/dL without transfusions or growth factors for at least 4 weeks.

After this 4-week period, BM biopsy and computed tomography (CT) scan must be performed to confirm CR. The BM biopsy and aspirate need not be performed at the beginning of this 4-week interval.

Complete response (CR): defined as the complete disappearance of all evidence of disease and requires all of the following:

- Normal CBC as exhibited by: ANC $\geq 1.5 \times 10^9/L$, platelets $\geq 100 \times 10^9/L$ and hemoglobin ≥11 g/dL

- Absence of hairy cells (by morphological examination) in the peripheral blood and in the BM

- Regression to normal of disease-related organomegaly

Confirmation of CR: At the confirmatory ≥6 month visit, a BM biopsy, hematology, immunoglobulins and flow cytometry will be reassessed.

Duration of CR: The duration of complete remission is calculated from the time of achievement of all criteria of CR to the time of relapse from CR.

Partial response (PR): requires all of the following for a period of at least 4 weeks:

- Normal peripheral blood counts (as in CR)
  - ANC $\geq 1.5 \times 10^9$/L or 50% improvement over baseline without growth factors for at least 4 weeks, **AND**
  - Platelets $\geq 100 \times 10^9$/L or 50% improvement over baseline, **AND**
  - Hemoglobin ≥11 g/dL or 50% improvement over baseline without transfusions or growth factors for at least 4 weeks.

    NOTE: For subjects who are transfusion-dependent at baseline, a hemoglobin of ≥9 g/dL without transfusions or growth factors for at least 4 weeks.

- circulating hairy cells ≤5% of lymphocytes
- ≥50% reduction of BM infiltration by hairy cells
- ≥50% reduction of palpable disease-related organomegaly.

Subject must have the following (if abnormal prior to treatment):

- ≥50% decrease or normalization (<5000/mm^3) in peripheral blood lymphocyte count from the pre-treatment baseline value
- ≥50% reduction in lymphadenopathy, based on sum of products of perpendicular diameters, or resolution to size consistent with CR
- ≥50% reduction in abnormal hepatosplenomegaly by imaging, or resolution to size consistent with CR

Minor response (MR): requires all of the following:

- ≥50% reduction of circulating hairy cells
- Improvement of one or more of the peripheral blood counts (ANC, platelets or hemoglobin).

Relapse: defined as the reappearance of leukemia/lymphoma in a subject who previously achieved a CR or documentation of PD in a subject who previously achieved a PR

Stable Disease (SD): defined as not meeting the criteria for CR, PR, MR or PD

Disease progression (PD): defined as the occurrence of at least one of the following compared to pre-treatment:

- ≥50% increase in the sum of the products of the greatest perpendicular dimensions of at least two lymph nodes on two consecutive examinations at least 2 weeks apart (at least one node must be ≥2 cm in minimum length) or appearance of new palpable lymph nodes

- ≥50% increase in the absolute number of circulating lymphocytes, on two consecutive examinations showing abnormal lymphocytosis at least 2 weeks apart

- ≥25% decrease in hemoglobin (must be <10g/dL), platelets (must be <100,000/uL), or ANC (must be <1500/uL) unless these are judged to be effects of treatment.

References:

Catovsky D, Golomb HM, Golde DW. Consensus Resolution: Proposed Criteria for Evaluation of Response to Treatment in Hairy Cell Leukemia. *Leukemia*. 1987;4(1):405-406.

Kreitman RJ, Fitzgerald DJ, Pastan I. Approach to the patient after relapse of hairy cell leukemia. *Leuk Lymphoma.* 2009a;50(suppl 1):32-37.

Kreitman RJ, Tallman MS, Robak T, et al. Phase I trial of anti-CD22 recombinant immunotoxin moxetumomab pasudotox (CAT-8015 or HA22) in patients with hairy cell leukemia. *J Clin Oncol*. 2012;30:1822-1828.

Kreitman, R.J., Stetler-Stevenson, M., Margulies, I., et al. Phase II trial of recombinant immunotoxin RFB4(dsFv)-PE38 (BL22) in patients with hairy cell leukaemia. *J Clin Oncol.* 2009b;27:2983–2990.

Nieva J, Bethel K, Saven A. Phase 2 study of rituximab in the treatment of cladribine-failed patients with hairy cell leukemia. *Blood* 2003;102:810-813.

Saven A, Burian C, Koziol JA, Piro LD. Long-term follow-up of patients with hairy cell leukemia after cladribine treatment. *Blood.* 1998;92(6):1918-1926.

21.13 Appendix 13: RANO Response Criteria: Response Assessment Criteria for WHO Grade 1 or 2Glioma

Measureable disease is defined as bidimensionally contrast non-enhancing or enhancing lesions with clearly defined margins by magnetic resonance imaging (MRI) scan, with two perpendicular diameters of at least 10 mm, visible on 2 or more axial slices that are preferably, at most, 5 mm apart with 0 mm skip.

Non-measurable disease is defined as either unidimensionally measurable lesions, masses with margins not clearly defined, or lesions with maximal perpendicular diameters less than 10 mm.

All measurable and non-measurable lesions must be assessed using the same techniques as at baseline.

Complete response (CR): Requires all the following criteria compared with thebaseline scan:

- Complete disappearance of the lesion on T2 or FLAIR (fluid attenuated inversion recovery) imaging (if enhancement had been present, it must have resolved completely);
- no new lesions, no new T2 or FLAIR abnormalities apart from those consistent with radiation effects, and no new or increased enhancement;
- subject must be off corticosteroids or only on physiological replacement doses, **and**
- subject should be stable or improved clinically

Partial response (PR): Requires all of the following criteria compared with the baseline scan:

- greater than or equal to 50% decrease in the product of perpendicular diameters of the lesion on T2 or FLAIR imaging sustained for at least 4 weeks compared with baseline;
- no new lesions, no new T2 or FLAIR abnormalities apart from those consistent with radiation effects, and no new or increased enhancement;
- subject should be on a corticosteroid dose that should not be greater than the dose at time of baseline scan, and should be stable or improved clinically

Minor response (MR): Requires the following criteria compared with the baseline scan:

- a decrease of the area of enhancing or non-enhancing lesion on T2 or FLAIR MR imaging between 25% and 50% compared with baseline
- no new lesions, no new T2 or FLAIR abnormalities apart from those consistent with radiation effect, and no new or increased enhancement; **and**
- subject should be on a corticosteroid dose that should not be greater than the dose at time of baseline scan, and should be stable or improved clinically

Stable disease (SD): Stable disease (SD) is present if the changes do not qualify for CR, PR, MR or progression and requires:

- stable area of enhancing or non-enhancing abnormalities on T2 or FLAIR imaging
- no new lesions, no new T2 or FLAIR abnormalities apart from those consistent with radiation effect, and no new or increased enhancement
- subject should be on a corticosteroid dose that should not be greater than the dose at time of baseline scan, and should be stable or improved clinically

Disease progression (PD): Disease progression (PD) is defined by any of the following:

- development of new lesions or increase of enhancement (radiological evidence of malignant transformation)
- a 25% increase in the sum of the products of perpendicular diameters of the T2 or FLAIR enhancing or non-enhancing lesion on stable or increasing doses of corticosteroids compared with baseline scan or best response after initiation of therapy, not attributable to radiation effect or to comorbid events
- definite clinical deterioration not attributable to other causes apart from the tumour, or decrease in corticosteroid dose;
- or failure to return for evaluation because of death or deteriorating condition, unless caused by documented non-related disorders

References:

van den Bent MJ, Wefel JS, Schiff D, et al. Response assessment in neuro-oncology (a report of the RANO group): assessment of outcome in trials of diffuse low grade gliomas. The Lancet. 2011;12:583-593.

21.14 Appendix 14: RANO Response Criteria: Updated Response Assessment Criteria for WHO Grade 3 and 4 Glioma

Measureable disease is defined as bidimensionally contrast-enhancing or enhancing lesions with clearly defined margins by magnetic resonance imaging (MRI) scan, with two perpendicular diameters of at least 10 mm, visible on 2 or more axial slices that are preferably, at most, 5 mm apart with 0 mm skip.

Non-measurable disease is defined as either unidimensionally measurable lesions, masses with margins not clearly defined, or lesions with maximal perpendicular diameters less than 10 mm.

All measurable and non-measurable lesions must be assessed using the same techniques as at baseline.

Complete response (CR): Requires all of the following:

- complete disappearance of all enhancing measurable and non-measurable disease sustained for at least 4 weeks
- no new lesions
- stable or improved non-enhancing (T2/FLAIR [fluid attenuated inversion recovery]) lesions
- subjects must be off corticosteroids (or on physiologic replacement doses only)
- stable or improved clinically.

Note: Subjects with non-measurable disease only cannot have a CR; the best response possible is stable disease (SD).

Partial response (PR): Requires all of the following:

- ≥50% decrease compared with baseline, in the sum of products of perpendicular diameters of all measurable enhancing lesions sustained for at least 4 weeks
- no progression of non-measurable disease; no new lesions
- stable or improved non-enhancing (T2/FLAIR) lesions on same or lower dose of corticosteroids compared with baseline scan

- corticosteroid dose at the time of the scan evaluation should be no greater than the dose at time of baseline scan

- stable or improved clinically.

Note: Subjects with non-measurable disease only cannot have a PR; the best response possible is SD.

Stable disease (SD): Requires all of the following:

- does not qualify for CR, PR, or progression

- stable non-enhancing (T2/FLAIR) lesions on same or lower dose of corticosteroids compared with baseline scan.

In the event that the corticosteroid dose was increased for new symptoms and signs without confirmation of disease progression (PD) on neuro-imaging, and subsequent follow-up imaging shows that this increase in corticosteroids was required because of PD, the last scan considered to show SD will be the scan obtained when the corticosteroid dose was equivalent to the baseline dose.

NOTE: Stable doses of corticosteroids include subjects not on corticosteroids.

Disease progression (PD): Disease progression (PD) is defined by any of the following:

- ≥25% increase in sum of the products of perpendicular diameters of enhancing lesions compared with the smallest tumor measurement obtained either at baseline (if no decrease) or best response, on stable or increasing doses ofcorticosteroids

- significant increase in T2/FLAIR non-enhancing lesion on stable or increasing doses of corticosteroids compared with baseline scan or best response after initiation of therapy not caused by co-morbid events (e.g., radiation therapy, demyelination, ischemic injury, infection, seizures, post-operative changes, or other treatment effects)

- any new lesion; clear clinical deterioration not attributable to other causes apart from the tumor (e.g., seizures, medication adverse effects, complications of therapy, cerebrovascular events, infection) or changes in corticosteroid dose

- failure to return for evaluation as a result of death or deteriorating condition

- clear progression of non-measurable disease.

Summary of RANO Response Criteria

Criterion	CR	PR	SD	PD
T1 gadolinium-enhancing disease	None	≥50% decrease	<50% decrease but <25% increase	≥25% increase[1]
T2/FLAIR	Stable or decrease	Stable or decreasing	Stable or decrease	Increase
New Lesion	None	None	None	Present
Corticosteroids	None	Stable or decreasing dose	Stable or Decreasing dose	NA[2]
Clinical Status	Stable or increase	Stable or increase	Stable or increase	Decrease
Requirement for Response	All	All	All	Any

Abbreviations: CR = complete response; FLAIR = fluid-attenuated inversion recovery; NA = not applicable; PD = progressive disease; PR = partial response; RANO = Response Assessment in Neuro-Oncology: SD = stable disease

Progression occurs when this criterion is present.
Increase in corticosteroids alone will not be taken into account in determining progression in the absence of persistent clinical deterioration.

References:

Wen PY. Updated Response Assessment Criteria for High-Grade Gliomas: Response Assessment in Neuro-Oncology Working Group. *J Clin Oncol*. 2010;28(11):1963-1972.

21.15 Appendix 15: Consensus Recommendations for the Uniform Reporting of Clinical Trials

Report of the International Myeloma Workshop Consensus Panel
International Myeloma Workshop Consensus (IMWC) Response Criteria:

Stringent Complete Response (sCR):

Complete response (CR) as defined below plus:

- normal free light chain (FLC) ratio and
- absence of clonal cells in bone marrow (BM) by immunohistochemistry (IHC) or 2 to 4 color flow cytometry

Complete Response (CR):

- Negative serum and urine immunofixation, and
- Disappearance of any soft tissue plasmacytomas, and
- ≤5% plasma cells in BM

Very Good Partial Response (VGPR):

- Serum and urine M-component detectable by immunofixation but not on electrophoresis

OR

- 90% or greater reduction in serum M-component plus urine M-component <100 mg per 24 hrs

Partial Response (PR):

- ≥50% reduction of serum M-protein and reduction in 24-hr urinary M-protein by ≥90% or to <200 mg per 24 hrs

AND

- If the serum and urine M-protein are not measurable, a ≥50% decrease in the difference between involved and uninvolved FLC levels is required in place of the M-protein criteria. If serum and urine M-protein are not measurable, and serum FLC assay is also not measurable, ≥50% reduction in BM plasma cells is required in place of M-protein, provided baseline BM plasma cell percentage was ≥30%, and in the size of the soft tissue plasmacytomas is also required.

Minimal Response (MR):

- ≥25% but ≤49% reduction of serum M-protein and reduction in 24-hr urinary M-protein by 50% to 89%

AND

- If present at baseline, 25% to 49% reduction in the size of soft tissue plasmacytomas is also required.

- No increase in size or number of lytic bone lesions (development of compression fracture does not exclude response).

Stable Disease (SD):

- Not meeting criteria for CR, VGPR, PR, MR or PD

Progressive Disease (PD):

Requires an increase of ≥25% from lowest response value in any one or more of the following:

- serum M-component (absolute increase must be ≥0.5 g/dL), or

- urine M-component (absolute increase must be ≥200 mg/24 hr), or

- the difference between involved and uninvolved FLC levels (absolute increase must be >10 mg/dL): only for subjects without measurable serum and urine M-protein levels, or

- bone marrow plasma cell percentage (the absolute % must be ≥10%) – only for subjects without measurable serum and urine M-protein levels and without measurable disease by FLC level

- definite development of new bone lesions or soft tissue plasmacytomas or definite increase in the size of existing bone lesions or soft tissue plasmacytomas

- development of hypercalcemia (corrected calcium >11.5 mg/dL or 2.65 mmol/L) that can be attributed solely to the plasma cell proliferative disorder

a. All response categories (CR, sCR, VGPR, PR, MR and PD) require 2 consecutive assessments made at any time before the institution of any new therapy; CR, sCR, VGPR, PR, MR, and SD categories also require no known evidence of progressive or new bone lesions if radiographic studies were performed. VGPR and CR categories require serum and urine studies regardless of whether disease at baseline was measurable on serum, urine, both, or neither. Radiographic studies are not required to satisfy these response requirements. Bone marrow assessments need not be confirmed. For PD, serum M- component increases of more than or equal to 1 g/dL are sufficient to define relapse if starting M-component is ≥5 g/dL.

b. Clarifications to IMWG criteria for coding CR and VGPR in subjects in whom the only measurable disease is by serum FLC levels: CR in such subjects indicates a normal FLC ratio of 0.26 to 1.65 in addition to CR criteria listed above. VGPR in such subjects requires a >90% decrease in the difference between involved and uninvolved FLC levels.

c. Clarifications to IMWG criteria for coding PD: Bone marrow criteria for PD are to be used only in subjects without measurable disease by M protein and by FLC levels; “25% increase” refers to M protein, FLC, and BM results, and does not refer to bone lesions, soft tissue plasmacytomas, or hypercalcemia and the “lowest response value” does not need to be a confirmed value.

References:

Durie BGM, Harousseau J-L, Miguel JS, et. al. International uniform response criteria for multiple myeloma. *Leukemia.* 2006:20:1467-73.

Rajkumar SV, Harousseau JL, Durie B, et al. Consensus recommendations for the uniform reporting of clinical trials: Report of the International Myeloma Workshop Consensus Panel 1. *Blood*. 2011;117:4691-4695.

21.16 Appendix 16: Imaging Guidelines for WHO Grade I or II Glioma

Basic MRI protocol:

- Axial FLAIR (canthomeatal alignment): 3 to 5 mm sections, 1 mm interslice gaps, slice registration preserved as much as possible between sequential studies
- Axial T2: 5 mm sections, 1 mm interslice gap
- Coronal T1: 5 mm sections, 1 mm interslice gap
- Post-gadolinium chelate (contrast agent per local clinical practice): cornoal T1, axial T1

Alternatively pre-gadolinium and post-gadolinium volumetric T1 may replace axial and coronal T1-weighted sequences.

Supplementary imaging methods:

- Two-dimensional or 3-dimensional spectroscopic imaging (MR spectroscopy)
- Perfusion imaging (DSC-MRI)
- Diffusion-weighted imaging (-B=0, B=1000)

PET imaging:

For ^{11}C-MET and ^{18}F-FET PET:

- Subject must fast for 4 hrs prior to imaging
- Images must be obtained in a supine position
- 10 min transmission scan must be done prior to tracer application
- C-MET or F-FET administered intravenously as a bolus injection of 740 MBq (20 mCi; MET) or 370 MBq (10 mCi; FET)

- Accumulation of tracer will be recorded over 60 min in 47 transaxial slices of entire brain
- Spatial resolution of C-MET PET and F-FET PET: 6 mm or better in all dimensions
- SUV, T/B ratios and uptake kinetics: calculated in tumor and corresponding mirror regions

Abbreviations: DSC, dynamic susceptibility contrast; FET, ^{18}F-fluoroethyltyrosine; FLAIR, fluid attenuation inversion recovery; MET, ^{11}C-methionine; PET, positron emission tomography; SUV, standard uptake value; T/B, tumor background ratio

References:

van den Bent MJ, Wefel JS, Schiff D, et al. Response assessment in neuro- oncology (a report of the RANO group): assessment of outcome in trials of diffuse low grade gliomas. The Lancet. 2011;12:583-593.

21.17 Appendix 17: Volumes of Collected Blood Samples

Assuming a subject remains on treatment for a period of 6 months, the total blood volume collected is estimated to be 306 mL (excluding any unpredictable events that would require additional blood samples to be obtained and the additional 25 mL for the Hairy Cell Leukemia Ex Vivo sub-study).

Reason for Blood Sample	Screening	Day 1	Day 15	Week 4	Week 8	Week 12	Week 16	Trmt Disc
PK Analysis	N/A	N/A	N/A	4mL	2mL	2mL	N/A	2mL
PGx Analysis (optional)	N/A	6mL	N/A	N/A	N/A	N/A	N/A	N/A
BRAF V600E<br><br>(HCL and MM cohorts only)	3mL	N/A	N/A	N/A	N/A	N/A	N/A	N/A
[redacted] Biomarker Analyses	21mL	N/A	18mL	N/A	N/A	N/A	N/A	18mL
Clinical Laboratory Assessments	17mL	17mL	N/A	17mL	17mL	17mL	17mL	17mL
**TOTAL Volume**	**38mL**	**23mL**	**18mL**	**21mL**	**19mL**	**19mL**	**17mL**	**37mL**
Ex Vivo Sub-study<br><br>(HCL cohort only)	25mL	N/A	N/A	N/A	N/A	N/A	N/A	25mL
**HCL TOTAL Volume**	**63mL**	**23mL**	**18mL**	**21mL**	**19mL**	**19mL**	**17mL**	**62mL**

Abbreviations: HCL = Hairy Cell Leukemia; MM =Multiple Myeloma[redacted]; PGx = Pharmacogenetics; [redacted]; Trmt Disc = treatment discontinuation

21.18 Appendix 18: Liver Safety Drug Restart or Rechallenge Guidelines

If subject meets liver chemistry stopping criteria do not restart/rechallenge subject with study treatment unless:

- Novartis Medical Governance approval **is granted** (as described below),
- Independent Ethics Committee (IEC) and/or institutional review board (IRB) approval is obtained, if required, and
- Separate consent for treatment restart/rechallenge is signed by the subject

If Novartis Medical Governance approval to restart/rechallenge subject with study treatment

is not granted, then subject must permanently discontinue study treatment and may continue in the study for protocol-specified follow-up assessments.

1. Rechallenge Following Liver Stopping Events that are Possibly Related to Study Treatment

Following drug-induced liver injury (DILI), **drug rechallenge is associated with a 13% mortality across all drugs in prospective studies** [Andrade, 2009] Clinical outcomes vary by drug, with nearly 50% fatality with halothane readministered within 1 month of initial injury. However, some drugs seldom result in recurrent liver injury or fatality.

Risk factors for a fatal drug rechallenge outcome include:

- hypersensitivity[1] with initial liver injury (e.g. fever, rash, eosinophilia)
- jaundice or bilirubin >2xULN with initial liver injury (direct bilirubin >35% of total)
- subject currently exhibits severe liver injury defined by: ALT≥3xULN, bilirubin ≥2xULN (direct bilirubin >35% of total), or INR≥1.5
- serious adverse event or fatality has earlier been observed with drug rechallenges [Papay, 2009; Hunt, 2010]

- evidence of drug-related preclinical liability (e.g. reactive metabolites; mitochondrial impairment) [Hunt, 2010]

Rechallenge refers to resuming study treatment following DILI. Because of the risks associated with rechallenge after DILI this should only be considered for a subject for whom there is compelling evidence of benefit from a critical or life-saving medicine, there is no alternative approved medicine available, and a benefit:risk assessment of rechallenge is considered to be favourable.

Approval by the sponsor for rechallenge with study treatment can be considered where:

- Investigator requests consideration of rechallenge with study treatment for a subject who is receiving compelling benefit with study treatment that exceeds risk, and no effective alternative therapy is available.

- EC or IRB approval for rechallenge with study treatment must be obtained, as required.

- If the rechallenge is approved by Novartis Medical Governance in writing, the subject must be provided with a clear description of the possible benefits and risks of study treatment administration, including the possibility of recurrent, more severe liver injury or death.

- The subject must also provide signed informed consent specifically for the rechallenge with study treatment. Documentation of informed consent must be recorded in the study chart.

- Study treatment must be administered at the dose specified by the sponsor.

- Subjects approved by Novartis Medical Governance for rechallenge with study treatment must return to the clinic twice a week for liver chemistry tests until stable liver chemistries have been demonstrated and then standard laboratory monitoring may resume as per protocol.

- If after study treatment rechallenge, subject meets protocol-defined liver chemistry stopping criteria, study treatment should be permanently discontinued.

- Medical Lead, and the IEC or IRB as required, must be informed of the subject's outcome following study treatment rechallenge.

- The sponsor to be notified of any AEs, as per Section 11.

2. Restart Following Transient Resolving Liver Stopping Events NOT Related to Study Treatment

Restart refers to resuming study treatment following liver stopping events in which there is a clear underlying cause (other than DILI) of the liver event (e.g. biliary obstruction, pancreatic events, hypotension, acute viral hepatitis). Furthermore, there should be no evidence of alcoholic hepatitis or hypersensitivity, and the study treatment should not be associated with HLA markers of liver injury.

Approval by the sponsor for study treatment restart can be considered where:

- Investigator requests consideration for study treatment restart if liver chemistries have a clear underlying cause (e.g., biliary obstruction, hypotension and liver chemistries have improved to normal or are within 1.5x baseline and ALT <3xULN).
- Restart risk factors (e.g. fever, rash, eosinophilia, or hypersensitivity, alcoholic hepatitis, possible study treatment-induced liver injury or study treatment has an HLA genetic marker associated with liver injury (e.g. lapatinib, abacavir, amoxicillin/clavulanate) are reviewed and excluded.
- IEC or IRB approval of study treatment restart must be obtained, as required.
- If restart of study treatment is approved by Novartis Medical Governance in writing, the subject must be provided with a clear description of the possible benefits and risks of study treatment administration, including the possibility of recurrent, more severe liver injury or death.
- The subject must also provide signed informed consent specifically for the study treatment restart. Documentation of informed consent must be recorded in the study chart.
- Study treatment must be administered at the dose specified by the sponsor.
- Subjects approved by Novartis Medical Governance for restarting study treatment must return to the clinic once a week for liver chemistry tests until stable liver chemistries have been demonstrated and then laboratory monitoring may resume as per protocol.
- If after study treatment re-start, subject meets protocol-defined liver chemistry stopping criteria, follow usual stopping criteria instructions.
- Medical Lead, and the IEC or IRB as required, must be informed of the subject’s outcome following study treatment restart.
- The sponsor to be notified of any AEs, as per Section 11.

References:

Andrade RJ, Robles M, Lucena MI. Rechallenge in drug-induced liver injury: the attractive hazard. Expert Opin Drug Saf. 2009;8:709-714.

Hunt, CM. Mitochondrial and immunoallergic injury increase risk of positive drug rechallenge after drug-induced liver injury: A systematic review. *Hepatol.* 2010;52:2216-2222.

Papay JI, Clines D, Rafi R, et al. Drug-induced liver injury following positive drug rechallenge. *Regul Tox Pharm*. 2009;54:84-90

21.19 Appendix 19: Protocol Amendment Changes

AMENDMENT 06

Where the Amendment Applies

Amendment 06 applies to all sites that are or will be participating in this study.

Summary of Amendment Changes

The changes made with Amendment No. 6 were to update contact details for Medical Monitor, to implement reference to new Investigator's Brochure (v7), to update list of abbreviation, to update risk assessment for dabrafenib + trametinib combination thereapy, to change in time window for radiotherapy in Glioma Grade 1-4 cohort only, to clarify assessment timelines and correctdiscrepancies between T&E Table and text, to implementing "Tissue Sample for Histology Confirmation" for ATC and WHO Grade 1- 4 Glioma cohort only, to clarifyDisease Assments Methods for Glioma Grade 1-4 chohorts, to correct timelines for re-escalation of toxicity induced dose reduction, to clarify instructios for prohibited medications and non-drug therapies, to add additional analysis populations that are planned for the interim analyses, to add an expansion cohort to allow enrollment of patients if a given cohort{ is stopped early for efficacy, to add the language to refer to the RAP for the response confirmation criteria for each histological cohort, to update Table 31 to include Marker-Negative CR, Marker-Positive CR, Marker-Negative PR and Marker-Positive PR as response categories for NSGCT/NGGCT cohort, to clarify the definition for Duration of Response to include all response categories as defined in Table 31 for NSGCT/NGGCT, WHO Grades 1 and 2Gliomas, MM and HCL cohorts, and to make other administrative or formatting revisions that did not affect the conduct of the study.

List of Specific Changes

Section Sponsor/Medical Monitor Inforation

Page: REVISED TEXT

Role	Name	Day Time Phone Number	After-hours Phone/Cell/ Pager Number	Fax Number	Novartis Address
[redacted]	[redacted] MD, PhD	[redacted]	[redacted]	[redacted]	Novartis Pharmaceuticals Corporation One Health Plaza East Hanover, NJ 07936-1080 USA
[redacted]	[redacted]	[redacted]	[redacted]	[redacted]	Novartis Pharmaceuticals

[redacted] [redacted] [redacted] [redacted] [redacted]
MD
Corporation One Health Plaza East
Hanover, NJ 07936-1080
USAEmail:
[redacted]

List of abbreviation

REVISED TEXT

SPEP Serum Protein Electrophoresis

UPEP Urine Proteine Electrophoresis

Section 1.2 Dabrafenib (GSK2118436): 3rd paragraph

REVISED TEXT

Refer to the dabrafenib monotherapy **and dabrafenib+trametinib combination therapy** Investigator's Brochure (IB) [GlaxoSmithKline Document Number CM2010/00010/05] for a complete summary of currently available preclinical, safety, [redacted] and clinical data.

Section 1.3 Trametinib (GSK1120212): 3rd paragraph

REVISED TEXT

Trametinib in combination with dabrafenib has been approved in the US**, EU**, Chile, and Australia for the treatment of patients with unresectable or metastatic melanoma with BRAF V600E or V600K mutation.

Refer to the **dabrafenib monotherapy and dabrafenib+trametinib** ~~f~~or a complete summary of the currently available chemistry, nonclinical, and clinical data to support the use of the combination in subjects with BRAF V600 mutation-positive solid tumors [GlaxoSmithKline Document Number CM2010/00010/05].

Section 1.4 Risk Assessment for Dabrafenib + Trametinib Combination Therapy

REVISED TEXT

The assessment of the risk of dabrafenib + trametinib combination therapy, and suggestions for management of risk, is based on non-clinical data and clinical data from the ongoing and completed dabrafenib and trametinib monotherapy and combination studies in adults. In the event that any adverse events (AEs) are reported or observed, supportive treatment will be provided according to standard medical practice. Subjects will be withdrawn from the study if a clinically significant toxicity is reported or observed.

The safety profile for dabrafenib + trametinib combination therapy has been established and is well-characterized in subjects with unresectable/metastatic melanoma, with consistent results across two randomized Phase III study. The safety profile of the combination of dabrafenib and trametinib generally reflect AEs of the individual agents; some notable exceptions are described below.

Cutaneous Squamous cell carcinoma (cuSCC): In vitro experiments have demonstrated a paradoxical activation of MAPK signalling in kerationcytes and potentially other cells harboring a wild-type BRAF kinase but a mutated RAS kinase upon exposure to a BRAFi. This paradoxical MAPK pathway activation is potentially associated with a higher risk for the development of cuSCC induction. Cases of cuSCC (which include those classified as keratoacanthoma or mixed keratoacanthoma subtypes) have been reported in subjects treated with dabrafenib + trametinib.

Skin examination should be performed prior to initiation of dabrafenib or dabrafenib + trametinib treatment and while receiving therapy. Dermatologic examinations should be performed following discontinuation of dabrafenib monotherapy or in combination with trametinib, or until initiation of another anti-neoplastic therapy (see Section 7.1, Time and Events Table for schedule of assessments). Cases of cuSCC should be managed by dermatological excision; dabrafenib **and trametinib** treatment should be continued without any dose adjustment**. A brief dermatological exam should be performed every 4 weeks (±7 days) for the first 6 months after discontinuation of study treatments to monitor for new cutaneous malignancies.**

Pyrexia has been reported in clinical trials with dabrafenib monotherapy and in combination with trametinib. In dabrafenib monotherapy studies, pyrexia was one of the most frequently occurring AEs in up to 27% of subjects across all dabrafenib studies.

Most of the pyrexia events (64%) were considered treatment-related. The incidence and severity of pyrexia are increased when dabrafenib is used in combination with trametinib. In subjects who received the combination dose of dabrafenib 150 mg twice daily (BID)and trametinib 2 mg once daily and developed pyrexia, approximately half of the first occurrences of pyrexia happened within the first months of therapy. About **one-third** of the subjects who experienced pyrexia had **3 or more** events. Pyrexia may be accompanied by severe chills/rigors, dehydration and hypotension which in some cases can lead to acute renal insufficiency.

Subjects should be instructed on the importance of immediately reporting febrile episodes. Therapy with dabrafenib should be interrupted if the patient’s temperature is ≥38.5ºC or 101.3°F but **trametinib should be continued at the same dose.** In the event of a fever, the subject should be instructed to take anti-pyretics (i.e., ibuprofen or acetaminophen/paracetamol) as appropriate to control fever. The use of oral corticosteroids should be considered in those instances in which anti-pyretics are insufficient. Monitor serum creatinine and other evidence of renal function during and following severe events of pyrexia. (See Section 8.2.3 for additional details.)

Decreased left ventricular ejection fraction (LVEF): Left ventricular dysfunction has been reported with trametinib as well as with other MEKi in clinical development. **In the 2 Phase III studies decreased ejection fraction occurred in 7% of subjects receiving combination therapy and the majority of the LVEF decreases that met interruption criteria were asymptomatic and resolved. Most subjects who were re-challenged were able to continue on treatment without further dose modification.** Subjects enrolled in studies with dabrafenib in combination with trametinib undergo regular cardiac assessments with echocardiograms (ECHOs). Guidelines for clinical management of treatment-related LVEF decreases have been provided in Section 8.1.1.2.

Haemorrhage: Haemorrhagic events, including major haemorrhagic events defined as symptomatic bleeding in a critical area or organ, and fatal intracranial haemorrhages, have occurred in patients taking dabrafenib in combination with trametinib. The majority of bleeding events were mild. Three of 209 subjects (1%) receiving trametinib in combination with dabrafenib in a phase III trial had fatal intracranial haemorrhagic events. The management of hemorrhagic events may require treatment interruption, dose reduction, or treatment discontinuation….

Hyperglycemia: Hyperglycemia requiring an increase in the dose of, or initiation of, insulin or oral therapy can occur with dabrafenib **or dabrafenib in combination with trametinib**. Monitor serum glucose levels as clinically appropriate during treatment with dabrafenib in subjects with pre-existing diabetes or hyperglycemia. Advise subjects to report symptoms of severe hyperglycemia such as excessive thirst or any increase in the volume or frequency of urination.

Pancreatitis: Subjects **receiving dabrafenib or dabrafenib in combination with trametinib** have reported AEs of acute pancreatitis or pancreatitis. In **some** cases, serious adverse events (SAEs) of Grade 4 pancreatitis were reported as treatment-related by the investigator. The time to onset of pancreatitis ranged from study Day 21 to 292 (median 138 days) **with dabrafenib monotherapy, and from Study Day 24 to 312 (median 50 days) with combination therapy**. Discontinuation of study treatment dueto pancreatitis was not deemed necessary in any of the cases by the investigators. For AEs of abdominal pain or suspected pancreatitis, amylase and lipase laboratory samples should be monitored locally. Subjects should be closely monitored when re-starting dabrafenib after an episode of pancreatitis.

Use during Pregnancy and Lactation

Fertility: There is no information on the effect of dabrafenib or trametinib on human fertility. Animal studies with dabrafenib monotherapy or in combination with trametinib have shown reproductive toxicity which may impair fertility in humans. In dogs, rats and mice given dabrafenib and in dogs given dabrafenib in combination with trametinib, male reproductive effects, including testicular degeneration/depletion with secondary epididymal oligospermia, have occurred without clear evidence of reversibility. Male subjects should be informed of the potential risk for impaired spermatogenesis, which may be irreversible. **Trametinib may impair fertility in humans.**

Pregnancy: No adequate and well-controlled studies of dabrafenib or trametinib in pregnant women have been conducted. Animal studies with trametinib and dabrafenib have shown embryofetal developmental toxicities, including teratogenic effects.

Dabrafenib or trametinib should not be administered to pregnant women or nursing mothers. Women of childbearing potential should use effective methods of contraception during therapy and for 4 months following discontinuation of trametinib **when taken** in combination with dabrafenib, or for 4 weeks following discontinuation of dabrafenib.

Dabrafenib may decrease the efficacy of hormonal contraceptives; therefore, an alternative method of contraception such as barrier methods should be used. If dabrafenib or trametinib is used during pregnancy, or if the subject becomes pregnant while taking dabrafenib or trametinib, the subject should be informed of the potential hazard to the fetus.

Section 1.5.6 Cohort 6: Non-Seminomatous/Non-Germinomatous Germ Cell Tumors (NSGCT/NGGCT): 2nd paragraph

REVISED TEXT

Non-seminomatous GCTs (NSGCTs) tend to be more clinically aggressive and grow quicker than the seminoma-type of GCT. If untreated, NSGCTs can spread to the lungs, liver, lymph nodes, or bones. Common risk factors include being male, under the age of 20 and having Klinefelter syndrome [Nichols, 1987]. Chest pain and difficulty breathing are early signs of NSGCT. Other symptoms include: cough, fever, headache, **and change** in bowel habits, fatigue, difficulty walking and visual problems (difficulty seeing or moving eyes). Testicular GCTs present with a painless, solid testicular mass and often discomfort or swelling; persistent conditions warrant further evaluation.

Section 2.1 Rationale for the Combination of Dabrafenib and Trametinib:

REVISED TEXT

Pre-clinical studies have demonstrated that the combination of dabrafenib and trametinib is synergistic and/or enhances cell growth inhibition in majority of the BRAF V600E mutant melanoma, colon, thyroid, and lung cancer cells. The combination delayed dabrafenib resistance and reduced hyperproliferation skin adverse effect in pre-clinical models. The scientific rationale suggesting MEK-mediated re-activation of the MAPK pathway as a prominent mechanism of resistance to BRAFi single-agent therapy supports the combination study of trametinib with BRAFi in cancer [Alcala, 2012; Greger, 2012]. Because BRAF V600E mutated tumors have been shown to develop resistance to BRAF inhibitors, the combination of a BRAFi and MEKi provides a rational approach for dual vertical inhibition within the MAPK pathway to address such drug resistance[Johannessen, 2010]. Both have demonstrated substantial clinical activity with ORR between 28 to 53% and PFS ranging between 4.8 to 5.1 months. Further, the combination of both dabrafenib and trametinib indicated increased efficacy over both monotherapies with ORR of 63% and an increase of PFS to approximately 10 to 11 months for the 150 mg BID dabrafenib and 2 mg once daily trametinib dose cohort [Flaherty, 2012; Hauschild, 2012; Weber, 2012]. **Furthermore, the combination of dabrafenib and rametinib has demonstrated overall survival of a median of more than 25 months in advanced melanoma. This robust**

survival data for the combination is further supported by other efficacy measures such as progression free survival of >11 months, overall response rates of about 64-69% (13-16% of patients achieving a complete response) with duration of response of 12.9-13.8 months and a disease control rate of 94% across two randomized Phase-III studies (Long GV ,2015; Robert C et al, 2015).

Section 2.3 Dose Rational:

REVISED TEXT

Administration of dabrafenib + trametinib in the ongoing study BRF113220 indicates that dabrafenib does not have a clinically relevant effect on trametinib PK [GlaxoSmithKline Document Number **CM2010/0010/05**]. Cross-study comparisons indicate that exposure to dabrafenib may be greater after repeat-dose administration of dabrafenib in combination with trametinib relative to administration of dabrafenib alone at 150 mg BID. The combination was relatively well tolerated in study **BRF113220 and study MEK115306**, with toxicities manageable with appropriate intervention.

In this study, dabrafenib will be administered at the dose of 150 mg BID continuously that demonstrated significant benefit in PFS in a Phase III trial [Hauschild, 2012].

Trametinib administered at a dose of 2 mg once daily has demonstrated significant benefit in PFS (hazard ratio [HR] = 0.45), a confirmed response rate (22% vs. 8%), and OS (HR = 0.54) relative to chemotherapy [Flaherty, 2012]. **As determined in Phase III studies, the safety profile of the combination of dabrafenib and trametinib generally reflects the well established safety profiles of the individual approved agents, with toxicities that are manageable with appropriate intervention.**

Section 4 Study Design: 4th paragraph

REVISED TEXT

This is a Phase II, open-label, non-randomized, multi-center study of oral dabrafenib in combination with oral trametinib in subjects with rare cancers with the BRAF V600E mutation. The following histologies will be included in this study: ATC, BTC, GIST, WHO Grade 1 or 2 glioma, WHO Grade 3 or 4 (high-grade) glioma, NSGCT/NGGCT, ASI, HCL and MM (Cohorts 1 to 9, respectively). This study is designed to determine the ORR of dabrafenib in combination with trametinib in subjects with rare BRAF V600E mutated cancers. Further supporting secondary objectives include the evaluation of duration of response, PFS, OS, and safety of the combination treatment. For each cohort, up to 25 evaluable subjects will be enrolled **in the primary analysis cohort as defined in Section 5.2**. **If a given cohort is stopped early for efficacy, a histology specific expansion cohort may be opened to allow for additional patient enrollment (see Section 5.3).** Only subjects with histologically or cytologically confirmed advanced disease with no available treatment options as determined by locally or regionally available standards of care and by the treating physician's discretion will be eligible for enrollment. Subjects may be enrolled based on local BRAF V600E mutation results and mutation status will be confirmed by a central reference laboratory. …

…Subjects will receive dabrafenib 150 mg BID orally plus trametinib 2 mg once daily orally on a continuous dosing schedule. Subjects will undergo disease assessment of response after receiving at least 8 weeks of treatment. …

Section 5.2 Primary analysis cohorts

New Section

5.1. Primary analysis cohorts

The "primary analysis cohort" will be comprised of those patients enrolled within a histology-specific group prior to capping at 25 patients per cohort or prior to early stopping for efficacy or futility. The primary analysis cohort will form the basis of the Bayesian modelling.

Section 5.2 Expansion cohorts

New Section

5.2. Expansion cohorts

If a cohort closes early at an interim analysis because it meets the rules for early stopping for efficacy, an expansion cohort may be opened to allow additional patient enrollment for that particular histology. The patients in the expansion cohort will provide supportive efficacy data and will NOT contribute to the Bayesian modeling. The expansion cohort(s) will enroll subjects for the duration of trial enrollment.

The same general and histology specific eligibility criteria and T&E tables and biomarker/histology confirmation analyses will apply to patients in the expansion cohort.

Section 5.3 Number of Subjects: 1st paragraph

REVISED TEXT

Section 5.1 became 5.3

Subjects with BRAF V600E positive-mutations will be enrolled in separate cohorts based on histologic type. Once 16 weeks of response data is available from a minimum of 5 subjects in a particular cohort, interim analyses will occur every 12 weeks to assess whether enrollment in the particular cohort will be continued or closed. **For the primary analysis cohort,** enrollment will not exceed 25 subjects per histology cohort to ensure adequate subjects in the BRAF V600E confirmed population. After 25 subjects have been enrolled, the histology cohort will be closed to further enrollment. See Section 18.2 for sample size assumptions.

Section 5.4 Eligibility: 1st paragraph

REVISED TEXT

Specific information regarding warnings, precautions, contraindications, AEs, and other pertinent information on the study treatment(s) that may impact subject eligibility is provided in the IB for dabrafenib + trametinib combination [GlaxoSmithKline Document Number **CM2010/00010/05**].

Section 5.4.1 Inclusion Criteria: 5th Table 3

REVISED TEXT

SYSTEM	TEST	Histology	LABORATORY VALUES[1]
**Hematologic**	ANC[3]	ATC, BTC, GIST, WHO Grade 1-4 Glioma, NSGCT/NGGCT, ASI	≥1.2 × 10⁹/L
		MM	≥1 × 10⁹/L
	Hemoglobin[3]	ATC, BTC, GIST, WHO Grade 1-4 Glioma, NSGCT/NGGCT, ASI	≥9 g/dL
	Platelets[3]	ATC, BTC, GIST, NSGCT/NGGCT, ASI	≥75 × 10⁹/L
		MM	≥50 × 10⁹/L
		WHO Grade 1-4 Glioma	≥100 × 10⁹/L
	PT, PTT, INR[2, 3]	ATC, BTC, GIST, WHO Grade 1-4 Glioma, NSGCT/NGGCT, ASI, HCL, MM	≤1.5 times ULN
**Hepatic**	Albumin	ATC, BTC, GIST, WHO Grade 1-4 Glioma, NSGCT/NGGCT, ASI, HCL, MM	≥2.5 g/dL
	Total Bilirubin[4]	ATC	≤1.5 times ULN
		BTC	≤3 times ULN if stable for 14 days prior to enrollment
		GIST, WHO Grade 1-4 Glioma, NSGCT/NGGCT, MM	≤2 times ULN
		HCL	<3 times ULN
		ASI	≤2 times ULN OR ≤3 times ULN if stable for 14 days prior to enrollment for ampullary and periampullary carcinoma
**SYSTEM**	**TEST**	**Histology**	**LABORATORY VALUES[1]**
	ALT and AST[5]	ATC, GIST, NSGCT/NGGCT	≤2 times ULN without liver metastases ≤2.5 times ULN if documented liver metastases
		BTC, ASI, MM	≤3 times ULN without liver metastases ≤5 times ULN if documented liver metastases

		HCL	<5 times ULN
		WHO Grade 1, 2, 3 or 4 Glioma	≤3 times ULN
**Renal**	Creatinine	ATC, BTC, GIST, WHO Grade 1-4 Glioma, NSGCT/NGGCT, ASI, HCL, MM	≤1.5 mg/dL
	**OR**		
	Calculated Creatinine Clearance[6] or 24-hr Urine Creatinine Clearance	ATC, BTC, GIST, WHO Grade 1-4 Glioma, NSGCT/NGGCT, ASI, HCL, MM	≥50 mL/min
**Cardiac**	LVEF	ATC, BTC, GIST, WHO Grade 1-4 Glioma, NSGCT/NGGCT, ASI, HCL, MM	≥LLN[7] by ECHO

Abbreviations: ALT, alanine aminotransferase; ANC, absolute neutrophil count; ASI, adenocarcinoma of small intestine AST, aspartate aminotransferase; CBC, complete blood count; ECHO, echocardiogram; HCL, hairy cell leukemia; hr, hour; INR, international normalization n ratio; LLN, lower limit of normal; LVEF, left ventricular ejection fraction; PT, prothrombin time; PTT, partial thromboplastin time; ULN, upper limit of normal

1. Laboratory values are obtained within 14 days prior to first dose of study treatment.
2. **PTT and PT**/INR >1.5 times ULN will be acceptable in case of subjects receiving therapeutic anticoagulants such as warfarin as long as INR is monitored during the study according to clinical practice.
3. HCL: No minimum CBC criteria if the cytopenias are due to disease under study.
4. HCL: Subjects will be allowed to enroll with indirect hyperbilirubinemia; however, this condition must be confirmed due to hemolysis, hypersplenism or direct bilirubin ratio <35%, and requires approval from the Medical Monitor.
5. HCL: Subjects will be allowed to enrol with elevated ALT/AST values up to 5 times ULN; however this condition must be confirmed due to hemolysis and/or hypersplenism and not associated with hepatic dysfunction and required approval from the Medical Monitor.
6. Calculated by the Cockcroft-Gault formula (see Appendix 3).
7. If LLN is not defined for a given institution, then ejection fraction must be ≥50%.

NOTE: Subjects with AST, ALT or total bilirubin values outside the range(s) in Table 3 due to Gilbert's syndrome or asymptomatic gallstones are not excluded. Laboratory results obtained during Screening should be used to determine eligibility criteria. In situations where laboratory results are outside the permitted range, the investigator may opt to retest the subject and the subsequent within-range screening result may be used to confirm eligibility.

Section 5.4.2 Exclusion Criteria: 3rd bulletpoint

REVISED TEXT

Prior radiotherapy less than 14 days prior to enrollment, *except* for WHO Grade 1-4 glioma and ATC. Treatment-related AEs must have resolved prior to enrollment.

For WHO Grades 1, 2, 3, or 4 Glioma ONLY: Radiotherapy is not permitted within 3 months prior to enrollment (extended period of time of >3 months needed to prevent subjects with pseudo-progression from radiotherapy from being enrolled in the study). Subjects may be ≥-**2** weeks from radiotherapy if a new lesion relative to the pre-radiation MRI develops outside the primary radiation field. Treatment-related AEs must have resolved prior to enrollment.

For ATC Only: Radiotherapy is not permitted within 7 days prior to enrollment. Treatment-related AE(s) must have resolved prior to enrollment.

Section 5.4.2.1.3 WHO Grade 1, 2, 3 or 4 Glioma: Note

REVISED TEXT

1. Prior treatment with enzyme-inducing anticonvulsants within 14 days prior to enrollment.

2. Radiotherapy treatment within 3 months prior to enrollment.

   NOTE: Extended period of time (>3 months) needed to prevent subjects with pseudo-progression from radiotherapy being enrolled in the study**. Subjects may be ≥2 weeks from radiotherapy if a new lesion relative to the pre-radiation MRI develops outside the primary radiation field. Treatment-related AEs must have resolved prior to enrollment.**

Section 7.1 Time and Event Table

REVISED TEXT

	Screening	Treatment[5]						Follow-up Visit[55]	Extended Follow-up[58]
		Day 1 [5,7]	Day 15 (±1 day)	Every 4 weeks (±3 days)	Every 8 weeks (±3 days)	Every 12 weeks (±3 days)	Every 16 weeks (±3 days)	Treatment or Study Withdrawal	Every 3 months (±14 days) *except when noted*
Clinical Assessments									
Informed Consent[1]	X								
Demographics	X								
Medical History[2]	X	X[8]							
Disease Characteristics[3]	X								
BRAF V600E Mutation Testing (Mandatory)[4]	X								
ECOG Performance Status	X[6]	X[8]		X				X	
Safety Assessments									
Physical Exam (including weight)	X (Complete)[9]	X (Brief)[10]		X (Brief)[10]				X (Complete)[9]	
Height	X[11]								
Dermatologic Exam	X (Full)[12]			X (Brief)[13]				X (Brief)[13]	X (Brief)[13]
Ophthalmic Exam[14]	X			Week 4 Only					
Vital Signs[15]	X[6]	X		X				X	
12-lead ECG[16]	X[6]	X[8]		X				X	
ECHO[17]	X			Week 4 Only		X		X[57]	
[redacted]									
Adverse Events		Continuous							

	Screening	Treatment[5]						Follow-up Visit[55]	Extended Follow-up[58]
		Day 1 [5,7]	Day 15 (±1 day)	Every 4 weeks (±3 days)	Every 8 weeks (±3 days)	Every 12 weeks (±3 days)	Every 16 weeks (±3 days)	Treatment or Study Withdrawal	Every 3 months (±14 days) *except when noted*
Safety Assessments continued									
Concomitant Medications	X[6]	X	X	X				X	
Laboratory Assessments: ALL Cohorts									
Chemistry and Hematology	X[6]	X[9]		X				X[56]	
Pregnancy test[19]	X[20] (serum)				X[21] (serum or urine)			X[21] (serum or urine)	
HbA1C	X[6]					X			
Coagulation: PT, PTT, INR	X[6]								
Urinalysis	X[6]	X[8]		X				X[56]	
Monitoring for Non-Cutaneous Secondary/Recurrent Malignancy[22]									
Head and neck exam[23]	X[6]					X			X[24]
Chest and Abdominal CT scan or MRI	X[6]	As clinically indicated							X[24]
Disease Assessment: ATC, ASI, BTC, GIST, NSGCT/NGGCT, and WHO Grade 1-4 Glioma Cohorts only									
Disease Assessment: Imaging[25]	X[26]				X[27]	X[27]		X[27]	X[27]
Disease Assessment: ATC Cohort ONLY									
TSH, Free T4	X[6]				X			X	
Histology Confirmation: ATC and WHO Grade 1-4 Glioma Cohorts ONLY									
**Tissue Sample for Histology Confirmation[28]**		**X**							
Disease Assessment: NSGCT/NGGCT Cohort ONLY									

		Treatment[5]						Follow-up Visit[55]	Extended Follow-up[58]
	Screening	Day 1 [5,7]	Day 15 (±1 day)	Every 4 weeks (±3 days)	Every 8 weeks (±3 days)	Every 12 weeks (±3 days)	Every 16 weeks (±3 days)	Treatment or Study Withdrawal	Every 3 months (±14 days) *except when noted*
AFP, β-HCG	X[6]			X[29]	Every 8 to 12 weeks[29]			At Disease Progression[29]	
Disease Assessment: BTC and ASI ONLY									
CEA, CA19-9	X[6]			X[30]	Every 8 to 12 weeks[30]			At Disease Progression[30]	
Disease Assessment: HCL Cohort ONLY[31]									
Blood sample for CBC	X[6]			X					
Peripheral Blood Sample Staining **for Hairy Cell count**	X			X					
BM biopsy **and aspirate** with H/E stain and IHC	X[34]	When counts are consistent with CR **or PR** for 4 weeks; if **evidence of response**, then **after** every 6 months twice, then **after a year** twice **and** then every 2 years[32]						At time of relapse	
Flow cytometry: BM aspirate sample[33]	X[34]	When counts are consistent with CR **or PR** for 4 weeks; if **evidence of response**, then **after** every 6 months twice, then **after a year** twice **and** then every 2 years[32]						At time of relapse	
Flow cytometry: peripheral blood sample[33]	X[34]			X					
Chest CT scan and Abdominal CT scan w/contrast	X[35]	When counts are consistent with CR **or PR** for 4 weeks; then 6 months later						At time of relapse	
Disease Assessments: MM Cohort ONLY									
Skeletal surveys[36]	X						X	X	
Extramedullary Disease Assessment[37]	X				X			X	
BM aspirate	X[38]				At Week 8 and at time of achieving CR or best response[39]			At Disease Progression	
BM biopsy	X[38]				At Week 8 and at time of achieving CR or best response[39]			At Disease Progression	
SPEP[40]	X			X		X		X	
UPEP[41,42]	X			X		X		X	

	Screening	Treatment[5]						Follow-up Visit[55]	Extended Follow-up[58]
		Day 1 [5,7]	Day 15 (±1 day)	Every 4 weeks (±3 days)	Every 8 weeks (±3 days)	Every 12 weeks (±3 days)	Every 16 weeks (±3 days)	Treatment or Study Withdrawal	Every 3 months (±14 days) *except when noted*
CRP, β2 microglobulin; IgG, IgA and IgM[43]	X			X		X		X	
Serum FLC assay[43]	X			X		X		X	
[redacted]									
Exploratory Assessments: All Cohorts									
Optional PGx: blood sample[46]		X							
Exploratory Assessments: ATC, ASI, BTC, GIST, NSGCT/NGGCT, and WHO Grade 1-4 Glioma Cohorts only									
Optional tumor tissue sample for biomarker analysis	X[47]		X[48]					At Disease Progression[48]	
Optional peripheral blood sample for biomarker analysis[49]	X		X					At Disease Progression	
Exploratory Assessments: HCL Cohort ONLY									
Optional Ex Vivo Assay[50]	X							X	
Exploratory Assessments: HCL and MM Cohorts ONLY									
Optional BM aspirate sample for biomarker analysis	X[51]		X[52]					At Disease Progression[52]	
Optional peripheral blood sample for biomarker analysis[53]	X		X					At Disease Progression	
Study Treatments									

	Screening	Treatment[5]						Follow-up Visit[55]	Extended Follow-up[58]
		Day 1 [5,7]	Day 15 (±1 day)	Every 4 weeks (±3 days)	Every 8 weeks (±3 days)	Every 12 weeks (±3 days)	Every 16 weeks (±3 days)	Treatment or Study Withdrawal	Every 3 months (±14 days) *except when noted*
Dispensation of study treatments		X[54]		X					
Dabrafenib Dosing		Continuous Twice Daily Dosing							
Trametinib Dosing		Continuous Once Daily Dosing							
Compliance Assessment				X				X	

Abbreviations: AFP, alpha- fetoprotein; ANC, absolute neutrophil count; AP, anteroposterior; ASI, adenocarcinoma of small intestine; β-HCG, beta-human chorionic gonadotropin; BM, bone marrow ; CBC, complete blood count; CR, complete response; CRP, C-reactive protein; CT, computed tomography; ECG, electrocardiogram; ECHO, echocardiogram; ECOG, Eastern Cooperative Oncology Group; [redacted]; FFPE, formalin-fixed, paraffin- embedded; FISH, fluorescence in situ hybridization; FLC, free light chain; H/E, Hematoxylin and eosin; hr(s), hour(s); ICF, informed consent form; IHC, immunohistochemistry; INR, international normalization ratio; [redacted]; PGx, pharmacogenetics; [redacted]; PT, prothrombin time; PTT, partial thromboplastin time; SPEP, serum protein electrophoresis; TSH, thyroid stimulating hormone; UPEP, urine protein electrophoresis

1. ICF: Informed consent should be obtained prior to performing any study-related procedure(s).
2. MEDICAL HISTORY: Medical History will include past and current medical conditions, including cardiovascular medical history and risk factors
3. DISEASE CHARACTERISTICS: Disease characteristics will include date of initial diagnosis, primary tumor type, histology, stage, etc.
4. MUTATION TESTING: Subjects **may** be enrolled based on local determination of BRAF V600E mutation status. A subject may be tested for the BRAF V600E mutation greater than 28 days prior to enrollment. (Any past results of local BRAF V600E mutation testing may be used to determine eligibility regardless of when the results were obtained.) To enroll a subject, the result of mutation testing from a sponsor designated central reference laboratory is not required; however, if the subject does not have a local result available, the sponsor designated central reference laboratory mutation test result will be required to determine eligibility for enrollment. NOTE: *For subjects with a solid tumor:* An archived or fresh tumor tissue sample must be available to send to a sponsor designated central reference laboratory for confirmation testing. NOTE: *For subjects with HCL or MM:* A fresh BM aspirate sample and a peripheral blood sample are required for confirmation testing by a sponsor designated central reference laboratory. If a BM aspirate cannot be obtained due to a dry tap, please contact the Medical Monitor. **In the event of a dry tap, fresh or archived tumor tissues such as lymph node or plasmacytoma biopsies may be acceptable pending medical monitor approval.** An archived tissue sample (such as FFPE tissue sample from a BM biopsy), BM core samples and BM clot samples cannot be used. The same BM aspirate sample may be used for BRAFmutation testing, the baseline assessment, [redacted]
5. Dose interruptions should not alter the assessment schedule.

6. **SCREENING** All screening assessments must be completed within 14 days prior to first dose except informed consent. Disease Assessment (if MRI is used) can occur within 35 days prior to the first dose. Note: Procedures conducted as part of the subject's routine clinical management (e.g., blood counts, imaging study) and obtained prior to signing of informed consent may be utilized for screening or baseline purposes provided these procedures meet the protocol requirements.
7. DAY 1: Day 1 is defined as the date of first dose of study treatments.

[redacted]

9. COMPLETE PHYSICAL EXAM: A complete physical exam, including pelvic and rectal exams, will be performed at Screening and at the Follow-Up Visit (see Section 7.4.1).
10. BRIEF PHYSICAL EXAM: A brief physical exam will be performed on Day 1 (if more than 72 hrs since the complete physical exam was performed for Screening) and every 4 weeks while on study treatment.
11. HEIGHT: Height will be measured as part of complete physical exam performed at Screening only.
12. FULL DERMATOLOGICAL EXAM: A full dermatological exam will be performed at Screening (**or within 35 days prior to the first dose**) only. A biopsy in and/or around a new skin lesion(s) or a lesion(s) that changes during the study is required if clinically indicated (refer to Section 7.4.2).
13. BRIEF DERMATOLOGICAL EXAM**:** A brief dermatological exam will be performed every 4 weeks thereafter unless otherwise required to be performed more frequently. A brief dermatological exam should be performed every 4 weeks (±7 days) for the first 6 months after discontinuation of study treatments. A biopsy in and/or around a new skin lesion(s) or a lesion(s) that changes during the study is required if clinically indicated (refer to Section 7.4.2).
14. **OPHTHALMIC** EXAM: **Ophthalmic** exams will be performed at Screening (**or within 35 days prior to the first dose**), Week 4, and as clinically indicated thereafter.
15. VITAL SIGNS: *Vital signs* include systolic/diastolic BP, temperature, pulse rate and respiratory rate. Vital signs should be measured in a semi-supine position after a 5 minute rest. *Blood pressure:* Three readings of BP and pulse rate should be taken. The first reading should be rejected and the second and third readings averaged to give the measurement to be recorded in the eCRF. If persistent hypertension (see Section 8.1.1.3.1) is noted in 3 consecutive visits, clinic visits to monitor the increased BP should be scheduled independently from the per-protocol visits. Ideally, subsequent BP assessments should be performed within 7 days of initial finding.
16. ECG: A single 12-lead ECG should be performed after vital signs and before blood draws if assessments are planned at the same nominal time point. If clinically significant abnormality(ies) are seen, confirm with 2 additional ECGs taken at least 5 minutes apart. See Section 7.4.6 and SPM for additional information on ECG collection.
17. ECHO: ECHOs will be performed at Screening (**or within 35 days prior to the first dose**), Week 4, Week 12, and then every 12 weeks thereafter. See Section 7.4.7 and SPM for additional information on ECHO collection.

[redacted]

19. PREGNANCY TEST: Perform only in female subjects of childbearing potential.
20. PREGNANCY TEST: A serum pregnancy test should be performed no more than 7 days prior to the first dose of study treatments.
21. PREGNANCY TEST: A serum or urine pregnancy test will be performed every 8 weeks during the first 48 weeks of study treatment, then every 12 weeks thereafter, and at the Follow-up Visit.
22. MONITORING for NON-CUTANEOUS SECONDARY/RECURRENT MALIGNANCY: During study treatment, monitor as clinically appropriate which may include a head/neck exam every 3 months and a chest/abdominal CT scan or MRI every 6 months.
23. MONITORING for NON-CUTANEOUS SECONDARY/RECURRENT MALIGNANCY: This assessment is to be part of the physical exam and should include visual inspection of the oral mucosa and lymph node palpation.
24. MONITORING for NON-CUTANEOUS SECONDARY/RECURRENT MALIGNANCY: Following discontinuation of study treatments, monitoring (which may include a chest/abdominal CT scan or MRI) should continue for up to 6 months or until initiation of new anti-cancer therapy.
25. DISEASE ASSESSMENTS (Solid Tumor Cohorts only): See Section 7.5.1 for details on baseline and post-baseline disease assessments for the solid tumor cohorts: ATC, ASI, BTC, GIST, NSGCT/NGGCT and WHO Grade 1-4 Glioma.

26. DISEASE ASSESSMENTS (Solid Tumor Cohorts only): Baseline disease assessments must be performed within 28 days prior to enrollment (or within 35 days if MRI is used for disease assessment).
27. DISEASE ASSESSMENTS (Solid Tumor Cohorts only): Disease assessments are every 8 weeks during the first 48 weeks of study treatment, then every 12 weeks thereafter unless otherwise noted, and at the Follow-up Visit. Confirmatory scan(s) are to be performed ≥28 days after documented CR or PR. If subject discontinues treatment for reasons other than disease progression, disease assessments should continue every 12 weeks until disease progression, initiation of new anti-cancer treatment, subject withdrawal of consent, or death.
28. **HISTOLOGY CONFIRMATION: (ATC and WHO grade 1-4 cohorts only) A tissue specimen for possible retrospective histology confirmation should be obtained and submitted to a Novartis designated central reference laboratory. Please refer to the SPM for the requirements of the tissue specimen that is required for the histology confirmation.**
29. **NSGCT/NGGCT ONLY: AFP, B-HCG and LDH are the disease assessment markers for the NSGCT/NGGCT cohort.** LDH result obtained from clinical chemistry panel can be used as a tumor marker result. Regardless of baseline values of AFP, β-HCG and LDH, a blood sample will be drawn every 4 weeks for first 48 weeks of study treatment, then every 8 to 12 weeks thereafter, and at the time of disease progression. Synchronize the collection of the blood sample with the imaging schedule.
30. BTC/ASI ONLY: A blood sample will be drawn to assess CEA and CA 19-9 every 4 weeks for first 48 weeks of study treatment, then every 8 to 12 weeks thereafter, and at the time of disease progression. Synchronize the collection of the blood sample with the imaging schedule.
31. **HCL ONLY: All required disease assessments necessary for a post-baseline response determination should be performed every 4 weeks +/- 3 days. A response is to be determined every 4 weeks based on the response criteria (please consult Appendix 12) using data collected from the scheduled disease assessments.**
32. HCL ONLY**: BM biopsy and aspiration with H/E stain and IHC will be performed when blood counts are consistent with CR or PR for 4 weeks (please consult Appendix 12). If a patient has a CR, and then blood counts become consistent with a CR for 4 weeks, a repeat BM aspirate and biopsy is required to assess a response of CR. Following best response, a bone marrow biopsy and aspirate must be performed every 6 months for two time intervals, then after a year for two time intervals, then every two years.**
33. HCL ONLY: Recommend that flow cytometry panel consists of CD19, CD20, CD22, SmIg, CD11c, CD25, CD103 and CD123. **Flow cytometry of the bone marrow aspirate sample will be performed when blood counts are consistent with CR or PR for 4 weeks (please consult Appendix 12). If a patient has a CR, and then blood counts become consistent with a CR for 4 weeks, a repeat flow cytometry of the BM aspirate is required to assess a response of CR. Following best response, a flow cytometry of the bone marrow aspirate must be performed every 6 months for two time intervals, then after a year for two time intervals, then every two years**.
34. **HCL ONLY: BM biopsy (with H/E stain and IHC), BM aspirate (with flow cytometry) and peripheral blood sample (with flow cytometry) will be assessed within 28 days prior to enrollment.**
35. HCL ONLY**:** A chest CT scan and an abdominal CT scan with contrast will be performed **within 28 days prior to enrollment**. If an abdominal CT scan with contrast cannot be performed, then a MRI may be performed within **35 days prior to enrollment**. If lymph nodes or spleen are enlarged at baseline, then the abnormal organ needs to be followed on post-baseline assessments using the same modality of imaging.
36. MM ONLY: **A skeletal survey should be performed within 28 days prior to enrollment.** If myeloma associated bony lesions are present at baseline, skeletal surveys should be performed every 16 weeks or more frequently in order to assess response or upon clinical suspicion of progressive disease. Skeletal surveys typically consist of lateral radiographs of the skull, AP and lateral views of the spine, and AP views of the humeri, ribs, pelvis, and femurs.
37. MM ONLY: **Each patient should be clinically examined for soft tissue plasmacytomas/extramedullary disease at each visit for response assessment**. If extramedullary disease is present at baseline, then Chest/Abdominal/Pelvis CT scan with contrast should be performed **to confirm and to determine size and location of the soft tissue plasmacytoma.** If no disease is identified at baseline, then subsequent scans are only required if clinically indicated. Alternative imaging modality (i.e., MRI) may be used if it is better to assess the lesion and approved by a Medical Monitor. The same imaging modality should be used for all subsequent assessments.
38. MM ONLY: BM aspirate and BM biopsy samples will be assessed by IHC, flow cytometry, FISH and cytogenetics **within 90 days prior enrollment**.

39. MM ONLY: Flow cytometry for plasma cell enumeration and phenotyping will be performed on BM aspirate and BM biopsy samples at Week 8 and then at the time of achieving a CR or best response. NOTE: Density gradient is not recommended.
40. MM ONLY: SPEP should be performed **within 28 days prior to enrollment**, every 4 weeks for the first 48 weeks of study treatment, then every 12 weeks thereafter, and at the Follow-up Visit.
41. MM ONLY**:** UPEP must be performed using a 24-hr urine sample.
42. MM ONLY: If UPEP is positive at Screening (**or within 28 days prior to enrollment**), then a UPEP should be performed every 4 weeks for first 48 weeks of study treatment, then every 12 weeks thereafter, and at the Follow-up Visit.
43. MM ONLY: CRP, β2 microglobulin; immunoglobulins (IgG, IgA and IgM) and serum FLC assay should be performed **within 28 days prior to ernollment**, every 4 weeks for the first 48 weeks of study treatment, then every 12 weeks thereafter, and at the Follow-up Visit.

[redacted]

46. PGx: (Optional) If informed consent for PGx research has been obtained, a 6 mL blood sample for PGx analysis will be collected and sent for central testing on or after Day 1.
47. PD Biomarkers (Optional): For ACT, ASI, BTC, GIST, NSGCT/NGGCT and WHO Grade 1- 4 Glioma: A pre-dose FFPE tumor tissue sample is to be obtained during Screening if consent is provided. If a fresh FFPE tumor tissue for BRAF mutation testing is obtained within 28 days of Screening, it can also be used for this pre-dose sample.
48. PD Biomarkers (Optional): For ACT, ASI, BTC, GIST, NSGCT/NGGCT, and WHO Grade 1-4 Glioma: On Day 15 (±3 days) and at the time of disease progression, a post-dose FFPE tumor tissue sample will be collected if a pre-dose tumor biopsy was obtained during Screening.
49. PD Biomarkers (Optional): For ACT, ASI, BTC, GIST, NSGCT/NGGCT, and WHO Grade 1-4 Glioma: If a tumor biopsy is performed at a given time point (see Footnotes 47 and 48), a single 7.5 mL peripheral blood sample will be collected at the corresponding time point: Screening, on Day 15 (±3 days), and at the time of disease progression.
50. Exploratory Assessments (Optional): For HCL Cohort only: Blood samples (approximately 25 mL) will be collected from HCL subjects (see Section 13.2 for further details) at the corresponding time point: Screening and at the Follow-up Visit. The investigator should use their clinical judgment in determining whether it is appropriate for a subject enrolled in the HCL cohort to have this additional blood drawn. In addition, if the IEC or IRB does not approve the site for participation in the sub-study, no subjects at those sites will be included in the sub-study.
51. PD Biomarkers (Optional): *For HCL and MM Cohorts:* A pre-dose BM aspirate sample is to be obtained during Screening if consent is provided. If a fresh BM aspirate sample was taken for BRAF mutation testing within 28 days of Screening, it can be used for this pre-dose sample.
52. PD Biomarkers (Optional): *For HCL and MM Cohorts*: On Day 15 (±3 days) and at the time of disease progression, a post-dose BM aspirate sample will be collected if a pre-dose BM aspirate sample was obtained during Screening.
53. PD Biomarkers (Optional): *For HCL and MM Cohorts:* If a BM aspirate is performed at a given time point (see Footnote 51 and Footnote 52), a single 7.5 mL peripheral blood sample will be collected at the corresponding time point: Screening, on Day 15 (±3 days), and at the time of disease progression.
54. STUDY TREATMENT: Subjects must receive their supply of study treatments within 3 days of enrollment.

55. FOLLOW-UP VISIT: The Follow-up Visit should take place within 28 days (+7 days) of the last dose of study treatments. If the subject is unable to return to clinic due to hospitalization, site staff is encouraged to call subject for assessment of AEs.
56. FOLLOW-UP VISIT: All safety and laboratory assessments should be performed unless the assessments were performed within 4 weeks prior to the Follow-up Visit.
57. FOLLOW-UP VISIT: If the last cardiac assessment was >12 weeks prior to the Follow-up Visit, an ECHO should be performed.
58. EXTENDED FOLLOW-UP: Subjects should be contacted every 4 weeks (±7 days) for the first 6 months and then every 3 months (±14 days) until death, withdrawal of consent, or subject is lost to follow-up. Contact may include a clinic visit, a telephone contact, an e-mail or by mail. The initiation of any new anti-cancer treatment(s) and date of last contact should also bedocumented.

Section 7.3.2 Baseline Documentation of Target and Non-Target Lesions for Solid Tumor Histologies

REVISED TEXT

All baseline lesion assessments must be performed within 28 days of enrollment **except where indicated differently in the T&E table (Section 7.1).** Guidance on baseline documentation of target and non-target lesions will be provided in the SPM.

Section 7.4.2 Dermatological Examinations; 1st paragraph

REVISED TEXT

Dermatological examinations will be performed by an investigator or the subject may be referred to a dermatologist, at the discretion of the investigator. **A complete or full body dermatological examination will be performed at the time points indicated in the Time and Event Table (Section 7.1) to identify any abnormal skin lesions.** All abnormal findings (i.e., suspicious for proliferative skin lesions) will be identified and photographed during the Screening examination. Brief dermatological examinations should be performed every 4weeks or more frequently if clinically indicated while on study treatment and every 4 weeks (±7 days) for the first 6 months following treatment discontinuation. Wherever possible, the same individual should perform all of the examinations. Follow-up skin examinations by a referral dermatologist should be conducted if clinically indicated.

Section 7.4.3 Ophthalmic Examinations; 1st paragraph

REVISED TEXT

Subjects are required to have a standard ophthalmology exam performed by an ophthalmologist at the time points indicated in the Time and Event Table (Section 7.1). The exam will include best corrected visual acuity, tonometry, slit lamp biomicroscopic examination, visual field examination, and dilated indirect fundoscopy with special attention to retinal abnormalities. Optical coherence tomography is strongly recommended at scheduled visits, and if retinal abnormalities are suspected. Other types of ancillary testing including color fundus photography and fluorescein angiography are also recommended if clinically indicated. Refer to Section 8.2.7 for additional details on management of visual changes.

Section 7.4.3 Echocardiogram; 1st paragraph

REVISED TEXT

Echocardiograms (ECHOs) will be performed to assess cardiac ejection fraction and cardiac valve abnormalities. **ECHOs will be performed at the time points indicated in the Time and Event Table (Section 7.1).** The evaluation by the echocardiographer should include an evaluation for LVEF and both right and left-sided valvular lesions.

The procedure (ECHO) to document the subject's baseline LVEF status must be used

consistently throughout the study. If possible, it is also preferred that interpretation of LVEF status be performed consistently by the same reviewer throughout the study.

Section 7.4.8 Clinical Laboratory Assessments; Table 3

REVISED TEXT

Hematology

RBC Count	RBC Indices (at Screening and if hemoglobin decreases ≥2 g/dL compared to baseline):	Automated WBC Differential:
Platelets<br>WBC Count (absolute)<br>Hemoglobin<br>Hematocrit<br>Absolute neutrophil count (ANC)	• MCV<br>• MCH<br>• MCHC<br>• Reticulocyte Count	• Lymphocytes<br>• Monocytes<br>• Eosinophils<br>• Basophils

Clinical Chemistry

BUN	Potassium	Total Protein	Total and direct bilirubin[1]
Creatinine[3]	Chloride[2]	Phosphorus	AST
Glucose	Uric Acid[2]	Magnesium	ALT
Sodium	Calcium	LDH	Alkaline Phosphatase
Albumin	HbA1c	**GGT**	

Coagulation

PT
PTT
INR

Urinalysis[4]

Color	Specific gravity	Ketones	Urobilinogen
Appearance	Glucose	Blood	
pH	Protein[5]	Leukocyte esterase	

Other Tests

**Amylase and lipase [monitor via local laboratory when clinically indicated to evaluate certain AEs (i.e., abdominal pain, pancreatitis, etc.)**
Serum or urine β-hCG[6]
For subjects with a history of chronic HBV and/or HCV, the following tests will be performed at Screening:<br>• Viral hepatitis serology;<br>• Hepatitis B surface antigen and Hepatitis B core antibody (IgM); and/or<br>• Hepatitis C RNA

Abbreviations: ALT, alanine aminotransferase; ANC, absolute neutrophil count; AST, aspartate aminotransferase; β-hCG, Beta-human chorionic gonadotropin; BUN, blood urea nitrogen; GGT, gamma glutamyl transferase; INR, international normalization ratio; HbA1C, hemoglobin A1C; HBV, hepatitis B virus; HCV, hepatitis C virus; LDH, lactate dehydrogenase; MCV, mean corpuscular volume; MCH, mean corpuscular hemoglobin; MCHC, mean corpuscular hemoglobulin concentration; PT, prothrombin time; PTT, partial thromboplastin time; RBC, red blood cells; TSH, thyroid stimulating hormone; ULN, upper limit of normal; WBC, white blood cells

1. Direct bilirubin is required only if the total bilirubin is elevated (≥2 times the ULN)
2. Chemistry evaluation of chloride or uric acid is not required where there are logistical constraints.
3. If serum creatinine is >2.0 mg/dL, calculate creatinine clearance using standard Cockcroft-Gault method (Appendix 3).
4. If urine protein result is not available, urine albumin result may be used.

5. For female subjects of childbearing potential only, unless subject has had a hysterectomy, undergone a tubal ligation within 1 year prior to Screening or has been post-menopausal for at least 1 year prior to Screening.

Section 7.4.8.1 Additional Histology Specific Assessments and Histology Confirmation; Headline

REVISED Header

Additional Histology Specific Assessments **and Histology Confirmation**

Section 7.4.8.1 Additional Histology Specific Assessments and Histology Confirmation; new paragraph (4th)

REVISED TEXT

Histology Confirmation for ATC and WHO Grade 1-4 Glioma Cohorts ONLY:

A tissue specimen from the biopsy of a primary or metastatic site must be submitted to a GSK designated central reference laboratory as soon as possible after enrollment for possible retrospective confirmation of histologic type. Refer to the SPM and the Lab Manual for details regarding sample submission.

Section 7.4.9 Monitoring for Non-Cutaneous Secondary/Recurrent Malignancy; 2nd paragraph

REVISED TEXT

Abnormal findings should be managed according to standard clinical practice.

Section 7.5.1 Disease Assessments for Solid Tumor Histologies; Tables

REVISED TEXT

Histology	Area(s) of Assessment	Imaging to be Performed	Alternative Imaging Methods if Contraindications are Present
ATC[1]	Brain	MRI with contrast	1st alternative: MRI without contrast<br>2nd alternative: CT scan with contrast
	Neck	CT scan with contrast	1st alternative: a MRI with contrast 2nd alternative: CT scan without contrast (if 1st alternative is contraindicated)
	Chest/Abdomen (including liver and adrenal glands)	CT scan[2]	

BTC/ASI	Chest/Abdomen/Pelvis	CT scan with contrast	CT scan without contrast of the Chest and MRI with contrast of Abdomen/Pelvis
**Histology**	**Area(s) of Assessment**	**Imaging to be Performed**	**Alternative Imaging Methods if Contraindications are Present**
NSGCT/NGGCT	Chest/Abdomen/Pelvis	CT scan with contrast	CT scan without contrast of the Chest and MRI with contrast of Abdomen/Pelvis
GIST	Chest/Abdomen/Pelvis	CT scan with contrast	CT scan without contrast of the Chest and MRI with contrast of Abdomen/Pelvis
ATC[1]<br>BTC<br>GIST<br>NSGCT/NGGCT<br>ASI<br>WHO Grade 1-4 Glioma	Regions with known or suspected metastatic lesions	CT scan, MRI, or bone scan as appropriate	
WHO Grade 1- 4 Glioma	Brain	MRI with contrast[3]	**N/A**
NSGCT/NGGCT[4]	Brain (for pituitary GCT)	MRI with contrast	1st alternative: MRI without contrast<br>2nd alternative: CT scan with contrast

Abbreviations: ASI, adenocarcinoma of small intestine; ATC, anaplastic thyroid carcinoma; BTC, biliary tract carcinoma; CNS, central nervous system; CR, complete response; CT, computed tomography; GCT, germ cell tumor; GIST, gastrointestinal stromal tumor; MRI, magnetic resonance imaging; NGGCT, non-geminomatous germ cell tumor; NSGCT, non-seminomatous germ cell tumor; PET, positron emission tomography; SPM, Study Procedures Manual; WHO, World Health Organization

1. For ATC subjects without CNS and neck disease at baseline, subsequent brain and neck imaging should only be performed as clinicallyindicated.
2. Optional: Whole body PET/CTscan
3. MRI of the brain with contrast is mandated in this cohort; CT scan is not permitted. Please refer to imaging details in the SPM.
4. For NSGCT/NGGCT subjects *without* CNS disease at baseline, subsequent MRI of the brain with contrast should only be performed as clinically indicated (e.g., symptoms suggestive of CNS progression). For subjects with CNS disease at baseline, a MRI of the brain with contrast is required every 4 to 8 weeks or as clinically indicated. In addition, in order to confirm a CR in a subject with brain disease at baseline, a MRI of the brain with contrast must be performed 1 week prior to the first set of images showing CR to 4 weeks after the next protocol specifiedassessment.

Post-Baseline Assessments: All post-baseline assessments require imaging of disease sites identified by baseline scans. The same imaging modality used at baseline should be utilized for all post-baseline assessments. If subject discontinues treatment prior to

Week 48 for reasons other than disease progression, disease assessments (imaging) should continue every 12 weeks until a new treatment isinitiated.

Histology	Area(s) of Assessment	Imaging to be Performed	Alternative Imaging Methods if Contraindications are Present

ATC	Chest/Abdomen (including liver and adrenal glands)	CT scan[1]	
BTC, ASI	Chest/Abdomen/Pelvis	CT scan with contrast	CT scan without contrast of the Chest and MRI with contrast of Abdomen/Pelvis
WHO Grade 1-4 Glioma	Brain	MRI with contrast[2]	**N/A**
NSGCT/NGGCT, GIST	Chest/Abdomen/Pelvis	CT scan with contrast	CT scan without contrast of the Chest and MRI with contrast of Abdomen/Pelvis

Abbreviations: ASI, adenocarcinoma of small intestine; ATC, anaplastic thyroid carcinoma; BTC, biliary tract carcinoma; CT, computed tomography; GIST, gastrointestinal stromal tumor; MRI, magnetic resonance imaging; NGGCT, non-geminomatous germ cell tumor; NSGCT, non-seminomatous germ cell tumor; SPM, Study Procedures Manual; WHO, World Health Organization

1. Optional: Whole body PET/CTscan
2. MRI with contrast is mandated in this cohort; CT scan is not permitted. Please refer to imaging details in SPM.

Section 7.5.2.1 Laboratory and Disease Assessments: HCL

REVISED TEXT

Baseline Assessments: The following baseline disease assessments are **will**

be performed at the time points indicated in the Time and Event Table (Section 7.1):

- Complete blood count
- Routine stain of peripheral blood sample
- BM **aspirate and** biopsy sample with H/E stain and IHC
- Flow cytometry on BM aspirate sample and peripheral blood sample
- Chest CT scan and abdominal CT scan with contrast

NOTE: MRI of the abdomen may be performed if abdominal CT cannot be performed.

The method used to document baseline status is used consistently throughout the study to facilitate comparison of results.

If lymph nodes and spleen were enlarged at baseline, they must be followed by post- baseline assessments using a consistent imaging modality.

Post-Baseline Assessments**: A response is to be determined every 4 weeks based on the HCL response criteria (please consult Appendix 12) using data collected from the scheduled disease assessments.**

- Complete blood count

- Routine stain of peripheral blood sample
- BM **aspirate and** biopsy sample with H/E stain and IHC
- Flow cytometry on BM aspirate sample will be performed **as** indicated in the Timeand Event Table (Section 7.1).
- Flow cytometry on peripheral blood sample will be performed as indicated in the Time and Event Table (Section 7.1)
- If lymph nodes and/**or** spleen were enlarged at baseline, they must be followed by post-baseline assessments using a consistent imaging modality as indicated in the Time and Event Table (Section 7.1).

Section 7.5.2.2 Laboratory and Disease Assessments: MM

REVISED TEXT

Baseline Assessments: The following baseline disease assessments **will be performed at the time points indicated in the Time and Event Table (Section 7.1):**

- Skeletal surveys
- Extramedullary disease assessment
- NOTE: An alternative imaging modality (i.e., MRI) may be used to assess extramedullary disease if the investigator considers the other modality to be better in assessing the lesion and if approval is received from a Medical Monitor. The same type of imaging should be used for all subsequent assessments.
- BM aspirate and biopsy samples: Samples will be assessed by IHC, flow cytometry, fluorescence *in situ* hybridization (FISH) and cytogenetics.
- UPEP: to be performed using a 24-hr urine collection
- SPEP
- CRP, β-2 microglobulin, serum FLC assay, levels of immunoglobulin (Ig)G, IgA, and IgM

Post-baseline Assessments: All post-baseline assessments require imaging of disease sites identified by baseline scans. The same imaging modality used at baseline should be utilized for all post-baseline assessments.

- BM aspirate and biopsy samples will be performed and assessed as indicated in the Time and Event Table (Section 7.1)
- Laboratory tests (SPEP, **UPEP,** CRP, β-2 microglobulin, serum FLC assay, levels of IgG,

IgA, and IgM will be performed as indicated in the Time and Event Table (Section 7.1).

- Skeletal surveys: Post-baseline assessments only need to be performed if disease is noted on the baseline assessment. If no disease is identified at baseline, then subsequent skeletal surveys are only required if clinically indicated.

- Extramedullary disease assessment: Post-baseline assessments only need to be performed if disease is noted on the baseline assessment. If no disease is identified at baseline, then subsequent assessments are only required if clinically indicated.

Section 8.1 Dose Modification; 2nd and 3rd paragraph

REVISED TEXT

If a dose reduction of trametinib is required, but the toxicity resolves and no additional toxicities are seen after **one** treatment cycles **(4 weeks)**, the dose of trametinib may be re-escalated but should not exceed 2 mg once daily.

If a dose reduction of dabrafenib is required, but the toxicity resolves and no additional toxicities are seen after one treatment cycle (4 weeks), the dose of dabrafenib may bere-escalated but should not exceed 150 mg BID daily.

Section 8.1.1.3.2 Management of Hypertension; Table 14

REVISED TEXT

Hypertension	Action and Dose Modification
(A). Asymptomatic and persistent[1] SBP of ≥140 mmHg and <160 mmHg, or DBP ≥90 mmHg and <100 mmHg, or a clinically significant increase in DBP of 20 mmHg (but still <100 mmHg).	• Continue study treatments at the current dose<br>• Adjust current or initiate new antihypertensive medication(s)<br>• Titrate antihypertensive medication(s) during the next 2 weeks as indicated to achieve well-controlled[2] BP<br>• If BP is not well controlled within 2 weeks, consider referral to a specialist and go to scenario (B).
(B). Asymptomatic SBP ≥160 mmHg, or DBP ≥100 mmHg, or failure to achieve well-controlled BP within 2 weeks in scenario (A).	• Interrupt study treatments if clinicallyindicated<br>• Adjust current or initiate new antihypertensive medication(s)<br>• Titrate antihypertensive medication(s) during the next 2 weeks as indicated to achieve well-controlled BP<br>• Once BP is well controlled[2], restart study treatments reduced by one dose level[3]
(C). Symptomatic[4] hypertension or persistent SBP ≥160 mmHg, or DBP ≥100 mmHg, despite antihypertensive medication(s) and dose reduction of study	• Interrupt study treatments<br>• Adjust current or initiate new antihypertensive medication(s)<br>• Titrate antihypertensive medication(s) during the next 2 weeks as indicated to achieve well-controlled BP<br>• Referral to a specialist for further evaluation and follow-up is
Hypertension	Action and Dose Modification

treatment	recommended<br>• Once BP is well controlled[2], restart study treatments reduced by one dose level[3]
(D). Refractory hypertension unresponsive to above interventions or hypertensive crisis	• Permanently discontinue study treatments<br>• Continue follow-up per protocol.

Abbreviations: BP, blood pressure; DBP, diastolic blood pressure; mmHg, millimeters of mercury; SBP, systolic blood pressure

1. Hypertension detected in 2 separate readings during up to 3 consecutive visits
2. Well-controlled BP defined as SBP ≤140 mmHg and DBP ≤90 mmHg in 2 separate readings during up to 3 consecutive visits
3. Escalation to previous dose level can be considered if BP remains well-controlled for 4 weeks after restarting. Approval from the Medical Monitor is required.
4. Symptomatic hypertension defined as hypertension aggravated by symptoms (e.g. headache, lightheadedness, vertigo, tinnitus, episodes of fainting) that resolve after the BP is controlled within the normal range.

Section 8.1.2 Pancreatitis; 1st paragraph

REVISED TEXT

In the event of abdominal pain or suspected pancreatitis, amylase and lipase laboratory samples should be collected for confirmation of the diagnosis. Subjects should be closely monitored when re-starting **study treatment** after an episode of pancreatitis.

Section 8.2.1.2 Palmar-plantar Erythrodysesthesia

NEW TEXT

Palmar-plantar erythrodysesthesia (PPE) syndrome: Measures for PPE syndrome should include lifestyle modification (avoidance of hot water, traumatic activity, constrictive footwear, or excessive friction on the skin and the use of thick cotton socks and gloves, and shoes with padded insoles) and symptomatic treatments. Apply moisturizing creams frequently, topical keratolytics (e.g., urea 20 to 40% cream, salicylic acid 6%, tazarotene 0.1% cream, fluorouracil 5% cream), clobetasol propionate 0.05% ointment for erythematous areas, topical lidocaine 2%, and/or systemic pain medication such as nonsteroidal anti-inflammatory drugs, codeine, and pregabalin for pain. Dose modification may also be required.

Section 8.2.2.1 Cutaneous squamous cell carcinoma (cuSCC); 1st paragraph

REVISED TEXT

Cases of cuSCC (which include those classified as KA or mixed KA subtype) have been observed in subjects treated with dabrafenib and the combination of dabrafenib and trametinib [GlaxoSmithKline Document Number **CM2010/00010/05. In a Phase III study, 10% of patients receiving dabrafenib monotherapy developed cuSCC, with a median time to onset of the first occurrence of approximately 8 weeks. In patients who received dabrafenib in combination with trametinib, 3% of patients developed cuSCC with median time to onset of the first occurrence of 32 weeks.**

CuSCC/KA should be surgically removed according to institutional practices. Dose modification or interruption of study treatment is not required for cuSCC or KA, however cuSCC should be reported as an SAE. Tumor tissue should also be submitted for further analyses as described in the SPM.

Section 8.2.2.3 Non-Cutaneous Malignancies; 2nd paragraph

REVISED TEXT

A biopsy of the new malignancy should be taken, where possible, and submitted **locally** for further analyses with the results provided to the sponsor. Testing of these biopsies may include analysis of genomic alterations, which include but not limited to DNA, RNA and protein analysis of these biopsy specimens, and would analyze the biological pathways known to be associated with, and relevant to, BRAF-mutant tumor activation **including BRAF and/or RAS mutation testing**.

Section 8.2.3 Pyrexia; 2nd paragraph

REVISED TEXT

Subjects should be instructed on the importance of immediately reporting febrile episodes. In the event of a fever, the subject should be instructed to take anti-pyretics (i.e., ibuprofen or acetaminophen/paracetamol) as appropriate to control fever. The use of oral corticosteroids should be considered in those instances in which anti-pyretics are insufficient. In subjects experiencing **and after** pyrexia associated with rigors, severe chills, dehydration, hypotension, serum creatinine and other evidence of renal function should be monitored carefully.

Section 8.2.7 Visual Changes; 3rd paragraph

REVISED TEXT

Guidelines regarding management and dose reduction for visual changes considered to be related to **trametinib** treatment are provided in Table 25. **For events of uveitis and related toxicities (e.g. iritis, iridocyclitis), no dose modifications are required as long as effective local therapies can control ocular inflammation. If uveitis does not respond to local ocular therapy, withhold dabrafenib until resolution of ocular inflammation and then**

restart dabrafenib reduced by one dose level. No dose modification of trametinib is required when taken in combination with dabrafenib.

Section 10.2 Prohibited Medications and Non-Drug Therapies

REVISED TEXT

The use of illicit drugs and the following medications within 28 days or 5 half-lives, whichever is shorter, prior to start of treatment will not be allowed:

- **Antiretroviral drugs**
- **Herbal remedies (e.g., St. John's wort)**
- **Drugs that are strong inhibitors or inducers of CYP2C8 or CYP3A4 (Table 27):**

The use of certain medications and illicit drugs within 28 days or 5 half-lives, whichever is shorter, prior to randomization and for the duration of the study will not be allowed.

The following medications or non-drug therapies (excluding topical formulations) are also prohibited while on treatment in this study:

- Other anti-cancer therapies
- Other investigational drugs
- Antiretroviral drugs
- Herbal remedies (e.g., St. John's wort)
- ***For MM cohort only***: non-steroidal anti-inflammatory drugs (NSAIDS)

NOTE: Use of NSAIDS by subjects with MM is prohibited as it will worsen renal insufficiency.

- Drugs that are strong inhibitors or inducers of CYP2C8 or CYP3A4 (Table 27):

NOTE: Dabrafenib is metabolized primarily by CYP2C8 and CYP3A4. Co- administration of dabrafenib with ketoconazole, a CYP3A4 inhibitor, or gemfibrozil, a CYP2C8 inhibitor, increased the area under the concentration-time curve (AUC) of dabrafenib by 71% and 47%, respectively. Drugs that are strong inhibitors or inducers of CYP2C8 or CYP3A4 are likely to increase or decrease, respectively, dabrafenib concentrations. Strong inhibitors **or inducers** should only be used under special circumstances (e.g., as a single use for a procedure) while study treatment is interrupted as they may alter dabrafenib concentrations; consider therapeutic substitutions for these medications. Approval from the Medical Monitor is required in these situations.

Section 10.3 Medications to e used with Caution; 2nd bullet point

REVISED TEXT

•Dabrafenib has been shown to induce CYP3A4 and CYP2C9 in vivo using midazolam (CYP3A4 substrate) and S-warfarin (CYP2C9 substrate). Dabrafenib is an in vitro inducer of CYP2B6 and may affect other enzymes such as CYP2C8, CYP2C19, UDP- glucuronyl transferases, and transporters. Co-administration of dabrafenib and medicinal products which are affected by the induction of these enzymes such as **hormonal contraceptives**, warfarin, dexamethasone, antiretroviral agents, or Immunosuppressants may result in decreased concentrations and loss of effectiveness. Where possible consider substitutions of these medicinal products if therapeutic effects cannot be monitored.

Section 11.2 Definition of Serious Adverse Event; point h. Protocol-specific SAEs: first bullet point

REVISED TEXT

h. Protocol- specific SAEs:

• cuSCC (Section 8.2.2.1), new primary melanomas (Section 8.2.2.2) **and non-cutaneous malignancies (Section 8.2.2.3)** with the exception of basal cell carcinoma (BCC). BCC should be reported as an AE or SAE based on the discretion of the investigator.

Section 18.1 Hypotheses and Treatment Comparisons; 3rd paragraph

REVISED TEXT

π_j is the true response rate for histology *j,* where *j = 1,..., 9* and indexes the nine histologies. C_j is the historical control response rate for the jth histology. The historical controls vary by histology and are provided in Table 29. **The study is powered to detect a high clinically meaningful response rate and is based on the hierarchical model assessment of whether there is sufficiently high probability that π_j exceeds C_j. The posterior probability that the ORR for a given histology is greater than C_j will be computed according to the following comparison:**

Section 18.2.1 Sample Size Assumption

REVISED TEXT

Each cohort of BRAF V600E mutation-positive tumor type of a given histology will enroll a maximum of 25 subjects **in the primary analysis chort as defined in Section 5.1.** If all histologies enroll the maximum of 25 subjects, this will result in no more than 225 subjects **in the primary analysis cohort**.

Enrollment into specific histology cohorts may be halted early based on results from interim analyses incorporating emerging response data. Response data from a minimum of 5 subjects will be required in a histologic cohort before it may discontinue enrollment for futility and

response data from a minimum of 10 subjects will be required before discontinuing a histologic cohort for efficacy. **If a cohort closes early for efficacy at an interim analysis, a histology specific expansion cohort (see Section 5.2) may be opened to allow additional enrollment. At** the final analysis and after the study has been closed, a minimum of 2 subjects will be required in a histologic cohort in order to meet statistical success at the final analysis. See Section 18.3.2 for more details.

Section 18.3.1 Analysis Population

REVISED TEXT

As the design for the study calls for repeated interim analyses to evaluate the accumulating efficacy data, it is necessary to shift the focus of the analysis population for efficacy depending upon the time at which the data are being analysed. Table 30 presents the population of interest based on the timing of the analysis for both efficacy and safety; accompanying definitions are provided beneath the table.

Table 30 Definition of Population for specific analysis

		**Analysis Period**	
	**Analysis Intent**	**Interim Analyses**	**Final Analysis**
**Efficacy**	Primary	ITT/Evaluable	BRAF V600E
	Supportive	BRAF V600/Evaluable	ITT Histology-specific cohorts[a]
**Safety**	Primary	ATS	ATS

[a] Subset of the BRAF V600E population that include histologically similar subjects for cohorts that were stopped for promising efficacy; includes those from the primary analysis cohort and those from the expansion cohort, as defined in Section 5.1and Section 5.2.

The BRAF V600E Population is defined as all enrolled subjects regardless of whether or not treatment was administered, who obtain positive verification of the BRAF V600E mutation from a certified central reference laboratory.

The Intent-to-Treat (ITT) population is defined as all enrolled subjects regardless of whether or not treatment was administered.

The All-Treated Subjects (ATS) population is defined as all subjects who receive at least one dose of dabrafenib (GSK2118436) or trametinib(GSK1120212).

The Intent-to-Treat (ITT)/Evaluable population is defined as all ITT subjects in the primary analysis cohort who are also evaluable according to the evaluability defined in the interim analysis RAP.

The BRAF V600E /Evaluable Population is defined as all BRAF V600E subjects in the primary analysis cohort who are also evaluable according to the evaluability defined in the interim analysis RAP.

Section 18.3.3 Final Analysis REVISED

TEXT

The final primary analysis for ORR will occur once all subjects **in the primary analysis cohorts have either** discontinue treatment or have received treatment for at least 6 months, whichever comes first. Secondary efficacy endpoints will likely not be mature at this time, but will still be reported. Subsequent analyses for secondary endpoints will occur as follows:

- Duration of response, PFS and OS when 70% of subjects have reached a PFS event (progressed or died)
- OS when 70% of subjects have died
- Safety will be updated once the last subject has discontinued and been off all study treatment for 30 days.

These latter analyses will provide mature estimates/incidence for duration of response, PFS, OS, and safety.

Section 18.3.3.1 Safety Analysis

REVISED TEXT

Safety data will be presented in tabular and/or graphical format and summarized descriptively according to GSK's IDSL standards.

The ATS population will be used for the analysis of safety data. In general, safety data will be summarized by histology and **across all cohorts**.

Complete details of the safety analyses will be provided in the RAP.

Section 18.3.3.1.3 Other Safety Measures

REVISED TEXT

The results of scheduled assessments of vital signs, 12-lead ECG, ECHO and ECOG performance status will be summarized. Summaries will include data from scheduled assessments only. All data will be reported according to the nominal visit date for which it was recorded; no visit windows will be applied. All data will be listed.

Section 18.3.3.2 Efficacy Analysis

REVISED TEXT

ORR will be analyzed using an integrated analysis with the hierarchical model as well as analyzed independently **for each histology**.

Section 18.3.3.2 Efficacy Analysis; Table 31 Response Subcategories Define as 'Response' by Hystology

REVISED TEXT

Histology	Response Criteria	Response Subcategories defined as 'Response'
ATC, BTC, GIST, Small Intestine Adenocarcinoma	RECIST 1.1	CR, PR
NSGCT/NGGCT	RECIST 1.1.<br>Tumor Markers: β-HCG and AFP	**Marker-Negative CR, Marker-Positive CR, Marker-Negative PR, Marker-Positive PR**
MM	IMWG Uniform Response Criteria for MM	sCR, CR, PR, VGPR
HCL	Adapted from NCCN guidelines, Consensus Resolution Criteria and previous studies definition	CR±MRD, PR
WHO Grade 1 and 2 Gliomas	Response Assessment Criteria for WHO Grade 1 or 2 Gliomas: RANO Working Group	CR, PR, MR
WHO Grade 3 and 4 Gliomas	Updated Response Assessment Criteria for WHO Grade 3 or 4 Gliomas: RANO Working Group	CR, PR

Abbreviations: AFP, alpha-fetoprotein; ATC, anaplastic thyroid cancer; β-hCG, beta-human chorionic gonadotropin; BTC, biliary tract cancer; CR, complete response; GIST, gastrointestinal stromal tumor; HCL, hairy cell leukemia; IMWG, International Myeloma Working Group; MM, multiple myeloma; MR, minor response; NCCN, National Comprehensive Cancer Network; NGGCT, non-geminomatous germ cell tumor; NSGCT, non-seminomatous germ cell tumor; PR, partial response; RANO, Response Assessment for Neuro-Oncology; RECIST, Response Evaluation Criteria in Solid Tumors; sCR, stringent complete response; VGPR, very good partial response; WHO, World Health Organization

Further details on response confirmation criteria for each histological cohort will be provided in the RAP.

Bayesian inference based on summary statistics from the posterior distributions of each ORR will be reported. The posterior mean and posterior 2.5% and 97.5% percentiles of the ORR will be calculated for each histology. In addition, the posterior probability that the ORR exceeds its corresponding historical control will be reported for each histology.

Duration of response is defined **for** the subset of subjects who **have** a confirmed **response (as defined in Table 31), as** the time from first documented evidence of **response** until the first documented sign of disease progression or death. Duration of response will be summarized descriptively for each histology, if data warrant, using Kaplan-Meier medians and quartiles. Details on rules for censoring will be provided in the RAP.

Section 18.4.1 Simulation Description

REVISED TEXT

Extensive simulations have been conducted to develop and understand the performance of the adaptive design; the hierarchical model including clustering mechanism, interim monitoring, and decision criteria. **Subject enrollments into the expansion cohorts as defined in Section 5.2 are not taken into consideration in the simulations presented in this section.**

Section 20 References

REVISED TEXT

GlaxoSmithKline Document Number CM2010/00010/05. Dabrafenib Investigator's Brochure. Version 07. Report dated 09-Oct-2015.

Long GV et al. Dabrafenib and trametinib versus dabrafenib and placebo for Val600 BRAF-mutant melanoma: a multicentre, double-blind, phase 3 randomised controlled trial. Lancet. 2015; 386(9992): 444-51

Robert C et al. Improved overall survival in melanoma with combined dabrafenib and trametinib. N Engl J Med. 2015; 372(1): 30 –9

AMENDMENT 07

This amendment applies to all study sites. Bolded text indicates revised text and strike through indicates deleted text.

Amendment Summary of Main Changes:

Section(s)	Change	Rationale
Sponsor signatory	Change of sponsor signatory	Change in study sponsor from GSK to Novartis
Multiple	Delete or replace references to GlaxoSmithKline or its	To align with the change of sponsorship from GSK to Novartis.

Section(s)	Change	Rationale
	staff with that of Novartis and its authorized agents	
Multiples	Change “medical monitor” to “Medical Lead”	To align with Novartis processes
Multiple	Make administrative changes	To align with the change of sponsorship from GSK to Novartis.

Amendment Details:

Section: Title Page:

Text changed:

The title page replaced as per Novartis requirements.

Reason for change:

Change in study sponsorship and to bring into line with Novartis processes.

Section: Sponsor Information Page

Text changed:

The GSK contact information has been replaced with Novartis details.

The term medical monitor has been replaced by Medical Lead and the email for Medical Lead provided.

Reason for change:

Change in study sponsorship and to bring into line with Novartis processes.

LIST OF ABBREVIATIONS

Text changed:

CRO Clinical Research Organization
GCPH Global Clinical Program Head
HA Health Authority

Reason for change:

Change in study sponsorship and to bring into line with Novartis processes.

Section: 7.4.10.2. Pregnancy Reporting

Text changed:

To ensure subject safety, each pregnancy must be reported to the sponsor within **24 hours** of learning of its occurrence.

Reason for change:

Change in study sponsorship and to bring into line with Novartis processes.

Section: 7.4.10.4. Action to be taken if pregnancy occurs in a female partner of a male study subject

Text changed:

The investigator will record pregnancy information on the appropriate form and submit it to the sponsor within **24 hours** of learning of the partner's pregnancy.

Reason for change:

Change in study sponsorship and to bring into line with Novartis processes.

Section: 11.7. Time Period and Frequency of Detecting AEs and SAEs

Text changed:

Reason for change:

Change in study sponsorship and to bring into line with Novartis processes.

Section: 11.13. Regulatory Reporting Requirements for SAEs

Text changed:

An investigator who receives an investigator safety report describing a SAE(s) or other specific safety information (e.g., summary or listing of SAEs) from the sponsor will file it with the IB and will notify the **IRB/IEC**, if appropriate according to local requirements.

Reason for change:

Change in study sponsorship and to bring into line with Novartis processes.

Section: 17. DATA MANAGEMENT

Text changed:

Adverse events (AEs) and concomitant medications terms will be coded using Medical Dictionary for Regulatory Activities (MedDRA) and **a custom drug dictionary**-.

-Reason for change:

Change in study sponsorship and to bring into line with Novartis processes.

Section: 19.4. Quality Control (Study Monitoring)

Text changed:

In accordance with applicable regulations, GCP, and **Novartis** procedures, **Novartis personnel (or designated Clinical Research Organization [CRO]) will contact** the site prior to the start of the study to review with the site staff the protocol, study requirements, and their responsibilities to satisfy regulatory, ethical, and the sponsor's requirements.

Reason for change:

Change in study sponsorship and to bring into line with Novartis processes.

Section: 19.6. Study and Site Closure

Text changed:

Upon completion or termination of the study, **Novartis personnel (or designated CRO)** will conduct site closure activities with the investigator or site staff (as appropriate), in accordance with applicable regulations, ICH GCP, and **Novartis** Standard Operating Procedures.

Reason for change:

Change in study sponsorship and to bring into line with Novartis processes.

Section: 19.7. Records Retention

Text changed:

Essential documents (written and electronic) should be retained for a period of not less than fifteen (15) years from the completion of the Clinical Trial unless the Sponsor provides written permission to dispose of them or, requires their retention for an additional period of time because of applicable laws, regulations and/or guidelines.

-Reason for change:

Change in study sponsorship and to bring into line with Novartis processes.

Section: 19.8. Provision of Study Results to Investigators, Posting of Information on Publicly Available Clinical Trials Registers and Publication

Text changed:

Novartis aims to post a results summary to the Novartis Clinical Trial Results website (www.novartisclinicaltrials.com) and other publicly available registers no later than twelve (12) months after the last subject's last visit (LSLV). In addition, upon study completion and finalization of study report, Novartis aims to submit results of the study for publication.

-When publication is not feasible, **please refer to the Novartis Clinical Trial Results website (www.novartisclinicaltrials.com) for a summary of the trial** results .-
