# Supplementary material for: Dabrafenib plus trametinib in BRAFV600E-mutated rare cancers: the phase 2 ROAR trial
Source: Nat Med. 2023 Apr 14;29(5):1103–12. doi: 10.1038/s41591-023-02321-8 (PMC10202803; doi:10.1038/s41591-023-02321-8)
Supplement: Supplementary file 2 — Reporting Summary [file 41591_2023_2321_MOESM2_ESM.pdf]

nature portfolio

Corresponding author(s): Vivek Subbiah; NMED-A123694

Last updated by author(s): 21 March, 2023

Reporting Summary

Nature Portfolio wishes to improve the reproducibility of the work that we publish. This form provides structure for consistency and transparency in reporting. For further information on Nature Portfolio policies, see our Editorial Policies and the Editorial Policy Checklist.

Statistics

For all statistical analyses, confirm that the following items are present in the figure legend, table legend, main text, or Methods section.

n/a	Confirmed	
☐	☒	The exact sample size (*n*) for each experimental group/condition, given as a discrete number and unit of measurement
☐	☒	A statement on whether measurements were taken from distinct samples or whether the same sample was measured repeatedly
☐	☒	The statistical test(s) used AND whether they are one- or two-sided<br>*Only common tests should be described solely by name; describe more complex techniques in the Methods section.*
☐	☒	A description of all covariates tested
☐	☒	A description of any assumptions or corrections, such as tests of normality and adjustment for multiple comparisons
☐	☒	A full description of the statistical parameters including central tendency (e.g. means) or other basic estimates (e.g. regression coefficient) AND variation (e.g. standard deviation) or associated estimates of uncertainty (e.g. confidence intervals)
☐	☒	For null hypothesis testing, the test statistic (e.g. *F*, *t*, *r*) with confidence intervals, effect sizes, degrees of freedom and *P* value noted<br>*Give P values as exact values whenever suitable.*
☐	☒	For Bayesian analysis, information on the choice of priors and Markov chain Monte Carlo settings
☒	☐	For hierarchical and complex designs, identification of the appropriate level for tests and full reporting of outcomes
☒	☐	Estimates of effect sizes (e.g. Cohen's *d*, Pearson's *r*), indicating how they were calculated

Our web collection on statistics for biologists contains articles on many of the points above.

Software and code

Policy information about availability of computer code

Data collection	Designated Investigator staff entered the information required by the protocol into the Electronic Case Report Forms (eCRF). The eCRFs were built using fully validated secure web-enabled software that conforms to 21 CFR Part 11 requirements. Automatic validation programs checked for data discrepancies in the eCRFs and allowed modification or verification of the entered data by the Investigator staff. The Investigator verified that the data entered into the CRFs was complete and accurate.
Data analysis	SAS (version 9.3; SAS Institute) was used for all statistical analysis except for the hierarchical Bayesian model, which used C++ and R version 2.15.2 to evaluate the performance of the design under various assumptions for the distribution of true ORRs across histological cohorts and accounting for anticipated small sample sizes. All the data analysis were performed according study protocol and statistical analysis plan.

For manuscripts utilizing custom algorithms or software that are central to the research but not yet described in published literature, software must be made available to editors and reviewers. We strongly encourage code deposition in a community repository (e.g. GitHub). See the Nature Portfolio guidelines for submitting code & software for further information.

Data

Policy information about availability of data

All manuscripts must include a data availability statement. This statement should provide the following information, where applicable:

- Accession codes, unique identifiers, or web links for publicly available datasets
- A description of any restrictions on data availability
- For clinical datasets or third party data, please ensure that the statement adheres to our policy

Novartis is committed to sharing with qualified external researchers access to patient-level data and supporting clinical documents from eligible studies. These requests are reviewed and approved by an independent review panel based on scientific merit. All data provided are anonymized to respect the privacy of patients who have participated in the trial, in line with applicable laws and regulations. This trial data availability is according to the criteria and process described on www.clinicalstudydatarequest.com. The authors declare that all data supporting the findings of this study are available within the article and its supplementary information files.

Human research participants

Policy information about studies involving human research participants and Sex and Gender in Research.

Reporting on sex and gender	The study recruited male and female adult patients. Most patients in the study were males (56%). No other gender specific analyses were done.
Population characteristics	Eligible patients were aged ≥18 years with histologically confirmed BRAFV600E mutation-positive advanced tumor with no standard treatment options and ECOG performance status score ≤2. Patients received oral dabrafenib (150 mg twice daily) and oral trametinib (2 mg once daily) on a continuous dosing schedule until unacceptable toxicity, disease progression, or death.
Recruitment	The patients with histologically confirmed BRAFV600E mutation-positive advanced tumor who meet the eligibility criteria were subsequently enrolled and assigned a patient ID. Study inclusion and exclusion criteria (In the methods section) clearly describe the study population and how a patient was selected. Some factors may have led to bias and impacted the study results. These include (1) Sample size in some historical cohorts are very small, which may not represent the true population in these cohorts; (2) Prior treatments may impact the study results; (3) Histological subtypes may have been recruited disproportionately in certain cohorts and these may respond differently to treatment.
Ethics oversight	The study was conducted in compliance with ICH Good Clinical Practice guidelines and ethical principles described in the Declaration of Helsinki. The study protocol and all amendments were reviewed by the Independent Ethics Committee (IEC) or Institutional Review Board (IRB) for each participating study center. Study centers include: AKH Wien-Vienna (Vienna, Austria), Universitaetsklinik Innsbruck-Innsbruck (Vienna, Austria), LKH Salzburg-Salzburg (Vienna, Austria), Krankenhaus der Elisabethinen Linz-Linz (Vienna, Austria), UZ Brussel-Brussel (Brussel, Belgium), Princess Margaret Hospital Toronto (Ontario, Canada), Rigshospitalet Onkologisk Afdeling, Fase 1 Enhed (Hillerød, Denmark), Institut Cancerologie de l'Ouest - Rene Gauducheau-Saint- Herblain cedex (Saint-Herblain, France), Institut de Cancerologie Gustave Roussy (Lyon, France), Institut Claudius Regaud - Toulouse cedex 9 (France), Centre Leon Berard (Lyon Cedex 08, France), Institut Bergonie (Lyon Cedex 08, France), CHU de Nantes - Hotel Dieu, Service Hematologie Clinique (Lyon Cedex 08, France), Universitaetsklinikum Freiburg-Inner-Freiburg (Freiburg im Breisgau, Germany), Universitaetsklinikum Mannheim GmbH-Haematologie-Mannheim (Tubingen, Germany), Universitaetsklinikum Eppendorf-II. Med. Klinik- Hamburg (Tubingen, Germany), Charite-Campus Virchow Klinikum-Onkologie (Berlin, Germany), Universitaetsklinikum Heidelberg-Medizinische Klinik V (Tubingen, Germany), Istituto Europeo di Oncologia (IRCCS) di Milano (Milano, Italy), Istituto Nazionale Tumori- Milano-Italy (Milano, Italy), Ospedale San Raffaele IRCCS-Milano-Italy (Milano, Italy), Seoul National University Hospital (Seoul, Korea), Gangnam Severance Hospital-Seoul-Korea (Seoul, Korea), The Netherlands Cancer Institute-Amsterdam (Amsterdam, Netherlands), UMC Utrecht (Amsterdam, Netherlands), Erasmus MC Rotterdam (Amsterdam, Netherlands), VU Medisch Centrum-Amsterdam (Amsterdam, Netherlands), REK Sør-Øst (Oslo, Norway), Dana Farber Cancer Institute (Massachusetts, United States), Sarah Cannon Cancer Center (Washington, United States), National Cancer Institute of Health (Maryland, United States), MD Anderson Cancer Center (Texas, United States), UCLA-Santa Monica (California, United States), University of Arkansas for Medical Sciences-Little Rock (Arkansas, United States), New York University Medical Center (New York, United States), Karolinska Universitetssjukhuset Solna (Solna, Sweden), Hospital Universitario Valle de Hebron (Barcelona, Spain), University Hospital 12 de Octubre (Madrid, Spain), National Cancer Center Hospital-Tokyo (Tokyo, Japan), National Cancer Center Hospital East-Chiba (Chiba, Japan).

Note that full information on the approval of the study protocol must also be provided in the manuscript.

Field-specific reporting

Please select the one below that is the best fit for your research. If you are not sure, read the appropriate sections before making your selection.

☒ Life sciences ☐ Behavioural & social sciences ☐ Ecological, evolutionary & environmental sciences

For a reference copy of the document with all sections, see nature.com/documents/nr-reporting-summary-flat.pdf

Life sciences study design

All studies must disclose on these points even when the disclosure is negative.

Sample size	Each cohort of BRAF V600E mutation-positive tumor type of a given histology was planned to enroll a maximum of 25 subjects in the primary analysis cohort.<br>Enrollment into specific histology cohorts was planned to be halted early based on results from quarterly performed interim analyses incorporating emerging response data. Response data from a minimum of five subjects was required in a histologic cohort before it discontinued enrollment for futility and response data from a minimum of 10 subjects was required before discontinuing a histologic cohort for efficacy. If any cohort closed early for efficacy at an interim analysis, a histology specific expansion cohort was planned to be opened to allow additional enrollment. At the final analysis and after the study had been closed, a minimum of two subjects in the primary analysis cohort were required in a histologic cohort in order to meet statistical success.<br>Simulation studies were conducted to evaluate the performance of the design under various assumptions for the distribution of true ORRs across the histologic cohorts and accounting for the anticipated small sample sizes. Operating characteristics including power, type I error, estimation of the ORR, and the probability of halting enrollment at interim analyses were assessed.<br>When the treatment effects are similar across all histologies, the design maintains power 84% to 98% and type I error rate ≤ 0.04.
Data exclusions	No data exclusion
Replication	The inclusion of specific tumor histologies supported the reproducibility of the study results. In the manuscript, we have compared the results of our study with those in the NCI-MATCH and other available evidence in literature.
Randomization	This was a single-arm, open-label study.
Blinding	The study was designed to be an open-label study. Given the rarity of the tumor types included in the study, it is challenging to design a randomized study for the included cohorts.

Reporting for specific materials, systems and methods

We require information from authors about some types of materials, experimental systems and methods used in many studies. Here, indicate whether each material, system or method listed is relevant to your study. If you are not sure if a list item applies to your research, read the appropriate section before selecting a response.

Materials & experimental systems

n/a	Involved in the study	
☒	☐	Antibodies
☒	☐	Eukaryotic cell lines
☒	☐	Palaeontology and archaeology
☒	☐	Animals and other organisms
☐	☒	Clinical data
☒	☐	Dual use research of concern

Methods

n/a	Involved in the study	
☒	☐	ChIP-seq
☐	☒	Flow cytometry
☒	☐	MRI-based neuroimaging

Clinical data

Policy information about clinical studies

All manuscripts should comply with the ICMJE guidelines for publication of clinical research and a completed CONSORT checklist must be included with all submissions.

Clinical trial registration	The study is registered in clinicaltrials.gov as NCT02034110 and EudraCT as 2013-001705-87.
Study protocol	Full study protocol is available in supplementary materials supporting this article.
Data collection	The study was conducted at 27 community and academic cancer centers in 13 countries (Austria, Belgium, Canada, France, Germany, Italy, Japan, the Netherlands, Norway, South Korea, Spain, Sweden, and the United States). The enrollment centers were either large commercial centers or academic institutions such as universities that specialize in cancer treatment. The study centers include: AKH Wien-Vienna (Vienna, Austria), Universitaetsklinik Innsbruck-Innsbruck (Vienna, Austria), LKH Salzburg-Salzburg (Vienna, Austria), Krankenhaus der Elisabethinen Linz-Linz (Vienna, Austria), UZ Brussel-Brussel (Brussel, Belgium), Princess Margaret Hospital Toronto (Ontario, Canada), Rigshospitalet Onkologisk Afdeling, Fase 1 Enhed (Hillerød, Denmark), Institut Cancerologie de l'Ouest - Rene Gauducheau-Saint- Herblain cedex (Saint-Herblain, France), Institut de Cancerologie Gustave Roussy (Lyon, France), Institut Claudius Regaud - Toulouse cedex 9 (France), Centre Leon Berard (Lyon Cedex 08, France), Institut Bergonie (Lyon Cedex 08, France), CHU de Nantes - Hotel Dieu, Service Hematologie Clinique (Lyon Cedex 08, France), Universitaetsklinikum Freiburg-Inner-Freiburg (Freiburg im Breisgau, Germany), Universitaetsklinikum Mannheim GmbH-Haematologie-Mannheim (Tubingen, Germany), Universitaetsklinikum Eppendorf-II. Med. Klinik- Hamburg (Tubingen, Germany), Charite-Campus Virchow Klinikum-Onkologie (Berlin, Germany), Universitaetsklinikum Heidelberg-Medizinische Klinik V (Tubingen, Germany), Istituto Europeo di Oncologia (IRCCS) di Milano (Milano, Italy), Istituto Nazionale Tumori- Milano-Italy (Milano, Italy), Ospedale San Raffaele IRCCS-Milano-Italy (Milano, Italy), Seoul National University Hospital (Seoul, Korea), Gangnam Severance Hospital-Seoul-Korea (Seoul, Korea), The Netherlands Cancer

	Institute-Amsterdam (Amsterdam, Netherlands), UMC Utrecht (Amsterdam, Netherlands), Erasmus MC Rotterdam (Amsterdam, Netherlands), VU Medisch Centrum-Amsterdam (Amsterdam, Netherlands), REK Sør-Øst (Oslo, Norway), Dana Farber Cancer Institute (Massachusetts, United States), Sarah Cannon Cancer Center (Washington, United States), National Cancer Institute of Health (Maryland, United States), MD Anderson Cancer Center (Texas, United States), UCLA-Santa Monica (California, United States), University of Arkansas for Medical Sciences-Little Rock (Arkansas, United States), New York University Medical Center (New York, United States), Karolinska Universitetssjukhuset Solna (Solna, Sweden), Hospital Universitario Valle de Hebron (Barcelona, Spain), University Hospital 12 de Octubre (Madrid, Spain), National Cancer Center Hospital-Tokyo (Tokyo, Japan), National Cancer Center Hospital East-Chiba (Chiba, Japan).<br>Designated Investigator staff entered the information required by the protocol into the Electronic Case Report Forms (eCRF). The eCRFs were built using fully validated secure web-enabled software that conforms to 21 CFR Part 11 requirements. Automatic validation programs checked for data discrepancies in the eCRFs and allowed modification or verification of the entered data by the Investigator staff. The Investigator verified that the data entered into the CRFs was complete and accurate.<br>The study recruited patients from March 2014 to December 2021.
Outcomes	Primary endpoint<br>The primary endpoint was tumor response as assessed by the investigator and defined by RECIST version 1.1 for solid tumor histologies including ATC, BTC, GIST, ASI, and NSGCT/NGGCT, the RANO criteria for LGG, and modified RANO for HGG. Response was also assessed centrally by independent radiology review. Tumor responses in MM were defined by the International Myeloma Working Group (IMWG) Uniform Response Criteria, and those for HCL were adapted from the National Comprehensive Cancer Network Clinical Practice Guidelines in Oncology for HCL, consensus resolution criteria and previous studies definition.<br><br>Secondary endpoints<br>The secondary endpoints included DoR (defined as the time from the first documented evidence of CR or PR until documented disease progression or death from any cause), PFS (defined as the time from the first dose to disease progression or death from any cause, whichever occurred earlier), OS (defined as the time from the first dose of the study drug until death from any cause), and safety as assessed by the investigator. Safety assessments included change from baseline in physical examination findings, vital signs, AEs, laboratory values, and cardiac assessments.

Flow Cytometry

Plots

Confirm that:

- ☐ The axis labels state the marker and fluorochrome used (e.g. CD4-FITC).
- ☐ The axis scales are clearly visible. Include numbers along axes only for bottom left plot of group (a 'group' is an analysis of identical markers).
- ☐ All plots are contour plots with outliers or pseudocolor plots.
- ☐ A numerical value for number of cells or percentage (with statistics) is provided.

Methodology

Sample preparation	*Describe the sample preparation, detailing the biological source of the cells and any tissue processing steps used.*
Instrument	*Identify the instrument used for data collection, specifying make and model number.*
Software	*Describe the software used to collect and analyze the flow cytometry data. For custom code that has been deposited into a community repository, provide accession details.*
Cell population abundance	*Describe the abundance of the relevant cell populations within post-sort fractions, providing details on the purity of the samples and how it was determined.*
Gating strategy	*Describe the gating strategy used for all relevant experiments, specifying the preliminary FSC/SSC gates of the starting cell population, indicating where boundaries between "positive" and "negative" staining cell populations are defined.*

☐ Tick this box to confirm that a figure exemplifying the gating strategy is provided in the Supplementary Information.
